# Crystal structures of three anionic lanthanide–aluminium [3.3.1] metallacryptate complexes

**DOI:** 10.1107/S2056989020010725

**Published:** 2020-08-14

**Authors:** Rachel E. Rheam, Matthias Zeller, Curtis M. Zaleski

**Affiliations:** a Department of Chemistry and Biochemistry, Shippensburg University, Shippensburg, PA 17257, USA; b Department of Chemistry, Purdue University, West Lafayette, IN 47907, USA

**Keywords:** metallacrown, metallacryptate, aluminium complex, crystal structure

## Abstract

The three isomorphous [3.3.1] metallacryptate complexes bis­(pyridinium) di­aqua­dipyridine­hexa­kis­[μ_3_-salicyl­hydroximato(3−)]bis­[μ_2_-salicyl­hydroxim­ato(1−)]hexa­aluminiumgadolinium–pyridine–water (1/7.396/1), (C_5_H_6_N)_2_[GdAl_6_(C_7_H_6_NO_3_)_2_(C_7_H_4_NO_3_)_7_(C_5_H_5_N)_1.855_(H_2_O)_2_]·7.396C_5_H_5_N·H_2_O or [Hpy]_2_[GdAl_6_(H_2_shi)_2_(shi)_7_(py)_1.855_(H_2_O)_2_]·7.396py·H_2_O, **1**, bis­(pyridinium) di­aqua­dipyridine­hexa­kis­[μ_3_-salicyl­hydroximato(3−)]bis­[μ_2_-salicyl­hydroxim­ato(1−)]hexa­aluminiumdysprosium–pyridine–water (1/7.429/1), (C_5_H_6_N)_2_[DyAl_6_(C_7_H_6_NO_3_)_2_(C_7_H_4_NO_3_)_7_(C_5_H_5_N)_1.855_(H_2_O)_2_]·7.429C_5_H_5_N·H_2_O or [Hpy]_2_[DyAl_6_(H_2_shi)_2_(shi)_7_(py)_1.891_(H_2_O)_2_]·7.429py·H_2_O, **2**, and bis­(pyrid­in­ium) di­aqua­dipyridine­hexa­kis­[μ_3_-salicyl­hydroximato(3−)]bis­[μ_2_-salicyl­hydrox­imato(1−)]hexa­aluminiumytterbium–pyridine–water (1/7.386/1), (C_5_H_6_N)_2_[YbAl_6_(C_7_H_6_NO_3_)_2_(C_7_H_4_NO_3_)_7_(C_5_H_5_N)_1.855_(H_2_O)_2_]·7.429C_5_H_5_N·H_2_O or [Hpy]_2_[YbAl_6_(H_2_shi)_2_(shi)_7_(py)_1.818_(H_2_O)_2_]·7.386py·H_2_O, **3**, where Hpy^+^ is pyridinium, shi^3−^ is salicyl­hydroximate, and py is pyridine, consist of an aluminium-based metallacryptand that captures an *Ln* ^III^ ion in the central cavity. The metallacryptand portions are comprised of an Al—N—O repeat unit; thus, they can be considered three-dimensional metallacrowns. The encapsulated *Ln* ^III^ ions are nine-coordinate with a spherical capped-square-anti­prism geometry, while the six Al^III^ ions are all octa­hedral. Four of the Al^III^ ions are chiral centers with 2 Δ and 2 Λ stereoconfigurations. The remaining two Al^III^ ions have *trans* chelate rings from two different shi^3−^ ligands. For **1**–**3**, a section of the main mol­ecule is disordered induced by the presence or absence of a pyridine ligand coordinated to one of the Al^III^ ions. In the absence of the pyridine moiety, an H_2_shi^−^ ligand moves into the space otherwise occupied by the pyridine and the phenol oxygen atom coordinates to the Al^III^ ion. The movement of the H_2_shi^−^ ligand induces movement for the *Ln* ^III^ ion, for another Al^III^ ion that also binds the same H_2_shi^−^ ligand, and for one of the shi^3−^ ligands coordinated to the latter Al^III^ ion. For **1**–**3** the occupancy ratio of the metallacryptand portions refined to 0.8550 (13):0.1450 (13), to 0.8909 (13):0.1091 (13), and to 0.8181 (14):0.1819 (14), respectively.

## Abstract 2

The three [3.3.1] metallacryptate complexes [Hpy]_2_[GdAl_6_(H_2_shi)_2_(shi)_7_(py)_1.855_(H_2_O)_2_]·7.396py·H_2_O, **1**, [Hpy]_2_[DyAl_6_(H_2_shi)_2_(shi)_7_(py)_1.891_(H_2_O)_2_]·7.429py·H_2_O, **2**, and [Hpy]_2_[YbAl_6_(H_2_shi)_2_(shi)_7_(py)_1.818_(H_2_O)_2_]·7.386py·H_2_O, **3**, where Hpy^+^ is pyridinium, shi^3−^ is salicyl­hydroximate, and py is pyridine, consist of an aluminium-based metallacryptand that captures an *Ln* ^III^ ion in the central cavity. The metallacryptand portions are comprised of an Al—N—O repeat unit; thus, they can be considered three-dimensional metallacrowns. The encapsulated *Ln* ^III^ ions are nine-coordinate with a spherical capped-square-anti­prism geometry, while the six Al^III^ ions are all octa­hedral. Four of the Al^III^ ions are chiral centers with 2 Δ and 2 Λ stereoconfigurations. The remaining two Al^III^ ions have *trans* chelate rings from two different shi^3−^ ligands.

## Chemical context   

Since the first report of metallacrowns (MC) in 1989, the chemistry of these mol­ecules has grown to include a number of different structural types and subsequent applications (Pecoraro, 1989 ▸). The first and archetypal metallacrowns are based on a macrocyclic metal–nitro­gen–oxygen repeat unit and these mol­ecules typically bind a metal ion in the central cavity similar to crown ethers (Pecoraro *et al.*, 1997 ▸). However, the structural variation has expanded to include mol­ecules such as aza­metallacrowns with a metal–nitro­gen–nitro­gen repeat, collapsed MCs – structures without a central cavity and metal ion, inverse MCs that bind a non-metal atom in the central cavity, and MC-like metallacryptands – complexes with an *M*–N–O repeat unit in a three-dimensional pattern (Mezei *et al.*, 2007 ▸). As structures have become more varied, so have the properties and applications of the mol­ecules. Potential applications include single-mol­ecule magnetism, magnetorefrigeration, MRI contrast agents, host–guest complexes, gas- and solvent-sorption materials, and optical imaging agents (Nguyen & Pecoraro, 2017 ▸; Lutter *et al.*, 2018*b* ▸; Pavlishchuk *et al.*, 2017 ▸; Atzeri *et al.*, 2016 ▸). The properties of the MCs are derived from the inter­play of the central metal ion and the ring metal ions. Often a transition metal is used for the ring metal ions, while either a lanthanide or transition-metal ion is captured in the central cavity. The use of lanthanide ions in particular can yield inter­esting single-mol­ecule magnet or luminescent properties with the correct choice of the ring metal. For instance, the combination of paramagnetic Dy^III^ and Mn^III^ ions leads to MCs with single-mol­ecule magnet properties (Zaleski *et al.*, 2004 ▸, 2007 ▸; Boron *et al.*, 2016 ▸; Cao *et al.*, 2016 ▸), while the use of Zn^II^ or Ga^III^ in combination with *Ln* ^III^ ions can lead to luminescent MCs as the closed-shell electron configuration of the ring ions does not quench the radiation emitted by the *Ln* ^III^ ions (Jankolovits *et al.*, 2011 ▸; Chow *et al.*, 2016 ▸; Martinić *et al.*, 2017 ▸). Indeed, several *Ln* ^III^–Ga^III^ MCs have garnered attention as optical imaging agents (Nguyen *et al.*, 2018 ▸; Lutter *et al.*, 2018*a* ▸, 2019 ▸, 2020 ▸).

To better understand the properties of the central *Ln* ^III^ ion in an MC framework (*M*–N–O repeat unit), we sought to isolate the *Ln* ^III^ ion from paramagnetic ring metal ions as these ions complicate the magnetism of the complexes and quench any luminescence. Thus, any magnetic or spectroscopic properties would be that of the *Ln* ^III^ ion inside an MC ligand environment. One suitable metal is aluminum(III) as the charge of this ion should allow substitution for Mn^III^ and Ga^III^ ions while maintaining overall mol­ecular charge balance. In addition, aluminum has not been explored in MC chemistry in detail as only two other aluminum-based metallacryptates have been reported to date (Travis *et al.*, 2020 ▸). Herein we present three [3.3.1] metallacryptate complexes [Hpy]_2_[GdAl_6_(H_2_shi)_2_(shi)_7_(py)_1.855_(H_2_O)_2_]·7.396py·H_2_O, **1**, [Hpy]_2_[DyAl_6_(H_2_shi)_2_(shi)_7_(py)_1.891_(H_2_O)_2_]·7.429py·H_2_O, **2**, and [Hpy]_2_[YbAl_6_(H_2_shi)_2_(shi)_7_(py)_1.818_(H_2_O)_2_]·7.386py·H_2_O, **3**, where Hpy^+^ is pyridinium, shi^3−^ is salicyl­hydroximate, and py is pyridine. Complexes **1**–**3**, which are isomorphous, differ from the previous aluminum-based metallacryptates in that **1**–**3** are discrete mol­ecules, while the latter structures are two-dimensional networks of metallacryptates. Future studies will investigate the magnetic properties of **1**–**3** to understand the behavior of the *Ln* ^III^ ions in a metallacrown-like framework.

## Structural commentary   

**Figure 1:**
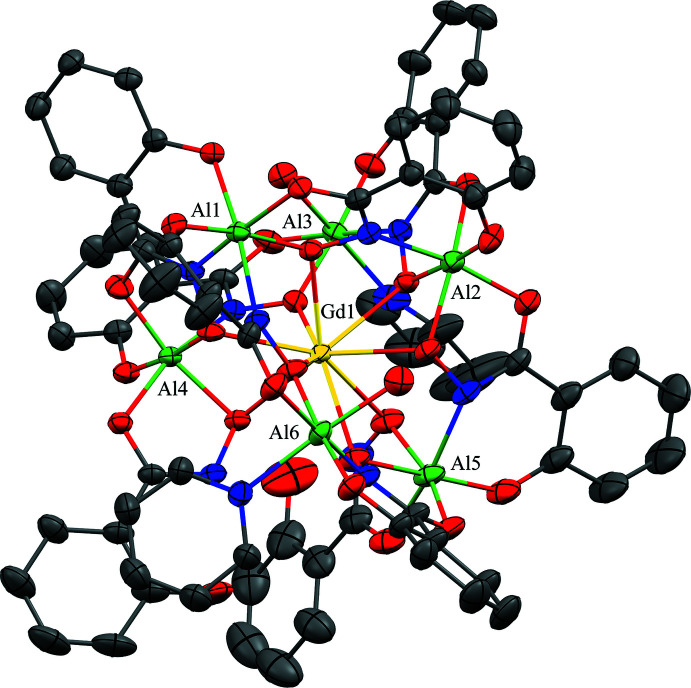
The single-crystal X-ray structure of [Hpy]_2_[GdAl_6_(H_2_shi)_2_(shi)_7_(py)_1.855_(H_2_O)_2_]·7.396py·H_2_O, **1**, with displacement ellipsoids at the 50% probability level. For clarity, only the metal ions have been labeled, and the lattice pyridinium cations, the lattice water and pyridine mol­ecules, the hydrogen atoms, and disorder have been omitted. Color scheme: green – Al, yellow – Gd, red – oxygen, blue – nitro­gen, and gray – carbon. All figures were generated with the program *Mercury* (Macrae *et al.*, 2020 ▸).

The structures of **1**–**3** are isomorphous with varying degrees of disorder in the metallacryptate and amount of lattice pyridine mol­ecules (Figs. 1 ▸–3 ▸ ▸). The overall structure of the complexes is akin to that of a [3.3.1] cryptand, where the numbers indicate the number of ether oxygen atoms in each carbon–oxygen chain between the nitro­gen atoms (Lehn, 1978 ▸; Krakowiak *et al.*, 1993 ▸).
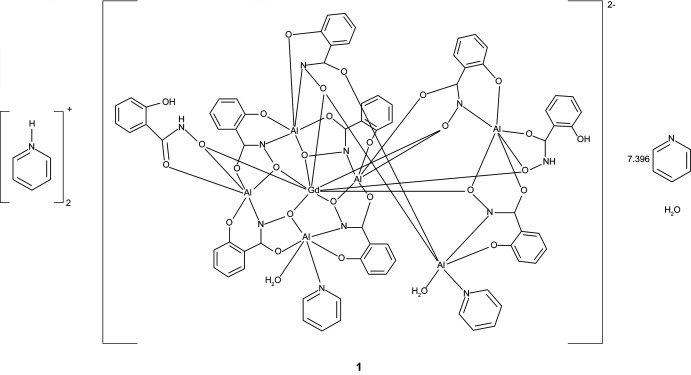
 
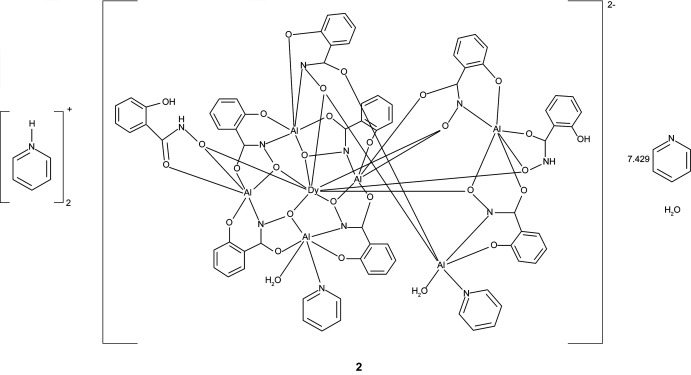
 
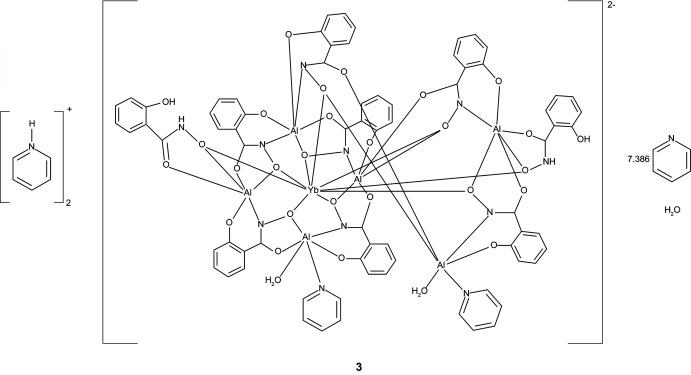


**Figure 4:**
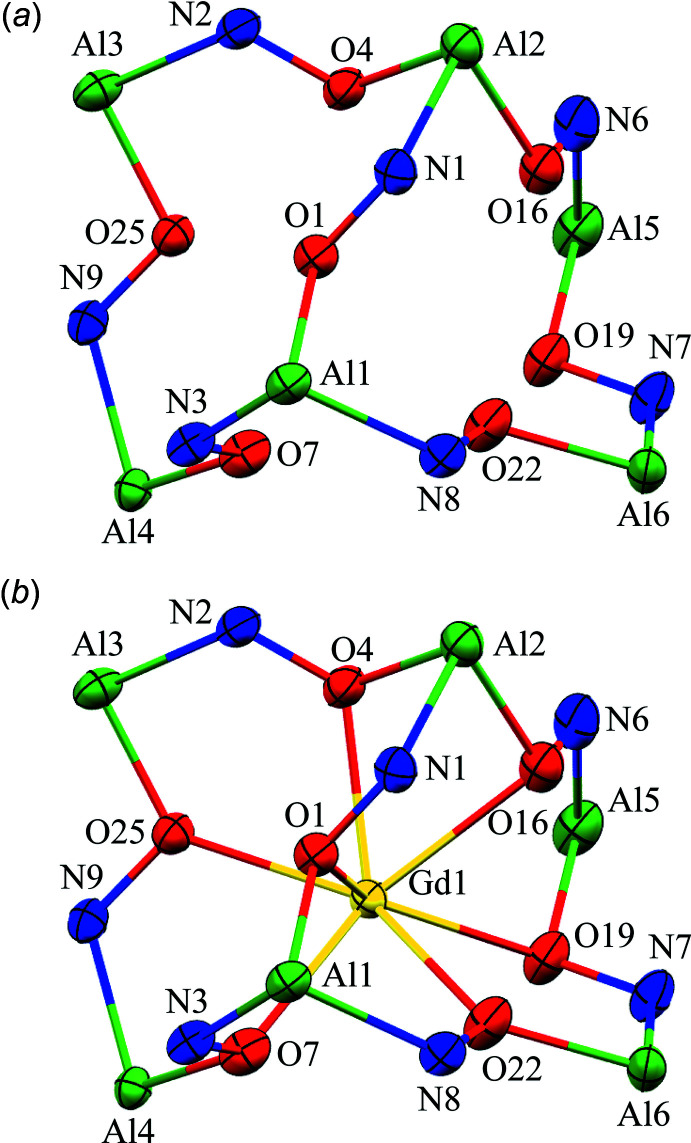
The (*a*) metallacryptand and (*b*) metallacryptate views of **1** highlighting the [3.3.1] connectivity between the metal ions. See Fig. 1 ▸ for additional display details.

In **1**–**3**, the [3.3.1] nomenclature is derived from the number of oxygen atoms in each Al–N–O chain between the anchoring Al^III^ ions (Fig. 4 ▸). Al1 and Al2 are analogous to the nitro­gen atoms of a cryptand, while the remaining Al^III^ ions form the metallacryptand. There are two longer O–N–Al–O–N–Al–O–N linkages and one shorter N–O linkage between Al1 and Al2. The metal–nitro­gen–oxygen repeat unit of each chain between Al1 and Al2 is that of an archetypal metallacrown; thus, the Al–N–O chains of **1**–**3** can be considered three-dimensional metallacrowns, *i.e.* metallacryptands. As these complexes bind a metal ion in the central cavity, they are more accurately described as metallacryptates just as the term cryptates is used to describe cryptands that bind a central guest. Each metallacryptate unit consists of one Ln^III^ ion and six Al^III^ ions (total charge of 21+) and of seven triply deprotonated shi^3−^ ligands and two H_2_shi^−^ (total charge of 23-). The metallacryptate dianion is charge-balanced by the presence of two pyridinium ions (total 2+ charge) in the lattice. The hydroximate groups of the seven shi^3−^ ligands provide the N–O linkages for the metallacryptand and the oxime oxygen atoms coordinate the central *Ln* ^III^ ion. The two remaining H_2_shi^−^ ligands also bind with their oxime oxygen to the central *Ln* ^III^ ion but do not participate in the metallacryptand shell. For the H_2_shi^−^ ligands, the oxime oxygen atoms are deprotonated, while the oxime nitro­gen and phenolate oxygen atoms remain protonated. As the oxime nitro­gen atoms are protonated, they do not bind to Al^III^ ions and are not involved in the N–O topology, although the H_2_shi^−^ ligands do serve to bridge the central *Ln* ^III^ to the Al^III^ ions. Beyond the overall mol­ecular charge considerations, the oxidation state assignments of the *Ln* ^III^ and Al^III^ ions are also confirmed by bond-valence-sum (BVS) values (Table 1 ▸; Brese & O’Keeffe, 1991 ▸; Trzesowska *et al.*, 2004 ▸).

For **1**–**3**, a section of the main mol­ecule is disordered induced by presence or absence of a pyridine ligand coordinated to Al6. In the absence of the pyridine moiety, an H_2_shi^−^ ligand (associated with N4) moves into the space otherwise occupied by the pyridine and the ligand’s protonated phenol oxygen atom coordinates to Al6. When the pyridine is bound to Al6, the phenol oxygen atom remains uncoordinated and hydrogen bonded to to a lattice pyridine mol­ecule (associated with N15). The movement of the H_2_shi^−^ ligand induces movement for the *Ln* ^III^ ion, for Al4, which also binds the same H_2_shi^−^ ligand, and for one of the shi^3−^ ligands (associated with N9) coordinated to Al4. For **1**–**3** the occupancy ratio of the metallacryptand portions refined to 0.8550 (13):0.1450 (13), to 0.8909 (13):0.1091 (13), and to 0.8181 (14):0.1819 (14), respectively. The structural description below will focus on the major moiety of each complex. A full description of disorder treatment can be found in the *Refinement* section.

**Figure 5:**
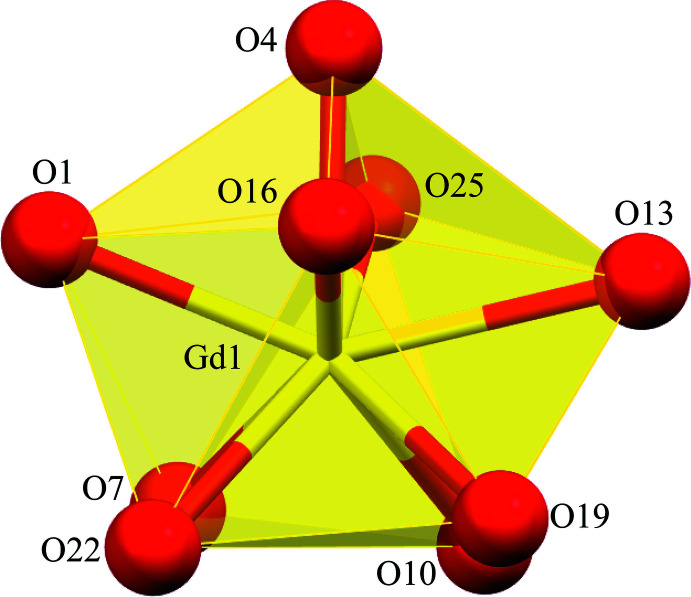
Polyhedral view of the spherical capped square anti­prism coordination geometry for Gd1 of **1**. See Fig. 1 ▸ for additional display details.

Each *Ln^I^* ^II^ ion is nine-coordinate with seven of the oxime oxygen atoms provided by the seven shi^3−^ ligands, which participate in the formation of the metallacryptand and form bridges to all six Al^III^ ions, and the two remaining oxime oxygen atoms are provided by the two H_2_shi^−^ ligands. Each oxime oxygen atom of the H_2_shi^−^ ligands also serves as a μ-bridge between the central *Ln* ^III^ ion and an Al^III^ ion. Based on a SHAPE (*SHAPE 2.1;* Llunell *et al.*, 2013 ▸; Pinsky & Avnir, 1998 ▸; Casanova *et al.*, 2004 ▸) analysis of the *Ln* ^III^ coordination sphere, the geometry can best be described as a spherical capped square anti­prism (Table 2 ▸; Fig. 5 ▸). The continuous shape measure (CShM) values for this geometry (1.083 for **1**, 0.991 for **2**, and 0.931 for **3**) are below or near 1.0, where a value less than 1.0 typically indicates only minor distortions from the ideal shape (Cirera *et al.*, 2005 ▸).

**Figure 6:**
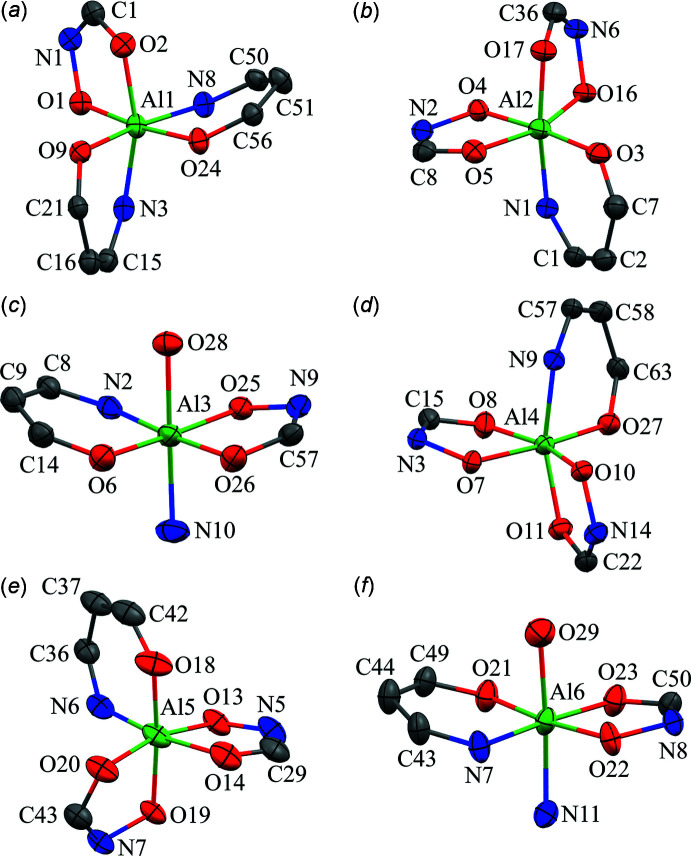
Coordination geometries for the Al^III^ ions of **1**: (*a*) Al1 – octa­hedral, Λ; (*b*) Al2 – octa­hedral, Δ; (*c*) Al3 – octa­hedral; (*d*) Al4 – octa­hedral, Δ; (*e*) Al5 – octa­hedral, Λ; (*f*) Al6 – octa­hedral. See Fig. 1 ▸ for additional display details.

The six Al^III^ ions of each metallacryptate are six-coordinate, all with an octa­hedral geometry as indicated by the CShM values (Table 3 ▸). Two of the Al^III^ ions have *trans* shi^3−^ ligands, while the remaining four Al^III^ are chiral centers (Fig. 6 ▸). For Al3 and Al6, the coordination sphere consists of two *trans*-chelate rings of two different shi^3−^ ligands. A six-membered ring comprised of an oxime nitro­gen atom and phenol oxygen atom of a shi^3−^ ligand is opposite of a five-membered chelate ring composed of oxime and carboxyl­ate oxygen atoms of a different shi^3−^ ligand. The coordination is completed by an oxygen atom of a water mol­ecule binding opposite to that of a nitro­gen atom of a pyridine mol­ecule. For Al1, Al2, A4, and Al5, the coordination sphere consists of three *cis* chelate rings in a propeller configuration with two Λ and two Δ stereoconfigurations per metallacryptand. Al1 and Al5 both have a Λ stereoconfiguration but different types of chelate rings. For Al1 the coordination is completed by one five-membered and two six-membered chelate rings from three shi^3−^ ligands, while for Al5 the coordination is completed by two five-membered rings from shi^3−^ and H_2_shi^−^ ligands and one six-membered ring from a shi^3−^ ligand. Both Al2 and Al4 have a Δ stereoconfiguration, but different types of chelate rings. For Al2 the coordination is completed by two five-membered and one six-membered chelate rings from three shi^3−^ ligands, while for Al4 the coordination is completed by two five-membered rings from shi^3−^ and H_2_shi^−^ ligands and one six-membered ring from a shi^3−^ ligand. For the Λ and Δ Al^III^ ions, the types of oxygen and nitro­gen atoms comprising the five- and six-membered chelate rings are the same as in Al3 and Al6.

For **1**–**3**, several pyridine mol­ecules are located in the lattice. Some of the pyridine mol­ecules are fully occupied and ordered (associated with N13, N18, N19, and N20, while others are disordered and/or partially occupied. The lattice pyridine mol­ecules associated with N15, N23, and N25 are correlated with the disorder of the metallacryptate (the presence or absence of the pyridine coord­in­ated to Al6). In addition, two other pyridine mol­ecules are independently disordered with a shared occupancy ratio (N14, N17 *vs* N22, N24). Lastly, the pyridine mol­ecule associated with N21 is partially occupied with 1:1 disorder around an inversion center. Complete details pertaining to the treatment of the pyridine disorder including occupancy ratios are described in the *Refinement* section.

## Supra­molecular features   

For **1**–**3**, similar numerous hydrogen bonds and weak C—H⋯O inter­actions exist within each metallacryptate, between the Hpy^+^ ions and the metallacryptate, and between the lattice pyridine mol­ecules and the metallacryptate (Tables 4 ▸–6 ▸ ▸). The protonated oxime nitro­gen atom of each of the two H_2_shi^−^ ligands forms a hydrogen bond with itself by inter­acting with the phenolate oxygen atom of the same H_2_shi^−^ ligand (N4—H4*N*⋯O12 and N5—H5*N*⋯O15). The protonated phenolate oxygen atoms of the H_2_shi^−^ ligands form hydrogen bonds to the nitro­gen atom of lattice pyridine mol­ecules (O12—H12*O*⋯N15, O15—H15⋯N14, O15—H15⋯N22). The water mol­ecules coordinated to the Al^III^ ions (O28 and O29) form hydrogen bonds to the nitro­gen atom of lattice pyridine mol­ecules (N13, N18, and N19) and to the oxime oxygen atom of a shi^3−^ ligand (O16). The lattice water mol­ecule (O30) also forms a hydrogen bond to a lattice pyridine mol­ecule (N20). The pyridinium ions form hydrogen bonds to the carboxyl­ate oxygen atom of shi^3−^ ligands (N12—H12*A*⋯O9 and N16—H16⋯O27). The shi^3−^ ligands and pyridine mol­ecules form several different types of C—H⋯O inter­actions. The carbon–hydrogen atom of a benzene ring of a shi^3−^ ligand forms an inter­action with a carboxyl­ate oxygen atom of a shi^3−^ ligand on a neighboring metallacryptate [C62—H62⋯O17^i^; symmetry code: (i) *x* + 1, *y*, *z*]. The carbon–hydrogen atom of a coordinated pyridine ligand forms an inter­action with the oxime oxygen atom of a shi^3−^ ligand (C68—H68⋯O4). The carbon–hydrogen atoms of lattice pyridine mol­ecules form inter­actions with the coordinated water mol­ecule (C74—H74⋯O28) and with phenolate oxygen atoms of shi^3−^ ligands (associated C94 and C130).

## Database survey   

A survey of the Cambridge Structural Database (CSD version 5.41, update March 2020, Groom *et al.*, 2016 ▸) reveals that there are three comparable metallacryptates. All are based on the [3.3.1] metallacryptand structure with six metal ions and seven shi^3−^ ligands forming the metallacryptand and the structures encapsulate an *Ln* ^III^ ion in the central cavity to form a metallacryptate. One structure is an individual mol­ecule as in **1**–**3** and is based on gallium(III) as the ring metal ions (DIBLOS; Lutter *et al.*, 2018*a* ▸). However, the mol­ecule contains one H_2_shi^−^ and one Hshi^2−^ ligand to help encapsulate a central Tb^III^ ion, and charge balance is maintained by three tri­ethyl­ammonium cations. Furthermore, the synthetic scheme for compounds **1**–**3** is based on the TbGa_6_ mol­ecule as the solvent choice and the ratios between the reactants are the same for both set of compounds (Lutter *et al.*, 2018*a* ▸). The other two metallacryptate structures are closely related and are also based on aluminum(III) as in **1**–**3**. However, in the other structures two Hshi^2−^ ligands complete the coordination of the central Dy^III^ ions (Travis *et al.*, 2020 ▸). In addition, the previously reported metallacryptates are connected in a two-dimensional network by a series of sodium–di­methyl­formamide bridges. The sodium ions also provide charge balance to the metallacryptate anion. The main structural difference between the previously reported Ga^III^ and Al^III^ metallacryptands and complexes **1**–**3** lies in the coordination geometries of the Ga^III^ and Al^III^ ions. In **1**–**3** all six Al^III^ ions are six-coordinate with four chiral centers (2Δ, 2Λ) and two Al^III^ ions with *trans*-chelate rings. For the previously reported metallacryptates, four of the Ga^III^ or Al^III^ ions are also chiral centers (2Δ, 2Λ); however, the other two Ga^III^ or Al^III^ ions are only five-coordinate with either a square-pyramidal or trigonal–bipyramidal geometry.

## Synthesis and crystallization   

**Synthetic materials**

Salicyl­hydroxamic acid (H_3_shi, 99%), gadolinium(III) nitrate hexa­hydrate (99.9%), and dysprosium(III) nitrate penta­hydrate (99.9%) were purchased from Alfa Aesar. Ytterbium(III) nitrate penta­hydrate (99.9%) was purchased from Strem Chemicals. Tri­ethyl­amine (99.5%) was purchased from Sigma–Aldrich. Aluminum nitrate nona­hydrate (Certified ACS grade) and pyridine (Certified ACS grade) were purchased from Fisher Scientific. Methanol (Certified ACS grade) were purchased from Pharmco–Aaper. All reagents were used as received and without further purification.

**General preparation of [3.3.1]** ***Ln*** **Al_6_ metallacryptate compounds.** The synthetic procedure for the [3.3.1]*Ln*Al_6_ metallacryptates is based on a modification of the procedure for the previously reported [3.3.1]*Ln*Ga_6_ metallacryptate compounds (Lutter *et al.*, 2018*a* ▸). The lanthanide(III) nitrate hydrate salt [0.167 mmol; 75.4 mg of Gd(NO_3_)_3_·6H_2_O for **1**, 74.2 mg of Dy(NO_3_)_3_·5H_2_O for **2**, and 75.8 mg of Yb(NO_3_)_3_·5H_2_O for **3**] and aluminum nitrate nona­hydrate (1.0 mmol, 0.3753 g for **1**, 0.3758 g for **2**, and 0.3759 g for **3**) were dissolved in 10 mL of methanol resulting in a clear and colorless solution. In a separate beaker, salicyl­hydroxamic acid (1.5 mmol, 0.2304 g for **1**, 0.2301 g for **2**, and 0.2307 g for **3**) and tri­ethyl­amine (4.5 mmol, 630 µL for **1**, **2**, and **3**) were dissolved in 10 mL of methanol, resulting in a slightly yellow solution. Then 10 mL of pyridine were added to the H_3_shi/tri­ethyl­amine solution and no color change was observed. The *Ln*/Al solution was then added to the H_3_shi/tri­ethyl­amine/pyridine solution, resulting initially in an opaque, white mixture. After stirring for approximately three hours, the solution turned clear and yellow. The solution was then gravity filtered. No precipitate was recovered and the filtrate had a pale-yellow color. Slow evaporation of the filtrate at room temperature afforded X-ray-quality, slightly pink, block-shaped crystals after ten weeks for **1**, five weeks for **2**, and eleven weeks for **3**.

**[Hpy]_2_[GdAl_6_(H_2_shi)_2_(shi)_7_(py)_1.855_(H_2_O)_2_]·7.396py·H_2_O, 1.** The percentage yield was 42% (184.6 mg, 7.046 × 10^−2^ mmol) based on gadolinium(III) nitrate hexa­hydrate.

**[Hpy]_2_[DyAl_6_(H_2_shi)_2_(shi)_7_(py)_1.891_(H_2_O)_2_]·7.429py·H_2_O, 2.** The percentage yield was 15% (65.4 mg, 2.49 × 10^−2^ mmol) based on dysprosium(III) nitrate penta­hydrate.

**[Hpy]_2_[YbAl_6_(H_2_shi)_2_(shi)_7_(py)_1.818_(H_2_O)_2_]·7.386py·H_2_O, 3.** The percentage yield was 21% (91.3 mg, 3.47 × 10^−2^ mmol) based on ytterbium(III) nitrate penta­hydrate.

## Refinement   

The structures of **1** and **3** were solved by isomorphous replacement from the analogue **2**.

For **1**–**3**, a section of the main mol­ecule is disordered induced by presence or absence of a pyridine ligand coordinated to Al6. In the absence of the pyridine moiety, an H_2_shi^−^ ligand (associated with N4) moves into the space otherwise occupied by the pyridine, and the phenol oxygen atom coord­inates to the aluminum (Al6). The movement of the H_2_shi^−^ ligand induces movement for the *Ln* ^III^ ion, for Al4, which also binds the same H_2_shi^−^ ligand, and for one of the shi^3−^ ligands (associated with N9) coordinated to Al4. For **3**, atoms C24*B* and C27*B* of the H_2_shi^−^ were constrained to have identical ADPs. The substantial movement of the H_2_shi^−^ ligand induces a shift of the solvate pyridine (associated with N15) that is hydrogen bonded to the phenol oxygen of the H_2_shi^−^. The two *Ln* ^III^ ions were constrained to have identical ADPs. Equivalent sections of the two disordered moieties were restrained to have similar geometries. Another solvate pyridine mol­ecule was included in the disorder and refined as threefold disordered (associated with N15, N23, and N25). The major disorder component solvate pyridine ring was refined as additionally disordered (associated with N15 and N23). The nitro­gen atoms of these two moieties were constrained to share positions and ADPs. The minor disorder component solvate pyridine ring (associated with N25) was constrained to resemble an ideal hexa­gon with C—C distances of 1.39 Å. The disordered pyridine rings were restrained to have similar geometries as another, not disordered pyridine ring. *U^ij^* components of ADPs for disordered atoms closer to each other than 2.0 Å were restrained to be similar. For **1**, subject to these conditions, the occupancy ratio refined to 0.8550 (13):0.1450 (13). The occupancy rates for the additionally split pyridine ring (associated with N15 and N23) are 0.531 (3) and 0.324 (3). For **2**, the occupancy ratio refined to 0.8909 (13):0.1091 (13). The occupancy rates for the additionally split pyridine ring (associated with N15 and N23) are 0.539 (3) and 0.352 (3). For **3**, the occupancy ratio refined to 0.8181 (14):0.1819 (14). The occupancy rates for the additionally split pyridine ring (associated with N15 and N23) are 0.391 (3) and 0.324 (3).

Two other solvate pyridine rings are independently disordered with a shared occupancy ratio (N14, N17 *vs* N22, N24). The disordered moieties were restrained to have similar geometries as another, not disordered pyridine ring. *U^ij^* components of ADPs for disordered atoms closer to each other than 2.0 Å were restrained to be similar. For **1**, subject to these conditions, the occupancy ratio for the pyridine mol­ecules refined to 0.613 (9) (N14 & N17):0.387 (9) (N22 & N24). For **2**, the occupancy ratio for the pyridine mol­ecules refined to 0.509 (8) (N14 & N17):0.491 (8) (N22 & N24). For **3**, the occupancy ratio for the pyridine mol­ecules refined to 0.473 (7) (N14 & N17):0.527 (7) (N22 & N24).

Another solvate pyridine (associated with N21) is 1:1 disordered around an inversion center. The disordered moieties were restrained to have similar geometries as another, not disordered pyridine ring. *U^ij^* components of ADPs for disordered atoms closer to each other than 2.0 Å were restrained to be similar. For **1**, subject to these conditions, the occupancy rate refined to 2 × 0.396 (4). For **2**, the occupancy rate refined to 2 × 0.429 (4). For **3**, the occupancy rate refined to 2 × 0.386 (5).

Water hydrogen-atom positions and some amine hydrogen-atom positions were refined and O—H and selected N—H distances were restrained to 0.84 (2) and 0.88 (2) Å, respectively. Some water, amine and phenol hydrogen-atom positions were further restrained based on hydrogen-bonding considerations (phenol hydrogen atoms were placed in calculated positions, but were allowed to rotate around the C—O axis). All other hydrogen atoms were placed in calculated positions and refined as riding on their carrier atoms with C—H distances of 0.95 Å for *sp* ^2^ carbon atoms and 0.98 Å for methyl carbon atoms. The *U*_iso_ values for hydrogen atoms were set to a multiple of the value of the carrying carbon atom (1.2 times for *sp* ^2^-hybridized carbon atoms or 1.5 times for methyl carbon atoms). Additional crystal data, data collection, and structure refinement details are summarized in Table 7 ▸.

## Supplementary Material

Crystal structure: contains datablock(s) 1, 2, 3. DOI: 10.1107/S2056989020010725/mw2167sup1.cif

Structure factors: contains datablock(s) 1. DOI: 10.1107/S2056989020010725/mw21671sup2.hkl

Structure factors: contains datablock(s) 2. DOI: 10.1107/S2056989020010725/mw21672sup3.hkl

Structure factors: contains datablock(s) 3. DOI: 10.1107/S2056989020010725/mw21673sup4.hkl

CCDC reference: 2021333

Additional supporting information:  crystallographic information; 3D view; checkCIF report

## Figures and Tables

**Figure 2:**
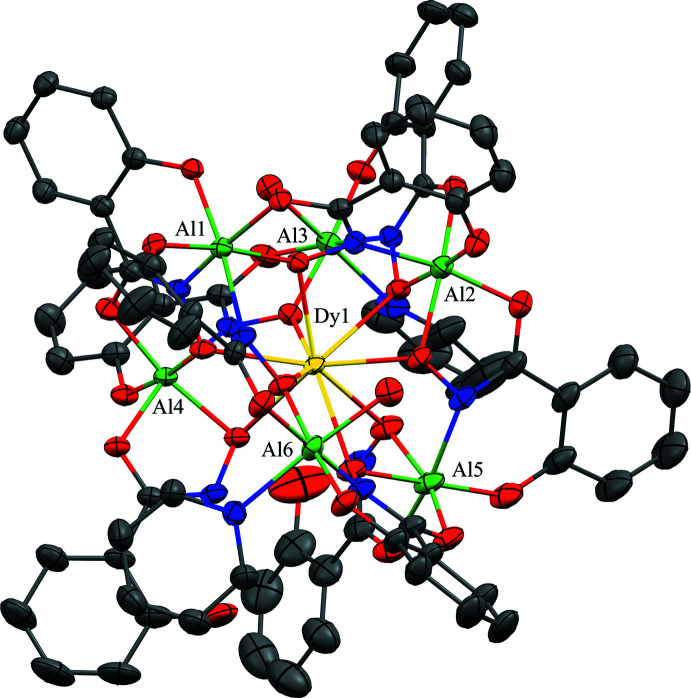
The single-crystal X-ray structure of [Hpy]_2_[DyAl_6_(H_2_shi)_2_(shi)_7_(py)_1.855_(H_2_O)_2_]·7.429py·H_2_O, **2**, with displacement ellipsoids at the 50% probability level. See Fig. 1 ▸ for additional display details.

**Figure 3:**
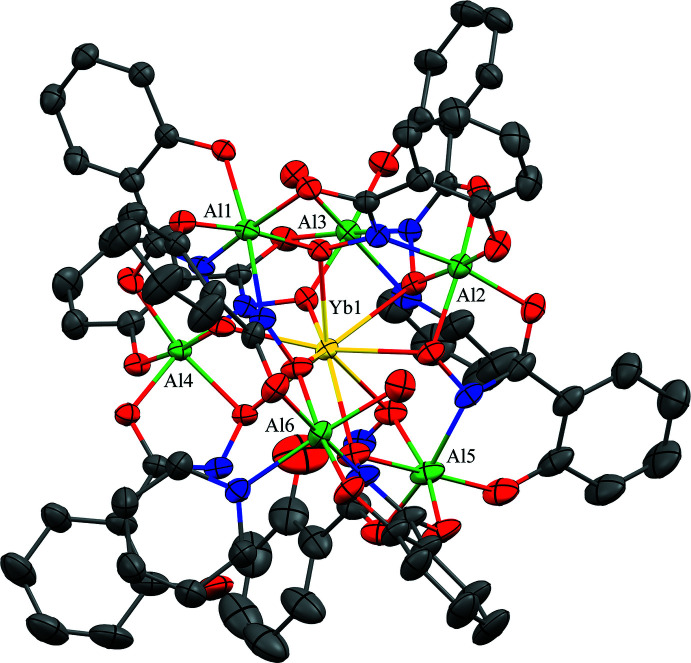
The single-crystal X-ray structure of [Hpy]_2_[YbAl_6_(H_2_shi)_2_(shi)_7_(py)_1.855_(H_2_O)_2_]·7.386py·H_2_O, **3**, with displacement ellipsoids at the 50% probability level. See Fig. 1 ▸ for additional display details.

**Table 1:** Average bond length (Å) and bond-valence-sum (BVS) values (v.u.) used to support assigned oxidation states of the lanthanide and aluminium ions of **1**–**3**

	Avg. Bond length	BVS value	Assigned oxidation state
**1**	2.451	2.96	3+
Gd1	1.927	3.07	3+
Al1	1.903	3.03	3+
Al2	1.903	3.03	3+
Al3	1.927	3.12	3+
Al4	1.904	3.03	3+
Al5	1.909	2.99	3+
Al6	1.925	3.13	3+
			
**2**			
Dy1	2.429	2.93	3+
Al1	1.928	3.06	3+
Al2	1.905	3.02	3+
Al3	1.928	3.13	3+
Al4	1.906	3.01	3+
Al5	1.910	2.98	3+
Al6	1.929	3.10	3+
			
**3**			
Yb1	2.401	3.01	3+
Al1	1.925	3.09	3+
Al2	1.903	3.04	3+
Al3	1.927	3.14	3+
Al4	1.903	3.04	3+
Al5	1.908	3.00	3+
Al6	1.923	3.16	3+

**Table 2:** Continuous Shapes Measures (CShM) values for the geometry about the nine-coordinate central *Ln* ^III^ ions in **1**–**3**

Shape	**1**	**2**	**3**
	Gd^III^	Dy^III^	Yb^III^
Enneagon (*D_9h_*)	36.299	36.527	36.772
Octa­gonal pyramid (*C_8v_*)	21.736	21.994	22.187
Heptagonal bipyramid (*D_7h_*)	18.428	18.337	18.184
Johnson triangular cupola (J3; *C_3v_*)	14.752	14.745	14.922
Capped cube (J8; *C_4v_*)	10.973	10.868	10.760
Spherical-relaxed capped cube (*C_4v_*)	9.628	9.517	9.336
Capped square anti­prism (J10; *C_4v_*)	2.167	2.069	1.995
Spherical capped square anti­prism (*C_4v_*)	1.083	0.991	0.931
Tricapped trigonal prism (J51; *D_3h_*)	3.615	3.502	3.477
Spherical tricapped trigonal prism (*D_3h_*)	1.630	1.495	1.416
Tridiminished icosa­hedron (J63; *C_3v_*)	9.897	10.055	10.392
Hula-hoop (*C_2v_*)	11.188	11.261	11.319
Muffin (*C_s_*)	1.512	1.410	1.323

**Table 3:** Continuous Shapes Measures (CShM) values for the geometry about the six-coordinate ring Al^III^ ions in **1**–**3**

Shape	Hexagon (*D_6h_*)	Penta­gonal pyramid (*C_5v_*)	Octa­hedron (*O_h_*)	Trigonal prism (*D_3h_*)	Johnson penta­gonal pyramid (J2; *C_5v_*)
**1**					
Al1	32.418	23.116	1.169	11.045	26.739
Al2	32.325	24.090	1.051	11.660	28.072
Al3	32.145	28.115	0.359	15.996	31.334
Al4	32.026	21.009	1.849	10.377	25.143
Al5	33.155	24.678	1.010	11.355	29.094
Al6	31.208	26.444	0.545	14.205	29.948
					
**2**					
Al1	32.411	22.992	1.193	10.964	26.633
Al2	32.195	24.025	1.062	11.646	28.028
Al3	32.171	28.051	0.373	15.798	31.301
Al4	31.906	20.957	1.876	10.211	25.080
Al5	33.259	24.897	0.991	11.361	29.274
Al6	31.373	26.373	0.523	14.176	29.954
					
**3**					
Al1	32.249	22.745	1.242	10.764	26.382
Al2	32.079	23.869	1.102	11.552	27.871
Al3	32.394	28.034	0.402	15.728	31.290
Al4	31.648	20.968	1.909	9.946	25.072
Al5	33.130	24.935	1.018	11.346	29.066
Al6	31.218	25.913	0.625	13.947	29.445

**Table 4:** Hydrogen-bond geometry (Å, °) for **1** [Chem scheme1]

*D*—H⋯*A*	*D*—H	H⋯*A*	*D*⋯*A*	*D*—H⋯*A*
N4—H4*N*⋯O12	0.88	1.94	2.590 (4)	130
O12—H12*O*⋯N15	0.84	1.82	2.615 (5)	158
C62—H62⋯O17^i^	0.95	2.54	3.354 (5)	143
O15—H15⋯N14	0.84	1.90	2.693 (14)	157
O15—H15⋯N22	0.84	1.69	2.51 (2)	163
O28—H28*A*⋯N13	0.86 (2)	1.78 (2)	2.630 (4)	175 (5)
O29—H29*A*⋯O16	0.89 (2)	1.84 (2)	2.723 (3)	172 (5)
O29—H29*B*⋯N18	0.90 (2)	1.88 (3)	2.701 (5)	150 (5)
O30—H30*A*⋯N19	0.85 (2)	2.11 (2)	2.830 (8)	142 (4)
O30—H30*B*⋯N20	0.87 (2)	2.22 (2)	3.033 (15)	156 (7)
N5—H5*N*⋯O15	0.88	1.95	2.617 (5)	131
C68—H68⋯O4	0.95	2.30	3.093 (5)	141
N12—H12*A*⋯O9	0.88	1.79	2.624 (4)	157
C74—H74⋯O28	0.95	2.46	3.379 (5)	164
C130—H130⋯O18^ii^	0.95	2.47	3.35 (2)	153
N16—H16⋯O27	0.88	1.76	2.632 (4)	168
C94—H94⋯O3^i^	0.95	2.29	3.131 (5)	147

Symmetry codes: (i) 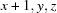; (ii) 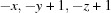.

**Table 5:** Hydrogen-bond geometry (Å, °) for **2** [Chem scheme1]

*D*—H⋯*A*	*D*—H	H⋯*A*	*D*⋯*A*	*D*—H⋯*A*
N4—H4*N*⋯O12	0.88	1.94	2.593 (4)	129
O12—H12*O*⋯N15	0.84	1.82	2.609 (5)	156
C62—H62⋯O17^i^	0.95	2.52	3.334 (4)	143
O15—H15⋯N14	0.84	1.89	2.713 (16)	165
O15—H15⋯N22	0.84	1.71	2.529 (17)	166
O28—H28*A*⋯N13	0.84 (2)	1.80 (2)	2.629 (3)	174 (4)
O29—H29*A*⋯O16	0.89 (2)	1.85 (2)	2.712 (3)	163 (4)
O29—H29*B*⋯N18	0.91 (2)	1.89 (3)	2.703 (4)	148 (4)
O30—H30*A*⋯N19	0.86 (2)	2.10 (2)	2.822 (7)	141 (4)
O30—H30*B*⋯N20	0.87 (2)	2.17 (2)	3.029 (12)	167 (10)
N5—H5*N*⋯O15	0.88	1.96	2.624 (4)	131
C68—H68⋯O4	0.95	2.29	3.073 (4)	140
N12—H12*A*⋯O9	0.88	1.79	2.625 (3)	157
C74—H74⋯O28	0.95	2.47	3.396 (4)	164
C130—H130⋯O18^ii^	0.95	2.52	3.36 (2)	149
N16—H16⋯O27	0.88	1.77	2.634 (4)	168
C94—H94⋯O3^i^	0.95	2.28	3.131 (4)	149

Symmetry codes: (i) 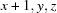; (ii) 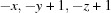.

**Table 6:** Hydrogen-bond geometry (Å, °) for **3** [Chem scheme1]

*D*—H⋯*A*	*D*—H	H⋯*A*	*D*⋯*A*	*D*—H⋯*A*
N4—H4*N*⋯O12	0.88	1.95	2.593 (4)	129
O12—H12*O*⋯N15	0.84	1.79	2.578 (6)	156
C62—H62⋯O17^i^	0.95	2.52	3.331 (5)	144
O15—H15⋯N14	0.84	1.95	2.762 (14)	162
O15—H15⋯N22	0.84	1.70	2.526 (16)	168
O28—H28*A*⋯N13	0.84 (2)	1.80 (2)	2.638 (4)	177 (4)
O29—H29*A*⋯O16	0.91 (2)	1.82 (2)	2.702 (3)	163 (5)
O29—H29*B*⋯N18	0.94 (2)	1.88 (3)	2.698 (5)	144 (4)
O30—H30*A*⋯N19	0.87 (2)	2.12 (2)	2.828 (8)	138 (4)
O30—H30*B*⋯N20	0.87 (2)	2.17 (2)	3.020 (12)	167 (10)
N5—H5*N*⋯O15	0.88	1.98	2.635 (4)	130
C68—H68⋯O4	0.95	2.30	3.078 (4)	139
N12—H12*A*⋯O9	0.88	1.78	2.612 (4)	157
C74—H74⋯O28	0.95	2.48	3.407 (5)	164
C130—H130⋯O18^ii^	0.95	2.49	3.306 (19)	144
N16—H16⋯O27	0.88	1.77	2.637 (4)	170
C94—H94⋯O3^i^	0.95	2.28	3.150 (5)	152

Symmetry codes: (i) 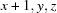; (ii) 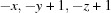.

**Table 7:** Experimental details

	**1**	**2**	**3**
Crystal data			
Chemical formula	(C_5_H_6_N)_2_[GdAl_6_(C_7_H_6_NO_3_)_2_(C_7_H_4_NO_3_)_7_(C_5_H_5_N)_1.855_(H_2_O)_2_]·7.396C_5_H_5_N·H_2_O	(C_5_H_6_N)_2_[DyAl_6_(C_7_H_6_NO_3_)_2_(C_7_H_4_NO_3_)_7_(C_5_H_5_N)_1.855_(H_2_O)_2_]·7.429C_5_H_5_N·H_2_O	(C_5_H_6_N)_2_[YbAl_6_(C_7_H_6_NO_3_)_2_(C_7_H_4_NO_3_)_7_(C_5_H_5_N)_1.855_(H_2_O)_2_]·7.386C_5_H_5_N·H_2_O
*M*_r_	2620.19	2630.90	2632.26
Crystal system, space group	Triclinic, *P* 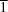	Triclinic, *P* 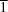	Triclinic, *P* 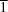
Temperature (K)	150	150	150
*a*, *b*, *c* (Å)	13.7267 (10), 16.0458 (11), 28.2591 (18)	13.7232 (6), 16.0871 (7), 28.2946 (13)	13.7254 (6), 16.0597 (8), 28.2523 (12)
α, β, γ (°)	80.675 (2), 84.459 (2), 71.757 (2)	80.632 (2), 83.989 (2), 71.660 (2)	80.5334 (16), 83.7992 (17), 72.0307 (17)
*V* (Å^3^)	5826.3 (7)	5840.6 (5)	5832.2 (5)
*Z*	2	2	2
Radiation type	Mo *K*α	Mo *K*α	Mo *K*α
μ (mm^−1^)	0.70	0.77	0.94
Crystal size (mm)	0.26 × 0.21 × 0.13	0.21 × 0.18 × 0.10	0.21 × 0.20 × 0.16

Data collection			
Diffractometer	Bruker AXS D8 Quest CMOS	Bruker AXS D8 Quest CMOS	Bruker AXS D8 Quest CMOS
Absorption correction	Multi-scan (*SADABS*; Krause *et al.*, 2015 ▸)	Multi-scan (*SADABS*; Krause *et al.*, 2015 ▸)	Multi-scan (*SADABS*; Krause *et al.*, 2015 ▸)
*T*_min_, *T*_max_	0.684, 0.746	0.225, 0.269	0.226, 0.266
No. of measured, independent and observed [*I* > 2σ(*I*)] reflections	284595, 35792, 28478	209417, 35615, 31175	103008, 33129, 26948
*R*_int_	0.056	0.045	0.043
(sin θ/λ)_max_ (Å^−1^)	0.717	0.714	0.716

Refinement			
*R*[*F* ^2^ > 2σ(*F* ^2^)], *wR*(*F* ^2^), *S*	0.054, 0.146, 1.09	0.048, 0.116, 1.14	0.048, 0.126, 1.04
No. of reflections	35792	35615	33129
No. of parameters	2119	2107	2113
No. of restraints	2173	2161	2126
H-atom treatment	H atoms treated by a mixture of independent and constrained refinement	H atoms treated by a mixture of independent and constrained refinement	H atoms treated by a mixture of independent and constrained refinement
Δρ_max_, Δρ_min_ (e Å^−3^)	1.44, −0.98	1.32, −1.09	1.27, −1.15

Computer programs: *APEX3* and *SAINT* (Bruker, 2018 ▸), *SHELXS97* (Sheldrick, 2008 ▸), *SHELXL2018/3* (Sheldrick, 2015 ▸), *shelXle* (Hübschle *et al.*, 2011 ▸), *Mercury* (Macrae *et al.*, 2020 ▸) and *publCIF* (Westrip, 2010 ▸).

## supplementary crystallographic information

### Bis(pyridinium) diaquadipyridinehexakis[µ3-salicylhydroximato(3-)]bis[µ2-salicylhydroximato(1-)]hexaaluminiumgadolinium–pyridine–water (1/7.396/1) (1) Crystal data

**Table:**

(C_5_H_6_N)_2_[GdAl_6_(C_7_H_6_NO_3_)_2_(C_7_H_4_NO_3_)_7_(C_5_H_5_N)_1.855_(H_2_O)_2_]·7.396C_5_H_5_N·H_2_O	*Z* = 2
*M**_r_* = 2620.19	*F*(000) = 2687.1
Triclinic, *P*1	*D*_x_ = 1.493 Mg m^−^^3^
*a* = 13.7267 (10) Å	Mo *K*α radiation, λ = 0.71073 Å
*b* = 16.0458 (11) Å	Cell parameters from 9126 reflections
*c* = 28.2591 (18) Å	θ = 2.5–29.7°
α = 80.675 (2)°	µ = 0.70 mm^−^^1^
β = 84.459 (2)°	*T* = 150 K
γ = 71.757 (2)°	Block, colourless
*V* = 5826.3 (7) Å^3^	0.26 × 0.21 × 0.13 mm

### Bis(pyridinium) diaquadipyridinehexakis[µ3-salicylhydroximato(3-)]bis[µ2-salicylhydroximato(1-)]hexaaluminiumgadolinium–pyridine–water (1/7.396/1) (1) Data collection

**Table:**

Bruker AXS D8 Quest CMOS diffractometer	35792 independent reflections
Radiation source: fine focus sealed tube X-ray source	28478 reflections with *I* > 2σ(*I*)
Triumph curved graphite crystal monochromator	*R*_int_ = 0.056
Detector resolution: 10.4167 pixels mm^-1^	θ_max_ = 30.6°, θ_min_ = 2.2°
ω and phi scans	*h* = −19→19
Absorption correction: multi-scan (SADABS; Krause *et al.*, 2015)	*k* = −22→22
*T*_min_ = 0.684, *T*_max_ = 0.746	*l* = −40→39
284595 measured reflections	

### Bis(pyridinium) diaquadipyridinehexakis[µ3-salicylhydroximato(3-)]bis[µ2-salicylhydroximato(1-)]hexaaluminiumgadolinium–pyridine–water (1/7.396/1) (1) Refinement

**Table:**

Refinement on *F*^2^	Primary atom site location: isomorphous structure methods
Least-squares matrix: full	Secondary atom site location: difference Fourier map
*R*[*F*^2^ > 2σ(*F*^2^)] = 0.054	Hydrogen site location: mixed
*wR*(*F*^2^) = 0.146	H atoms treated by a mixture of independent and constrained refinement
*S* = 1.09	*w* = 1/[σ^2^(*F*_o_^2^) + (0.0552*P*)^2^ + 12.4814*P*] where *P* = (*F*_o_^2^ + 2*F*_c_^2^)/3
35792 reflections	(Δ/σ)_max_ = 0.002
2119 parameters	Δρ_max_ = 1.44 e Å^−^^3^
2173 restraints	Δρ_min_ = −0.98 e Å^−^^3^

### Bis(pyridinium) diaquadipyridinehexakis[µ3-salicylhydroximato(3-)]bis[µ2-salicylhydroximato(1-)]hexaaluminiumgadolinium–pyridine–water (1/7.396/1) (1) Special details

**Table:**

Geometry. All esds (except the esd in the dihedral angle between two l.s. planes) are estimated using the full covariance matrix. The cell esds are taken into account individually in the estimation of esds in distances, angles and torsion angles; correlations between esds in cell parameters are only used when they are defined by crystal symmetry. An approximate (isotropic) treatment of cell esds is used for estimating esds involving l.s. planes.
Refinement. The structure was solved by isomorphous replacement from its Dy analogue, RR1_65. A section of the main molecule is disordered induced by presence or absence of an aluminum coordinated pyridine ligand. In the absence of the pyridine moiety a salicylate ligand swings into the space otherwise occupied by the pyridine, and the phenol oxygen atom coordinates to the aluminum (Al6). The movement of the salicylate ligand induces shifts to the Gd ion, to the aluminum ion the other two oxygen atoms are coordinated to (Al4) and one of the other salicylate ligands coordinated to Al4. The substantial movement of the first salicylate ligand induces a shift of the solvate pyridine its phenol oxygen is hydrogen bonded to. Another solvate pyridine molecule (adjacent to the second disordered salicylate ligand) was included in the disorder. The two Gd ions were constrained to have identical ADPs. Equivalent sections of the two disordered moieties were restrained to have similar geometries. The minor solvate pyridine ring was constrained to resemble an ideal hexagon with C-C distances of 1.39 Angstrom. The major moiety solvate pyridine ring was refined as additionally disordered. The nitrogen atoms of these two moieties were constrained to share position and ADP. Its two moieties and the other disordered pyridyl ring were restrained to have similar geometries as another not disordered pyridine ring. Uij components of ADPs for disordered atoms closer to each other than 2.0 Angstrom were restrained to be similar. Subject to these conditions the occupancy ratio refined to 0.8550 (13) to 0.1450 (13). The occupancy rates for the additionally split ring are 0.531 (3) and 0.324 (3).Two other solvate pyridyl rings are independently disordered with a shared occupancy ratio (N14, N17 vs N22, N24). The disordered moieties were restrained to have similar geometries as another not disordered pyridine ring. Uij components of ADPs for disordered atoms closer to each other than 2.0 Angstrom were restrained to be similar. Subject to these conditions the occupancy ratio for the one disordered in a general position refined to 0.613 (9) to 0.387 (9). Another solvate pyridyl ring is 1:1 disordered around an inversion center and partially occupied. The disordered moieties were restrained to have similar geometries as another not disordered pyridine ring. Uij components of ADPs for disordered atoms closer to each other than 2.0 Angstrom were restrained to be similar. Subject to these conditions the occupancy rate refined to two times 0.396 (4).Water H atom positions and some amine H atom positions were refined and O-H and selected N-H distances were restrained to 0.84 (2) and 0.88 (2) Angstrom, respectively. Some water, amine and phenol H atom positions were further restrained based on hydrogen bonding considerations (phenol H atoms were placed in calculated positions, but were allowed to rotate around the C-O axis).

### Bis(pyridinium) diaquadipyridinehexakis[µ3-salicylhydroximato(3-)]bis[µ2-salicylhydroximato(1-)]hexaaluminiumgadolinium–pyridine–water (1/7.396/1) (1) Fractional atomic coordinates and isotropic or equivalent isotropic displacement parameters (Å^2^)

**Table:**

	*x*	*y*	*z*	*U*_iso_*/*U*_eq_	Occ. (<1)
Al1	−0.03254 (7)	0.87424 (6)	0.12743 (3)	0.02226 (17)	
Al2	−0.31499 (7)	0.93466 (6)	0.23781 (3)	0.02663 (18)	
Al3	−0.11826 (7)	1.10637 (6)	0.25737 (3)	0.02660 (18)	
Al5	−0.13915 (9)	0.75747 (7)	0.36787 (3)	0.0327 (2)	
Al6	−0.04737 (7)	0.61891 (6)	0.23589 (3)	0.02525 (18)	
O1	−0.11609 (16)	0.91539 (13)	0.18193 (7)	0.0238 (4)	
O2	−0.16100 (17)	0.88827 (15)	0.10415 (7)	0.0272 (4)	
O3	−0.40256 (19)	0.89815 (17)	0.20781 (8)	0.0343 (5)	
O4	−0.21721 (17)	0.97252 (13)	0.26414 (7)	0.0255 (4)	
O5	−0.37369 (17)	1.05765 (15)	0.21974 (8)	0.0291 (4)	
O7	0.08928 (17)	0.84930 (14)	0.20983 (7)	0.0267 (4)	
O8	0.21067 (18)	0.93296 (15)	0.17940 (8)	0.0308 (5)	
O9	−0.03315 (16)	0.98454 (13)	0.09082 (7)	0.0243 (4)	
Gd1	−0.05698 (3)	0.85203 (2)	0.26033 (2)	0.01944 (6)	0.8550 (13)
Al4	0.18272 (10)	0.88646 (8)	0.24141 (5)	0.0204 (3)	0.8550 (13)
O10	0.1206 (2)	0.82580 (17)	0.29520 (10)	0.0247 (5)	0.8550 (13)
N4	0.1822 (2)	0.74022 (19)	0.30500 (11)	0.0276 (6)	0.8550 (13)
H4N	0.163874	0.701853	0.327113	0.033*	0.8550 (13)
O11	0.2875 (2)	0.77628 (17)	0.24650 (9)	0.0252 (5)	0.8550 (13)
C22	0.2697 (3)	0.7179 (2)	0.27998 (12)	0.0256 (6)	0.8550 (13)
C23	0.3489 (3)	0.6320 (2)	0.29183 (14)	0.0330 (8)	0.8550 (13)
C24	0.4402 (3)	0.6159 (3)	0.26443 (18)	0.0396 (9)	0.8550 (13)
H24	0.448377	0.658326	0.237963	0.048*	0.8550 (13)
C25	0.5201 (4)	0.5385 (3)	0.2751 (2)	0.0524 (12)	0.8550 (13)
H25	0.582948	0.528058	0.256429	0.063*	0.8550 (13)
C26	0.5069 (5)	0.4767 (3)	0.3135 (2)	0.0610 (14)	0.8550 (13)
H26	0.561263	0.423548	0.320898	0.073*	0.8550 (13)
C27	0.4171 (5)	0.4908 (3)	0.3407 (2)	0.0587 (13)	0.8550 (13)
H27	0.409927	0.447460	0.366876	0.070*	0.8550 (13)
C28	0.3349 (4)	0.5690 (3)	0.33051 (17)	0.0444 (10)	0.8550 (13)
O12	0.2453 (3)	0.5859 (2)	0.35611 (13)	0.0575 (10)	0.8550 (13)
H12O	0.242645	0.539950	0.374585	0.086*	0.8550 (13)
O25	−0.0279 (3)	0.9909 (2)	0.26351 (16)	0.0240 (8)	0.8550 (13)
N9	0.0750 (3)	0.9934 (2)	0.25845 (13)	0.0237 (7)	0.8550 (13)
O26	−0.0017 (3)	1.1390 (2)	0.26320 (16)	0.0311 (9)	0.8550 (13)
C57	0.0813 (3)	1.0721 (3)	0.25978 (15)	0.0259 (8)	0.8550 (13)
C58	0.1824 (3)	1.0861 (3)	0.25764 (14)	0.0282 (7)	0.8550 (13)
C59	0.1867 (3)	1.1730 (3)	0.25357 (17)	0.0374 (9)	0.8550 (13)
H59	0.124982	1.221068	0.251227	0.045*	0.8550 (13)
C60	0.2801 (4)	1.1895 (3)	0.25294 (19)	0.0447 (10)	0.8550 (13)
H60	0.282401	1.248519	0.250848	0.054*	0.8550 (13)
C61	0.3702 (4)	1.1191 (3)	0.25537 (19)	0.0426 (9)	0.8550 (13)
H61	0.434511	1.130246	0.254333	0.051*	0.8550 (13)
C62	0.3670 (3)	1.0330 (3)	0.25931 (15)	0.0327 (8)	0.8550 (13)
H62	0.429248	0.985413	0.260909	0.039*	0.8550 (13)
C63	0.2735 (3)	1.0151 (3)	0.26095 (13)	0.0269 (7)	0.8550 (13)
O27	0.2742 (2)	0.9302 (2)	0.26605 (10)	0.0256 (5)	0.8550 (13)
N11	0.1094 (3)	0.5515 (2)	0.24676 (13)	0.0286 (6)	0.8550 (13)
C69	0.1359 (3)	0.4813 (3)	0.28040 (16)	0.0369 (8)	0.8550 (13)
H69	0.084267	0.469275	0.302930	0.044*	0.8550 (13)
C70	0.2347 (4)	0.4252 (3)	0.28400 (19)	0.0460 (10)	0.8550 (13)
H70	0.250606	0.376167	0.308827	0.055*	0.8550 (13)
C71	0.3094 (4)	0.4406 (3)	0.25175 (19)	0.0464 (10)	0.8550 (13)
H71	0.377781	0.401765	0.253123	0.056*	0.8550 (13)
C72	0.2838 (3)	0.5135 (3)	0.21711 (18)	0.0480 (10)	0.8550 (13)
H72	0.334273	0.526246	0.194101	0.058*	0.8550 (13)
C73	0.1835 (3)	0.5680 (3)	0.21634 (16)	0.0380 (8)	0.8550 (13)
H73	0.166740	0.619538	0.193079	0.046*	0.8550 (13)
Gd1B	−0.08664 (15)	0.85006 (12)	0.26260 (6)	0.01944 (6)	0.1450 (13)
Al4B	0.1660 (6)	0.8615 (5)	0.2476 (3)	0.0267 (15)	0.1450 (13)
O10B	0.0972 (10)	0.8007 (9)	0.2973 (5)	0.029 (3)	0.1450 (13)
N4B	0.1365 (12)	0.7116 (9)	0.2916 (6)	0.029 (2)	0.1450 (13)
H4B	0.104 (15)	0.671 (11)	0.297 (5)	0.035*	0.1450 (13)
O11B	0.2609 (11)	0.7462 (8)	0.2466 (6)	0.028 (2)	0.1450 (13)
C22B	0.2139 (13)	0.6878 (9)	0.2606 (7)	0.030 (2)	0.1450 (13)
C23B	0.2495 (13)	0.5960 (11)	0.2493 (9)	0.039 (3)	0.1450 (13)
C24B	0.3519 (14)	0.5654 (14)	0.2336 (10)	0.044 (3)	0.1450 (13)
H24B	0.396126	0.600372	0.234198	0.053*	0.1450 (13)
C25B	0.3897 (16)	0.4839 (14)	0.2172 (11)	0.048 (3)	0.1450 (13)
H25B	0.458763	0.463863	0.205005	0.057*	0.1450 (13)
C26B	0.3252 (15)	0.4322 (15)	0.2187 (11)	0.045 (3)	0.1450 (13)
H26B	0.353712	0.373011	0.212026	0.055*	0.1450 (13)
C27B	0.2238 (14)	0.4624 (13)	0.2294 (9)	0.041 (2)	0.1450 (13)
H27B	0.179564	0.430078	0.223919	0.049*	0.1450 (13)
C28B	0.1838 (14)	0.5434 (13)	0.2489 (10)	0.040 (2)	0.1450 (13)
O12B	0.0869 (12)	0.5726 (11)	0.2661 (7)	0.034 (3)	0.1450 (13)
H12P	0.080233	0.543352	0.293099	0.051*	0.1450 (13)
O25B	−0.0389 (14)	0.9773 (11)	0.2710 (12)	0.025 (3)	0.1450 (13)
N9B	0.0673 (12)	0.9715 (9)	0.2658 (9)	0.022 (2)	0.1450 (13)
O26B	0.0045 (18)	1.1177 (12)	0.2708 (11)	0.026 (3)	0.1450 (13)
C57B	0.0828 (13)	1.0467 (12)	0.2668 (12)	0.027 (3)	0.1450 (13)
C58B	0.1883 (13)	1.0523 (12)	0.2671 (10)	0.029 (2)	0.1450 (13)
C59B	0.1999 (15)	1.1360 (14)	0.2638 (11)	0.036 (3)	0.1450 (13)
H59B	0.141268	1.186987	0.260908	0.043*	0.1450 (13)
C60B	0.2986 (16)	1.1459 (14)	0.2647 (11)	0.039 (2)	0.1450 (13)
H60B	0.307486	1.202210	0.264794	0.047*	0.1450 (13)
C61B	0.3811 (16)	1.0701 (15)	0.2653 (10)	0.038 (3)	0.1450 (13)
H61B	0.448451	1.075348	0.263583	0.046*	0.1450 (13)
C62B	0.3698 (14)	0.9871 (14)	0.2683 (9)	0.031 (3)	0.1450 (13)
H62B	0.429097	0.936620	0.270313	0.037*	0.1450 (13)
C63B	0.2735 (13)	0.9756 (12)	0.2685 (9)	0.028 (2)	0.1450 (13)
O27B	0.2651 (11)	0.8937 (10)	0.2738 (6)	0.028 (2)	0.1450 (13)
O13	−0.0963 (2)	0.86128 (15)	0.34925 (8)	0.0353 (5)	
O14	−0.0258 (2)	0.72662 (17)	0.40994 (9)	0.0424 (6)	
O15	0.1158 (4)	0.9201 (3)	0.40772 (15)	0.0919 (15)	
H15	0.126385	0.959898	0.420603	0.138*	
O16	−0.22929 (18)	0.82362 (14)	0.27040 (7)	0.0284 (4)	
O17	−0.39396 (19)	0.93089 (16)	0.29572 (8)	0.0324 (5)	
O18	−0.2290 (2)	0.79005 (17)	0.41746 (8)	0.0448 (7)	
O19	−0.05118 (19)	0.71694 (14)	0.31458 (7)	0.0294 (5)	
O20	−0.1509 (2)	0.64366 (16)	0.37567 (8)	0.0381 (6)	
O21	−0.07470 (19)	0.51469 (14)	0.25136 (8)	0.0314 (5)	
O22	−0.03044 (19)	0.73117 (14)	0.21565 (7)	0.0304 (5)	
O23	−0.02071 (19)	0.60680 (14)	0.17198 (8)	0.0307 (5)	
O24	0.04367 (17)	0.81776 (14)	0.07902 (7)	0.0262 (4)	
O28	−0.08824 (19)	1.10936 (15)	0.18666 (8)	0.0308 (5)	
H28A	−0.063 (3)	1.150 (2)	0.1723 (15)	0.046*	
H28B	−0.069 (3)	1.0607 (18)	0.1765 (15)	0.046*	
O29	−0.1971 (2)	0.67574 (17)	0.22813 (10)	0.0380 (5)	
H29A	−0.209 (4)	0.727 (2)	0.2391 (17)	0.057*	
H29B	−0.237 (3)	0.675 (3)	0.2043 (13)	0.057*	
O30	0.4067 (7)	0.4773 (7)	0.0089 (4)	0.185 (4)	
H30A	0.429 (5)	0.493 (8)	0.032 (4)	0.278*	
H30B	0.394 (13)	0.427 (5)	0.014 (3)	0.278*	
N1	−0.2189 (2)	0.91576 (16)	0.17976 (9)	0.0245 (5)	
N2	−0.2302 (2)	1.06264 (16)	0.25044 (9)	0.0256 (5)	
N3	0.0767 (2)	0.89080 (16)	0.16225 (8)	0.0241 (5)	
N5	−0.0181 (3)	0.8586 (2)	0.37670 (10)	0.0406 (7)	
H5N	0.006809	0.903465	0.374182	0.049*	
N6	−0.2517 (2)	0.81974 (18)	0.32141 (9)	0.0324 (6)	
N7	−0.0710 (2)	0.64255 (17)	0.30169 (9)	0.0297 (5)	
N8	−0.0161 (2)	0.74817 (16)	0.16488 (8)	0.0243 (5)	
C1	−0.2350 (2)	0.9029 (2)	0.13694 (10)	0.0262 (6)	
C2	−0.3380 (3)	0.9057 (2)	0.12538 (11)	0.0296 (6)	
C3	−0.3576 (3)	0.9099 (2)	0.07725 (12)	0.0355 (7)	
H3	−0.306134	0.915825	0.053055	0.043*	
C4	−0.4499 (3)	0.9056 (3)	0.06418 (14)	0.0434 (9)	
H4	−0.462226	0.908496	0.031364	0.052*	
C5	−0.5244 (3)	0.8971 (3)	0.09979 (15)	0.0461 (9)	
H5	−0.587595	0.892475	0.091244	0.055*	
C6	−0.5081 (3)	0.8953 (3)	0.14788 (14)	0.0411 (8)	
H6	−0.560709	0.890266	0.171611	0.049*	
C7	−0.4154 (3)	0.9006 (2)	0.16174 (12)	0.0318 (6)	
C8	−0.3147 (2)	1.1020 (2)	0.22776 (11)	0.0269 (6)	0.8550 (13)
C9	−0.3444 (7)	1.1987 (3)	0.2133 (4)	0.0293 (14)	0.8550 (13)
C10	−0.4351 (3)	1.2391 (3)	0.18888 (17)	0.0353 (12)	0.8550 (13)
H10	−0.473680	1.203386	0.181514	0.042*	0.8550 (13)
C11	−0.4696 (4)	1.3295 (3)	0.17533 (19)	0.0427 (10)	0.8550 (13)
H11	−0.530746	1.355649	0.158340	0.051*	0.8550 (13)
C12	−0.4140 (4)	1.3818 (3)	0.18680 (18)	0.0425 (10)	0.8550 (13)
H12	−0.437381	1.444229	0.177683	0.051*	0.8550 (13)
C13	−0.3256 (4)	1.3440 (3)	0.21120 (17)	0.0380 (10)	0.8550 (13)
H13	−0.289175	1.381013	0.219118	0.046*	0.8550 (13)
C14	−0.2874 (4)	1.2517 (3)	0.2248 (2)	0.0301 (12)	0.8550 (13)
O6	−0.20050 (19)	1.21963 (14)	0.24792 (9)	0.0329 (5)	0.8550 (13)
C8B	−0.3147 (2)	1.1020 (2)	0.22776 (11)	0.0269 (6)	0.1450 (13)
C9B	−0.339 (5)	1.1972 (17)	0.209 (3)	0.030 (3)	0.1450 (13)
C10B	−0.421 (2)	1.2347 (16)	0.1784 (12)	0.036 (3)	0.1450 (13)
H10B	−0.461124	1.199155	0.172773	0.043*	0.1450 (13)
C11B	−0.445 (2)	1.3213 (14)	0.1564 (10)	0.041 (3)	0.1450 (13)
H11B	−0.501727	1.345684	0.136585	0.049*	0.1450 (13)
C12B	−0.3839 (19)	1.3722 (14)	0.1639 (10)	0.043 (3)	0.1450 (13)
H12B	−0.398034	1.431496	0.148296	0.052*	0.1450 (13)
C13B	−0.3032 (19)	1.3372 (13)	0.1937 (9)	0.036 (3)	0.1450 (13)
H13B	−0.262695	1.373472	0.198218	0.043*	0.1450 (13)
C14B	−0.278 (3)	1.2498 (16)	0.2178 (16)	0.032 (3)	0.1450 (13)
O6B	−0.20050 (19)	1.21963 (14)	0.24792 (9)	0.0329 (5)	0.1450 (13)
C15	0.1427 (2)	0.93515 (19)	0.14993 (10)	0.0253 (6)	
C16	0.1364 (2)	0.9902 (2)	0.10288 (10)	0.0266 (6)	
C17	0.2158 (3)	1.0267 (2)	0.08670 (12)	0.0327 (7)	
H17	0.272637	1.014418	0.106186	0.039*	
C18	0.2134 (3)	1.0802 (2)	0.04290 (12)	0.0351 (7)	
H18	0.267831	1.104536	0.032295	0.042*	
C19	0.1300 (3)	1.0977 (2)	0.01483 (11)	0.0314 (6)	
H19	0.128171	1.133062	−0.015666	0.038*	
C20	0.0493 (2)	1.0641 (2)	0.03070 (11)	0.0274 (6)	
H20	−0.007190	1.077221	0.010911	0.033*	
C21	0.0492 (2)	1.01113 (19)	0.07536 (10)	0.0240 (5)	
C29	0.0180 (3)	0.7864 (3)	0.40685 (12)	0.0400 (8)	
C30	0.1080 (3)	0.7746 (3)	0.43510 (13)	0.0496 (10)	
C31	0.1489 (4)	0.6931 (4)	0.46198 (18)	0.0642 (13)	
H31	0.118148	0.647462	0.462442	0.077*	
C32	0.2350 (5)	0.6773 (5)	0.4885 (2)	0.0831 (19)	
H32	0.262569	0.621290	0.507192	0.100*	
C33	0.2798 (5)	0.7432 (5)	0.4874 (2)	0.0824 (19)	
H33	0.338351	0.732319	0.505570	0.099*	
C34	0.2414 (4)	0.8238 (5)	0.46076 (19)	0.0729 (16)	
H34	0.273508	0.868508	0.460162	0.087*	
C35	0.1554 (4)	0.8405 (4)	0.43443 (15)	0.0614 (13)	
C36	−0.3401 (3)	0.8789 (2)	0.33004 (11)	0.0318 (7)	
C37	−0.3830 (3)	0.8861 (2)	0.37991 (12)	0.0397 (8)	
C38	−0.4843 (4)	0.9403 (3)	0.38611 (15)	0.0507 (10)	
H38	−0.521146	0.972382	0.358743	0.061*	
C39	−0.5319 (4)	0.9480 (4)	0.43178 (17)	0.0700 (15)	
H39	−0.600899	0.984133	0.435723	0.084*	
C40	−0.4764 (5)	0.9017 (4)	0.47123 (17)	0.0798 (18)	
H40	−0.507898	0.906289	0.502540	0.096*	
C41	−0.3763 (4)	0.8491 (3)	0.46590 (15)	0.0650 (14)	
H41	−0.340443	0.818254	0.493761	0.078*	
C42	−0.3252 (3)	0.8395 (3)	0.42029 (13)	0.0434 (9)	
C43	−0.1219 (3)	0.6076 (2)	0.33659 (11)	0.0322 (7)	
C44	−0.1484 (3)	0.5269 (2)	0.33213 (12)	0.0354 (7)	
C45	−0.1970 (3)	0.4893 (3)	0.37196 (15)	0.0463 (9)	
H45	−0.211886	0.516375	0.400399	0.056*	
C46	−0.2237 (4)	0.4136 (3)	0.37072 (16)	0.0536 (11)	
H46	−0.256380	0.388542	0.398024	0.064*	
C47	−0.2021 (4)	0.3744 (3)	0.32881 (17)	0.0505 (10)	
H47	−0.221586	0.323079	0.327342	0.061*	
C48	−0.1528 (3)	0.4094 (2)	0.28948 (14)	0.0389 (8)	
H48	−0.137836	0.380897	0.261503	0.047*	
C49	−0.1240 (3)	0.4861 (2)	0.28967 (12)	0.0315 (6)	
C50	−0.0097 (2)	0.6777 (2)	0.14566 (10)	0.0253 (6)	
C51	0.0155 (3)	0.6755 (2)	0.09404 (11)	0.0297 (6)	
C52	0.0176 (4)	0.5993 (3)	0.07557 (14)	0.0504 (11)	
H52	−0.006898	0.555525	0.095300	0.060*	
C53	0.0545 (5)	0.5862 (3)	0.02911 (15)	0.0599 (13)	
H53	0.054620	0.534470	0.016835	0.072*	
C54	0.0914 (4)	0.6501 (3)	0.00081 (13)	0.0484 (10)	
H54	0.120340	0.640680	−0.030515	0.058*	
C55	0.0861 (3)	0.7270 (2)	0.01791 (11)	0.0334 (7)	
H55	0.109441	0.770795	−0.002444	0.040*	
C56	0.0471 (2)	0.7427 (2)	0.06476 (11)	0.0259 (6)	
N10	−0.1493 (3)	1.0980 (2)	0.33242 (11)	0.0441 (8)	
C64	−0.1096 (4)	1.1424 (3)	0.35821 (17)	0.0619 (13)	
H64	−0.066956	1.175876	0.342235	0.074*	
C65	−0.1295 (7)	1.1406 (4)	0.4074 (2)	0.098 (3)	
H65	−0.102546	1.173764	0.424731	0.118*	
C66	−0.1881 (8)	1.0905 (4)	0.4305 (2)	0.120 (4)	
H66	−0.200994	1.087322	0.464263	0.145*	
C67	−0.2284 (8)	1.0448 (4)	0.40507 (18)	0.117 (3)	
H67	−0.269944	1.009879	0.420546	0.140*	
C68	−0.2067 (5)	1.0509 (3)	0.35591 (15)	0.0651 (15)	
H68	−0.234804	1.019266	0.338075	0.078*	
N12	−0.2019 (3)	1.1049 (2)	0.06022 (13)	0.0509 (9)	
H12A	−0.154837	1.054596	0.069481	0.061*	
C74	−0.2317 (4)	1.1653 (4)	0.08914 (16)	0.0621 (13)	
H74	−0.204145	1.153116	0.119963	0.075*	
C75	−0.3016 (4)	1.2452 (4)	0.0757 (2)	0.0675 (14)	
H75	−0.322019	1.289051	0.096606	0.081*	
C76	−0.3422 (4)	1.2615 (3)	0.0312 (2)	0.0689 (15)	
H76	−0.390094	1.317170	0.020697	0.083*	
C77	−0.3120 (4)	1.1955 (4)	0.00222 (18)	0.0696 (15)	
H77	−0.341094	1.204661	−0.028086	0.084*	
C78	−0.2406 (4)	1.1173 (3)	0.01714 (18)	0.0563 (11)	
H78	−0.218474	1.072003	−0.002882	0.068*	
N13	−0.0104 (3)	1.2363 (2)	0.14749 (11)	0.0408 (7)	
C79	−0.0215 (4)	1.2980 (3)	0.17547 (15)	0.0470 (9)	
H79	−0.060531	1.294380	0.204958	0.056*	
C80	0.0199 (4)	1.3667 (3)	0.16445 (18)	0.0549 (11)	
H80	0.011418	1.408813	0.185753	0.066*	
C81	0.0739 (5)	1.3717 (3)	0.1215 (2)	0.0754 (16)	
H81	0.103871	1.418174	0.112267	0.091*	
C82	0.0850 (5)	1.3093 (4)	0.0916 (2)	0.0800 (18)	
H82	0.123042	1.311785	0.061721	0.096*	
C83	0.0394 (4)	1.2428 (3)	0.10590 (15)	0.0536 (11)	
H83	0.044511	1.200797	0.084917	0.064*	
N14	0.1584 (19)	1.0659 (11)	0.4234 (5)	0.085 (2)	0.613 (9)
C84	0.141 (2)	1.1032 (11)	0.4639 (6)	0.088 (2)	0.613 (9)
H84	0.140587	1.066982	0.494043	0.106*	0.613 (9)
C85	0.1242 (11)	1.1912 (9)	0.4627 (4)	0.090 (2)	0.613 (9)
H85	0.111379	1.216438	0.491727	0.108*	0.613 (9)
C86	0.1256 (12)	1.2428 (9)	0.4202 (4)	0.103 (3)	0.613 (9)
H86	0.116460	1.304237	0.419236	0.123*	0.613 (9)
C87	0.1401 (12)	1.2068 (9)	0.3785 (4)	0.103 (3)	0.613 (9)
H87	0.135822	1.242828	0.348103	0.123*	0.613 (9)
C88	0.1610 (12)	1.1167 (9)	0.3821 (5)	0.097 (3)	0.613 (9)
H88	0.178189	1.089818	0.353594	0.116*	0.613 (9)
N22	0.152 (3)	1.053 (2)	0.4289 (10)	0.094 (3)	0.387 (9)
C124	0.135 (3)	1.090 (2)	0.4693 (9)	0.092 (3)	0.387 (9)
H124	0.099485	1.065411	0.495687	0.111*	0.387 (9)
C125	0.1658 (19)	1.1602 (15)	0.4746 (6)	0.098 (3)	0.387 (9)
H125	0.159596	1.180233	0.504911	0.117*	0.387 (9)
C126	0.206 (2)	1.2008 (14)	0.4355 (6)	0.108 (3)	0.387 (9)
H126	0.230293	1.248747	0.438355	0.129*	0.387 (9)
C127	0.210 (2)	1.1728 (14)	0.3913 (6)	0.107 (3)	0.387 (9)
H127	0.224618	1.207326	0.362354	0.129*	0.387 (9)
C128	0.194 (2)	1.0925 (14)	0.3912 (8)	0.100 (3)	0.387 (9)
H128	0.212778	1.064417	0.363078	0.120*	0.387 (9)
N15	0.1912 (4)	0.4712 (3)	0.42149 (17)	0.0623 (11)	0.531 (3)
C89	0.2358 (11)	0.4290 (11)	0.4610 (5)	0.065 (3)	0.531 (3)
H89	0.304369	0.429093	0.463258	0.078*	0.531 (3)
C90	0.1963 (10)	0.3852 (16)	0.4989 (6)	0.070 (2)	0.531 (3)
H90	0.233609	0.356420	0.526625	0.083*	0.531 (3)
C91	0.0972 (10)	0.3856 (8)	0.4941 (4)	0.083 (2)	0.531 (3)
H91	0.064286	0.354841	0.518800	0.100*	0.531 (3)
C92	0.0441 (9)	0.4315 (9)	0.4524 (4)	0.088 (2)	0.531 (3)
H92	−0.022910	0.429893	0.447925	0.105*	0.531 (3)
C93	0.0931 (8)	0.4784 (9)	0.4189 (5)	0.080 (2)	0.531 (3)
H93	0.055753	0.517035	0.393166	0.096*	0.531 (3)
N23	0.1912 (4)	0.4712 (3)	0.42149 (17)	0.0623 (11)	0.324 (3)
C129	0.2319 (16)	0.4161 (18)	0.4593 (7)	0.063 (3)	0.324 (3)
H129	0.304683	0.393450	0.458964	0.076*	0.324 (3)
C130	0.1793 (14)	0.389 (3)	0.4987 (10)	0.068 (3)	0.324 (3)
H130	0.212707	0.344566	0.523506	0.081*	0.324 (3)
C131	0.0750 (14)	0.4286 (14)	0.5008 (6)	0.082 (3)	0.324 (3)
H131	0.035142	0.418729	0.529334	0.098*	0.324 (3)
C132	0.0280 (12)	0.4837 (14)	0.4605 (6)	0.083 (3)	0.324 (3)
H132	−0.044605	0.507639	0.459918	0.100*	0.324 (3)
C133	0.0905 (10)	0.5021 (14)	0.4218 (7)	0.075 (3)	0.324 (3)
H133	0.059414	0.538775	0.393980	0.090*	0.324 (3)
N25	0.090 (2)	0.4310 (18)	0.3595 (7)	0.090 (4)	0.1450 (13)
C139	0.154 (2)	0.4454 (17)	0.3903 (9)	0.078 (3)	0.1450 (13)
H139	0.200558	0.478083	0.378220	0.094*	0.1450 (13)
C140	0.151 (2)	0.4119 (19)	0.4388 (8)	0.077 (2)	0.1450 (13)
H140	0.195206	0.421686	0.459830	0.092*	0.1450 (13)
C141	0.084 (2)	0.364 (2)	0.4565 (7)	0.086 (3)	0.1450 (13)
H141	0.081489	0.341077	0.489614	0.104*	0.1450 (13)
C142	0.019 (2)	0.350 (2)	0.4257 (11)	0.093 (5)	0.1450 (13)
H142	−0.026879	0.316865	0.437788	0.111*	0.1450 (13)
C143	0.022 (2)	0.383 (2)	0.3772 (10)	0.097 (6)	0.1450 (13)
H143	−0.021529	0.373262	0.356178	0.117*	0.1450 (13)
N16	0.4282 (3)	0.8108 (2)	0.31066 (12)	0.0444 (7)	
H16	0.383372	0.853116	0.293116	0.053*	
C94	0.5230 (3)	0.7746 (3)	0.29105 (15)	0.0467 (9)	
H94	0.540186	0.794856	0.258802	0.056*	
C95	0.5930 (4)	0.7099 (3)	0.31728 (17)	0.0532 (10)	
H95	0.659532	0.683591	0.303687	0.064*	
C96	0.5662 (4)	0.6820 (4)	0.3648 (2)	0.0732 (16)	
H96	0.615229	0.637713	0.384091	0.088*	
C97	0.4690 (4)	0.7187 (4)	0.38343 (19)	0.0781 (18)	
H97	0.449337	0.698679	0.415347	0.094*	
C98	0.4007 (4)	0.7845 (3)	0.35542 (16)	0.0562 (11)	
H98	0.333483	0.811390	0.368089	0.067*	
N17	0.4739 (8)	1.2711 (9)	0.4138 (4)	0.079 (2)	0.613 (9)
C99	0.5597 (10)	1.2690 (12)	0.3889 (5)	0.075 (2)	0.613 (9)
H99	0.608603	1.289865	0.401019	0.090*	0.613 (9)
C100	0.5802 (10)	1.2375 (11)	0.3458 (5)	0.085 (3)	0.613 (9)
H100	0.629640	1.253317	0.323214	0.103*	0.613 (9)
C101	0.5290 (8)	1.1833 (9)	0.3358 (4)	0.082 (3)	0.613 (9)
H101	0.558128	1.138494	0.315870	0.099*	0.613 (9)
C102	0.4286 (9)	1.1986 (9)	0.3573 (4)	0.086 (3)	0.613 (9)
H102	0.376811	1.184269	0.343226	0.103*	0.613 (9)
C103	0.4090 (9)	1.2348 (9)	0.3992 (4)	0.081 (3)	0.613 (9)
H103	0.348202	1.234022	0.418134	0.098*	0.613 (9)
N24	0.5123 (13)	1.2593 (16)	0.4148 (7)	0.080 (3)	0.387 (9)
C134	0.5869 (16)	1.2620 (19)	0.3823 (8)	0.075 (3)	0.387 (9)
H134	0.632478	1.293862	0.386566	0.089*	0.387 (9)
C135	0.6007 (14)	1.2205 (18)	0.3426 (8)	0.080 (3)	0.387 (9)
H135	0.666145	1.196680	0.327380	0.096*	0.387 (9)
C136	0.5120 (14)	1.2164 (14)	0.3270 (6)	0.083 (3)	0.387 (9)
H136	0.489454	1.237078	0.295312	0.099*	0.387 (9)
C137	0.4575 (15)	1.1768 (14)	0.3650 (6)	0.084 (3)	0.387 (9)
H137	0.433010	1.128266	0.363045	0.101*	0.387 (9)
C138	0.4455 (15)	1.2193 (17)	0.4050 (7)	0.085 (3)	0.387 (9)
H138	0.387595	1.220194	0.426413	0.102*	0.387 (9)
N18	−0.2501 (3)	0.6346 (3)	0.14728 (16)	0.0629 (11)	
C104	−0.2281 (4)	0.5461 (4)	0.1506 (2)	0.0673 (14)	
H104	−0.211733	0.512153	0.181211	0.081*	
C105	−0.2276 (5)	0.5021 (4)	0.1139 (2)	0.0752 (15)	
H105	−0.212683	0.439485	0.118952	0.090*	
C106	−0.2486 (5)	0.5484 (4)	0.0696 (2)	0.0790 (17)	
H106	−0.247807	0.519069	0.042848	0.095*	
C107	−0.2715 (7)	0.6412 (5)	0.0642 (2)	0.098 (2)	
H107	−0.287083	0.676994	0.034027	0.118*	
C108	−0.2702 (6)	0.6767 (4)	0.1042 (3)	0.103 (3)	
H108	−0.285688	0.739347	0.100486	0.123*	
N19	0.3822 (4)	0.5784 (3)	0.08496 (19)	0.0761 (13)	
C109	0.4613 (4)	0.5973 (4)	0.0972 (2)	0.0683 (14)	
H109	0.527073	0.561506	0.087218	0.082*	
C110	0.4598 (5)	0.6624 (5)	0.1226 (2)	0.0816 (18)	
H110	0.520847	0.670767	0.131416	0.098*	
C111	0.3563 (4)	0.7196 (3)	0.13569 (16)	0.0612 (12)	
H111	0.346385	0.768304	0.152668	0.073*	
C112	0.2766 (4)	0.6981 (3)	0.12195 (18)	0.0572 (12)	
H112	0.208216	0.732418	0.129156	0.069*	
C113	0.2936 (5)	0.6280 (4)	0.0980 (2)	0.0706 (14)	
H113	0.235096	0.614288	0.090230	0.085*	
N20	0.3892 (6)	0.2974 (6)	0.0540 (3)	0.117 (2)	
C114	0.4355 (7)	0.2136 (8)	0.0478 (4)	0.124 (4)	
H114	0.469918	0.201196	0.017797	0.149*	
C115	0.4353 (8)	0.1419 (7)	0.0846 (5)	0.140 (4)	
H115	0.472436	0.082476	0.080394	0.167*	
C116	0.3796 (7)	0.1618 (7)	0.1262 (3)	0.109 (3)	
H116	0.374375	0.116507	0.151639	0.131*	
C117	0.3317 (8)	0.2491 (8)	0.1301 (4)	0.132 (4)	
H117	0.291830	0.264837	0.158519	0.158*	
C118	0.3401 (7)	0.3132 (7)	0.0942 (4)	0.115 (3)	
H118	0.308150	0.373196	0.098950	0.138*	
N21	0.4407 (18)	0.4449 (14)	0.5223 (8)	0.067 (5)	0.396 (4)
C119	0.483 (3)	0.4423 (19)	0.4794 (8)	0.062 (5)	0.396 (4)
H119	0.479924	0.398030	0.461497	0.074*	0.396 (4)
C120	0.533 (4)	0.502 (2)	0.4593 (9)	0.062 (5)	0.396 (4)
H120	0.564735	0.499128	0.427853	0.074*	0.396 (4)
C121	0.5383 (18)	0.5687 (15)	0.4854 (10)	0.056 (4)	0.396 (4)
H121	0.562446	0.616830	0.471222	0.067*	0.396 (4)
C122	0.506 (2)	0.5588 (17)	0.5322 (7)	0.052 (4)	0.396 (4)
H122	0.515503	0.596258	0.552946	0.063*	0.396 (4)
C123	0.461 (4)	0.497 (2)	0.5494 (8)	0.056 (4)	0.396 (4)
H123	0.442092	0.489824	0.582721	0.067*	0.396 (4)

### Bis(pyridinium) diaquadipyridinehexakis[µ3-salicylhydroximato(3-)]bis[µ2-salicylhydroximato(1-)]hexaaluminiumgadolinium–pyridine–water (1/7.396/1) (1) Atomic displacement parameters (Å^2^)

**Table:**

	*U*^11^	*U*^22^	*U*^33^	*U*^12^	*U*^13^	*U*^23^
Al1	0.0274 (4)	0.0208 (4)	0.0171 (4)	−0.0046 (3)	−0.0018 (3)	−0.0033 (3)
Al2	0.0299 (5)	0.0266 (4)	0.0231 (4)	−0.0094 (4)	0.0018 (3)	−0.0028 (3)
Al3	0.0295 (5)	0.0216 (4)	0.0275 (4)	−0.0031 (3)	−0.0047 (4)	−0.0076 (3)
Al5	0.0511 (6)	0.0268 (5)	0.0187 (4)	−0.0107 (4)	0.0009 (4)	−0.0024 (3)
Al6	0.0322 (5)	0.0228 (4)	0.0234 (4)	−0.0127 (4)	0.0047 (3)	−0.0056 (3)
O1	0.0270 (10)	0.0239 (9)	0.0199 (9)	−0.0068 (8)	−0.0010 (8)	−0.0029 (7)
O2	0.0289 (11)	0.0327 (11)	0.0185 (9)	−0.0073 (9)	−0.0012 (8)	−0.0038 (8)
O3	0.0398 (13)	0.0455 (14)	0.0244 (10)	−0.0239 (11)	0.0049 (9)	−0.0058 (9)
O4	0.0301 (11)	0.0195 (9)	0.0251 (10)	−0.0059 (8)	0.0011 (8)	−0.0021 (7)
O5	0.0260 (11)	0.0293 (11)	0.0310 (11)	−0.0077 (9)	−0.0005 (9)	−0.0030 (9)
O7	0.0349 (11)	0.0249 (10)	0.0166 (9)	−0.0036 (9)	−0.0056 (8)	−0.0007 (7)
O8	0.0316 (11)	0.0361 (12)	0.0249 (10)	−0.0085 (9)	−0.0081 (9)	−0.0045 (9)
O9	0.0251 (10)	0.0232 (9)	0.0232 (9)	−0.0061 (8)	−0.0030 (8)	−0.0006 (7)
Gd1	0.02211 (14)	0.01892 (7)	0.01705 (7)	−0.00647 (9)	0.00091 (8)	−0.00238 (5)
Al4	0.0233 (6)	0.0187 (6)	0.0198 (5)	−0.0071 (4)	−0.0038 (4)	−0.0008 (4)
O10	0.0306 (13)	0.0196 (12)	0.0221 (11)	−0.0054 (10)	−0.0030 (10)	−0.0011 (9)
N4	0.0334 (15)	0.0215 (13)	0.0249 (13)	−0.0064 (11)	−0.0031 (11)	0.0018 (10)
O11	0.0267 (12)	0.0208 (11)	0.0276 (12)	−0.0074 (9)	−0.0027 (10)	−0.0005 (9)
C22	0.0320 (16)	0.0204 (14)	0.0246 (15)	−0.0078 (12)	−0.0057 (13)	−0.0016 (12)
C23	0.041 (2)	0.0216 (15)	0.0345 (18)	−0.0064 (14)	−0.0085 (15)	0.0001 (13)
C24	0.035 (2)	0.0262 (17)	0.056 (2)	−0.0050 (15)	−0.0083 (18)	−0.0062 (16)
C25	0.043 (2)	0.030 (2)	0.079 (3)	0.0010 (18)	−0.013 (2)	−0.010 (2)
C26	0.058 (3)	0.029 (2)	0.086 (4)	0.003 (2)	−0.028 (3)	0.000 (2)
C27	0.078 (3)	0.029 (2)	0.061 (3)	−0.005 (2)	−0.027 (3)	0.010 (2)
C28	0.059 (3)	0.0277 (18)	0.042 (2)	−0.0076 (18)	−0.015 (2)	0.0047 (16)
O12	0.077 (3)	0.0349 (17)	0.0446 (19)	−0.0068 (16)	0.0039 (17)	0.0170 (14)
O25	0.0258 (13)	0.0207 (13)	0.026 (2)	−0.0068 (10)	−0.0020 (11)	−0.0044 (13)
N9	0.0268 (14)	0.0205 (14)	0.0252 (17)	−0.0083 (12)	−0.0051 (11)	−0.0026 (12)
O26	0.0367 (15)	0.0173 (15)	0.040 (2)	−0.0044 (13)	−0.0117 (13)	−0.0073 (15)
C57	0.0331 (17)	0.0185 (16)	0.0261 (18)	−0.0055 (14)	−0.0090 (13)	−0.0032 (14)
C58	0.0346 (17)	0.0210 (16)	0.0320 (18)	−0.0110 (14)	−0.0084 (14)	−0.0029 (14)
C59	0.041 (2)	0.0251 (18)	0.050 (2)	−0.0140 (16)	−0.0092 (17)	−0.0051 (16)
C60	0.046 (2)	0.0308 (19)	0.065 (3)	−0.0212 (18)	−0.008 (2)	−0.0074 (19)
C61	0.041 (2)	0.034 (2)	0.061 (3)	−0.0201 (18)	−0.0035 (19)	−0.0091 (19)
C62	0.0310 (17)	0.0294 (18)	0.0420 (19)	−0.0133 (15)	−0.0054 (15)	−0.0063 (16)
C63	0.0347 (17)	0.0233 (15)	0.0259 (15)	−0.0116 (14)	−0.0057 (13)	−0.0043 (13)
O27	0.0290 (12)	0.0220 (13)	0.0278 (12)	−0.0107 (11)	−0.0067 (10)	−0.0001 (11)
N11	0.0300 (16)	0.0243 (14)	0.0320 (16)	−0.0098 (12)	0.0030 (13)	−0.0050 (12)
C69	0.0335 (19)	0.0329 (18)	0.040 (2)	−0.0069 (15)	0.0001 (15)	−0.0007 (15)
C70	0.043 (2)	0.034 (2)	0.054 (2)	−0.0032 (17)	−0.0047 (19)	−0.0002 (18)
C71	0.035 (2)	0.045 (2)	0.054 (2)	−0.0020 (17)	−0.0033 (18)	−0.0120 (19)
C72	0.0301 (19)	0.056 (2)	0.050 (2)	−0.0065 (17)	0.0050 (17)	−0.0024 (19)
C73	0.0301 (18)	0.0411 (19)	0.040 (2)	−0.0111 (15)	0.0010 (15)	0.0020 (16)
Gd1B	0.02211 (14)	0.01892 (7)	0.01705 (7)	−0.00647 (9)	0.00091 (8)	−0.00238 (5)
Al4B	0.029 (3)	0.028 (3)	0.027 (3)	−0.012 (2)	−0.007 (2)	−0.005 (3)
O10B	0.036 (5)	0.026 (5)	0.024 (4)	−0.007 (4)	−0.009 (4)	−0.003 (4)
N4B	0.033 (4)	0.024 (4)	0.029 (4)	−0.005 (4)	−0.005 (4)	−0.003 (4)
O11B	0.028 (4)	0.026 (4)	0.028 (4)	−0.005 (4)	−0.003 (4)	−0.002 (4)
C22B	0.030 (4)	0.027 (4)	0.031 (4)	−0.006 (4)	−0.006 (4)	−0.001 (4)
C23B	0.030 (4)	0.040 (5)	0.044 (5)	−0.007 (4)	0.002 (4)	−0.002 (4)
C24B	0.031 (5)	0.043 (5)	0.054 (5)	−0.006 (5)	0.000 (5)	−0.004 (5)
C25B	0.033 (5)	0.048 (5)	0.056 (5)	−0.005 (5)	0.004 (5)	−0.007 (5)
C26B	0.033 (5)	0.046 (5)	0.055 (5)	−0.008 (5)	0.003 (5)	−0.008 (5)
C27B	0.032 (4)	0.039 (4)	0.048 (4)	−0.008 (3)	0.000 (4)	0.000 (4)
C28B	0.033 (3)	0.037 (3)	0.044 (4)	−0.005 (3)	0.002 (3)	0.000 (3)
O12B	0.031 (5)	0.029 (4)	0.037 (5)	−0.007 (4)	0.004 (4)	−0.002 (4)
O25B	0.030 (5)	0.022 (5)	0.023 (6)	−0.007 (5)	−0.003 (5)	0.001 (5)
N9B	0.027 (4)	0.020 (4)	0.023 (4)	−0.009 (4)	−0.006 (4)	−0.005 (4)
O26B	0.032 (5)	0.019 (5)	0.029 (5)	−0.007 (4)	−0.012 (4)	−0.001 (5)
C57B	0.032 (4)	0.021 (4)	0.030 (4)	−0.006 (4)	−0.008 (4)	−0.004 (4)
C58B	0.036 (4)	0.022 (4)	0.032 (4)	−0.012 (4)	−0.010 (3)	−0.006 (4)
C59B	0.037 (4)	0.028 (4)	0.045 (4)	−0.011 (4)	−0.008 (4)	−0.006 (4)
C60B	0.039 (4)	0.030 (4)	0.053 (4)	−0.016 (4)	−0.008 (4)	−0.006 (4)
C61B	0.038 (4)	0.031 (4)	0.048 (4)	−0.013 (4)	−0.004 (4)	−0.005 (4)
C62B	0.032 (4)	0.030 (4)	0.036 (4)	−0.013 (4)	−0.008 (4)	−0.006 (4)
C63B	0.030 (4)	0.026 (4)	0.031 (4)	−0.011 (4)	−0.008 (4)	−0.006 (4)
O27B	0.029 (4)	0.028 (4)	0.028 (4)	−0.009 (4)	−0.008 (4)	−0.004 (4)
O13	0.0554 (15)	0.0301 (11)	0.0218 (10)	−0.0137 (11)	−0.0029 (10)	−0.0053 (9)
O14	0.0612 (17)	0.0387 (13)	0.0244 (11)	−0.0108 (12)	−0.0056 (11)	−0.0024 (10)
O15	0.141 (4)	0.101 (3)	0.064 (2)	−0.077 (3)	−0.042 (3)	0.006 (2)
O16	0.0385 (12)	0.0274 (10)	0.0193 (9)	−0.0117 (9)	0.0037 (8)	−0.0026 (8)
O17	0.0366 (12)	0.0351 (12)	0.0257 (11)	−0.0133 (10)	0.0048 (9)	−0.0037 (9)
O18	0.0647 (18)	0.0379 (13)	0.0211 (11)	−0.0036 (12)	0.0054 (11)	−0.0021 (9)
O19	0.0465 (13)	0.0209 (9)	0.0208 (10)	−0.0105 (9)	−0.0014 (9)	−0.0028 (8)
O20	0.0622 (17)	0.0286 (11)	0.0220 (10)	−0.0156 (11)	0.0074 (10)	−0.0014 (9)
O21	0.0443 (13)	0.0251 (10)	0.0288 (11)	−0.0179 (10)	0.0054 (9)	−0.0047 (8)
O22	0.0499 (14)	0.0217 (10)	0.0177 (9)	−0.0092 (9)	0.0012 (9)	−0.0025 (7)
O23	0.0429 (13)	0.0264 (10)	0.0279 (11)	−0.0183 (10)	0.0074 (9)	−0.0077 (8)
O24	0.0341 (11)	0.0225 (9)	0.0213 (9)	−0.0072 (8)	0.0011 (8)	−0.0052 (7)
O28	0.0392 (13)	0.0261 (11)	0.0291 (11)	−0.0114 (10)	−0.0012 (9)	−0.0066 (9)
O29	0.0403 (14)	0.0359 (13)	0.0410 (14)	−0.0145 (11)	−0.0015 (11)	−0.0082 (11)
O30	0.156 (6)	0.246 (10)	0.205 (8)	−0.078 (7)	0.019 (6)	−0.164 (8)
N1	0.0271 (12)	0.0251 (11)	0.0216 (11)	−0.0088 (10)	−0.0008 (9)	−0.0022 (9)
N2	0.0287 (13)	0.0199 (11)	0.0249 (12)	−0.0032 (9)	0.0010 (10)	−0.0036 (9)
N3	0.0308 (13)	0.0211 (11)	0.0173 (10)	−0.0029 (9)	−0.0043 (9)	−0.0020 (8)
N5	0.062 (2)	0.0407 (16)	0.0233 (13)	−0.0182 (15)	−0.0029 (13)	−0.0104 (12)
N6	0.0477 (17)	0.0310 (13)	0.0201 (12)	−0.0166 (12)	0.0051 (11)	−0.0031 (10)
N7	0.0456 (16)	0.0205 (11)	0.0226 (12)	−0.0111 (11)	0.0009 (11)	−0.0015 (9)
N8	0.0313 (13)	0.0239 (11)	0.0167 (10)	−0.0070 (10)	0.0006 (9)	−0.0040 (8)
C1	0.0315 (15)	0.0247 (13)	0.0220 (13)	−0.0090 (11)	−0.0023 (11)	−0.0009 (10)
C2	0.0310 (16)	0.0331 (15)	0.0254 (14)	−0.0108 (13)	−0.0011 (12)	−0.0040 (12)
C3	0.0328 (17)	0.0435 (19)	0.0290 (16)	−0.0094 (14)	−0.0037 (13)	−0.0048 (13)
C4	0.0351 (18)	0.061 (2)	0.0365 (18)	−0.0137 (17)	−0.0070 (15)	−0.0116 (17)
C5	0.0346 (19)	0.062 (3)	0.048 (2)	−0.0174 (18)	−0.0064 (16)	−0.0164 (19)
C6	0.0345 (18)	0.055 (2)	0.0409 (19)	−0.0221 (17)	0.0018 (15)	−0.0109 (16)
C7	0.0325 (16)	0.0342 (16)	0.0309 (15)	−0.0133 (13)	−0.0022 (12)	−0.0036 (12)
C8	0.0257 (14)	0.0260 (13)	0.0264 (13)	−0.0039 (11)	0.0025 (11)	−0.0058 (11)
C9	0.029 (2)	0.0255 (16)	0.030 (3)	−0.0031 (14)	−0.001 (2)	−0.0043 (15)
C10	0.028 (2)	0.0347 (19)	0.038 (2)	−0.0021 (16)	−0.0019 (17)	−0.0059 (17)
C11	0.041 (2)	0.034 (2)	0.043 (2)	0.0049 (17)	−0.0077 (18)	−0.0041 (18)
C12	0.048 (2)	0.0248 (17)	0.046 (2)	0.0020 (16)	−0.0106 (19)	−0.0016 (16)
C13	0.046 (2)	0.0228 (17)	0.040 (2)	−0.0014 (16)	−0.0053 (18)	−0.0057 (16)
C14	0.033 (2)	0.0232 (15)	0.032 (2)	−0.0038 (14)	−0.0019 (18)	−0.0067 (14)
O6	0.0366 (12)	0.0230 (10)	0.0380 (12)	−0.0041 (9)	−0.0074 (10)	−0.0072 (9)
C8B	0.0257 (14)	0.0260 (13)	0.0264 (13)	−0.0039 (11)	0.0025 (11)	−0.0058 (11)
C9B	0.029 (4)	0.026 (4)	0.033 (4)	−0.004 (4)	−0.002 (4)	−0.006 (4)
C10B	0.033 (5)	0.031 (4)	0.038 (5)	0.000 (4)	−0.003 (5)	−0.006 (4)
C11B	0.040 (5)	0.031 (4)	0.043 (5)	0.003 (4)	−0.007 (5)	−0.004 (5)
C12B	0.045 (5)	0.029 (4)	0.046 (5)	0.003 (4)	−0.006 (5)	−0.004 (5)
C13B	0.041 (5)	0.024 (4)	0.042 (5)	−0.004 (4)	−0.005 (5)	−0.008 (4)
C14B	0.034 (4)	0.023 (4)	0.037 (4)	−0.004 (4)	−0.006 (4)	−0.008 (4)
O6B	0.0366 (12)	0.0230 (10)	0.0380 (12)	−0.0041 (9)	−0.0074 (10)	−0.0072 (9)
C15	0.0266 (14)	0.0247 (13)	0.0234 (13)	−0.0036 (11)	−0.0041 (11)	−0.0067 (10)
C16	0.0309 (15)	0.0264 (14)	0.0219 (13)	−0.0067 (12)	−0.0023 (11)	−0.0056 (11)
C17	0.0315 (16)	0.0394 (17)	0.0295 (15)	−0.0140 (14)	−0.0039 (12)	−0.0034 (13)
C18	0.0330 (17)	0.0467 (19)	0.0297 (16)	−0.0194 (15)	−0.0005 (13)	−0.0029 (14)
C19	0.0368 (17)	0.0360 (16)	0.0229 (14)	−0.0148 (14)	−0.0002 (12)	−0.0018 (12)
C20	0.0330 (16)	0.0269 (14)	0.0229 (13)	−0.0081 (12)	−0.0058 (11)	−0.0047 (11)
C21	0.0285 (14)	0.0214 (12)	0.0225 (13)	−0.0071 (11)	−0.0015 (11)	−0.0052 (10)
C29	0.056 (2)	0.0421 (19)	0.0225 (15)	−0.0127 (17)	0.0012 (14)	−0.0115 (13)
C30	0.054 (2)	0.066 (3)	0.0273 (17)	−0.012 (2)	−0.0009 (16)	−0.0161 (17)
C31	0.066 (3)	0.068 (3)	0.053 (3)	−0.003 (2)	−0.015 (2)	−0.018 (2)
C32	0.079 (4)	0.092 (4)	0.065 (3)	0.005 (3)	−0.029 (3)	−0.023 (3)
C33	0.066 (3)	0.113 (5)	0.066 (3)	−0.003 (3)	−0.018 (3)	−0.047 (4)
C34	0.068 (3)	0.112 (5)	0.051 (3)	−0.033 (3)	−0.003 (2)	−0.036 (3)
C35	0.076 (3)	0.088 (4)	0.032 (2)	−0.036 (3)	−0.003 (2)	−0.020 (2)
C36	0.0419 (18)	0.0349 (16)	0.0228 (14)	−0.0191 (14)	0.0049 (12)	−0.0050 (12)
C37	0.058 (2)	0.0356 (17)	0.0258 (15)	−0.0160 (16)	0.0107 (15)	−0.0081 (13)
C38	0.055 (2)	0.059 (3)	0.0339 (19)	−0.013 (2)	0.0095 (17)	−0.0104 (17)
C39	0.065 (3)	0.086 (4)	0.043 (2)	−0.004 (3)	0.019 (2)	−0.016 (2)
C40	0.104 (5)	0.081 (4)	0.033 (2)	−0.004 (3)	0.026 (3)	−0.010 (2)
C41	0.091 (4)	0.059 (3)	0.0243 (18)	0.000 (3)	0.014 (2)	−0.0056 (17)
C42	0.065 (3)	0.0365 (18)	0.0260 (16)	−0.0145 (18)	0.0090 (16)	−0.0057 (13)
C43	0.0469 (19)	0.0262 (14)	0.0232 (14)	−0.0131 (13)	0.0023 (13)	−0.0007 (11)
C44	0.050 (2)	0.0261 (15)	0.0299 (16)	−0.0147 (14)	0.0065 (14)	−0.0011 (12)
C45	0.063 (3)	0.0348 (18)	0.040 (2)	−0.0196 (18)	0.0137 (18)	−0.0022 (15)
C46	0.068 (3)	0.042 (2)	0.051 (2)	−0.029 (2)	0.020 (2)	−0.0007 (18)
C47	0.058 (3)	0.0342 (19)	0.062 (3)	−0.0248 (18)	0.014 (2)	−0.0046 (17)
C48	0.044 (2)	0.0311 (16)	0.045 (2)	−0.0174 (15)	0.0080 (16)	−0.0088 (14)
C49	0.0372 (17)	0.0264 (14)	0.0311 (15)	−0.0135 (13)	0.0040 (13)	−0.0003 (12)
C50	0.0286 (14)	0.0273 (14)	0.0238 (13)	−0.0131 (11)	0.0033 (11)	−0.0075 (11)
C51	0.0403 (17)	0.0296 (15)	0.0240 (14)	−0.0167 (13)	0.0050 (12)	−0.0089 (11)
C52	0.088 (3)	0.043 (2)	0.0347 (19)	−0.039 (2)	0.0144 (19)	−0.0156 (16)
C53	0.109 (4)	0.047 (2)	0.039 (2)	−0.044 (3)	0.018 (2)	−0.0238 (18)
C54	0.078 (3)	0.042 (2)	0.0294 (17)	−0.023 (2)	0.0134 (18)	−0.0174 (15)
C55	0.0456 (19)	0.0294 (15)	0.0251 (14)	−0.0117 (14)	0.0048 (13)	−0.0066 (12)
C56	0.0268 (14)	0.0274 (14)	0.0241 (13)	−0.0073 (11)	−0.0007 (11)	−0.0072 (11)
N10	0.064 (2)	0.0300 (14)	0.0286 (14)	0.0051 (14)	−0.0080 (14)	−0.0113 (11)
C64	0.081 (3)	0.056 (3)	0.046 (2)	−0.002 (2)	−0.019 (2)	−0.027 (2)
C65	0.168 (7)	0.077 (4)	0.043 (3)	−0.012 (4)	−0.024 (4)	−0.030 (3)
C66	0.258 (11)	0.065 (4)	0.030 (2)	−0.036 (5)	0.010 (4)	−0.018 (2)
C67	0.260 (10)	0.046 (3)	0.033 (2)	−0.039 (4)	0.042 (4)	−0.017 (2)
C68	0.122 (5)	0.0327 (19)	0.0321 (19)	−0.014 (2)	0.016 (2)	−0.0114 (16)
N12	0.0429 (19)	0.0445 (19)	0.053 (2)	−0.0049 (15)	0.0016 (15)	0.0093 (16)
C74	0.051 (3)	0.093 (4)	0.035 (2)	−0.010 (3)	−0.0028 (18)	−0.010 (2)
C75	0.063 (3)	0.067 (3)	0.068 (3)	−0.009 (3)	0.010 (3)	−0.029 (3)
C76	0.058 (3)	0.051 (3)	0.076 (4)	0.003 (2)	0.001 (3)	0.014 (2)
C77	0.061 (3)	0.098 (4)	0.043 (2)	−0.017 (3)	−0.018 (2)	0.004 (3)
C78	0.057 (3)	0.055 (3)	0.062 (3)	−0.019 (2)	0.001 (2)	−0.021 (2)
N13	0.0521 (19)	0.0368 (16)	0.0363 (16)	−0.0169 (14)	0.0025 (14)	−0.0087 (12)
C79	0.062 (3)	0.0368 (19)	0.044 (2)	−0.0160 (18)	0.0050 (18)	−0.0133 (16)
C80	0.057 (3)	0.036 (2)	0.074 (3)	−0.0141 (19)	−0.001 (2)	−0.015 (2)
C81	0.080 (4)	0.048 (3)	0.104 (5)	−0.036 (3)	0.008 (3)	−0.002 (3)
C82	0.100 (5)	0.084 (4)	0.068 (3)	−0.054 (4)	0.027 (3)	−0.010 (3)
C83	0.068 (3)	0.056 (3)	0.043 (2)	−0.028 (2)	0.008 (2)	−0.0138 (19)
N14	0.111 (5)	0.108 (5)	0.060 (4)	−0.062 (4)	−0.023 (4)	−0.010 (4)
C84	0.117 (5)	0.107 (6)	0.059 (4)	−0.057 (4)	−0.017 (4)	−0.008 (4)
C85	0.125 (5)	0.104 (5)	0.062 (4)	−0.066 (4)	−0.012 (4)	−0.004 (4)
C86	0.144 (6)	0.110 (6)	0.075 (5)	−0.071 (5)	−0.004 (4)	−0.006 (4)
C87	0.143 (6)	0.114 (5)	0.073 (5)	−0.072 (5)	−0.009 (5)	−0.009 (4)
C88	0.132 (6)	0.112 (6)	0.066 (5)	−0.063 (5)	−0.013 (4)	−0.012 (4)
N22	0.119 (5)	0.114 (6)	0.065 (5)	−0.054 (5)	−0.024 (4)	−0.011 (5)
C124	0.119 (5)	0.110 (6)	0.064 (5)	−0.054 (5)	−0.019 (5)	−0.012 (5)
C125	0.133 (6)	0.109 (6)	0.069 (5)	−0.060 (5)	−0.011 (5)	−0.013 (5)
C126	0.144 (6)	0.115 (6)	0.076 (5)	−0.057 (5)	−0.006 (5)	−0.010 (5)
C127	0.145 (6)	0.114 (6)	0.075 (5)	−0.059 (5)	−0.008 (5)	−0.010 (5)
C128	0.131 (6)	0.116 (6)	0.068 (5)	−0.058 (5)	−0.016 (5)	−0.008 (5)
N15	0.075 (2)	0.054 (2)	0.051 (2)	−0.020 (2)	−0.0067 (19)	0.0158 (18)
C89	0.072 (4)	0.055 (4)	0.059 (3)	−0.017 (3)	−0.004 (3)	0.017 (3)
C90	0.084 (4)	0.054 (4)	0.055 (3)	−0.013 (4)	0.005 (4)	0.015 (3)
C91	0.090 (4)	0.072 (4)	0.071 (4)	−0.016 (4)	0.006 (4)	0.014 (4)
C92	0.084 (4)	0.080 (4)	0.083 (4)	−0.016 (4)	0.002 (4)	0.013 (4)
C93	0.083 (4)	0.072 (4)	0.068 (4)	−0.009 (4)	−0.007 (3)	0.014 (4)
N23	0.075 (2)	0.054 (2)	0.051 (2)	−0.020 (2)	−0.0067 (19)	0.0158 (18)
C129	0.072 (4)	0.055 (4)	0.056 (4)	−0.018 (4)	−0.007 (4)	0.013 (4)
C130	0.077 (5)	0.058 (4)	0.056 (4)	−0.017 (4)	0.000 (4)	0.016 (4)
C131	0.086 (4)	0.071 (5)	0.072 (4)	−0.015 (4)	0.005 (4)	0.015 (4)
C132	0.082 (4)	0.076 (5)	0.076 (4)	−0.014 (4)	0.001 (4)	0.013 (4)
C133	0.080 (4)	0.066 (4)	0.066 (4)	−0.015 (4)	−0.007 (4)	0.012 (4)
N25	0.091 (7)	0.082 (7)	0.081 (7)	−0.012 (7)	−0.015 (7)	0.015 (7)
C139	0.085 (5)	0.069 (5)	0.068 (5)	−0.014 (5)	−0.008 (5)	0.012 (5)
C140	0.082 (3)	0.067 (3)	0.066 (3)	−0.014 (3)	−0.002 (3)	0.014 (3)
C141	0.088 (5)	0.077 (5)	0.078 (5)	−0.014 (5)	0.002 (5)	0.012 (5)
C142	0.090 (7)	0.086 (7)	0.086 (7)	−0.016 (7)	−0.001 (7)	0.010 (7)
C143	0.094 (9)	0.090 (9)	0.088 (9)	−0.012 (9)	−0.008 (9)	0.015 (9)
N16	0.0479 (19)	0.0414 (17)	0.0461 (18)	−0.0158 (15)	−0.0158 (15)	0.0005 (14)
C94	0.046 (2)	0.056 (2)	0.041 (2)	−0.0236 (19)	−0.0059 (17)	0.0013 (17)
C95	0.047 (2)	0.052 (2)	0.059 (3)	−0.0157 (19)	−0.002 (2)	−0.001 (2)
C96	0.059 (3)	0.073 (3)	0.062 (3)	0.001 (3)	−0.005 (2)	0.021 (3)
C97	0.067 (3)	0.085 (4)	0.049 (3)	0.006 (3)	0.004 (2)	0.023 (3)
C98	0.055 (3)	0.052 (2)	0.048 (2)	−0.003 (2)	−0.0020 (19)	0.0064 (19)
N17	0.080 (5)	0.082 (4)	0.074 (4)	−0.020 (5)	0.011 (4)	−0.030 (3)
C99	0.073 (5)	0.080 (4)	0.073 (4)	−0.027 (5)	0.011 (4)	−0.011 (4)
C100	0.075 (5)	0.101 (6)	0.073 (4)	−0.020 (5)	0.013 (4)	−0.017 (4)
C101	0.071 (4)	0.107 (6)	0.067 (4)	−0.019 (4)	0.011 (4)	−0.028 (4)
C102	0.074 (5)	0.109 (6)	0.068 (4)	−0.019 (5)	0.009 (4)	−0.019 (4)
C103	0.074 (5)	0.095 (5)	0.065 (4)	−0.017 (5)	0.016 (4)	−0.013 (4)
N24	0.079 (6)	0.089 (5)	0.069 (4)	−0.019 (6)	0.013 (5)	−0.022 (4)
C134	0.073 (6)	0.083 (5)	0.069 (5)	−0.028 (5)	0.013 (5)	−0.016 (4)
C135	0.072 (5)	0.095 (6)	0.069 (5)	−0.018 (5)	0.010 (5)	−0.022 (4)
C136	0.074 (5)	0.103 (6)	0.067 (5)	−0.020 (5)	0.007 (4)	−0.021 (5)
C137	0.079 (6)	0.100 (6)	0.070 (5)	−0.022 (5)	0.009 (5)	−0.022 (5)
C138	0.081 (6)	0.097 (5)	0.071 (4)	−0.024 (5)	0.014 (5)	−0.014 (4)
N18	0.057 (2)	0.070 (3)	0.064 (3)	−0.013 (2)	−0.018 (2)	−0.018 (2)
C104	0.074 (3)	0.056 (3)	0.076 (4)	−0.024 (3)	−0.017 (3)	−0.007 (3)
C105	0.078 (4)	0.066 (3)	0.077 (4)	−0.017 (3)	−0.020 (3)	0.001 (3)
C106	0.077 (4)	0.084 (4)	0.078 (4)	−0.010 (3)	−0.016 (3)	−0.041 (3)
C107	0.153 (7)	0.078 (4)	0.059 (3)	−0.017 (4)	−0.042 (4)	−0.009 (3)
C108	0.133 (6)	0.066 (4)	0.088 (5)	0.020 (4)	−0.055 (4)	−0.022 (3)
N19	0.066 (3)	0.078 (3)	0.080 (3)	−0.013 (2)	−0.003 (2)	−0.016 (3)
C109	0.044 (3)	0.078 (4)	0.072 (3)	0.005 (2)	0.002 (2)	−0.027 (3)
C110	0.067 (4)	0.131 (6)	0.063 (3)	−0.051 (4)	−0.003 (3)	−0.020 (4)
C111	0.086 (4)	0.060 (3)	0.040 (2)	−0.026 (3)	0.004 (2)	−0.007 (2)
C112	0.044 (2)	0.052 (3)	0.063 (3)	−0.0045 (19)	0.003 (2)	0.006 (2)
C113	0.067 (3)	0.062 (3)	0.076 (4)	−0.013 (3)	−0.008 (3)	0.000 (3)
N20	0.099 (5)	0.142 (7)	0.136 (6)	−0.049 (5)	−0.020 (5)	−0.056 (5)
C114	0.110 (7)	0.152 (9)	0.153 (9)	−0.076 (7)	0.025 (6)	−0.087 (8)
C115	0.117 (7)	0.123 (8)	0.210 (13)	−0.040 (6)	−0.037 (8)	−0.087 (9)
C116	0.111 (6)	0.114 (7)	0.115 (7)	−0.030 (5)	−0.053 (5)	−0.030 (5)
C117	0.115 (7)	0.169 (10)	0.092 (6)	−0.003 (7)	−0.031 (5)	−0.030 (7)
C118	0.092 (6)	0.129 (7)	0.112 (7)	0.002 (5)	−0.035 (5)	−0.035 (6)
N21	0.082 (9)	0.072 (7)	0.039 (7)	−0.016 (6)	0.003 (6)	−0.002 (6)
C119	0.080 (9)	0.066 (6)	0.038 (9)	−0.012 (6)	−0.004 (8)	−0.019 (7)
C120	0.070 (8)	0.072 (7)	0.036 (9)	−0.012 (6)	0.003 (9)	−0.011 (7)
C121	0.061 (8)	0.057 (6)	0.048 (9)	−0.022 (6)	0.001 (7)	0.001 (6)
C122	0.073 (7)	0.049 (5)	0.029 (8)	−0.011 (5)	0.001 (7)	−0.011 (6)
C123	0.070 (7)	0.066 (6)	0.022 (6)	−0.011 (6)	0.006 (6)	−0.002 (5)

### Bis(pyridinium) diaquadipyridinehexakis[µ3-salicylhydroximato(3-)]bis[µ2-salicylhydroximato(1-)]hexaaluminiumgadolinium–pyridine–water (1/7.396/1) (1) Geometric parameters (Å, º)

**Table:**

Al1—O24	1.822 (2)	C20—C21	1.403 (4)
Al1—O2	1.876 (2)	C20—H20	0.9500
Al1—O9	1.898 (2)	C29—C30	1.482 (6)
Al1—O1	1.907 (2)	C30—C31	1.379 (7)
Al1—N3	1.977 (3)	C30—C35	1.402 (7)
Al1—N8	2.081 (3)	C31—C32	1.395 (8)
Al2—O3	1.818 (3)	C31—H31	0.9500
Al2—O17	1.877 (2)	C32—C33	1.375 (10)
Al2—O5	1.887 (2)	C32—H32	0.9500
Al2—O4	1.890 (2)	C33—C34	1.360 (9)
Al2—O16	1.942 (2)	C33—H33	0.9500
Al2—N1	2.004 (3)	C34—C35	1.388 (7)
Al2—Gd1B	3.100 (2)	C34—H34	0.9500
Al2—Gd1	3.4536 (11)	C36—C37	1.482 (4)
Al3—O6B	1.807 (2)	C37—C38	1.403 (6)
Al3—O6	1.807 (2)	C37—C42	1.420 (6)
Al3—O26B	1.83 (3)	C38—C39	1.395 (6)
Al3—O26	1.861 (5)	C38—H38	0.9500
Al3—O25	1.870 (3)	C39—C40	1.384 (8)
Al3—N2	1.916 (3)	C39—H39	0.9500
Al3—O28	1.997 (2)	C40—C41	1.378 (8)
Al3—O25B	2.009 (19)	C40—H40	0.9500
Al3—N10	2.113 (3)	C41—C42	1.414 (5)
Al5—O18	1.805 (3)	C41—H41	0.9500
Al5—O20	1.859 (3)	C43—C44	1.476 (5)
Al5—O19	1.904 (2)	C44—C45	1.401 (5)
Al5—O13	1.915 (3)	C44—C49	1.421 (5)
Al5—O14	1.940 (3)	C45—C46	1.381 (6)
Al5—N6	2.028 (3)	C45—H45	0.9500
Al5—Gd1B	3.225 (2)	C46—C47	1.394 (6)
Al5—Gd1	3.4243 (10)	C46—H46	0.9500
Al6—O21	1.804 (2)	C47—C48	1.378 (5)
Al6—O23	1.837 (2)	C47—H47	0.9500
Al6—O22	1.878 (2)	C48—C49	1.408 (5)
Al6—N7	1.934 (3)	C48—H48	0.9500
Al6—O12B	1.980 (17)	C50—C51	1.470 (4)
Al6—O29	1.988 (3)	C51—C52	1.397 (5)
Al6—N11	2.111 (4)	C51—C56	1.404 (4)
O1—N1	1.417 (3)	C52—C53	1.386 (5)
O1—Gd1B	2.366 (3)	C52—H52	0.9500
O1—Gd1	2.388 (2)	C53—C54	1.388 (6)
O2—C1	1.298 (4)	C53—H53	0.9500
O3—C7	1.323 (4)	C54—C55	1.375 (5)
O4—N2	1.394 (3)	C54—H54	0.9500
O4—Gd1B	2.210 (3)	C55—C56	1.411 (4)
O4—Gd1	2.438 (2)	C55—H55	0.9500
O5—C8B	1.289 (4)	N10—C68	1.320 (6)
O5—C8	1.289 (4)	N10—C64	1.350 (6)
O7—N3	1.401 (3)	C64—C65	1.388 (7)
O7—Al4B	1.643 (9)	C64—H64	0.9500
O7—Al4	1.915 (3)	C65—C66	1.361 (11)
O7—Gd1	2.341 (2)	C65—H65	0.9500
O7—Gd1B	2.711 (3)	C66—C67	1.364 (11)
O8—C15	1.298 (4)	C66—H66	0.9500
O8—Al4	1.844 (3)	C67—C68	1.387 (6)
O8—Al4B	2.213 (10)	C67—H67	0.9500
O9—C21	1.341 (4)	C68—H68	0.9500
Gd1—O22	2.398 (2)	N12—C74	1.311 (6)
Gd1—O25	2.401 (3)	N12—C78	1.336 (6)
Gd1—O19	2.428 (2)	N12—H12A	0.8800
Gd1—O16	2.527 (2)	C74—C75	1.361 (7)
Gd1—O13	2.537 (2)	C74—H74	0.9500
Gd1—O10	2.603 (3)	C75—C76	1.376 (8)
Gd1—Al4	3.4813 (14)	C75—H75	0.9500
Al4—O27	1.854 (3)	C76—C77	1.381 (8)
Al4—O11	1.892 (3)	C76—H76	0.9500
Al4—O10	1.943 (3)	C77—C78	1.361 (7)
Al4—N9	1.976 (3)	C77—H77	0.9500
O10—N4	1.368 (4)	C78—H78	0.9500
N4—C22	1.311 (5)	N13—C83	1.307 (5)
N4—H4N	0.8800	N13—C79	1.329 (5)
O11—C22	1.282 (4)	C79—C80	1.371 (6)
C22—C23	1.476 (5)	C79—H79	0.9500
C23—C24	1.383 (6)	C80—C81	1.364 (7)
C23—C28	1.406 (6)	C80—H80	0.9500
C24—C25	1.388 (6)	C81—C82	1.376 (7)
C24—H24	0.9500	C81—H81	0.9500
C25—C26	1.383 (8)	C82—C83	1.387 (6)
C25—H25	0.9500	C82—H82	0.9500
C26—C27	1.364 (9)	C83—H83	0.9500
C26—H26	0.9500	N14—C88	1.314 (11)
C27—C28	1.410 (6)	N14—C84	1.345 (10)
C27—H27	0.9500	C84—C85	1.353 (12)
C28—O12	1.340 (6)	C84—H84	0.9500
O12—H12O	0.8400	C85—C86	1.346 (11)
O25—N9	1.419 (4)	C85—H85	0.9500
N9—C57	1.300 (5)	C86—C87	1.367 (11)
O26—C57	1.307 (5)	C86—H86	0.9500
C57—C58	1.468 (5)	C87—C88	1.372 (12)
C58—C59	1.399 (6)	C87—H87	0.9500
C58—C63	1.402 (5)	C88—H88	0.9500
C59—C60	1.386 (6)	N22—C128	1.325 (14)
C59—H59	0.9500	N22—C124	1.337 (13)
C60—C61	1.388 (7)	C124—C125	1.359 (14)
C60—H60	0.9500	C124—H124	0.9500
C61—C62	1.383 (6)	C125—C126	1.346 (14)
C61—H61	0.9500	C125—H125	0.9500
C62—C63	1.396 (5)	C126—C127	1.385 (14)
C62—H62	0.9500	C126—H126	0.9500
C63—O27	1.343 (4)	C127—C128	1.379 (14)
N11—C69	1.330 (5)	C127—H127	0.9500
N11—C73	1.331 (5)	C128—H128	0.9500
C69—C70	1.376 (6)	N15—C89	1.307 (10)
C69—H69	0.9500	N15—C133	1.314 (13)
C70—C71	1.360 (7)	N15—C129	1.318 (12)
C70—H70	0.9500	N15—C93	1.323 (11)
C71—C72	1.375 (7)	C89—N23	1.307 (10)
C71—H71	0.9500	C89—C90	1.349 (11)
C72—C73	1.381 (6)	C89—C130	1.44 (3)
C72—H72	0.9500	C89—H89	0.9500
C73—H73	0.9500	C90—C129	1.27 (3)
Gd1B—O16	2.114 (3)	C90—C91	1.377 (13)
Gd1B—O19	2.330 (3)	C90—C131	1.59 (3)
Gd1B—O25B	2.383 (15)	C90—H90	0.9500
Gd1B—O22	2.387 (3)	C91—C131	0.71 (2)
Gd1B—O13	2.472 (3)	C91—C130	1.16 (3)
Gd1B—O10B	2.633 (13)	C91—C92	1.416 (12)
Gd1B—N6	2.799 (3)	C91—H91	0.9500
Gd1B—N1	2.951 (3)	C92—C132	0.86 (2)
Al4B—O27B	1.851 (13)	C92—C93	1.371 (11)
Al4B—O11B	1.904 (13)	C92—C131	1.46 (3)
Al4B—O10B	1.922 (14)	C92—C133	1.57 (2)
Al4B—N9B	1.970 (14)	C92—H92	0.9500
O10B—N4B	1.392 (15)	C93—N23	1.323 (11)
N4B—C22B	1.310 (16)	C93—C132	1.40 (2)
N4B—H4B	0.88 (2)	C93—H93	0.9500
O11B—C22B	1.288 (16)	N23—C133	1.314 (13)
C22B—C23B	1.477 (15)	N23—C129	1.318 (12)
C23B—C24B	1.390 (17)	C129—C130	1.355 (13)
C23B—C28B	1.416 (17)	C129—H129	0.9500
C24B—C25B	1.386 (18)	C130—C131	1.372 (14)
C24B—H24B	0.9500	C130—H130	0.9500
C25B—C26B	1.384 (18)	C131—C132	1.397 (13)
C25B—H25B	0.9500	C131—H131	0.9500
C26B—C27B	1.343 (18)	C132—C133	1.377 (14)
C26B—H26B	0.9500	C132—H132	0.9500
C27B—C28B	1.423 (17)	C133—H133	0.9500
C27B—H27B	0.9500	N25—C139	1.3900
C28B—O12B	1.337 (16)	N25—C143	1.3900
O12B—H12P	0.8400	C139—C140	1.3900
O25B—N9B	1.426 (16)	C139—H139	0.9500
N9B—C57B	1.293 (16)	C140—C141	1.3900
O26B—C57B	1.312 (16)	C140—H140	0.9500
C57B—C58B	1.480 (16)	C141—C142	1.3900
C58B—C59B	1.389 (17)	C141—H141	0.9500
C58B—C63B	1.407 (17)	C142—C143	1.3900
C59B—C60B	1.415 (17)	C142—H142	0.9500
C59B—H59B	0.9500	C143—H143	0.9500
C60B—C61B	1.377 (18)	N16—C98	1.325 (6)
C60B—H60B	0.9500	N16—C94	1.352 (6)
C61B—C62B	1.377 (18)	N16—H16	0.8800
C61B—H61B	0.9500	C94—C95	1.347 (6)
C62B—C63B	1.390 (16)	C94—H94	0.9500
C62B—H62B	0.9500	C95—C96	1.396 (7)
C63B—O27B	1.337 (16)	C95—H95	0.9500
O13—N5	1.369 (4)	C96—C97	1.370 (8)
O14—C29	1.271 (5)	C96—H96	0.9500
O15—C35	1.352 (7)	C97—C98	1.369 (6)
O15—H15	0.8400	C97—H97	0.9500
O16—N6	1.440 (3)	C98—H98	0.9500
O17—C36	1.293 (4)	N17—C99	1.306 (10)
O18—C42	1.313 (5)	N17—C103	1.332 (11)
O19—N7	1.412 (3)	C99—C100	1.365 (11)
O20—C43	1.301 (4)	C99—H99	0.9500
O21—C49	1.316 (4)	C100—C101	1.353 (12)
O22—N8	1.423 (3)	C100—H100	0.9500
O23—C50	1.297 (4)	C101—C102	1.415 (11)
O24—C56	1.316 (4)	C101—H101	0.9500
O28—H28A	0.857 (19)	C102—C103	1.369 (11)
O28—H28B	0.832 (19)	C102—H102	0.9500
O29—H29A	0.887 (19)	C103—H103	0.9500
O29—H29B	0.904 (19)	N24—C134	1.314 (13)
O30—H30A	0.85 (2)	N24—C138	1.343 (13)
O30—H30B	0.87 (2)	C134—C135	1.363 (13)
N1—C1	1.310 (4)	C134—H134	0.9500
N2—C8B	1.307 (4)	C135—C136	1.358 (14)
N2—C8	1.307 (4)	C135—H135	0.9500
N3—C15	1.307 (4)	C136—C137	1.427 (14)
N5—C29	1.309 (5)	C136—H136	0.9500
N5—H5N	0.8800	C137—C138	1.383 (13)
N6—C36	1.315 (5)	C137—H137	0.9500
N7—C43	1.308 (4)	C138—H138	0.9500
N8—C50	1.308 (4)	N18—C108	1.303 (8)
C1—C2	1.468 (4)	N18—C104	1.346 (7)
C2—C3	1.398 (4)	C104—C105	1.344 (8)
C2—C7	1.417 (5)	C104—H104	0.9500
C3—C4	1.378 (5)	C105—C106	1.357 (8)
C3—H3	0.9500	C105—H105	0.9500
C4—C5	1.386 (6)	C106—C107	1.408 (9)
C4—H4	0.9500	C106—H106	0.9500
C5—C6	1.393 (5)	C107—C108	1.348 (8)
C5—H5	0.9500	C107—H107	0.9500
C6—C7	1.399 (5)	C108—H108	0.9500
C6—H6	0.9500	N19—C113	1.285 (7)
C8—C9	1.473 (5)	N19—C109	1.305 (7)
C9—C10	1.400 (6)	C109—C110	1.354 (8)
C9—C14	1.413 (5)	C109—H109	0.9500
C10—C11	1.378 (6)	C110—C111	1.480 (9)
C10—H10	0.9500	C110—H110	0.9500
C11—C12	1.390 (7)	C111—C112	1.352 (7)
C11—H11	0.9500	C111—H111	0.9500
C12—C13	1.372 (6)	C112—C113	1.351 (8)
C12—H12	0.9500	C112—H112	0.9500
C13—C14	1.408 (5)	C113—H113	0.9500
C13—H13	0.9500	N20—C118	1.284 (11)
C14—O6	1.332 (5)	N20—C114	1.327 (12)
C8B—C9B	1.477 (15)	C114—C115	1.420 (15)
C9B—C10B	1.404 (17)	C114—H114	0.9500
C9B—C14B	1.419 (16)	C115—C116	1.367 (14)
C10B—C11B	1.378 (17)	C115—H115	0.9500
C10B—H10B	0.9500	C116—C117	1.366 (13)
C11B—C12B	1.387 (18)	C116—H116	0.9500
C11B—H11B	0.9500	C117—C118	1.346 (14)
C12B—C13B	1.375 (17)	C117—H117	0.9500
C12B—H12B	0.9500	C118—H118	0.9500
C13B—C14B	1.408 (16)	N21—C119	1.298 (14)
C13B—H13B	0.9500	N21—C123	1.322 (16)
C14B—O6B	1.346 (15)	C119—C120	1.372 (17)
C15—C16	1.466 (4)	C119—H119	0.9500
C16—C17	1.399 (5)	C120—C121	1.413 (17)
C16—C21	1.411 (4)	C120—H120	0.9500
C17—C18	1.384 (5)	C121—C122	1.353 (16)
C17—H17	0.9500	C121—H121	0.9500
C18—C19	1.386 (5)	C122—C123	1.333 (16)
C18—H18	0.9500	C122—H122	0.9500
C19—C20	1.384 (5)	C123—H123	0.9500
C19—H19	0.9500		
			
O24—Al1—O2	96.07 (10)	C36—N6—Al5	127.4 (2)
O24—Al1—O9	89.33 (10)	O16—N6—Al5	120.3 (2)
O2—Al1—O9	88.93 (10)	C36—N6—Gd1B	126.6 (2)
O24—Al1—O1	170.69 (10)	O16—N6—Gd1B	47.56 (13)
O2—Al1—O1	81.82 (10)	Al5—N6—Gd1B	82.17 (11)
O9—Al1—O1	99.67 (10)	C43—N7—O19	110.6 (2)
O24—Al1—N3	100.54 (11)	C43—N7—Al6	128.0 (2)
O2—Al1—N3	162.06 (11)	O19—N7—Al6	120.14 (18)
O9—Al1—N3	84.54 (10)	C50—N8—O22	110.2 (2)
O1—Al1—N3	82.83 (10)	C50—N8—Al1	125.5 (2)
O24—Al1—N8	85.98 (10)	O22—N8—Al1	123.30 (17)
O2—Al1—N8	95.26 (11)	O2—C1—N1	121.0 (3)
O9—Al1—N8	174.01 (11)	O2—C1—C2	119.1 (3)
O1—Al1—N8	85.19 (9)	N1—C1—C2	120.0 (3)
N3—Al1—N8	92.66 (10)	C3—C2—C7	120.0 (3)
O3—Al2—O17	91.27 (11)	C3—C2—C1	118.3 (3)
O3—Al2—O5	97.48 (12)	C7—C2—C1	121.6 (3)
O17—Al2—O5	94.56 (11)	C4—C3—C2	121.4 (3)
O3—Al2—O4	175.21 (11)	C4—C3—H3	119.3
O17—Al2—O4	93.52 (10)	C2—C3—H3	119.3
O5—Al2—O4	82.32 (10)	C3—C4—C5	118.8 (3)
O3—Al2—O16	102.13 (11)	C3—C4—H4	120.6
O17—Al2—O16	81.64 (10)	C5—C4—H4	120.6
O5—Al2—O16	160.08 (11)	C4—C5—C6	121.0 (4)
O4—Al2—O16	78.43 (10)	C4—C5—H5	119.5
O3—Al2—N1	87.87 (11)	C6—C5—H5	119.5
O17—Al2—N1	169.82 (12)	C5—C6—C7	120.9 (3)
O5—Al2—N1	95.60 (10)	C5—C6—H6	119.5
O4—Al2—N1	87.39 (10)	C7—C6—H6	119.5
O16—Al2—N1	88.63 (10)	O3—C7—C6	119.2 (3)
O3—Al2—Gd1B	133.06 (10)	O3—C7—C2	123.1 (3)
O17—Al2—Gd1B	107.12 (9)	C6—C7—C2	117.7 (3)
O5—Al2—Gd1B	122.65 (9)	O5—C8—N2	120.6 (3)
O4—Al2—Gd1B	44.91 (7)	O5—C8—C9	120.1 (3)
O16—Al2—Gd1B	42.25 (8)	N2—C8—C9	119.2 (3)
N1—Al2—Gd1B	66.70 (8)	C10—C9—C14	119.4 (4)
O3—Al2—Gd1	134.65 (9)	C10—C9—C8	118.0 (4)
O17—Al2—Gd1	109.83 (8)	C14—C9—C8	122.6 (4)
O5—Al2—Gd1	119.24 (8)	C11—C10—C9	121.5 (4)
O4—Al2—Gd1	42.97 (6)	C11—C10—H10	119.2
O16—Al2—Gd1	46.03 (7)	C9—C10—H10	119.2
N1—Al2—Gd1	64.43 (8)	C10—C11—C12	119.2 (4)
O6B—Al3—O26B	103.3 (5)	C10—C11—H11	120.4
O6—Al3—O26	93.52 (13)	C12—C11—H11	120.4
O6—Al3—O25	176.19 (16)	C13—C12—C11	120.4 (4)
O26—Al3—O25	84.57 (15)	C13—C12—H12	119.8
O6B—Al3—N2	91.34 (11)	C11—C12—H12	119.8
O6—Al3—N2	91.34 (11)	C12—C13—C14	121.7 (4)
O26B—Al3—N2	164.8 (6)	C12—C13—H13	119.2
O26—Al3—N2	174.92 (14)	C14—C13—H13	119.2
O25—Al3—N2	90.50 (14)	O6—C14—C13	118.3 (4)
O6B—Al3—O28	90.16 (11)	O6—C14—C9	123.9 (4)
O6—Al3—O28	90.16 (11)	C13—C14—C9	117.8 (4)
O26B—Al3—O28	95.9 (10)	C14—O6—Al3	128.1 (3)
O26—Al3—O28	90.36 (17)	O5—C8B—N2	120.6 (3)
O25—Al3—O28	86.56 (15)	O5—C8B—C9B	120.3 (9)
N2—Al3—O28	88.12 (11)	N2—C8B—C9B	118.9 (10)
O6B—Al3—O25B	174.4 (6)	C10B—C9B—C14B	119.4 (15)
O26B—Al3—O25B	81.2 (7)	C10B—C9B—C8B	117.8 (15)
N2—Al3—O25B	84.0 (5)	C14B—C9B—C8B	122.7 (16)
O28—Al3—O25B	92.8 (9)	C11B—C10B—C9B	122.2 (19)
O6B—Al3—N10	91.73 (12)	C11B—C10B—H10B	118.9
O6—Al3—N10	91.73 (12)	C9B—C10B—H10B	118.9
O26B—Al3—N10	84.8 (10)	C10B—C11B—C12B	118.6 (18)
O26—Al3—N10	90.61 (18)	C10B—C11B—H11B	120.7
O25—Al3—N10	91.59 (16)	C12B—C11B—H11B	120.7
N2—Al3—N10	90.74 (14)	C13B—C12B—C11B	120.4 (18)
O28—Al3—N10	177.82 (12)	C13B—C12B—H12B	119.8
O25B—Al3—N10	85.3 (9)	C11B—C12B—H12B	119.8
O18—Al5—O20	93.64 (13)	C12B—C13B—C14B	122.6 (17)
O18—Al5—O19	175.11 (14)	C12B—C13B—H13B	118.7
O20—Al5—O19	82.27 (10)	C14B—C13B—H13B	118.7
O18—Al5—O13	101.06 (12)	O6B—C14B—C13B	120.3 (15)
O20—Al5—O13	164.87 (13)	O6B—C14B—C9B	122.8 (16)
O19—Al5—O13	83.20 (10)	C13B—C14B—C9B	116.8 (14)
O18—Al5—O14	90.13 (13)	C14B—O6B—Al3	124.6 (14)
O20—Al5—O14	94.82 (13)	O8—C15—N3	120.7 (3)
O19—Al5—O14	92.89 (12)	O8—C15—C16	120.3 (3)
O13—Al5—O14	81.69 (12)	N3—C15—C16	118.9 (3)
O18—Al5—N6	89.81 (13)	C17—C16—C21	119.9 (3)
O20—Al5—N6	99.48 (13)	C17—C16—C15	118.8 (3)
O19—Al5—N6	88.22 (11)	C21—C16—C15	121.2 (3)
O13—Al5—N6	84.26 (11)	C18—C17—C16	121.4 (3)
O14—Al5—N6	165.68 (13)	C18—C17—H17	119.3
O18—Al5—Gd1B	136.17 (10)	C16—C17—H17	119.3
O20—Al5—Gd1B	119.77 (9)	C17—C18—C19	118.7 (3)
O19—Al5—Gd1B	45.56 (7)	C17—C18—H18	120.6
O13—Al5—Gd1B	49.90 (8)	C19—C18—H18	120.6
O14—Al5—Gd1B	112.35 (10)	C20—C19—C18	120.8 (3)
N6—Al5—Gd1B	59.30 (9)	C20—C19—H19	119.6
O18—Al5—Gd1	138.88 (9)	C18—C19—H19	119.6
O20—Al5—Gd1	121.42 (8)	C19—C20—C21	121.5 (3)
O19—Al5—Gd1	43.51 (7)	C19—C20—H20	119.3
O13—Al5—Gd1	46.93 (7)	C21—C20—H20	119.3
O14—Al5—Gd1	106.05 (9)	O9—C21—C20	119.7 (3)
N6—Al5—Gd1	65.52 (8)	O9—C21—C16	122.7 (3)
O21—Al6—O23	93.71 (10)	C20—C21—C16	117.6 (3)
O21—Al6—O22	174.08 (12)	O14—C29—N5	117.2 (4)
O23—Al6—O22	83.50 (10)	O14—C29—C30	121.7 (4)
O21—Al6—N7	92.17 (11)	N5—C29—C30	121.0 (4)
O23—Al6—N7	174.09 (11)	C31—C30—C35	118.9 (5)
O22—Al6—N7	90.69 (10)	C31—C30—C29	117.7 (4)
O21—Al6—O12B	93.0 (5)	C35—C30—C29	123.4 (4)
O23—Al6—O12B	106.3 (5)	C30—C31—C32	120.3 (6)
O22—Al6—O12B	92.8 (5)	C30—C31—H31	119.8
N7—Al6—O12B	72.7 (5)	C32—C31—H31	119.8
O21—Al6—O29	87.38 (12)	C33—C32—C31	119.7 (6)
O23—Al6—O29	93.34 (12)	C33—C32—H32	120.1
O22—Al6—O29	87.57 (11)	C31—C32—H32	120.1
N7—Al6—O29	87.50 (12)	C34—C33—C32	120.9 (6)
O12B—Al6—O29	160.2 (5)	C34—C33—H33	119.5
O21—Al6—N11	89.17 (12)	C32—C33—H33	119.5
O23—Al6—N11	88.08 (13)	C33—C34—C35	120.0 (6)
O22—Al6—N11	95.95 (12)	C33—C34—H34	120.0
N7—Al6—N11	91.43 (14)	C35—C34—H34	120.0
O29—Al6—N11	176.34 (13)	O15—C35—C34	121.1 (5)
N1—O1—Al1	112.32 (16)	O15—C35—C30	118.7 (4)
N1—O1—Gd1B	99.49 (15)	C34—C35—C30	120.2 (5)
Al1—O1—Gd1B	125.27 (11)	O17—C36—N6	121.8 (3)
N1—O1—Gd1	109.53 (14)	O17—C36—C37	117.6 (3)
Al1—O1—Gd1	118.91 (10)	N6—C36—C37	120.6 (3)
C1—O2—Al1	112.91 (19)	C38—C37—C42	120.6 (3)
C7—O3—Al2	131.5 (2)	C38—C37—C36	117.4 (4)
N2—O4—Al2	112.23 (17)	C42—C37—C36	122.0 (4)
N2—O4—Gd1B	134.97 (18)	C39—C38—C37	121.1 (4)
Al2—O4—Gd1B	97.94 (10)	C39—C38—H38	119.4
N2—O4—Gd1	126.62 (16)	C37—C38—H38	119.4
Al2—O4—Gd1	105.14 (9)	C40—C39—C38	118.5 (5)
C8B—O5—Al2	112.05 (19)	C40—C39—H39	120.7
C8—O5—Al2	112.05 (19)	C38—C39—H39	120.7
N3—O7—Al4B	126.1 (4)	C41—C40—C39	121.2 (4)
N3—O7—Al4	111.95 (17)	C41—C40—H40	119.4
N3—O7—Gd1	118.86 (16)	C39—C40—H40	119.4
Al4—O7—Gd1	109.34 (10)	C40—C41—C42	122.1 (4)
N3—O7—Gd1B	115.55 (16)	C40—C41—H41	119.0
Al4B—O7—Gd1B	104.9 (3)	C42—C41—H41	119.0
C15—O8—Al4	112.9 (2)	O18—C42—C41	119.4 (4)
C15—O8—Al4B	104.1 (3)	O18—C42—C37	124.1 (3)
C21—O9—Al1	126.50 (18)	C41—C42—C37	116.5 (4)
O7—Gd1—O1	73.36 (7)	O20—C43—N7	120.5 (3)
O7—Gd1—O22	73.90 (8)	O20—C43—C44	118.9 (3)
O1—Gd1—O22	73.27 (7)	N7—C43—C44	120.6 (3)
O7—Gd1—O25	75.40 (10)	C45—C44—C49	119.9 (3)
O1—Gd1—O25	85.73 (12)	C45—C44—C43	117.9 (3)
O22—Gd1—O25	146.64 (11)	C49—C44—C43	122.2 (3)
O7—Gd1—O19	116.31 (8)	C46—C45—C44	121.3 (4)
O1—Gd1—O19	136.36 (7)	C46—C45—H45	119.3
O22—Gd1—O19	69.83 (7)	C44—C45—H45	119.3
O25—Gd1—O19	137.48 (12)	C45—C46—C47	119.0 (4)
O7—Gd1—O4	128.23 (7)	C45—C46—H46	120.5
O1—Gd1—O4	68.79 (7)	C47—C46—H46	120.5
O22—Gd1—O4	124.22 (8)	C48—C47—C46	120.7 (4)
O25—Gd1—O4	67.92 (10)	C48—C47—H47	119.6
O19—Gd1—O4	115.44 (8)	C46—C47—H47	119.6
O7—Gd1—O16	145.99 (7)	C47—C48—C49	121.7 (4)
O1—Gd1—O16	81.59 (7)	C47—C48—H48	119.1
O22—Gd1—O16	77.13 (8)	C49—C48—H48	119.1
O25—Gd1—O16	125.87 (10)	O21—C49—C48	118.6 (3)
O19—Gd1—O16	68.30 (8)	O21—C49—C44	124.1 (3)
O4—Gd1—O16	58.39 (7)	C48—C49—C44	117.3 (3)
O7—Gd1—O13	133.20 (8)	O23—C50—N8	120.9 (3)
O1—Gd1—O13	144.13 (8)	O23—C50—C51	118.5 (3)
O22—Gd1—O13	130.84 (7)	N8—C50—C51	120.6 (3)
O25—Gd1—O13	80.69 (12)	C52—C51—C56	120.1 (3)
O19—Gd1—O13	61.37 (7)	C52—C51—C50	117.6 (3)
O4—Gd1—O13	75.34 (8)	C56—C51—C50	122.0 (3)
O16—Gd1—O13	79.91 (8)	C53—C52—C51	121.5 (3)
O7—Gd1—O10	58.83 (8)	C53—C52—H52	119.3
O1—Gd1—O10	129.95 (8)	C51—C52—H52	119.3
O22—Gd1—O10	104.41 (9)	C52—C53—C54	118.8 (3)
O25—Gd1—O10	69.59 (11)	C52—C53—H53	120.6
O19—Gd1—O10	82.09 (8)	C54—C53—H53	120.6
O4—Gd1—O10	131.26 (8)	C55—C54—C53	120.3 (3)
O16—Gd1—O10	148.01 (8)	C55—C54—H54	119.8
O13—Gd1—O10	75.31 (9)	C53—C54—H54	119.8
O7—Gd1—Al5	142.96 (5)	C54—C55—C56	122.0 (3)
O1—Gd1—Al5	140.24 (6)	C54—C55—H55	119.0
O22—Gd1—Al5	98.36 (5)	C56—C55—H55	119.0
O25—Gd1—Al5	114.07 (10)	O24—C56—C51	123.6 (3)
O19—Gd1—Al5	32.68 (5)	O24—C56—C55	119.2 (3)
O4—Gd1—Al5	86.53 (5)	C51—C56—C55	117.2 (3)
O16—Gd1—Al5	58.75 (5)	C68—N10—C64	117.5 (4)
O13—Gd1—Al5	33.46 (6)	C68—N10—Al3	122.9 (3)
O10—Gd1—Al5	89.79 (6)	C64—N10—Al3	119.6 (3)
O7—Gd1—Al2	131.11 (5)	N10—C64—C65	121.9 (6)
O1—Gd1—Al2	57.80 (5)	N10—C64—H64	119.1
O22—Gd1—Al2	92.90 (6)	C65—C64—H64	119.1
O25—Gd1—Al2	97.53 (9)	C66—C65—C64	119.0 (6)
O19—Gd1—Al2	101.50 (6)	C66—C65—H65	120.5
O4—Gd1—Al2	31.90 (5)	C64—C65—H65	120.5
O16—Gd1—Al2	33.58 (5)	C65—C66—C67	120.0 (5)
O13—Gd1—Al2	91.19 (6)	C65—C66—H66	120.0
O10—Gd1—Al2	162.43 (6)	C67—C66—H66	120.0
Al5—Gd1—Al2	84.65 (3)	C66—C67—C68	117.9 (7)
O7—Gd1—Al4	31.28 (6)	C66—C67—H67	121.1
O1—Gd1—Al4	96.67 (6)	C68—C67—H67	121.1
O22—Gd1—Al4	100.33 (7)	N10—C68—C67	123.9 (6)
O25—Gd1—Al4	55.92 (8)	N10—C68—H68	118.1
O19—Gd1—Al4	112.00 (6)	C67—C68—H68	118.1
O4—Gd1—Al4	123.02 (5)	C74—N12—C78	122.0 (4)
O16—Gd1—Al4	177.24 (5)	C74—N12—H12A	119.0
O13—Gd1—Al4	102.68 (7)	C78—N12—H12A	119.0
O10—Gd1—Al4	33.46 (6)	N12—C74—C75	121.0 (4)
Al5—Gd1—Al4	123.07 (3)	N12—C74—H74	119.5
Al2—Gd1—Al4	146.43 (3)	C75—C74—H74	119.5
O8—Al4—O27	92.32 (12)	C74—C75—C76	118.9 (5)
O8—Al4—O11	100.14 (12)	C74—C75—H75	120.5
O27—Al4—O11	86.45 (13)	C76—C75—H75	120.5
O8—Al4—O7	81.85 (11)	C75—C76—C77	118.8 (5)
O27—Al4—O7	173.99 (13)	C75—C76—H76	120.6
O11—Al4—O7	95.99 (12)	C77—C76—H76	120.6
O8—Al4—O10	160.27 (14)	C78—C77—C76	119.8 (5)
O27—Al4—O10	107.39 (13)	C78—C77—H77	120.1
O11—Al4—O10	82.37 (12)	C76—C77—H77	120.1
O7—Al4—O10	78.43 (11)	N12—C78—C77	119.3 (4)
O8—Al4—N9	96.80 (13)	N12—C78—H78	120.3
O27—Al4—N9	86.24 (15)	C77—C78—H78	120.3
O11—Al4—N9	161.79 (15)	C83—N13—C79	118.4 (4)
O7—Al4—N9	92.97 (14)	N13—C79—C80	124.1 (4)
O10—Al4—N9	83.94 (13)	N13—C79—H79	118.0
O8—Al4—Gd1	114.36 (9)	C80—C79—H79	118.0
O27—Al4—Gd1	144.40 (10)	C81—C80—C79	117.0 (4)
O11—Al4—Gd1	109.92 (10)	C81—C80—H80	121.5
O7—Al4—Gd1	39.39 (7)	C79—C80—H80	121.5
O10—Al4—Gd1	47.63 (9)	C80—C81—C82	120.0 (4)
N9—Al4—Gd1	68.33 (11)	C80—C81—H81	120.0
N4—O10—Al4	109.0 (2)	C82—C81—H81	120.0
N4—O10—Gd1	117.8 (2)	C81—C82—C83	118.6 (5)
Al4—O10—Gd1	98.91 (11)	C81—C82—H82	120.7
C22—N4—O10	117.0 (3)	C83—C82—H82	120.7
C22—N4—H4N	121.5	N13—C83—C82	121.9 (4)
O10—N4—H4N	121.5	N13—C83—H83	119.1
C22—O11—Al4	114.0 (2)	C82—C83—H83	119.1
O11—C22—N4	117.4 (3)	C88—N14—C84	118.5 (11)
O11—C22—C23	120.4 (3)	N14—C84—C85	121.3 (11)
N4—C22—C23	122.0 (3)	N14—C84—H84	119.3
C24—C23—C28	120.2 (4)	C85—C84—H84	119.3
C24—C23—C22	117.7 (3)	C86—C85—C84	119.7 (11)
C28—C23—C22	122.0 (4)	C86—C85—H85	120.1
C23—C24—C25	120.8 (4)	C84—C85—H85	120.1
C23—C24—H24	119.6	C85—C86—C87	119.9 (11)
C25—C24—H24	119.6	C85—C86—H86	120.0
C26—C25—C24	119.1 (5)	C87—C86—H86	120.0
C26—C25—H25	120.5	C86—C87—C88	117.5 (10)
C24—C25—H25	120.5	C86—C87—H87	121.2
C27—C26—C25	121.1 (4)	C88—C87—H87	121.2
C27—C26—H26	119.4	N14—C88—C87	122.7 (11)
C25—C26—H26	119.4	N14—C88—H88	118.6
C26—C27—C28	120.8 (5)	C87—C88—H88	118.6
C26—C27—H27	119.6	C128—N22—C124	117.4 (16)
C28—C27—H27	119.6	N22—C124—C125	123.5 (17)
O12—C28—C23	119.0 (4)	N22—C124—H124	118.2
O12—C28—C27	123.1 (4)	C125—C124—H124	118.2
C23—C28—C27	118.0 (5)	C126—C125—C124	117.9 (15)
C28—O12—H12O	109.5	C126—C125—H125	121.0
N9—O25—Al3	109.9 (2)	C124—C125—H125	121.0
N9—O25—Gd1	117.7 (2)	C125—C126—C127	120.2 (15)
Al3—O25—Gd1	130.76 (19)	C125—C126—H126	119.9
C57—N9—O25	112.4 (3)	C127—C126—H126	119.9
C57—N9—Al4	129.3 (3)	C128—C127—C126	116.8 (14)
O25—N9—Al4	117.6 (3)	C128—C127—H127	121.6
C57—O26—Al3	110.7 (3)	C126—C127—H127	121.6
N9—C57—O26	120.5 (4)	N22—C128—C127	122.3 (16)
N9—C57—C58	119.9 (3)	N22—C128—H128	118.9
O26—C57—C58	119.6 (4)	C127—C128—H128	118.9
C59—C58—C63	119.7 (4)	C89—N15—C133	119.5 (11)
C59—C58—C57	118.4 (4)	C133—N15—C129	117.1 (12)
C63—C58—C57	121.8 (4)	C89—N15—C93	117.5 (9)
C60—C59—C58	120.6 (4)	C129—N15—C93	112.0 (12)
C60—C59—H59	119.7	N23—C89—C90	127.8 (12)
C58—C59—H59	119.7	N15—C89—C90	127.8 (12)
C59—C60—C61	119.5 (4)	N23—C89—C130	119.8 (14)
C59—C60—H60	120.2	N15—C89—C130	119.8 (14)
C61—C60—H60	120.2	N23—C89—H89	116.1
C62—C61—C60	120.4 (4)	N15—C89—H89	116.1
C62—C61—H61	119.8	C90—C89—H89	116.1
C60—C61—H61	119.8	C130—C89—H89	124.0
C61—C62—C63	120.8 (4)	C129—C90—C91	109.8 (14)
C61—C62—H62	119.6	C89—C90—C91	114.5 (11)
C63—C62—H62	119.6	C129—C90—C131	107.9 (12)
O27—C63—C62	118.6 (4)	C89—C90—C131	107.9 (12)
O27—C63—C58	122.5 (3)	C129—C90—H90	126.6
C62—C63—C58	118.9 (4)	C89—C90—H90	122.8
C63—O27—Al4	126.9 (2)	C91—C90—H90	122.8
C69—N11—C73	117.3 (4)	C131—C90—H90	122.9
C69—N11—Al6	119.8 (3)	C131—C91—C130	91 (3)
C73—N11—Al6	122.0 (3)	C131—C91—C90	94 (3)
N11—C69—C70	122.9 (4)	C131—C91—C92	79 (2)
N11—C69—H69	118.6	C130—C91—C92	119.8 (14)
C70—C69—H69	118.6	C90—C91—C92	120.4 (10)
C71—C70—C69	119.5 (4)	C131—C91—C132	50.1 (17)
C71—C70—H70	120.2	C130—C91—C132	109.1 (18)
C69—C70—H70	120.2	C90—C91—C132	111.0 (12)
C70—C71—C72	118.4 (4)	C131—C91—H91	97.3
C70—C71—H71	120.8	C130—C91—H91	120.3
C72—C71—H71	120.8	C90—C91—H91	119.8
C71—C72—C73	118.9 (4)	C92—C91—H91	119.8
C71—C72—H72	120.6	C132—C91—H91	121.1
C73—C72—H72	120.6	C132—C92—C93	74.1 (15)
N11—C73—C72	122.9 (4)	C132—C92—C91	96.8 (17)
N11—C73—H73	118.6	C93—C92—C91	117.5 (11)
C72—C73—H73	118.6	C132—C92—C131	68.6 (16)
O16—Gd1B—O4	68.13 (10)	C93—C92—C131	111.5 (14)
O16—Gd1B—O19	77.37 (10)	C132—C92—C133	61.0 (13)
O4—Gd1B—O19	129.98 (12)	C91—C92—C133	115.3 (11)
O16—Gd1B—O1	91.46 (10)	C131—C92—C133	103.2 (14)
O4—Gd1B—O1	73.07 (9)	C132—C92—H92	98.6
O19—Gd1B—O1	144.37 (11)	C93—C92—H92	121.2
O16—Gd1B—O25B	132.6 (5)	C91—C92—H92	121.2
O4—Gd1B—O25B	65.8 (5)	C131—C92—H92	119.6
O19—Gd1B—O25B	126.6 (7)	C133—C92—H92	121.8
O1—Gd1B—O25B	85.7 (8)	N23—C93—C92	121.6 (10)
O16—Gd1B—O22	85.86 (11)	N15—C93—C92	121.6 (10)
O4—Gd1B—O22	136.84 (12)	N23—C93—C132	120.2 (14)
O19—Gd1B—O22	71.68 (9)	N15—C93—C132	120.2 (14)
O1—Gd1B—O22	73.86 (9)	N23—C93—H93	119.2
O25B—Gd1B—O22	137.3 (6)	N15—C93—H93	119.2
O16—Gd1B—O13	89.98 (11)	C92—C93—H93	119.2
O4—Gd1B—O13	80.82 (10)	C132—C93—H93	108.3
O19—Gd1B—O13	63.66 (9)	C89—N23—C133	119.5 (11)
O1—Gd1B—O13	151.16 (11)	C133—N23—C129	117.1 (12)
O25B—Gd1B—O13	72.4 (8)	C89—N23—C93	117.5 (9)
O22—Gd1B—O13	134.95 (11)	C129—N23—C93	112.0 (12)
O16—Gd1B—O10B	143.4 (3)	C90—C129—N23	134.7 (19)
O4—Gd1B—O10B	132.0 (3)	C90—C129—N15	134.7 (19)
O19—Gd1B—O10B	66.7 (3)	N23—C129—C130	125.9 (16)
O1—Gd1B—O10B	121.9 (3)	N15—C129—C130	125.9 (16)
O25B—Gd1B—O10B	70.1 (6)	C90—C129—H129	108.2
O22—Gd1B—O10B	89.3 (3)	N23—C129—H129	117.1
O13—Gd1B—O10B	68.4 (3)	N15—C129—H129	117.1
O16—Gd1B—O7	149.35 (11)	C130—C129—H129	117.1
O4—Gd1B—O7	121.52 (10)	C91—C130—C129	118 (2)
O19—Gd1B—O7	106.75 (10)	C129—C130—C131	116.3 (16)
O1—Gd1B—O7	67.22 (8)	C91—C130—C89	122.6 (18)
O25B—Gd1B—O7	70.0 (6)	C131—C130—C89	115.3 (16)
O22—Gd1B—O7	67.62 (9)	C91—C130—H130	111.3
O13—Gd1B—O7	119.46 (11)	C129—C130—H130	121.8
O10B—Gd1B—O7	55.1 (3)	C131—C130—H130	121.8
O16—Gd1B—N6	30.18 (8)	C89—C130—H130	122.0
O4—Gd1B—N6	68.31 (9)	C91—C131—C130	58 (3)
O19—Gd1B—N6	63.76 (10)	C91—C131—C132	107 (3)
O1—Gd1B—N6	118.58 (11)	C130—C131—C132	119.4 (13)
O25B—Gd1B—N6	117.2 (6)	C91—C131—C92	72 (2)
O22—Gd1B—N6	105.49 (10)	C130—C131—C92	104.3 (19)
O13—Gd1B—N6	59.88 (9)	C91—C131—C90	60 (2)
O10B—Gd1B—N6	119.5 (3)	C132—C131—C90	119.3 (14)
O7—Gd1B—N6	170.17 (10)	C92—C131—C90	105.3 (15)
O16—Gd1B—N1	63.44 (9)	C91—C131—H131	103.7
O4—Gd1B—N1	60.87 (9)	C130—C131—H131	120.3
O19—Gd1B—N1	130.94 (11)	C132—C131—H131	120.3
O1—Gd1B—N1	28.26 (7)	C92—C131—H131	124.5
O25B—Gd1B—N1	102.0 (7)	C90—C131—H131	120.4
O22—Gd1B—N1	76.96 (9)	C92—C132—C133	85.9 (19)
O13—Gd1B—N1	138.89 (11)	C92—C132—C131	76.5 (18)
O10B—Gd1B—N1	149.6 (3)	C133—C132—C131	117.7 (14)
O7—Gd1B—N1	94.57 (8)	C92—C132—C93	69.9 (15)
N6—Gd1B—N1	90.49 (10)	C131—C132—C93	113.2 (13)
O16—Gd1B—Al2	38.15 (7)	C92—C132—C91	53.9 (12)
O4—Gd1B—Al2	37.15 (7)	C133—C132—C91	107.5 (13)
O19—Gd1B—Al2	115.10 (10)	C93—C132—C91	97.9 (12)
O1—Gd1B—Al2	64.27 (7)	C92—C132—H132	107.2
O25B—Gd1B—Al2	101.5 (5)	C133—C132—H132	121.1
O22—Gd1B—Al2	102.52 (9)	C131—C132—H132	121.1
O13—Gd1B—Al2	101.31 (9)	C93—C132—H132	123.2
O10B—Gd1B—Al2	168.0 (3)	C91—C132—H132	126.8
O7—Gd1B—Al2	131.26 (8)	N23—C133—C132	122.9 (14)
N6—Gd1B—Al2	55.90 (7)	N15—C133—C132	122.9 (14)
N1—Gd1B—Al2	38.57 (6)	N23—C133—C92	108.9 (12)
O7—Al4B—O27B	162.9 (8)	N15—C133—C92	108.9 (12)
O7—Al4B—O11B	94.8 (6)	N23—C133—H133	118.5
O27B—Al4B—O11B	88.6 (7)	N15—C133—H133	118.5
O7—Al4B—O10B	87.4 (6)	C132—C133—H133	118.5
O27B—Al4B—O10B	109.7 (8)	C92—C133—H133	122.5
O11B—Al4B—O10B	82.9 (6)	C139—N25—C143	120.0
O7—Al4B—N9B	93.6 (7)	N25—C139—C140	120.0
O27B—Al4B—N9B	86.7 (7)	N25—C139—H139	120.0
O11B—Al4B—N9B	165.8 (10)	C140—C139—H139	120.0
O10B—Al4B—N9B	86.0 (7)	C141—C140—C139	120.0
O7—Al4B—O8	77.9 (4)	C141—C140—H140	120.0
O27B—Al4B—O8	85.1 (6)	C139—C140—H140	120.0
O11B—Al4B—O8	101.4 (6)	C140—C141—C142	120.0
O10B—Al4B—O8	164.9 (6)	C140—C141—H141	120.0
N9B—Al4B—O8	91.6 (8)	C142—C141—H141	120.0
O7—Al4B—Gd1B	48.3 (2)	C141—C142—C143	120.0
O27B—Al4B—Gd1B	144.9 (6)	C141—C142—H142	120.0
O11B—Al4B—Gd1B	110.3 (5)	C143—C142—H142	120.0
O10B—Al4B—Gd1B	47.7 (4)	C142—C143—N25	120.0
N9B—Al4B—Gd1B	67.6 (5)	C142—C143—H143	120.0
O8—Al4B—Gd1B	117.9 (3)	N25—C143—H143	120.0
N4B—O10B—Al4B	105.3 (10)	C98—N16—C94	122.1 (4)
N4B—O10B—Gd1B	103.8 (10)	C98—N16—H16	118.9
Al4B—O10B—Gd1B	99.7 (6)	C94—N16—H16	118.9
C22B—N4B—O10B	118.5 (14)	C95—C94—N16	120.0 (4)
C22B—N4B—H4B	109 (7)	C95—C94—H94	120.0
O10B—N4B—H4B	128 (10)	N16—C94—H94	120.0
C22B—O11B—Al4B	109.3 (11)	C94—C95—C96	118.9 (4)
O11B—C22B—N4B	114.7 (13)	C94—C95—H95	120.6
O11B—C22B—C23B	124.8 (14)	C96—C95—H95	120.6
N4B—C22B—C23B	120.0 (14)	C97—C96—C95	119.9 (5)
C24B—C23B—C28B	119.9 (14)	C97—C96—H96	120.0
C24B—C23B—C22B	115.9 (14)	C95—C96—H96	120.0
C28B—C23B—C22B	123.9 (14)	C98—C97—C96	119.0 (5)
C25B—C24B—C23B	120.2 (17)	C98—C97—H97	120.5
C25B—C24B—H24B	119.9	C96—C97—H97	120.5
C23B—C24B—H24B	119.9	N16—C98—C97	120.1 (5)
C26B—C25B—C24B	118.9 (18)	N16—C98—H98	120.0
C26B—C25B—H25B	120.5	C97—C98—H98	120.0
C24B—C25B—H25B	120.5	C99—N17—C103	119.7 (9)
C27B—C26B—C25B	122.6 (18)	N17—C99—C100	121.2 (10)
C27B—C26B—H26B	118.7	N17—C99—H99	119.4
C25B—C26B—H26B	118.7	C100—C99—H99	119.4
C26B—C27B—C28B	119.0 (17)	C101—C100—C99	118.8 (11)
C26B—C27B—H27B	120.5	C101—C100—H100	120.6
C28B—C27B—H27B	120.5	C99—C100—H100	120.6
O12B—C28B—C23B	118.8 (15)	C100—C101—C102	114.9 (11)
O12B—C28B—C27B	122.9 (16)	C100—C101—H101	122.6
C23B—C28B—C27B	118.3 (14)	C102—C101—H101	122.6
C28B—O12B—Al6	133.4 (17)	C103—C102—C101	117.4 (10)
C28B—O12B—H12P	109.5	C103—C102—H102	121.3
Al6—O12B—H12P	109.5	C101—C102—H102	121.3
N9B—O25B—Al3	106.8 (10)	N17—C103—C102	121.1 (9)
N9B—O25B—Gd1B	118.4 (11)	N17—C103—H103	119.4
Al3—O25B—Gd1B	129.7 (10)	C102—C103—H103	119.4
C57B—N9B—O25B	112.7 (13)	C134—N24—C138	116.3 (14)
C57B—N9B—Al4B	128.2 (13)	N24—C134—C135	121.9 (15)
O25B—N9B—Al4B	118.2 (12)	N24—C134—H134	119.0
C57B—O26B—Al3	113.7 (16)	C135—C134—H134	119.0
N9B—C57B—O26B	119.9 (16)	C136—C135—C134	113.6 (15)
N9B—C57B—C58B	120.7 (15)	C136—C135—H135	123.2
O26B—C57B—C58B	119.2 (17)	C134—C135—H135	123.2
C59B—C58B—C63B	121.6 (15)	C135—C136—C137	110.5 (16)
C59B—C58B—C57B	117.7 (14)	C135—C136—H136	124.8
C63B—C58B—C57B	120.7 (14)	C137—C136—H136	124.8
C58B—C59B—C60B	120.4 (17)	C138—C137—C136	110.0 (15)
C58B—C59B—H59B	119.8	C138—C137—H137	125.0
C60B—C59B—H59B	119.8	C136—C137—H137	125.0
C61B—C60B—C59B	117.0 (18)	N24—C138—C137	122.8 (14)
C61B—C60B—H60B	121.5	N24—C138—H138	118.6
C59B—C60B—H60B	121.5	C137—C138—H138	118.6
C60B—C61B—C62B	122.6 (18)	C108—N18—C104	114.2 (5)
C60B—C61B—H61B	118.7	C105—C104—N18	124.9 (6)
C62B—C61B—H61B	118.7	C105—C104—H104	117.6
C61B—C62B—C63B	121.4 (17)	N18—C104—H104	117.6
C61B—C62B—H62B	119.3	C104—C105—C106	119.0 (6)
C63B—C62B—H62B	119.3	C104—C105—H105	120.5
O27B—C63B—C62B	119.7 (16)	C106—C105—H105	120.5
O27B—C63B—C58B	123.2 (15)	C105—C106—C107	118.1 (5)
C62B—C63B—C58B	116.9 (15)	C105—C106—H106	120.9
C63B—O27B—Al4B	125.5 (13)	C107—C106—H106	120.9
N5—O13—Al5	110.2 (2)	C108—C107—C106	116.5 (6)
N5—O13—Gd1B	127.5 (2)	C108—C107—H107	121.7
Al5—O13—Gd1B	93.77 (10)	C106—C107—H107	121.7
N5—O13—Gd1	118.2 (2)	N18—C108—C107	127.2 (7)
Al5—O13—Gd1	99.61 (9)	N18—C108—H108	116.4
C29—O14—Al5	113.4 (2)	C107—C108—H108	116.4
C35—O15—H15	109.5	C113—N19—C109	116.0 (5)
N6—O16—Al2	109.55 (17)	N19—C109—C110	127.0 (5)
N6—O16—Gd1B	102.26 (18)	N19—C109—H109	116.5
Al2—O16—Gd1B	99.60 (11)	C110—C109—H109	116.5
N6—O16—Gd1	102.87 (17)	C109—C110—C111	115.1 (5)
Al2—O16—Gd1	100.39 (9)	C109—C110—H110	122.4
C36—O17—Al2	111.5 (2)	C111—C110—H110	122.4
C42—O18—Al5	133.5 (2)	C112—C111—C110	115.8 (5)
N7—O19—Al5	110.17 (18)	C112—C111—H111	122.1
N7—O19—Gd1B	120.47 (17)	C110—C111—H111	122.1
Al5—O19—Gd1B	98.75 (10)	C113—C112—C111	120.5 (5)
N7—O19—Gd1	124.67 (16)	C113—C112—H112	119.8
Al5—O19—Gd1	103.82 (9)	C111—C112—H112	119.8
C43—O20—Al5	111.5 (2)	N19—C113—C112	125.5 (6)
C49—O21—Al6	129.4 (2)	N19—C113—H113	117.3
N8—O22—Al6	112.02 (16)	C112—C113—H113	117.3
N8—O22—Gd1B	120.76 (16)	C118—N20—C114	118.2 (10)
Al6—O22—Gd1B	123.66 (11)	N20—C114—C115	122.3 (10)
N8—O22—Gd1	117.71 (15)	N20—C114—H114	118.8
Al6—O22—Gd1	129.40 (10)	C115—C114—H114	118.8
C50—O23—Al6	113.16 (19)	C116—C115—C114	117.2 (9)
C56—O24—Al1	130.7 (2)	C116—C115—H115	121.4
Al3—O28—H28A	117 (3)	C114—C115—H115	121.4
Al3—O28—H28B	116 (3)	C117—C116—C115	117.7 (10)
H28A—O28—H28B	117 (4)	C117—C116—H116	121.1
Al6—O29—H29A	104 (3)	C115—C116—H116	121.1
Al6—O29—H29B	130 (3)	C118—C117—C116	121.0 (10)
H29A—O29—H29B	116 (5)	C118—C117—H117	119.5
H30A—O30—H30B	119 (10)	C116—C117—H117	119.5
C1—N1—O1	110.2 (2)	N20—C118—C117	123.4 (10)
C1—N1—Al2	130.0 (2)	N20—C118—H118	118.3
O1—N1—Al2	119.76 (17)	C117—C118—H118	118.3
C1—N1—Gd1B	145.7 (2)	C119—N21—C123	118.2 (15)
O1—N1—Gd1B	52.25 (12)	N21—C119—C120	121.1 (15)
Al2—N1—Gd1B	74.73 (9)	N21—C119—H119	119.4
C8B—N2—O4	111.7 (3)	C120—C119—H119	119.4
C8—N2—O4	111.7 (3)	C119—C120—C121	119.8 (14)
C8B—N2—Al3	129.3 (2)	C119—C120—H120	120.1
C8—N2—Al3	129.3 (2)	C122—C121—C120	115.1 (14)
O4—N2—Al3	118.19 (18)	C122—C121—H121	122.4
C15—N3—O7	110.2 (2)	C120—C121—H121	122.4
C15—N3—Al1	132.6 (2)	C123—C122—C121	120.7 (13)
O7—N3—Al1	117.26 (18)	C123—C122—H122	119.7
C29—N5—O13	117.3 (3)	C121—C122—H122	119.7
C29—N5—H5N	121.4	N21—C123—C122	123.2 (13)
O13—N5—H5N	121.4	N21—C123—H123	118.4
C36—N6—O16	109.8 (3)	C122—C123—H123	118.4
			
O24—Al1—O2—C1	160.5 (2)	Gd1B—N6—C36—O17	50.8 (4)
O9—Al1—O2—C1	−110.3 (2)	O16—N6—C36—C37	177.6 (3)
O1—Al1—O2—C1	−10.4 (2)	Al5—N6—C36—C37	−20.4 (5)
N3—Al1—O2—C1	−41.8 (4)	Gd1B—N6—C36—C37	−131.1 (3)
N8—Al1—O2—C1	74.0 (2)	O17—C36—C37—C38	8.1 (5)
O17—Al2—O3—C7	163.5 (3)	N6—C36—C37—C38	−170.0 (4)
O5—Al2—O3—C7	68.8 (3)	O17—C36—C37—C42	−172.7 (3)
O16—Al2—O3—C7	−114.8 (3)	N6—C36—C37—C42	9.2 (5)
N1—Al2—O3—C7	−26.6 (3)	C42—C37—C38—C39	−1.8 (7)
Gd1B—Al2—O3—C7	−81.4 (3)	C36—C37—C38—C39	177.4 (4)
Gd1—Al2—O3—C7	−76.6 (3)	C37—C38—C39—C40	0.9 (9)
O17—Al2—O4—N2	−103.27 (18)	C38—C39—C40—C41	0.0 (10)
O5—Al2—O4—N2	−9.13 (17)	C39—C40—C41—C42	0.0 (10)
O16—Al2—O4—N2	176.02 (18)	Al5—O18—C42—C41	177.2 (4)
N1—Al2—O4—N2	86.88 (18)	Al5—O18—C42—C37	−3.5 (6)
Gd1B—Al2—O4—N2	145.9 (2)	C40—C41—C42—O18	178.5 (5)
Gd1—Al2—O4—N2	141.1 (2)	C40—C41—C42—C37	−0.9 (8)
O17—Al2—O4—Gd1B	110.86 (11)	C38—C37—C42—O18	−177.5 (4)
O5—Al2—O4—Gd1B	−155.01 (11)	C36—C37—C42—O18	3.3 (6)
O16—Al2—O4—Gd1B	30.14 (10)	C38—C37—C42—C41	1.8 (6)
N1—Al2—O4—Gd1B	−58.99 (11)	C36—C37—C42—C41	−177.4 (4)
O17—Al2—O4—Gd1	115.64 (10)	Al5—O20—C43—N7	13.7 (4)
O5—Al2—O4—Gd1	−150.22 (10)	Al5—O20—C43—C44	−165.7 (3)
O16—Al2—O4—Gd1	34.92 (9)	O19—N7—C43—O20	2.7 (5)
N1—Al2—O4—Gd1	−54.21 (10)	Al6—N7—C43—O20	−164.5 (3)
O3—Al2—O5—C8B	−166.7 (2)	O19—N7—C43—C44	−177.8 (3)
O17—Al2—O5—C8B	101.5 (2)	Al6—N7—C43—C44	14.9 (5)
O4—Al2—O5—C8B	8.5 (2)	O20—C43—C44—C45	−4.7 (5)
O16—Al2—O5—C8B	23.5 (4)	N7—C43—C44—C45	175.8 (4)
N1—Al2—O5—C8B	−78.1 (2)	O20—C43—C44—C49	176.9 (3)
Gd1B—Al2—O5—C8B	−12.2 (2)	N7—C43—C44—C49	−2.6 (6)
O3—Al2—O5—C8	−166.7 (2)	C49—C44—C45—C46	−1.2 (7)
O17—Al2—O5—C8	101.5 (2)	C43—C44—C45—C46	−179.6 (4)
O4—Al2—O5—C8	8.5 (2)	C44—C45—C46—C47	−0.3 (7)
O16—Al2—O5—C8	23.5 (4)	C45—C46—C47—C48	1.4 (7)
N1—Al2—O5—C8	−78.1 (2)	C46—C47—C48—C49	−1.1 (7)
Gd1—Al2—O5—C8	−14.3 (2)	Al6—O21—C49—C48	167.9 (3)
O24—Al1—O9—C21	−60.3 (2)	Al6—O21—C49—C44	−13.5 (5)
O2—Al1—O9—C21	−156.4 (2)	C47—C48—C49—O21	178.2 (4)
O1—Al1—O9—C21	122.1 (2)	C47—C48—C49—C44	−0.4 (6)
N3—Al1—O9—C21	40.3 (2)	C45—C44—C49—O21	−177.1 (4)
C15—O8—Al4—O27	−164.5 (2)	C43—C44—C49—O21	1.3 (6)
C15—O8—Al4—O11	108.7 (2)	C45—C44—C49—C48	1.5 (6)
C15—O8—Al4—O7	14.0 (2)	C43—C44—C49—C48	179.9 (3)
C15—O8—Al4—O10	13.0 (5)	Al6—O23—C50—N8	1.1 (4)
C15—O8—Al4—N9	−78.0 (2)	Al6—O23—C50—C51	177.9 (2)
C15—O8—Al4—Gd1	−8.8 (2)	O22—N8—C50—O23	2.8 (4)
Al4—O10—N4—C22	4.2 (4)	Al1—N8—C50—O23	−165.7 (2)
Gd1—O10—N4—C22	115.7 (3)	O22—N8—C50—C51	−173.8 (3)
O8—Al4—O11—C22	−159.7 (2)	Al1—N8—C50—C51	17.6 (4)
O27—Al4—O11—C22	108.5 (3)	O23—C50—C51—C52	5.4 (5)
O7—Al4—O11—C22	−77.0 (3)	N8—C50—C51—C52	−177.9 (4)
O10—Al4—O11—C22	0.5 (3)	O23—C50—C51—C56	−168.0 (3)
N9—Al4—O11—C22	42.1 (6)	N8—C50—C51—C56	8.7 (5)
Gd1—Al4—O11—C22	−39.0 (3)	C56—C51—C52—C53	2.8 (7)
Al4—O11—C22—N4	1.8 (4)	C50—C51—C52—C53	−170.7 (4)
Al4—O11—C22—C23	−173.5 (3)	C51—C52—C53—C54	0.9 (8)
O10—N4—C22—O11	−4.2 (5)	C52—C53—C54—C55	−3.4 (8)
O10—N4—C22—C23	171.1 (3)	C53—C54—C55—C56	2.3 (7)
O11—C22—C23—C24	−2.5 (6)	Al1—O24—C56—C51	−21.6 (5)
N4—C22—C23—C24	−177.6 (4)	Al1—O24—C56—C55	159.6 (2)
O11—C22—C23—C28	175.3 (4)	C52—C51—C56—O24	177.3 (4)
N4—C22—C23—C28	0.2 (6)	C50—C51—C56—O24	−9.4 (5)
C28—C23—C24—C25	−1.3 (7)	C52—C51—C56—C55	−3.8 (5)
C22—C23—C24—C25	176.6 (4)	C50—C51—C56—C55	169.4 (3)
C23—C24—C25—C26	0.7 (8)	C54—C55—C56—O24	−179.7 (4)
C24—C25—C26—C27	−0.1 (9)	C54—C55—C56—C51	1.3 (5)
C25—C26—C27—C28	0.1 (9)	C68—N10—C64—C65	−1.2 (7)
C24—C23—C28—O12	−179.3 (4)	Al3—N10—C64—C65	178.6 (4)
C22—C23—C28—O12	2.9 (7)	N10—C64—C65—C66	1.9 (10)
C24—C23—C28—C27	1.2 (7)	C64—C65—C66—C67	−1.6 (12)
C22—C23—C28—C27	−176.6 (4)	C65—C66—C67—C68	0.5 (13)
C26—C27—C28—O12	179.9 (5)	C64—N10—C68—C67	0.1 (8)
C26—C27—C28—C23	−0.7 (8)	Al3—N10—C68—C67	−179.7 (5)
O26—Al3—O25—N9	−12.5 (3)	C66—C67—C68—N10	0.2 (11)
N2—Al3—O25—N9	166.2 (3)	C78—N12—C74—C75	−2.3 (8)
O28—Al3—O25—N9	78.1 (3)	N12—C74—C75—C76	1.0 (9)
N10—Al3—O25—N9	−103.0 (3)	C74—C75—C76—C77	1.2 (9)
O26—Al3—O25—Gd1	−177.2 (3)	C75—C76—C77—C78	−2.3 (9)
N2—Al3—O25—Gd1	1.6 (3)	C74—N12—C78—C77	1.1 (7)
O28—Al3—O25—Gd1	−86.5 (3)	C76—C77—C78—N12	1.2 (8)
N10—Al3—O25—Gd1	92.3 (3)	C83—N13—C79—C80	−2.9 (7)
Al3—O25—N9—C57	11.8 (4)	N13—C79—C80—C81	1.3 (8)
Gd1—O25—N9—C57	178.7 (3)	C79—C80—C81—C82	−0.2 (9)
Al3—O25—N9—Al4	−159.6 (2)	C80—C81—C82—C83	0.7 (10)
Gd1—O25—N9—Al4	7.3 (4)	C79—N13—C83—C82	3.4 (8)
O6—Al3—O26—C57	−165.5 (3)	C81—C82—C83—N13	−2.3 (10)
O25—Al3—O26—C57	11.2 (3)	C88—N14—C84—C85	2 (3)
O28—Al3—O26—C57	−75.3 (3)	N14—C84—C85—C86	−1 (3)
N10—Al3—O26—C57	102.7 (3)	C84—C85—C86—C87	2 (3)
O25—N9—C57—O26	−3.0 (6)	C85—C86—C87—C88	−5 (2)
Al4—N9—C57—O26	167.2 (3)	C84—N14—C88—C87	−5 (3)
O25—N9—C57—C58	176.8 (4)	C86—C87—C88—N14	6 (3)
Al4—N9—C57—C58	−13.1 (6)	C128—N22—C124—C125	−6 (6)
Al3—O26—C57—N9	−7.5 (5)	N22—C124—C125—C126	7 (5)
Al3—O26—C57—C58	172.7 (3)	C124—C125—C126—C127	2 (4)
N9—C57—C58—C59	172.8 (4)	C125—C126—C127—C128	−13 (4)
O26—C57—C58—C59	−7.5 (6)	C124—N22—C128—C127	−6 (5)
N9—C57—C58—C63	−9.1 (6)	C126—C127—C128—N22	15 (4)
O26—C57—C58—C63	170.6 (4)	C133—N15—C89—N23	0 (100)
C63—C58—C59—C60	−0.2 (7)	C129—N15—C89—N23	0 (100)
C57—C58—C59—C60	178.0 (4)	C93—N15—C89—N23	0 (100)
C58—C59—C60—C61	1.3 (8)	C133—N15—C89—C90	25 (3)
C59—C60—C61—C62	−1.2 (8)	C129—N15—C89—C90	−55 (8)
C60—C61—C62—C63	−0.1 (7)	C93—N15—C89—C90	5 (3)
C61—C62—C63—O27	−178.3 (4)	C133—N15—C89—C130	22 (3)
C61—C62—C63—C58	1.2 (6)	C129—N15—C89—C130	−58 (8)
C59—C58—C63—O27	178.4 (4)	C93—N15—C89—C130	3 (3)
C57—C58—C63—O27	0.3 (6)	N23—C89—C90—C129	65 (7)
C59—C58—C63—C62	−1.0 (6)	N15—C89—C90—C129	65 (7)
C57—C58—C63—C62	−179.1 (4)	C130—C89—C90—C129	81 (23)
C62—C63—O27—Al4	−148.1 (3)	N23—C89—C90—C91	1 (4)
C58—C63—O27—Al4	32.5 (5)	N15—C89—C90—C91	1 (4)
O8—Al4—O27—C63	56.9 (3)	C130—C89—C90—C91	16 (20)
O11—Al4—O27—C63	157.0 (3)	N23—C89—C90—C131	−27 (3)
O10—Al4—O27—C63	−122.2 (3)	N15—C89—C90—C131	−27 (3)
N9—Al4—O27—C63	−39.7 (3)	C130—C89—C90—C131	−11 (20)
Gd1—Al4—O27—C63	−83.0 (3)	C129—C90—C91—C131	−91 (3)
C73—N11—C69—C70	−1.5 (7)	C89—C90—C91—C131	−81 (3)
Al6—N11—C69—C70	168.3 (4)	C89—C90—C91—C130	−81 (28)
N11—C69—C70—C71	−0.8 (8)	C131—C90—C91—C130	0 (28)
C69—C70—C71—C72	1.6 (8)	C129—C90—C91—C92	−11 (3)
C70—C71—C72—C73	−0.2 (8)	C89—C90—C91—C92	−2 (3)
C69—N11—C73—C72	3.1 (7)	C131—C90—C91—C92	79 (2)
Al6—N11—C73—C72	−166.5 (4)	C129—C90—C91—C132	−42 (3)
C71—C72—C73—N11	−2.3 (8)	C89—C90—C91—C132	−33 (3)
N3—O7—Al4B—O27B	−8 (3)	C131—C90—C91—C132	48.4 (16)
Gd1B—O7—Al4B—O27B	−146 (2)	C131—C91—C92—C132	9 (2)
N3—O7—Al4B—O11B	−108.6 (6)	C130—C91—C92—C132	−75 (3)
Gd1B—O7—Al4B—O11B	113.1 (5)	C90—C91—C92—C132	−79 (2)
N3—O7—Al4B—O10B	168.7 (4)	C131—C91—C92—C93	85 (2)
Gd1B—O7—Al4B—O10B	30.4 (5)	C130—C91—C92—C93	0 (3)
N3—O7—Al4B—N9B	82.8 (7)	C90—C91—C92—C93	−3 (2)
Gd1B—O7—Al4B—N9B	−55.4 (7)	C132—C91—C92—C93	75.6 (18)
N3—O7—Al4B—O8	−8.0 (4)	C130—C91—C92—C131	−85 (3)
Gd1B—O7—Al4B—O8	−146.31 (15)	C90—C91—C92—C131	−88 (3)
N3—O7—Al4B—Gd1B	138.3 (4)	C132—C91—C92—C131	−9 (2)
Al4B—O10B—N4B—C22B	8 (2)	C131—C91—C92—C133	70 (2)
Gd1B—O10B—N4B—C22B	112.0 (17)	C130—C91—C92—C133	−14 (3)
Al4B—O11B—C22B—N4B	−31 (2)	C90—C91—C92—C133	−18 (2)
Al4B—O11B—C22B—C23B	157.8 (19)	C132—C91—C92—C133	61.1 (15)
O10B—N4B—C22B—O11B	15 (3)	C89—N15—C93—N23	0 (100)
O10B—N4B—C22B—C23B	−172.6 (18)	C133—N15—C93—N23	0 (100)
O11B—C22B—C23B—C24B	17 (4)	C129—N15—C93—N23	0 (100)
N4B—C22B—C23B—C24B	−154 (2)	C89—N15—C93—C92	−11 (2)
O11B—C22B—C23B—C28B	−156 (2)	C133—N15—C93—C92	−112 (5)
N4B—C22B—C23B—C28B	33 (4)	C129—N15—C93—C92	−1 (2)
C28B—C23B—C24B—C25B	1 (4)	C89—N15—C93—C132	31.6 (19)
C22B—C23B—C24B—C25B	−173 (3)	C133—N15—C93—C132	−70 (4)
C23B—C24B—C25B—C26B	−3 (4)	C129—N15—C93—C132	42 (2)
C24B—C25B—C26B—C27B	9 (5)	C132—C92—C93—N23	99 (2)
C25B—C26B—C27B—C28B	−13 (5)	C91—C92—C93—N23	10 (2)
C24B—C23B—C28B—O12B	177 (2)	C131—C92—C93—N23	40 (2)
C22B—C23B—C28B—O12B	−10 (4)	C133—C92—C93—N23	93 (4)
C24B—C23B—C28B—C27B	−4 (4)	C132—C92—C93—N15	99 (2)
C22B—C23B—C28B—C27B	169 (2)	C91—C92—C93—N15	10 (2)
C26B—C27B—C28B—O12B	−171 (3)	C131—C92—C93—N15	40 (2)
C26B—C27B—C28B—C23B	10 (4)	C133—C92—C93—N15	93 (4)
C23B—C28B—O12B—Al6	109 (3)	C91—C92—C93—C132	−89.5 (19)
C27B—C28B—O12B—Al6	−70 (3)	C131—C92—C93—C132	−58.7 (16)
Al3—O25B—N9B—C57B	17 (3)	C133—C92—C93—C132	−6 (4)
Gd1B—O25B—N9B—C57B	174 (2)	N15—C89—N23—C133	0 (100)
Al3—O25B—N9B—Al4B	−152.9 (14)	C90—C89—N23—C133	25 (3)
Gd1B—O25B—N9B—Al4B	4 (3)	C130—C89—N23—C133	22 (3)
O6B—Al3—O26B—C57B	−163 (2)	N15—C89—N23—C129	0 (100)
N2—Al3—O26B—C57B	33 (5)	C90—C89—N23—C129	−55 (8)
O28—Al3—O26B—C57B	−72 (2)	C130—C89—N23—C129	−58 (8)
O25B—Al3—O26B—C57B	20 (2)	N15—C89—N23—C93	0 (82)
N10—Al3—O26B—C57B	106 (2)	C90—C89—N23—C93	5 (3)
O25B—N9B—C57B—O26B	−2 (4)	C130—C89—N23—C93	3 (3)
Al4B—N9B—C57B—O26B	167 (2)	N15—C93—N23—C89	0 (100)
O25B—N9B—C57B—C58B	173 (3)	C92—C93—N23—C89	−11 (2)
Al4B—N9B—C57B—C58B	−18 (4)	C132—C93—N23—C89	31.6 (19)
Al3—O26B—C57B—N9B	−17 (4)	N15—C93—N23—C133	0 (100)
Al3—O26B—C57B—C58B	168 (2)	C92—C93—N23—C133	−112 (5)
N9B—C57B—C58B—C59B	174 (3)	C132—C93—N23—C133	−70 (4)
O26B—C57B—C58B—C59B	−10 (4)	N15—C93—N23—C129	0 (100)
N9B—C57B—C58B—C63B	−3 (4)	C92—C93—N23—C129	−1 (2)
O26B—C57B—C58B—C63B	173 (3)	C132—C93—N23—C129	42 (2)
C63B—C58B—C59B—C60B	−4 (4)	C89—C90—C129—N23	−94 (9)
C57B—C58B—C59B—C60B	179 (3)	C91—C90—C129—N23	26 (5)
C58B—C59B—C60B—C61B	5 (4)	C131—C90—C129—N23	−2 (5)
C59B—C60B—C61B—C62B	−4 (4)	C89—C90—C129—N15	−94 (9)
C60B—C61B—C62B—C63B	3 (4)	C91—C90—C129—N15	26 (5)
C61B—C62B—C63B—O27B	−177 (2)	C131—C90—C129—N15	−2 (5)
C61B—C62B—C63B—C58B	−2 (4)	C89—C90—C129—C130	−103 (23)
C59B—C58B—C63B—O27B	177 (2)	C91—C90—C129—C130	16 (20)
C57B—C58B—C63B—O27B	−6 (4)	C131—C90—C129—C130	−12 (20)
C59B—C58B—C63B—C62B	2 (4)	C89—N23—C129—C90	103 (11)
C57B—C58B—C63B—C62B	180 (2)	C133—N23—C129—C90	−2 (5)
C62B—C63B—O27B—Al4B	−148.9 (18)	C93—N23—C129—C90	−20 (5)
C58B—C63B—O27B—Al4B	37 (3)	C89—N23—C129—N15	0 (100)
O7—Al4B—O27B—C63B	50 (3)	C133—N23—C129—N15	0 (100)
O11B—Al4B—O27B—C63B	151.5 (18)	C93—N23—C129—N15	0 (100)
O10B—Al4B—O27B—C63B	−126.5 (17)	C89—N23—C129—C130	105 (11)
N9B—Al4B—O27B—C63B	−41.9 (19)	C133—N23—C129—C130	−1 (5)
O8—Al4B—O27B—C63B	50.0 (18)	C93—N23—C129—C130	−18 (4)
Gd1B—Al4B—O27B—C63B	−84 (2)	C89—N15—C129—C90	103 (11)
O3—Al2—O17—C36	122.5 (2)	C133—N15—C129—C90	−2 (5)
O5—Al2—O17—C36	−139.9 (2)	C93—N15—C129—C90	−20 (5)
O4—Al2—O17—C36	−57.3 (2)	C89—N15—C129—N23	0 (100)
O16—Al2—O17—C36	20.4 (2)	C133—N15—C129—N23	0 (100)
N1—Al2—O17—C36	37.5 (8)	C93—N15—C129—N23	0 (100)
Gd1B—Al2—O17—C36	−13.7 (2)	C89—N15—C129—C130	105 (11)
Gd1—Al2—O17—C36	−16.6 (2)	C133—N15—C129—C130	−1 (5)
O20—Al5—O18—C42	−103.8 (4)	C93—N15—C129—C130	−18 (4)
O13—Al5—O18—C42	79.8 (4)	C131—C91—C130—C129	−95 (4)
O14—Al5—O18—C42	161.4 (4)	C90—C91—C130—C129	85 (29)
N6—Al5—O18—C42	−4.3 (4)	C92—C91—C130—C129	−17 (5)
Gd1B—Al5—O18—C42	37.9 (5)	C132—C91—C130—C129	−47 (4)
Gd1—Al5—O18—C42	46.3 (5)	C90—C91—C130—C131	180 (100)
O18—Al5—O20—C43	159.1 (3)	C92—C91—C130—C131	78 (2)
O19—Al5—O20—C43	−18.2 (3)	C132—C91—C130—C131	47.6 (17)
O13—Al5—O20—C43	−34.6 (6)	C131—C91—C130—C89	−86 (4)
O14—Al5—O20—C43	−110.5 (3)	C92—C91—C130—C89	−8 (5)
N6—Al5—O20—C43	68.7 (3)	C132—C91—C130—C89	−38 (5)
Gd1B—Al5—O20—C43	8.7 (3)	C90—C129—C130—C91	−159 (25)
Gd1—Al5—O20—C43	1.7 (3)	N23—C129—C130—C91	29 (6)
O23—Al6—O21—C49	−161.8 (3)	N15—C129—C130—C91	29 (6)
N7—Al6—O21—C49	18.8 (3)	C90—C129—C130—C131	166 (25)
O12B—Al6—O21—C49	91.6 (6)	N23—C129—C130—C131	−6 (6)
O29—Al6—O21—C49	−68.6 (3)	N15—C129—C130—C131	−6 (6)
N11—Al6—O21—C49	110.2 (3)	C90—C129—C130—C89	79 (23)
O23—Al6—O22—N8	4.80 (19)	N23—C129—C130—C89	−92 (8)
N7—Al6—O22—N8	−176.3 (2)	N15—C129—C130—C89	−92 (8)
O12B—Al6—O22—N8	110.9 (5)	N23—C89—C130—C91	7 (5)
O29—Al6—O22—N8	−88.8 (2)	N15—C89—C130—C91	7 (5)
N11—Al6—O22—N8	92.2 (2)	C90—C89—C130—C91	−159 (26)
O23—Al6—O22—Gd1B	163.54 (16)	N23—C89—C130—C129	70 (6)
N7—Al6—O22—Gd1B	−17.57 (17)	N15—C89—C130—C129	70 (6)
O12B—Al6—O22—Gd1B	−90.3 (5)	C90—C89—C130—C129	−96 (23)
O29—Al6—O22—Gd1B	69.89 (16)	N23—C89—C130—C131	−28 (5)
O23—Al6—O22—Gd1	173.71 (17)	N15—C89—C130—C131	−28 (5)
N7—Al6—O22—Gd1	−7.41 (18)	C90—C89—C130—C131	166 (25)
O29—Al6—O22—Gd1	80.06 (16)	C90—C91—C131—C130	0.0 (14)
N11—Al6—O22—Gd1	−98.92 (17)	C92—C91—C131—C130	−120.3 (13)
O21—Al6—O23—C50	171.4 (2)	C132—C91—C131—C130	−114.5 (17)
O22—Al6—O23—C50	−3.4 (2)	C130—C91—C131—C132	114.5 (17)
O12B—Al6—O23—C50	−94.4 (6)	C90—C91—C131—C132	114.5 (17)
O29—Al6—O23—C50	83.8 (2)	C92—C91—C131—C132	−5.8 (15)
N11—Al6—O23—C50	−99.6 (2)	C130—C91—C131—C92	120.3 (13)
O2—Al1—O24—C56	−60.1 (3)	C90—C91—C131—C92	120.3 (10)
O9—Al1—O24—C56	−149.0 (3)	C132—C91—C131—C92	5.8 (15)
N3—Al1—O24—C56	126.7 (3)	C130—C91—C131—C90	0.0 (14)
N8—Al1—O24—C56	34.8 (3)	C92—C91—C131—C90	−120.3 (10)
Al1—O1—N1—C1	−11.4 (3)	C132—C91—C131—C90	−114.5 (17)
Gd1B—O1—N1—C1	−145.9 (2)	C129—C130—C131—C91	101 (4)
Gd1—O1—N1—C1	−145.81 (19)	C89—C130—C131—C91	112 (4)
Al1—O1—N1—Al2	168.80 (12)	C91—C130—C131—C132	−92 (3)
Gd1B—O1—N1—Al2	34.26 (19)	C129—C130—C131—C132	9 (5)
Gd1—O1—N1—Al2	34.4 (2)	C89—C130—C131—C132	19 (5)
Al1—O1—N1—Gd1B	134.55 (17)	C91—C130—C131—C92	−58 (2)
Al2—O4—N2—C8B	8.2 (3)	C129—C130—C131—C92	43 (4)
Gd1B—O4—N2—C8B	136.5 (2)	C89—C130—C131—C92	53 (4)
Al2—O4—N2—C8	8.2 (3)	C132—C92—C131—C91	−170 (2)
Gd1—O4—N2—C8	139.1 (2)	C93—C92—C131—C91	−108 (2)
Al2—O4—N2—Al3	−162.24 (12)	C133—C92—C131—C91	−119 (2)
Gd1B—O4—N2—Al3	−34.0 (3)	C132—C92—C131—C130	−121 (2)
Gd1—O4—N2—Al3	−31.3 (3)	C93—C92—C131—C130	−59 (2)
Al4B—O7—N3—C15	6.1 (5)	C91—C92—C131—C130	49 (2)
Al4—O7—N3—C15	8.4 (3)	C133—C92—C131—C130	−70 (2)
Gd1—O7—N3—C15	137.5 (2)	C93—C92—C131—C132	61.9 (15)
Gd1B—O7—N3—C15	140.7 (2)	C91—C92—C131—C132	170 (2)
Al4B—O7—N3—Al1	−173.2 (4)	C133—C92—C131—C132	51.2 (13)
Al4—O7—N3—Al1	−170.95 (12)	C132—C92—C131—C90	−120 (2)
Gd1—O7—N3—Al1	−41.9 (2)	C93—C92—C131—C90	−57.7 (19)
Gd1B—O7—N3—Al1	−38.7 (2)	C91—C92—C131—C90	50.7 (17)
Al5—O13—N5—C29	5.0 (4)	C133—C92—C131—C90	−68.4 (17)
Gd1B—O13—N5—C29	−106.7 (3)	C129—C90—C131—C91	99 (3)
Gd1—O13—N5—C29	−108.5 (3)	C89—C90—C131—C91	109 (3)
Al2—O16—N6—C36	16.9 (3)	C129—C90—C131—C132	5 (4)
Gd1B—O16—N6—C36	121.9 (2)	C89—C90—C131—C132	16 (3)
Gd1—O16—N6—C36	123.0 (2)	C91—C90—C131—C132	−93 (3)
Al2—O16—N6—Al5	−146.49 (15)	C129—C90—C131—C92	40 (3)
Gd1B—O16—N6—Al5	−41.5 (2)	C89—C90—C131—C92	50 (2)
Gd1—O16—N6—Al5	−40.4 (2)	C91—C90—C131—C92	−58.6 (19)
Al2—O16—N6—Gd1B	−104.97 (16)	C93—C92—C132—C133	−1.6 (10)
Al5—O19—N7—C43	−17.3 (3)	C91—C92—C132—C133	115.2 (11)
Gd1B—O19—N7—C43	−131.2 (2)	C131—C92—C132—C133	119.8 (13)
Gd1—O19—N7—C43	−141.6 (2)	C93—C92—C132—C131	−121.4 (11)
Al5—O19—N7—Al6	151.09 (15)	C91—C92—C132—C131	−4.6 (12)
Gd1B—O19—N7—Al6	37.3 (3)	C133—C92—C132—C131	−119.8 (13)
Gd1—O19—N7—Al6	26.8 (3)	C91—C92—C132—C93	116.7 (11)
Al6—O22—N8—C50	−5.3 (3)	C131—C92—C132—C93	121.4 (11)
Gd1B—O22—N8—C50	−164.8 (2)	C133—C92—C132—C93	1.6 (10)
Gd1—O22—N8—C50	−175.7 (2)	C93—C92—C132—C91	−116.7 (11)
Al6—O22—N8—Al1	163.55 (14)	C131—C92—C132—C91	4.6 (12)
Gd1B—O22—N8—Al1	4.1 (3)	C133—C92—C132—C91	−115.2 (11)
Gd1—O22—N8—Al1	−6.8 (3)	C91—C131—C132—C92	10 (2)
Al1—O2—C1—N1	7.0 (4)	C130—C131—C132—C92	72 (3)
Al1—O2—C1—C2	−173.4 (2)	C90—C131—C132—C92	74 (3)
O1—N1—C1—O2	3.0 (4)	C91—C131—C132—C133	−68 (3)
Al2—N1—C1—O2	−177.2 (2)	C130—C131—C132—C133	−6 (4)
Gd1B—N1—C1—O2	−48.8 (5)	C92—C131—C132—C133	−78 (2)
O1—N1—C1—C2	−176.5 (2)	C90—C131—C132—C133	−4 (4)
Al2—N1—C1—C2	3.3 (4)	C91—C131—C132—C93	−51 (3)
Gd1B—N1—C1—C2	131.6 (3)	C130—C131—C132—C93	11 (4)
O2—C1—C2—C3	−12.4 (4)	C92—C131—C132—C93	−60.7 (17)
N1—C1—C2—C3	167.2 (3)	C90—C131—C132—C93	13 (3)
O2—C1—C2—C7	165.0 (3)	C130—C131—C132—C91	62 (3)
N1—C1—C2—C7	−15.4 (5)	C92—C131—C132—C91	−10 (3)
C7—C2—C3—C4	−2.6 (5)	C90—C131—C132—C91	64 (2)
C1—C2—C3—C4	174.8 (3)	N23—C93—C132—C92	−103.4 (18)
C2—C3—C4—C5	0.1 (6)	N15—C93—C132—C92	−103.4 (18)
C3—C4—C5—C6	1.7 (7)	N23—C93—C132—C133	71 (3)
C4—C5—C6—C7	−0.9 (7)	N15—C93—C132—C133	71 (3)
Al2—O3—C7—C6	−159.3 (3)	C92—C93—C132—C133	174 (4)
Al2—O3—C7—C2	22.8 (5)	N23—C93—C132—C131	−39 (3)
C5—C6—C7—O3	−179.6 (4)	N15—C93—C132—C131	−39 (3)
C5—C6—C7—C2	−1.6 (6)	C92—C93—C132—C131	65 (2)
C3—C2—C7—O3	−178.7 (3)	N23—C93—C132—C91	−56.6 (18)
C1—C2—C7—O3	3.9 (5)	N15—C93—C132—C91	−56.6 (18)
C3—C2—C7—C6	3.3 (5)	C92—C93—C132—C91	46.7 (11)
C1—C2—C7—C6	−174.0 (3)	C131—C91—C132—C92	−168 (3)
Al2—O5—C8—N2	−6.5 (4)	C130—C91—C132—C92	117 (3)
Al2—O5—C8—C9	176.3 (6)	C90—C91—C132—C92	115 (2)
O4—N2—C8—O5	−1.1 (4)	C131—C91—C132—C133	120 (3)
Al3—N2—C8—O5	168.0 (2)	C130—C91—C132—C133	46 (3)
O4—N2—C8—C9	176.1 (6)	C90—C91—C132—C133	44 (2)
Al3—N2—C8—C9	−14.8 (7)	C92—C91—C132—C133	−71.2 (19)
O5—C8—C9—C10	−2.9 (12)	C130—C91—C132—C131	−74 (3)
N2—C8—C9—C10	179.8 (6)	C90—C91—C132—C131	−77 (3)
O5—C8—C9—C14	174.4 (7)	C92—C91—C132—C131	168 (3)
N2—C8—C9—C14	−2.9 (13)	C131—C91—C132—C93	134 (3)
C14—C9—C10—C11	0.8 (13)	C130—C91—C132—C93	59 (3)
C8—C9—C10—C11	178.1 (7)	C90—C91—C132—C93	57 (2)
C9—C10—C11—C12	−1.0 (9)	C92—C91—C132—C93	−57.9 (15)
C10—C11—C12—C13	0.2 (8)	C89—N23—C133—N15	0 (100)
C11—C12—C13—C14	0.9 (8)	C129—N23—C133—N15	0 (100)
C12—C13—C14—O6	179.2 (5)	C93—N23—C133—N15	0 (100)
C12—C13—C14—C9	−1.1 (10)	C89—N23—C133—C132	−8 (3)
C10—C9—C14—O6	179.9 (7)	C129—N23—C133—C132	4 (3)
C8—C9—C14—O6	2.7 (14)	C93—N23—C133—C132	80 (4)
C10—C9—C14—C13	0.3 (13)	C89—N23—C133—C92	−41 (2)
C8—C9—C14—C13	−176.9 (8)	C129—N23—C133—C92	−29 (3)
C13—C14—O6—Al3	−164.3 (4)	C93—N23—C133—C92	47 (3)
C9—C14—O6—Al3	16.2 (9)	C89—N15—C133—N23	0 (100)
O26—Al3—O6—C14	154.2 (4)	C129—N15—C133—N23	0 (100)
N2—Al3—O6—C14	−24.3 (4)	C93—N15—C133—N23	0 (100)
O28—Al3—O6—C14	63.8 (4)	C89—N15—C133—C132	−8 (3)
N10—Al3—O6—C14	−115.1 (4)	C129—N15—C133—C132	4 (3)
Al2—O5—C8B—N2	−6.5 (4)	C93—N15—C133—C132	80 (4)
Al2—O5—C8B—C9B	170 (4)	C89—N15—C133—C92	−41 (2)
O4—N2—C8B—O5	−1.1 (4)	C129—N15—C133—C92	−29 (3)
Al3—N2—C8B—O5	168.0 (2)	C93—N15—C133—C92	47 (3)
O4—N2—C8B—C9B	−178 (4)	C92—C132—C133—N23	−73 (3)
Al3—N2—C8B—C9B	−9 (4)	C131—C132—C133—N23	−1 (4)
O5—C8B—C9B—C10B	−6 (8)	C93—C132—C133—N23	−79 (3)
N2—C8B—C9B—C10B	170 (4)	C91—C132—C133—N23	−23 (3)
O5—C8B—C9B—C14B	178 (5)	C92—C132—C133—N15	−73 (3)
N2—C8B—C9B—C14B	−5 (9)	C131—C132—C133—N15	−1 (4)
C14B—C9B—C10B—C11B	0 (9)	C93—C132—C133—N15	−79 (3)
C8B—C9B—C10B—C11B	−175 (5)	C91—C132—C133—N15	−23 (3)
C9B—C10B—C11B—C12B	2 (7)	C131—C132—C133—C92	72 (2)
C10B—C11B—C12B—C13B	−2 (5)	C93—C132—C133—C92	−5 (3)
C11B—C12B—C13B—C14B	0 (5)	C91—C132—C133—C92	50.0 (13)
C12B—C13B—C14B—O6B	−178 (4)	C132—C92—C133—N23	122 (2)
C12B—C13B—C14B—C9B	2 (7)	C93—C92—C133—N23	−65 (3)
C10B—C9B—C14B—O6B	178 (6)	C91—C92—C133—N23	38 (2)
C8B—C9B—C14B—O6B	−7 (10)	C131—C92—C133—N23	65.7 (19)
C10B—C9B—C14B—C13B	−2 (9)	C132—C92—C133—N15	122 (2)
C8B—C9B—C14B—C13B	174 (6)	C93—C92—C133—N15	−65 (3)
C13B—C14B—O6B—Al3	−147 (3)	C91—C92—C133—N15	38 (2)
C9B—C14B—O6B—Al3	33 (7)	C131—C92—C133—N15	65.7 (19)
O26B—Al3—O6B—C14B	149 (3)	C93—C92—C133—C132	173 (4)
N2—Al3—O6B—C14B	−35 (3)	C91—C92—C133—C132	−83.6 (19)
O28—Al3—O6B—C14B	53 (3)	C131—C92—C133—C132	−56.1 (16)
N10—Al3—O6B—C14B	−126 (3)	C143—N25—C139—C140	0.0
Al4—O8—C15—N3	−13.9 (4)	N25—C139—C140—C141	0.0
Al4B—O8—C15—N3	−7.9 (4)	C139—C140—C141—C142	0.0
Al4—O8—C15—C16	163.8 (2)	C140—C141—C142—C143	0.0
Al4B—O8—C15—C16	169.8 (3)	C141—C142—C143—N25	0.0
O7—N3—C15—O8	3.3 (4)	C139—N25—C143—C142	0.0
Al1—N3—C15—O8	−177.5 (2)	C98—N16—C94—C95	−0.2 (7)
O7—N3—C15—C16	−174.5 (2)	N16—C94—C95—C96	−0.7 (7)
Al1—N3—C15—C16	4.8 (4)	C94—C95—C96—C97	1.9 (9)
O8—C15—C16—C17	11.8 (4)	C95—C96—C97—C98	−2.2 (10)
N3—C15—C16—C17	−170.4 (3)	C94—N16—C98—C97	−0.1 (8)
O8—C15—C16—C21	−164.2 (3)	C96—C97—C98—N16	1.3 (10)
N3—C15—C16—C21	13.6 (4)	C103—N17—C99—C100	6 (2)
C21—C16—C17—C18	−3.0 (5)	N17—C99—C100—C101	−21 (2)
C15—C16—C17—C18	−179.0 (3)	C99—C100—C101—C102	30 (2)
C16—C17—C18—C19	0.1 (5)	C100—C101—C102—C103	−27 (2)
C17—C18—C19—C20	1.6 (5)	C99—N17—C103—C102	−2.8 (19)
C18—C19—C20—C21	−0.4 (5)	C101—C102—C103—N17	13.7 (19)
Al1—O9—C21—C20	144.9 (2)	C138—N24—C134—C135	−5 (3)
Al1—O9—C21—C16	−37.0 (4)	N24—C134—C135—C136	31 (3)
C19—C20—C21—O9	175.8 (3)	C134—C135—C136—C137	−54 (3)
C19—C20—C21—C16	−2.5 (4)	C135—C136—C137—C138	52 (3)
C17—C16—C21—O9	−174.1 (3)	C134—N24—C138—C137	5 (3)
C15—C16—C21—O9	1.9 (4)	C136—C137—C138—N24	−29 (3)
C17—C16—C21—C20	4.1 (4)	C108—N18—C104—C105	−1.0 (9)
C15—C16—C21—C20	−180.0 (3)	N18—C104—C105—C106	1.3 (10)
Al5—O14—C29—N5	0.1 (4)	C104—C105—C106—C107	−0.9 (10)
Al5—O14—C29—C30	−178.3 (3)	C105—C106—C107—C108	0.3 (12)
O13—N5—C29—O14	−3.5 (5)	C104—N18—C108—C107	0.3 (12)
O13—N5—C29—C30	175.0 (3)	C106—C107—C108—N18	0.0 (14)
O14—C29—C30—C31	5.5 (6)	C113—N19—C109—C110	1.0 (10)
N5—C29—C30—C31	−172.8 (4)	N19—C109—C110—C111	−2.7 (10)
O14—C29—C30—C35	−176.8 (4)	C109—C110—C111—C112	1.9 (8)
N5—C29—C30—C35	4.8 (6)	C110—C111—C112—C113	0.3 (7)
C35—C30—C31—C32	0.9 (7)	C109—N19—C113—C112	1.7 (9)
C29—C30—C31—C32	178.6 (5)	C111—C112—C113—N19	−2.3 (9)
C30—C31—C32—C33	−0.6 (9)	C118—N20—C114—C115	−2.2 (13)
C31—C32—C33—C34	−0.1 (9)	N20—C114—C115—C116	4.0 (13)
C32—C33—C34—C35	0.5 (9)	C114—C115—C116—C117	−2.5 (12)
C33—C34—C35—O15	179.9 (5)	C115—C116—C117—C118	−0.7 (14)
C33—C34—C35—C30	−0.2 (8)	C114—N20—C118—C117	−1.3 (13)
C31—C30—C35—O15	179.4 (5)	C116—C117—C118—N20	2.8 (15)
C29—C30—C35—O15	1.8 (7)	C123—N21—C119—C120	−11 (3)
C31—C30—C35—C34	−0.5 (7)	N21—C119—C120—C121	0 (4)
C29—C30—C35—C34	−178.1 (4)	C119—C120—C121—C122	10 (4)
Al2—O17—C36—N6	−17.1 (4)	C120—C121—C122—C123	−8 (3)
Al2—O17—C36—C37	164.8 (2)	C119—N21—C123—C122	13 (5)
O16—N6—C36—O17	−0.4 (4)	C121—C122—C123—N21	−3 (4)
Al5—N6—C36—O17	161.5 (2)		

### Bis(pyridinium) diaquadipyridinehexakis[µ3-salicylhydroximato(3-)]bis[µ2-salicylhydroximato(1-)]hexaaluminiumgadolinium–pyridine–water (1/7.396/1) (1) Hydrogen-bond geometry (Å, º)

**Table:**

*D*—H···*A*	*D*—H	H···*A*	*D*···*A*	*D*—H···*A*
N4—H4*N*···O12	0.88	1.94	2.590 (4)	130
O12—H12*O*···N15	0.84	1.82	2.615 (5)	158
C62—H62···O17^i^	0.95	2.54	3.354 (5)	143
O15—H15···N14	0.84	1.90	2.693 (14)	157
O15—H15···N22	0.84	1.69	2.51 (2)	163
O28—H28*A*···N13	0.86 (2)	1.78 (2)	2.630 (4)	175 (5)
O29—H29*A*···O16	0.89 (2)	1.84 (2)	2.723 (3)	172 (5)
O29—H29*B*···N18	0.90 (2)	1.88 (3)	2.701 (5)	150 (5)
O30—H30*A*···N19	0.85 (2)	2.11 (2)	2.830 (8)	142 (4)
O30—H30*B*···N20	0.87 (2)	2.22 (2)	3.033 (15)	156 (7)
N5—H5*N*···O15	0.88	1.95	2.617 (5)	131
C68—H68···O4	0.95	2.30	3.093 (5)	141
N12—H12*A*···O9	0.88	1.79	2.624 (4)	157
C74—H74···O28	0.95	2.46	3.379 (5)	164
C130—H130···O18^ii^	0.95	2.47	3.35 (2)	153
N16—H16···O27	0.88	1.76	2.632 (4)	168
C94—H94···O3^i^	0.95	2.29	3.131 (5)	147

Symmetry codes: (i) *x*+1, *y*, *z*; (ii) −*x*, −*y*+1, −*z*+1.

### Bis(pyridinium) diaquadipyridinehexakis[µ3-salicylhydroximato(3-)]bis[µ2-salicylhydroximato(1-)]hexaaluminiumdysprosium–pyridine–water (1/7.429/1) (2) Crystal data

**Table:**

(C_5_H_6_N)_2_[DyAl_6_(C_7_H_6_NO_3_)_2_(C_7_H_4_NO_3_)_7_(C_5_H_5_N)_1.855_(H_2_O)_2_]·7.429C_5_H_5_N·H_2_O	*Z* = 2
*M**_r_* = 2630.90	*F*(000) = 2696.9
Triclinic, *P*1	*D*_x_ = 1.496 Mg m^−^^3^
*a* = 13.7232 (6) Å	Mo *K*α radiation, λ = 0.71073 Å
*b* = 16.0871 (7) Å	Cell parameters from 9221 reflections
*c* = 28.2946 (13) Å	θ = 2.4–32.8°
α = 80.632 (2)°	µ = 0.77 mm^−^^1^
β = 83.989 (2)°	*T* = 150 K
γ = 71.660 (2)°	Block, colourless
*V* = 5840.6 (5) Å^3^	0.21 × 0.18 × 0.10 mm

### Bis(pyridinium) diaquadipyridinehexakis[µ3-salicylhydroximato(3-)]bis[µ2-salicylhydroximato(1-)]hexaaluminiumdysprosium–pyridine–water (1/7.429/1) (2) Data collection

**Table:**

Bruker AXS D8 Quest CMOS diffractometer	35615 independent reflections
Radiation source: fine focus sealed tube X-ray source	31175 reflections with *I* > 2σ(*I*)
Triumph curved graphite crystal monochromator	*R*_int_ = 0.045
Detector resolution: 10.4167 pixels mm^-1^	θ_max_ = 30.5°, θ_min_ = 2.2°
ω and phi scans	*h* = −19→19
Absorption correction: multi-scan (SADABS; Krause *et al.*, 2015)	*k* = −22→22
*T*_min_ = 0.225, *T*_max_ = 0.269	*l* = −40→40
209417 measured reflections	

### Bis(pyridinium) diaquadipyridinehexakis[µ3-salicylhydroximato(3-)]bis[µ2-salicylhydroximato(1-)]hexaaluminiumdysprosium–pyridine–water (1/7.429/1) (2) Refinement

**Table:**

Refinement on *F*^2^	Primary atom site location: structure-invariant direct methods
Least-squares matrix: full	Secondary atom site location: difference Fourier map
*R*[*F*^2^ > 2σ(*F*^2^)] = 0.048	Hydrogen site location: mixed
*wR*(*F*^2^) = 0.116	H atoms treated by a mixture of independent and constrained refinement
*S* = 1.14	*w* = 1/[σ^2^(*F*_o_^2^) + (0.0312*P*)^2^ + 11.504*P*] where *P* = (*F*_o_^2^ + 2*F*_c_^2^)/3
35615 reflections	(Δ/σ)_max_ = 0.001
2107 parameters	Δρ_max_ = 1.32 e Å^−^^3^
2161 restraints	Δρ_min_ = −1.09 e Å^−^^3^

### Bis(pyridinium) diaquadipyridinehexakis[µ3-salicylhydroximato(3-)]bis[µ2-salicylhydroximato(1-)]hexaaluminiumdysprosium–pyridine–water (1/7.429/1) (2) Special details

**Table:**

Geometry. All esds (except the esd in the dihedral angle between two l.s. planes) are estimated using the full covariance matrix. The cell esds are taken into account individually in the estimation of esds in distances, angles and torsion angles; correlations between esds in cell parameters are only used when they are defined by crystal symmetry. An approximate (isotropic) treatment of cell esds is used for estimating esds involving l.s. planes.
Refinement. A section of the main molecule is disordered induced by presence or absence of an aluminum coordinated pyridine ligand. In the absence of the pyridine moiety a salicylate ligand swings into the space otherwise occupied by the pyridine, and the phenol oxygen atom coordinates to the aluminum (Al6). The movement of the salicylate ligand induces shifts to the Dy ion, to the aluminum ion the other two oxygen atoms are coordinated to (Al4) and one of the other salicylate ligands coordinated to Al4. The substantial movement of the first salicylate ligand induces a shift of the solvate pyridine its phenol oxygen is hydrogen bonded to. Another solvate pyridine molecule (adjacent to the second disordered salicylate ligand) was included in the disorder. The two Dy ions were constrained to have identical ADPs. Equivalent sections of the two disordered moieties were restrained to have similar geometries. The minor solvate pyridine ring was constrained to resemble an ideal hexagon with C-C distances of 1.39 Angstrom. The major moiety solvate pyridine ring was refined as additionally disordered. The nitrogen atoms of these two moieties were constrained to share position and ADP. Its two moieties and the other disordered pyridyl ring were restrained to have similar geometries as another not disordered pyridine ring. Uij components of ADPs for disordered atoms closer to each other than 2.0 Angstrom were restrained to be similar. Subject to these conditions the occupancy ratio refined to 0.8909 (13) to 0.1091 (13). The occupancy rates for the additionally split ring are 0.539 (3) and 0.352 (3).Two other solvate pyridyl rings are independently disordered with a shared occupancy ratio (N14, N17 vs N22, N24). The disordered moieties were restrained to have similar geometries as another not disordered pyridine ring. Uij components of ADPs for disordered atoms closer to each other than 2.0 Angstrom were restrained to be similar. Subject to these conditions the occupancy ratio for the one disordered in a general position refined to 0.509 (8) to 0.491 (8).Another solvate pyridyl ring is 1:1 disordered around an inversion center. The disordered moieties were restrained to have similar geometries as another not disordered pyridine ring. Uij components of ADPs for disordered atoms closer to each other than 2.0 Angstrom were restrained to be similar. Subject to these conditions the occupancy rate refined to two times 0.429 (4).Water H atom positions and some amine H atom positions were refined and O-H and selected N-H distances were restrained to 0.84 (2) and 0.88 (2) Angstrom, respectively. Some water, amine and phenol H atom positions were further restrained based on hydrogen bonding considerations (phenol H atoms were placed in calculated positions, but were allowed to rotate around the C-O axis).

### Bis(pyridinium) diaquadipyridinehexakis[µ3-salicylhydroximato(3-)]bis[µ2-salicylhydroximato(1-)]hexaaluminiumdysprosium–pyridine–water (1/7.429/1) (2) Fractional atomic coordinates and isotropic or equivalent isotropic displacement parameters (Å^2^)

**Table:**

	*x*	*y*	*z*	*U*_iso_*/*U*_eq_	Occ. (<1)
Al1	−0.03575 (6)	0.87433 (5)	0.12685 (3)	0.01965 (14)	
Al2	−0.32053 (6)	0.93311 (5)	0.23690 (3)	0.02397 (15)	
Al3	−0.12509 (6)	1.10409 (5)	0.25810 (3)	0.02347 (15)	
Al5	−0.14505 (8)	0.75818 (6)	0.36629 (3)	0.03108 (19)	
Al6	−0.05267 (7)	0.61929 (5)	0.23474 (3)	0.02360 (15)	
O1	−0.12047 (14)	0.91471 (12)	0.18165 (6)	0.0213 (3)	
O2	−0.16407 (14)	0.88908 (13)	0.10334 (6)	0.0244 (4)	
O3	−0.40734 (16)	0.89651 (14)	0.20674 (7)	0.0308 (4)	
O4	−0.22283 (15)	0.97062 (11)	0.26338 (6)	0.0230 (4)	
O5	−0.37951 (14)	1.05623 (12)	0.21895 (7)	0.0264 (4)	
O7	0.08325 (15)	0.84877 (12)	0.20950 (6)	0.0235 (4)	
O8	0.20693 (15)	0.93126 (13)	0.17978 (7)	0.0271 (4)	
O9	−0.03543 (14)	0.98456 (12)	0.09054 (6)	0.0220 (3)	
Dy1	−0.06406 (3)	0.85152 (2)	0.25896 (2)	0.01896 (5)	0.8909 (13)
Al4	0.17617 (9)	0.88390 (7)	0.24152 (4)	0.0194 (2)	0.8909 (13)
O10	0.11224 (18)	0.82265 (14)	0.29460 (8)	0.0237 (4)	0.8909 (13)
N4	0.1743 (2)	0.73691 (16)	0.30418 (9)	0.0272 (5)	0.8909 (13)
H4N	0.155528	0.698368	0.326012	0.033*	0.8909 (13)
O11	0.28111 (17)	0.77344 (14)	0.24617 (8)	0.0240 (4)	0.8909 (13)
C22	0.2627 (2)	0.71501 (19)	0.27934 (11)	0.0246 (5)	0.8909 (13)
C23	0.3418 (3)	0.6297 (2)	0.29142 (12)	0.0319 (7)	0.8909 (13)
C24	0.4349 (3)	0.6135 (2)	0.26406 (15)	0.0405 (8)	0.8909 (13)
H24	0.443194	0.655392	0.237361	0.049*	0.8909 (13)
C25	0.5159 (3)	0.5370 (3)	0.27525 (19)	0.0536 (11)	0.8909 (13)
H25	0.579025	0.526425	0.256472	0.064*	0.8909 (13)
C26	0.5028 (4)	0.4765 (3)	0.3142 (2)	0.0635 (13)	0.8909 (13)
H26	0.558116	0.424587	0.322315	0.076*	0.8909 (13)
C27	0.4119 (4)	0.4899 (3)	0.34124 (18)	0.0579 (12)	0.8909 (13)
H27	0.404578	0.447005	0.367583	0.069*	0.8909 (13)
C28	0.3295 (3)	0.5666 (2)	0.33033 (14)	0.0436 (9)	0.8909 (13)
O12	0.2379 (3)	0.58357 (19)	0.35585 (11)	0.0565 (8)	0.8909 (13)
H12O	0.239906	0.542222	0.378443	0.085*	0.8909 (13)
O25	−0.0351 (2)	0.98802 (17)	0.26394 (12)	0.0220 (6)	0.8909 (13)
N9	0.0679 (2)	0.99014 (18)	0.25926 (10)	0.0221 (5)	0.8909 (13)
O26	−0.0091 (3)	1.13528 (17)	0.26573 (14)	0.0289 (7)	0.8909 (13)
C57	0.0736 (3)	1.0690 (2)	0.26153 (12)	0.0248 (6)	0.8909 (13)
C58	0.1753 (2)	1.0822 (2)	0.25957 (12)	0.0265 (6)	0.8909 (13)
C59	0.1799 (3)	1.1691 (2)	0.25638 (14)	0.0363 (7)	0.8909 (13)
H59	0.118013	1.217033	0.254807	0.044*	0.8909 (13)
C60	0.2725 (3)	1.1860 (3)	0.25549 (16)	0.0428 (8)	0.8909 (13)
H60	0.274637	1.244925	0.253772	0.051*	0.8909 (13)
C61	0.3628 (3)	1.1156 (3)	0.25716 (16)	0.0414 (8)	0.8909 (13)
H61	0.427074	1.126832	0.255943	0.050*	0.8909 (13)
C62	0.3606 (3)	1.0291 (3)	0.26056 (13)	0.0325 (7)	0.8909 (13)
H62	0.423184	0.981903	0.261736	0.039*	0.8909 (13)
C63	0.2671 (2)	1.0112 (2)	0.26228 (11)	0.0258 (6)	0.8909 (13)
O27	0.26772 (17)	0.92654 (16)	0.26688 (8)	0.0246 (4)	0.8909 (13)
N11	0.1047 (2)	0.55147 (18)	0.24520 (11)	0.0267 (5)	0.8909 (13)
C69	0.1312 (3)	0.4816 (2)	0.27959 (13)	0.0367 (7)	0.8909 (13)
H69	0.079212	0.470386	0.302327	0.044*	0.8909 (13)
C70	0.2301 (3)	0.4253 (3)	0.28352 (15)	0.0446 (9)	0.8909 (13)
H70	0.245748	0.377276	0.308779	0.054*	0.8909 (13)
C71	0.3056 (3)	0.4398 (3)	0.25037 (16)	0.0449 (9)	0.8909 (13)
H71	0.373893	0.400809	0.251547	0.054*	0.8909 (13)
C72	0.2803 (3)	0.5118 (3)	0.21554 (15)	0.0468 (9)	0.8909 (13)
H72	0.331147	0.523686	0.192341	0.056*	0.8909 (13)
C73	0.1798 (3)	0.5669 (3)	0.21446 (14)	0.0374 (7)	0.8909 (13)
H73	0.163671	0.617793	0.190905	0.045*	0.8909 (13)
Dy1B	−0.08916 (18)	0.84865 (15)	0.26279 (8)	0.01896 (5)	0.1091 (13)
Al4B	0.1627 (7)	0.8583 (6)	0.2480 (4)	0.0226 (16)	0.1091 (13)
O10B	0.0932 (10)	0.7949 (9)	0.2969 (6)	0.026 (3)	0.1091 (13)
N4B	0.1299 (13)	0.7060 (10)	0.2908 (7)	0.026 (2)	0.1091 (13)
H4B	0.11 (2)	0.661 (11)	0.298 (3)	0.032*	0.1091 (13)
O11B	0.2563 (12)	0.7426 (9)	0.2460 (6)	0.024 (2)	0.1091 (13)
C22B	0.2098 (15)	0.6838 (10)	0.2598 (8)	0.026 (2)	0.1091 (13)
C23B	0.2444 (15)	0.5941 (12)	0.2464 (10)	0.036 (3)	0.1091 (13)
C24B	0.3479 (16)	0.5654 (15)	0.2298 (12)	0.042 (4)	0.1091 (13)
H24B	0.387886	0.604955	0.225820	0.050*	0.1091 (13)
C25B	0.3925 (17)	0.4796 (16)	0.2190 (12)	0.045 (3)	0.1091 (13)
H25B	0.465039	0.454383	0.215597	0.054*	0.1091 (13)
C26B	0.3265 (16)	0.4328 (18)	0.2134 (12)	0.045 (3)	0.1091 (13)
H26B	0.353478	0.381531	0.197853	0.054*	0.1091 (13)
C27B	0.2263 (14)	0.4554 (14)	0.2285 (9)	0.029 (2)	0.1091 (13)
H27B	0.185655	0.417827	0.227067	0.035*	0.1091 (13)
C28B	0.1834 (15)	0.5382 (14)	0.2468 (11)	0.037 (2)	0.1091 (13)
O12B	0.0860 (14)	0.5671 (14)	0.2643 (8)	0.033 (3)	0.1091 (13)
H12P	0.080152	0.540460	0.292030	0.049*	0.1091 (13)
O25B	−0.0424 (15)	0.9735 (12)	0.2722 (12)	0.023 (3)	0.1091 (13)
N9B	0.0633 (12)	0.9664 (10)	0.2682 (10)	0.021 (2)	0.1091 (13)
O26B	−0.0021 (19)	1.1137 (13)	0.2725 (13)	0.024 (3)	0.1091 (13)
C57B	0.0758 (14)	1.0425 (13)	0.2695 (13)	0.025 (3)	0.1091 (13)
C58B	0.1819 (13)	1.0484 (13)	0.2688 (11)	0.026 (2)	0.1091 (13)
C59B	0.1939 (17)	1.1312 (15)	0.2672 (12)	0.034 (3)	0.1091 (13)
H59B	0.134387	1.181389	0.265900	0.040*	0.1091 (13)
C60B	0.2901 (17)	1.1437 (16)	0.2673 (13)	0.038 (3)	0.1091 (13)
H60B	0.298511	1.199831	0.268121	0.045*	0.1091 (13)
C61B	0.3720 (17)	1.0674 (16)	0.2661 (12)	0.035 (3)	0.1091 (13)
H61B	0.439146	1.073390	0.263115	0.041*	0.1091 (13)
C62B	0.3644 (15)	0.9839 (16)	0.2687 (10)	0.030 (3)	0.1091 (13)
H62B	0.424563	0.934309	0.269244	0.036*	0.1091 (13)
C63B	0.2674 (14)	0.9721 (13)	0.2707 (10)	0.024 (2)	0.1091 (13)
O27B	0.2613 (12)	0.8899 (11)	0.2744 (7)	0.024 (2)	0.1091 (13)
O13	−0.10422 (18)	0.86287 (13)	0.34692 (7)	0.0332 (5)	
O14	−0.0310 (2)	0.72887 (15)	0.40760 (7)	0.0387 (5)	
O15	0.0998 (4)	0.9286 (3)	0.40707 (14)	0.0952 (14)	
H15	0.120870	0.966676	0.416271	0.143*	
O16	−0.23392 (16)	0.82229 (13)	0.26932 (6)	0.0260 (4)	
O17	−0.40045 (16)	0.92895 (14)	0.29474 (7)	0.0295 (4)	
O18	−0.2353 (2)	0.79038 (15)	0.41609 (7)	0.0425 (6)	
O19	−0.05625 (16)	0.71810 (12)	0.31273 (7)	0.0272 (4)	
O20	−0.15369 (19)	0.64342 (14)	0.37500 (7)	0.0347 (5)	
O21	−0.07903 (17)	0.51449 (13)	0.25066 (7)	0.0302 (4)	
O22	−0.03514 (16)	0.73167 (12)	0.21481 (6)	0.0264 (4)	
O23	−0.02519 (16)	0.60680 (12)	0.17083 (7)	0.0273 (4)	
O24	0.04193 (15)	0.81789 (12)	0.07843 (6)	0.0232 (4)	
O28	−0.09153 (16)	1.10779 (13)	0.18742 (7)	0.0276 (4)	
H28A	−0.067 (3)	1.147 (2)	0.1732 (13)	0.041*	
H28B	−0.070 (3)	1.0608 (17)	0.1763 (13)	0.041*	
O29	−0.20208 (17)	0.67514 (15)	0.22742 (8)	0.0333 (4)	
H29A	−0.222 (3)	0.7282 (16)	0.2367 (14)	0.050*	
H29B	−0.242 (3)	0.677 (3)	0.2031 (11)	0.050*	
O30	0.4117 (6)	0.4703 (6)	0.0116 (3)	0.172 (3)	
H30A	0.424 (8)	0.479 (4)	0.039 (2)	0.259*	
H30B	0.399 (11)	0.420 (5)	0.021 (4)	0.259*	
N1	−0.22343 (17)	0.91486 (14)	0.17907 (8)	0.0230 (4)	
N2	−0.23575 (17)	1.06082 (14)	0.24987 (8)	0.0228 (4)	
N3	0.07267 (17)	0.89051 (14)	0.16197 (7)	0.0210 (4)	
N5	−0.0277 (2)	0.86239 (18)	0.37434 (9)	0.0372 (6)	
H5N	−0.004792	0.908330	0.371968	0.045*	
N6	−0.2581 (2)	0.81811 (16)	0.32019 (8)	0.0288 (5)	
N7	−0.0751 (2)	0.64288 (14)	0.30061 (8)	0.0269 (5)	
N8	−0.02010 (18)	0.74862 (14)	0.16399 (7)	0.0227 (4)	
C1	−0.2386 (2)	0.90271 (17)	0.13596 (9)	0.0237 (5)	
C2	−0.3413 (2)	0.90484 (19)	0.12457 (10)	0.0264 (5)	
C3	−0.3598 (2)	0.9086 (2)	0.07619 (10)	0.0330 (6)	
H3	−0.307804	0.914234	0.052049	0.040*	
C4	−0.4521 (3)	0.9041 (2)	0.06309 (12)	0.0406 (7)	
H4	−0.464070	0.907265	0.030289	0.049*	
C5	−0.5267 (3)	0.8949 (2)	0.09876 (13)	0.0410 (7)	
H5	−0.589470	0.889764	0.090189	0.049*	
C6	−0.5118 (2)	0.8931 (2)	0.14669 (12)	0.0372 (7)	
H6	−0.564774	0.887660	0.170348	0.045*	
C7	−0.4193 (2)	0.89909 (19)	0.16063 (10)	0.0289 (5)	
C8	−0.3208 (2)	1.10073 (17)	0.22730 (9)	0.0243 (5)	0.8909 (13)
C9	−0.3496 (8)	1.1973 (3)	0.2128 (5)	0.0271 (11)	0.8909 (13)
C10	−0.4397 (3)	1.2377 (3)	0.18800 (14)	0.0331 (10)	0.8909 (13)
H10	−0.477679	1.202138	0.180190	0.040*	0.8909 (13)
C11	−0.4745 (3)	1.3289 (3)	0.17454 (15)	0.0382 (8)	0.8909 (13)
H11	−0.535445	1.355499	0.157664	0.046*	0.8909 (13)
C12	−0.4183 (3)	1.3802 (2)	0.18629 (15)	0.0388 (8)	0.8909 (13)
H12	−0.440925	1.442429	0.177117	0.047*	0.8909 (13)
C13	−0.3300 (3)	1.3420 (2)	0.21116 (14)	0.0342 (8)	0.8909 (13)
H13	−0.293588	1.378667	0.219165	0.041*	0.8909 (13)
C14	−0.2927 (3)	1.2497 (2)	0.22494 (13)	0.0282 (9)	0.8909 (13)
O6	−0.20658 (16)	1.21751 (12)	0.24873 (7)	0.0288 (4)	0.8909 (13)
C8B	−0.3208 (2)	1.10073 (17)	0.22730 (9)	0.0243 (5)	0.1091 (13)
C9B	−0.345 (7)	1.197 (2)	0.210 (5)	0.028 (3)	0.1091 (13)
C10B	−0.424 (3)	1.2359 (17)	0.1786 (14)	0.032 (3)	0.1091 (13)
H10B	−0.463004	1.201005	0.170435	0.038*	0.1091 (13)
C11B	−0.447 (2)	1.3246 (17)	0.1586 (12)	0.037 (3)	0.1091 (13)
H11B	−0.505495	1.351685	0.139827	0.044*	0.1091 (13)
C12B	−0.384 (2)	1.3724 (16)	0.1667 (11)	0.037 (3)	0.1091 (13)
H12B	−0.396658	1.431658	0.151414	0.045*	0.1091 (13)
C13B	−0.303 (2)	1.3358 (15)	0.1964 (11)	0.033 (3)	0.1091 (13)
H13B	−0.262675	1.370822	0.202684	0.039*	0.1091 (13)
C14B	−0.280 (3)	1.2465 (17)	0.2175 (15)	0.030 (3)	0.1091 (13)
O6B	−0.20658 (16)	1.21751 (12)	0.24873 (7)	0.0288 (4)	0.1091 (13)
C15	0.1394 (2)	0.93432 (17)	0.15006 (9)	0.0225 (5)	
C16	0.1344 (2)	0.98972 (17)	0.10306 (9)	0.0230 (5)	
C17	0.2144 (2)	1.0254 (2)	0.08735 (10)	0.0293 (5)	
H17	0.270814	1.012746	0.106961	0.035*	
C18	0.2132 (2)	1.0790 (2)	0.04367 (10)	0.0324 (6)	
H18	0.268152	1.102917	0.033320	0.039*	
C19	0.1304 (2)	1.09728 (19)	0.01524 (10)	0.0287 (5)	
H19	0.129500	1.132995	−0.015057	0.034*	
C20	0.0489 (2)	1.06384 (17)	0.03067 (9)	0.0241 (5)	
H20	−0.006968	1.077114	0.010661	0.029*	
C21	0.0474 (2)	1.01085 (16)	0.07520 (9)	0.0211 (4)	
C29	0.0103 (3)	0.7906 (2)	0.40431 (10)	0.0377 (7)	
C30	0.0998 (3)	0.7804 (3)	0.43219 (12)	0.0470 (9)	
C31	0.1446 (3)	0.6987 (3)	0.45789 (16)	0.0585 (11)	
H31	0.116420	0.651827	0.457610	0.070*	
C32	0.2293 (4)	0.6838 (4)	0.48396 (19)	0.0755 (15)	
H32	0.258194	0.627700	0.502147	0.091*	
C33	0.2718 (4)	0.7511 (4)	0.48339 (18)	0.0757 (16)	
H33	0.330848	0.740793	0.500934	0.091*	
C34	0.2302 (4)	0.8321 (4)	0.45797 (16)	0.0714 (14)	
H34	0.260274	0.877836	0.458010	0.086*	
C35	0.1439 (4)	0.8485 (3)	0.43194 (14)	0.0601 (11)	
C36	−0.3472 (2)	0.87714 (19)	0.32892 (10)	0.0298 (6)	
C37	−0.3902 (3)	0.8842 (2)	0.37895 (11)	0.0365 (7)	
C38	−0.4914 (3)	0.9381 (3)	0.38536 (13)	0.0473 (8)	
H38	−0.528632	0.968986	0.358069	0.057*	
C39	−0.5383 (4)	0.9472 (3)	0.43092 (15)	0.0623 (12)	
H39	−0.607284	0.983744	0.434899	0.075*	
C40	−0.4835 (4)	0.9025 (3)	0.47025 (15)	0.0709 (14)	
H40	−0.515223	0.908129	0.501557	0.085*	
C41	−0.3836 (4)	0.8499 (3)	0.46479 (13)	0.0609 (12)	
H41	−0.347589	0.819759	0.492551	0.073*	
C42	−0.3328 (3)	0.8394 (2)	0.41898 (11)	0.0414 (8)	
C43	−0.1246 (2)	0.60731 (18)	0.33593 (10)	0.0295 (6)	
C44	−0.1489 (2)	0.52571 (19)	0.33205 (10)	0.0314 (6)	
C45	−0.1964 (3)	0.4871 (2)	0.37241 (12)	0.0418 (8)	
H45	−0.211280	0.514128	0.400870	0.050*	
C46	−0.2220 (3)	0.4108 (2)	0.37158 (14)	0.0507 (9)	
H46	−0.252996	0.385131	0.399314	0.061*	
C47	−0.2017 (3)	0.3721 (2)	0.32973 (14)	0.0461 (8)	
H47	−0.220708	0.320520	0.328653	0.055*	
C48	−0.1544 (3)	0.4077 (2)	0.28975 (12)	0.0354 (6)	
H48	−0.140322	0.379598	0.261671	0.042*	
C49	−0.1264 (2)	0.48500 (18)	0.28958 (10)	0.0297 (6)	
C50	−0.0139 (2)	0.67809 (17)	0.14478 (9)	0.0224 (5)	
C51	0.0126 (2)	0.67607 (18)	0.09317 (9)	0.0256 (5)	
C52	0.0151 (3)	0.6000 (2)	0.07450 (12)	0.0421 (8)	
H52	−0.010704	0.556783	0.093941	0.051*	
C53	0.0544 (4)	0.5863 (2)	0.02832 (12)	0.0525 (10)	
H53	0.055901	0.534276	0.016151	0.063*	
C54	0.0916 (3)	0.6500 (2)	0.00019 (11)	0.0412 (8)	
H54	0.121214	0.640569	−0.031122	0.049*	
C55	0.0859 (2)	0.72695 (19)	0.01732 (10)	0.0300 (6)	
H55	0.110325	0.770332	−0.002853	0.036*	
C56	0.0449 (2)	0.74309 (17)	0.06386 (9)	0.0230 (5)	
N10	−0.1602 (2)	1.09471 (17)	0.33315 (9)	0.0379 (6)	
C64	−0.1216 (4)	1.1366 (3)	0.36016 (14)	0.0545 (10)	
H64	−0.075731	1.167981	0.345401	0.065*	
C65	−0.1467 (5)	1.1356 (4)	0.40926 (16)	0.0823 (18)	
H65	−0.119752	1.167107	0.427457	0.099*	
C66	−0.2107 (7)	1.0885 (4)	0.43088 (15)	0.106 (3)	
H66	−0.227154	1.086015	0.464437	0.127*	
C67	−0.2507 (6)	1.0452 (3)	0.40411 (15)	0.091 (2)	
H67	−0.295694	1.012661	0.418483	0.109*	
C68	−0.2238 (4)	1.0501 (2)	0.35516 (12)	0.0548 (11)	
H68	−0.251967	1.020351	0.336380	0.066*	
N12	−0.2035 (2)	1.1056 (2)	0.06021 (11)	0.0452 (7)	
H12A	−0.156488	1.055423	0.069355	0.054*	
C74	−0.2336 (3)	1.1667 (3)	0.08923 (14)	0.0569 (11)	
H74	−0.206445	1.154671	0.120012	0.068*	
C75	−0.3029 (3)	1.2464 (3)	0.07556 (17)	0.0593 (11)	
H75	−0.322806	1.290643	0.096218	0.071*	
C76	−0.3439 (3)	1.2623 (3)	0.03143 (17)	0.0612 (12)	
H76	−0.392308	1.317615	0.021200	0.073*	
C77	−0.3136 (3)	1.1964 (3)	0.00215 (15)	0.0616 (12)	
H77	−0.342125	1.205785	−0.028164	0.074*	
C78	−0.2425 (3)	1.1180 (3)	0.01721 (16)	0.0519 (9)	
H78	−0.220822	1.072499	−0.002652	0.062*	
N13	−0.0132 (2)	1.23443 (18)	0.14860 (10)	0.0380 (6)	
C79	−0.0246 (3)	1.2955 (2)	0.17715 (13)	0.0433 (8)	
H79	−0.062509	1.290737	0.207003	0.052*	
C80	0.0156 (3)	1.3648 (2)	0.16578 (15)	0.0498 (9)	
H80	0.006224	1.406935	0.187134	0.060*	
C81	0.0693 (4)	1.3711 (3)	0.12281 (18)	0.0635 (12)	
H81	0.099415	1.417440	0.113916	0.076*	
C82	0.0799 (4)	1.3095 (3)	0.09202 (17)	0.0673 (13)	
H82	0.115892	1.313795	0.061594	0.081*	
C83	0.0372 (3)	1.2423 (3)	0.10656 (13)	0.0477 (8)	
H83	0.044095	1.199916	0.085629	0.057*	
N14	0.148 (2)	1.0717 (12)	0.4240 (6)	0.081 (2)	0.509 (8)
C84	0.134 (2)	1.1063 (13)	0.4653 (6)	0.084 (3)	0.509 (8)
H84	0.134387	1.069012	0.495000	0.101*	0.509 (8)
C85	0.1193 (13)	1.1941 (10)	0.4651 (4)	0.093 (3)	0.509 (8)
H85	0.112993	1.216830	0.494624	0.111*	0.509 (8)
C86	0.1136 (14)	1.2493 (9)	0.4233 (4)	0.105 (3)	0.509 (8)
H86	0.104635	1.310193	0.423685	0.126*	0.509 (8)
C87	0.1207 (13)	1.2179 (9)	0.3801 (4)	0.102 (3)	0.509 (8)
H87	0.110672	1.255644	0.350347	0.122*	0.509 (8)
C88	0.1436 (13)	1.1276 (9)	0.3837 (5)	0.092 (3)	0.509 (8)
H88	0.157339	1.102906	0.354525	0.111*	0.509 (8)
N22	0.138 (2)	1.0610 (14)	0.4291 (7)	0.087 (3)	0.491 (8)
C124	0.130 (3)	1.0922 (14)	0.4709 (7)	0.086 (3)	0.491 (8)
H124	0.099501	1.064343	0.498088	0.104*	0.491 (8)
C125	0.1621 (12)	1.1613 (10)	0.4767 (4)	0.089 (2)	0.491 (8)
H125	0.160193	1.178437	0.507432	0.107*	0.491 (8)
C126	0.1976 (14)	1.2053 (10)	0.4363 (4)	0.104 (3)	0.491 (8)
H126	0.222465	1.253132	0.438819	0.125*	0.491 (8)
C127	0.1972 (14)	1.1797 (10)	0.3915 (4)	0.101 (3)	0.491 (8)
H127	0.208933	1.215423	0.362562	0.121*	0.491 (8)
C128	0.1792 (14)	1.1015 (10)	0.3911 (6)	0.098 (3)	0.491 (8)
H128	0.196817	1.074553	0.362584	0.117*	0.491 (8)
N15	0.1864 (4)	0.4704 (3)	0.42276 (15)	0.0635 (10)	0.539 (3)
C89	0.2339 (10)	0.4308 (9)	0.4621 (4)	0.065 (2)	0.539 (3)
H89	0.301090	0.434758	0.463689	0.078*	0.539 (3)
C90	0.1974 (9)	0.3850 (14)	0.5004 (5)	0.073 (2)	0.539 (3)
H90	0.236658	0.357211	0.527562	0.088*	0.539 (3)
C91	0.0976 (9)	0.3814 (7)	0.4973 (4)	0.083 (2)	0.539 (3)
H91	0.066370	0.351060	0.523027	0.100*	0.539 (3)
C92	0.0437 (9)	0.4231 (8)	0.4558 (4)	0.088 (2)	0.539 (3)
H92	−0.022847	0.419205	0.452479	0.106*	0.539 (3)
C93	0.0894 (8)	0.4689 (7)	0.4209 (4)	0.082 (2)	0.539 (3)
H93	0.051428	0.501162	0.394027	0.098*	0.539 (3)
N23	0.1864 (4)	0.4704 (3)	0.42276 (15)	0.0635 (10)	0.352 (3)
C129	0.2305 (14)	0.4160 (14)	0.4603 (6)	0.064 (3)	0.352 (3)
H129	0.303354	0.392653	0.458314	0.077*	0.352 (3)
C130	0.1800 (13)	0.391 (2)	0.5013 (8)	0.070 (2)	0.352 (3)
H130	0.215557	0.350329	0.526724	0.084*	0.352 (3)
C131	0.0750 (12)	0.4272 (12)	0.5043 (5)	0.085 (3)	0.352 (3)
H131	0.036619	0.416741	0.533343	0.101*	0.352 (3)
C132	0.0251 (11)	0.4796 (12)	0.4642 (5)	0.085 (3)	0.352 (3)
H132	−0.047819	0.500315	0.464351	0.101*	0.352 (3)
C133	0.0842 (9)	0.5003 (10)	0.4248 (6)	0.073 (2)	0.352 (3)
H133	0.050898	0.537454	0.397823	0.088*	0.352 (3)
N25	0.095 (3)	0.435 (2)	0.3624 (8)	0.086 (5)	0.1091 (13)
C139	0.158 (2)	0.449 (2)	0.3934 (11)	0.077 (3)	0.1091 (13)
H139	0.203043	0.483897	0.381725	0.092*	0.1091 (13)
C140	0.157 (3)	0.413 (2)	0.4414 (10)	0.076 (2)	0.1091 (13)
H140	0.199878	0.423186	0.462642	0.091*	0.1091 (13)
C141	0.091 (3)	0.363 (2)	0.4585 (8)	0.083 (3)	0.1091 (13)
H141	0.090065	0.338254	0.491345	0.100*	0.1091 (13)
C142	0.028 (3)	0.349 (2)	0.4275 (12)	0.090 (5)	0.1091 (13)
H142	−0.016583	0.314033	0.439131	0.108*	0.1091 (13)
C143	0.030 (3)	0.385 (2)	0.3794 (11)	0.092 (6)	0.1091 (13)
H143	−0.013419	0.374743	0.358214	0.110*	0.1091 (13)
N16	0.4229 (2)	0.80658 (19)	0.30999 (10)	0.0398 (6)	
H16	0.377471	0.849042	0.292992	0.048*	
C94	0.5182 (3)	0.7726 (2)	0.28991 (13)	0.0420 (7)	
H94	0.535523	0.794825	0.258004	0.050*	
C95	0.5890 (3)	0.7067 (3)	0.31545 (15)	0.0485 (8)	
H95	0.655848	0.681599	0.301529	0.058*	
C96	0.5622 (4)	0.6766 (3)	0.36225 (17)	0.0687 (14)	
H96	0.611344	0.631296	0.380823	0.082*	
C97	0.4645 (4)	0.7123 (4)	0.38184 (17)	0.0766 (16)	
H97	0.445461	0.691695	0.413778	0.092*	
C98	0.3956 (3)	0.7779 (3)	0.35459 (14)	0.0548 (10)	
H98	0.327894	0.803064	0.367544	0.066*	
N17	0.4686 (9)	1.2708 (9)	0.4142 (5)	0.080 (3)	0.509 (8)
C99	0.5553 (14)	1.2683 (16)	0.3892 (6)	0.070 (2)	0.509 (8)
H99	0.600624	1.294737	0.399730	0.084*	0.509 (8)
C100	0.5825 (13)	1.2285 (11)	0.3482 (5)	0.076 (3)	0.509 (8)
H100	0.644058	1.229940	0.329788	0.091*	0.509 (8)
C101	0.5202 (12)	1.1877 (10)	0.3348 (5)	0.077 (3)	0.509 (8)
H101	0.542564	1.150437	0.310487	0.092*	0.509 (8)
C102	0.4210 (10)	1.2012 (10)	0.3577 (5)	0.082 (3)	0.509 (8)
H102	0.368992	1.186348	0.344326	0.098*	0.509 (8)
C103	0.4024 (10)	1.2363 (9)	0.3998 (4)	0.077 (3)	0.509 (8)
H103	0.341343	1.236153	0.418990	0.093*	0.509 (8)
N24	0.5057 (10)	1.2543 (10)	0.4177 (5)	0.080 (2)	0.491 (8)
C134	0.5745 (16)	1.2693 (17)	0.3844 (6)	0.069 (2)	0.491 (8)
H134	0.622138	1.296232	0.392124	0.083*	0.491 (8)
C135	0.5810 (14)	1.2483 (11)	0.3396 (5)	0.073 (3)	0.491 (8)
H135	0.634010	1.258474	0.317239	0.088*	0.491 (8)
C136	0.5129 (13)	1.2131 (10)	0.3267 (5)	0.072 (3)	0.491 (8)
H136	0.503829	1.212955	0.293845	0.086*	0.491 (8)
C137	0.4551 (11)	1.1763 (10)	0.3638 (5)	0.082 (3)	0.491 (8)
H137	0.425441	1.132296	0.359418	0.098*	0.491 (8)
C138	0.4451 (12)	1.2087 (10)	0.4066 (5)	0.084 (3)	0.491 (8)
H138	0.393075	1.198686	0.429702	0.101*	0.491 (8)
N18	−0.2545 (3)	0.6330 (2)	0.14700 (13)	0.0539 (8)	
C104	−0.2314 (4)	0.5449 (3)	0.15067 (18)	0.0614 (11)	
H104	−0.215220	0.511332	0.181258	0.074*	
C105	−0.2296 (4)	0.5003 (3)	0.11344 (18)	0.0653 (12)	
H105	−0.213921	0.437723	0.118445	0.078*	
C106	−0.2505 (4)	0.5462 (3)	0.06910 (18)	0.0661 (13)	
H106	−0.249154	0.516737	0.042440	0.079*	
C107	−0.2744 (5)	0.6392 (4)	0.06374 (19)	0.0862 (18)	
H107	−0.289999	0.674611	0.033560	0.103*	
C108	−0.2739 (5)	0.6756 (3)	0.1038 (2)	0.0816 (17)	
H108	−0.289157	0.738086	0.100083	0.098*	
N19	0.3811 (3)	0.5770 (3)	0.08504 (15)	0.0661 (10)	
C109	0.4599 (3)	0.5957 (3)	0.09723 (17)	0.0642 (12)	
H109	0.526337	0.560233	0.087589	0.077*	
C110	0.4548 (4)	0.6627 (4)	0.12290 (18)	0.0737 (14)	
H110	0.515056	0.671696	0.132006	0.088*	
C111	0.3531 (4)	0.7188 (3)	0.13536 (14)	0.0553 (10)	
H111	0.342740	0.767370	0.152405	0.066*	
C112	0.2732 (3)	0.6976 (3)	0.12117 (15)	0.0506 (9)	
H112	0.204794	0.732656	0.128068	0.061*	
C113	0.2898 (4)	0.6268 (3)	0.09716 (17)	0.0599 (11)	
H113	0.231711	0.613234	0.088889	0.072*	
N20	0.3890 (5)	0.2927 (5)	0.0567 (2)	0.1086 (19)	
C114	0.4374 (6)	0.2077 (7)	0.0515 (3)	0.115 (3)	
H114	0.476408	0.194395	0.022431	0.138*	
C115	0.4324 (6)	0.1384 (6)	0.0875 (4)	0.117 (3)	
H115	0.468351	0.078821	0.083334	0.140*	
C116	0.3751 (6)	0.1582 (5)	0.1283 (3)	0.099 (2)	
H116	0.369261	0.112901	0.153580	0.118*	
C117	0.3261 (6)	0.2447 (7)	0.1323 (3)	0.117 (3)	
H117	0.284153	0.260071	0.160412	0.141*	
C118	0.3364 (6)	0.3092 (6)	0.0966 (3)	0.107 (2)	
H118	0.303567	0.369102	0.101102	0.128*	
N21	0.448 (2)	0.4470 (17)	0.5236 (6)	0.074 (4)	0.429 (4)
C119	0.491 (2)	0.4440 (15)	0.4770 (6)	0.062 (3)	0.429 (4)
H119	0.484419	0.401249	0.458967	0.074*	0.429 (4)
C120	0.543 (2)	0.5037 (15)	0.4568 (6)	0.057 (3)	0.429 (4)
H120	0.572384	0.501731	0.424925	0.068*	0.429 (4)
C121	0.552 (2)	0.5663 (17)	0.4832 (7)	0.073 (5)	0.429 (4)
H121	0.587729	0.607135	0.469372	0.088*	0.429 (4)
C122	0.509 (2)	0.5693 (18)	0.5298 (7)	0.082 (5)	0.429 (4)
H122	0.515110	0.612059	0.547861	0.099*	0.429 (4)
C123	0.457 (2)	0.5096 (19)	0.5500 (6)	0.083 (5)	0.429 (4)
H123	0.427144	0.511577	0.581904	0.100*	0.429 (4)

### Bis(pyridinium) diaquadipyridinehexakis[µ3-salicylhydroximato(3-)]bis[µ2-salicylhydroximato(1-)]hexaaluminiumdysprosium–pyridine–water (1/7.429/1) (2) Atomic displacement parameters (Å^2^)

**Table:**

	*U*^11^	*U*^22^	*U*^33^	*U*^12^	*U*^13^	*U*^23^
Al1	0.0229 (4)	0.0193 (3)	0.0159 (3)	−0.0044 (3)	−0.0019 (3)	−0.0037 (3)
Al2	0.0259 (4)	0.0250 (4)	0.0215 (4)	−0.0088 (3)	0.0011 (3)	−0.0039 (3)
Al3	0.0259 (4)	0.0200 (3)	0.0243 (4)	−0.0039 (3)	−0.0050 (3)	−0.0065 (3)
Al5	0.0489 (5)	0.0256 (4)	0.0172 (4)	−0.0106 (4)	0.0010 (3)	−0.0020 (3)
Al6	0.0296 (4)	0.0213 (3)	0.0222 (4)	−0.0118 (3)	0.0045 (3)	−0.0052 (3)
O1	0.0225 (8)	0.0222 (8)	0.0178 (8)	−0.0043 (7)	−0.0017 (6)	−0.0037 (6)
O2	0.0240 (9)	0.0313 (9)	0.0176 (8)	−0.0077 (7)	−0.0017 (7)	−0.0040 (7)
O3	0.0319 (10)	0.0410 (11)	0.0251 (9)	−0.0193 (9)	0.0022 (8)	−0.0060 (8)
O4	0.0283 (9)	0.0187 (8)	0.0210 (8)	−0.0059 (7)	0.0003 (7)	−0.0030 (6)
O5	0.0224 (9)	0.0253 (9)	0.0314 (10)	−0.0074 (7)	−0.0015 (7)	−0.0034 (7)
O7	0.0299 (9)	0.0230 (8)	0.0158 (8)	−0.0050 (7)	−0.0061 (7)	−0.0010 (6)
O8	0.0271 (10)	0.0310 (10)	0.0233 (9)	−0.0073 (8)	−0.0083 (7)	−0.0030 (7)
O9	0.0215 (8)	0.0213 (8)	0.0225 (8)	−0.0060 (7)	−0.0040 (7)	−0.0009 (6)
Dy1	0.02342 (12)	0.01765 (6)	0.01528 (6)	−0.00596 (7)	0.00056 (7)	−0.00239 (4)
Al4	0.0226 (5)	0.0175 (5)	0.0182 (5)	−0.0056 (4)	−0.0049 (3)	−0.0010 (4)
O10	0.0296 (11)	0.0191 (10)	0.0198 (9)	−0.0038 (8)	−0.0038 (8)	−0.0007 (8)
N4	0.0358 (14)	0.0184 (11)	0.0240 (11)	−0.0061 (10)	−0.0029 (10)	0.0029 (9)
O11	0.0276 (11)	0.0184 (9)	0.0248 (10)	−0.0059 (8)	−0.0036 (8)	0.0002 (8)
C22	0.0293 (14)	0.0195 (12)	0.0242 (13)	−0.0051 (10)	−0.0073 (11)	−0.0016 (10)
C23	0.0366 (16)	0.0203 (13)	0.0354 (16)	−0.0038 (12)	−0.0097 (13)	0.0006 (11)
C24	0.0384 (19)	0.0245 (15)	0.054 (2)	−0.0022 (13)	−0.0089 (16)	−0.0041 (14)
C25	0.042 (2)	0.0283 (17)	0.082 (3)	0.0031 (16)	−0.012 (2)	−0.0063 (18)
C26	0.058 (3)	0.0316 (19)	0.088 (3)	0.0076 (18)	−0.028 (2)	0.002 (2)
C27	0.070 (3)	0.0283 (18)	0.063 (3)	−0.0007 (19)	−0.022 (2)	0.0120 (18)
C28	0.057 (2)	0.0262 (16)	0.0412 (19)	−0.0045 (15)	−0.0126 (17)	0.0053 (14)
O12	0.074 (2)	0.0358 (15)	0.0430 (16)	−0.0067 (14)	0.0039 (15)	0.0167 (12)
O25	0.0222 (10)	0.0191 (11)	0.0251 (17)	−0.0056 (8)	−0.0034 (9)	−0.0046 (10)
N9	0.0234 (11)	0.0191 (12)	0.0240 (14)	−0.0054 (9)	−0.0064 (9)	−0.0029 (10)
O26	0.0330 (13)	0.0179 (12)	0.0361 (17)	−0.0038 (11)	−0.0108 (11)	−0.0067 (12)
C57	0.0304 (14)	0.0197 (13)	0.0245 (15)	−0.0056 (12)	−0.0079 (11)	−0.0039 (12)
C58	0.0303 (14)	0.0214 (14)	0.0307 (15)	−0.0096 (12)	−0.0096 (11)	−0.0031 (11)
C59	0.0397 (18)	0.0232 (15)	0.049 (2)	−0.0105 (13)	−0.0122 (15)	−0.0055 (14)
C60	0.0437 (19)	0.0293 (17)	0.063 (2)	−0.0176 (15)	−0.0127 (17)	−0.0074 (16)
C61	0.0374 (18)	0.0349 (18)	0.060 (2)	−0.0190 (15)	−0.0073 (16)	−0.0093 (16)
C62	0.0309 (15)	0.0297 (16)	0.0405 (17)	−0.0115 (13)	−0.0080 (13)	−0.0063 (14)
C63	0.0314 (14)	0.0233 (13)	0.0261 (13)	−0.0111 (12)	−0.0070 (11)	−0.0039 (11)
O27	0.0284 (10)	0.0212 (11)	0.0257 (10)	−0.0087 (9)	−0.0085 (8)	−0.0008 (9)
N11	0.0266 (13)	0.0246 (12)	0.0285 (13)	−0.0074 (10)	0.0009 (11)	−0.0048 (10)
C69	0.0364 (17)	0.0317 (16)	0.0375 (17)	−0.0078 (13)	0.0005 (14)	0.0013 (13)
C70	0.043 (2)	0.0328 (17)	0.048 (2)	0.0002 (15)	−0.0053 (16)	0.0021 (15)
C71	0.0320 (17)	0.0438 (19)	0.051 (2)	0.0018 (15)	−0.0044 (15)	−0.0102 (16)
C72	0.0287 (16)	0.059 (2)	0.0470 (19)	−0.0094 (15)	0.0045 (14)	−0.0029 (17)
C73	0.0291 (15)	0.0407 (17)	0.0387 (17)	−0.0101 (13)	0.0007 (13)	0.0026 (14)
Dy1B	0.02342 (12)	0.01765 (6)	0.01528 (6)	−0.00596 (7)	0.00056 (7)	−0.00239 (4)
Al4B	0.026 (3)	0.021 (3)	0.023 (3)	−0.008 (3)	−0.009 (2)	−0.002 (3)
O10B	0.031 (5)	0.020 (5)	0.025 (5)	−0.004 (4)	−0.006 (4)	−0.001 (4)
N4B	0.031 (4)	0.021 (4)	0.027 (4)	−0.006 (4)	−0.008 (4)	−0.002 (4)
O11B	0.026 (4)	0.019 (4)	0.025 (4)	−0.006 (4)	−0.003 (4)	−0.002 (4)
C22B	0.027 (4)	0.023 (4)	0.029 (4)	−0.010 (4)	−0.009 (4)	0.001 (4)
C23B	0.030 (5)	0.038 (5)	0.040 (5)	−0.012 (4)	0.001 (4)	−0.003 (4)
C24B	0.030 (6)	0.044 (6)	0.049 (6)	−0.009 (6)	0.003 (6)	−0.006 (6)
C25B	0.031 (5)	0.048 (5)	0.052 (5)	−0.007 (5)	0.000 (5)	−0.008 (5)
C26B	0.031 (5)	0.048 (5)	0.050 (5)	−0.004 (5)	−0.001 (5)	−0.006 (5)
C27B	0.018 (3)	0.036 (4)	0.038 (4)	−0.015 (3)	−0.009 (3)	−0.004 (3)
C28B	0.032 (3)	0.036 (3)	0.040 (4)	−0.008 (3)	0.000 (3)	−0.002 (3)
O12B	0.032 (5)	0.029 (5)	0.037 (5)	−0.011 (4)	0.003 (5)	0.000 (5)
O25B	0.024 (5)	0.021 (5)	0.021 (6)	−0.003 (5)	−0.006 (5)	0.001 (5)
N9B	0.025 (4)	0.019 (4)	0.022 (4)	−0.007 (4)	−0.008 (4)	−0.003 (4)
O26B	0.028 (5)	0.018 (5)	0.027 (5)	−0.005 (4)	−0.012 (4)	−0.006 (5)
C57B	0.028 (4)	0.020 (4)	0.028 (4)	−0.004 (4)	−0.007 (4)	−0.005 (4)
C58B	0.032 (4)	0.020 (4)	0.031 (4)	−0.011 (4)	−0.009 (3)	−0.005 (4)
C59B	0.035 (4)	0.025 (4)	0.042 (4)	−0.010 (4)	−0.007 (4)	−0.003 (4)
C60B	0.038 (4)	0.028 (4)	0.051 (4)	−0.014 (4)	−0.008 (4)	−0.005 (4)
C61B	0.035 (4)	0.027 (4)	0.046 (4)	−0.014 (4)	−0.008 (4)	−0.005 (4)
C62B	0.032 (4)	0.027 (4)	0.034 (4)	−0.010 (4)	−0.007 (4)	−0.003 (4)
C63B	0.027 (4)	0.022 (4)	0.029 (4)	−0.012 (4)	−0.010 (4)	−0.006 (4)
O27B	0.026 (4)	0.023 (4)	0.026 (4)	−0.009 (4)	−0.009 (4)	−0.003 (4)
O13	0.0540 (14)	0.0283 (10)	0.0184 (9)	−0.0126 (9)	−0.0037 (8)	−0.0047 (7)
O14	0.0556 (15)	0.0348 (11)	0.0238 (10)	−0.0107 (10)	−0.0061 (9)	−0.0025 (8)
O15	0.157 (4)	0.089 (3)	0.070 (2)	−0.075 (3)	−0.060 (3)	0.013 (2)
O16	0.0349 (10)	0.0275 (9)	0.0164 (8)	−0.0118 (8)	0.0035 (7)	−0.0042 (7)
O17	0.0310 (10)	0.0341 (10)	0.0246 (9)	−0.0128 (8)	0.0038 (8)	−0.0049 (8)
O18	0.0626 (16)	0.0360 (12)	0.0188 (9)	−0.0037 (11)	0.0057 (9)	−0.0026 (8)
O19	0.0420 (11)	0.0197 (8)	0.0206 (8)	−0.0103 (8)	−0.0015 (8)	−0.0030 (7)
O20	0.0550 (14)	0.0281 (10)	0.0196 (9)	−0.0138 (10)	0.0046 (9)	−0.0013 (7)
O21	0.0451 (12)	0.0223 (9)	0.0267 (9)	−0.0170 (9)	0.0062 (8)	−0.0043 (7)
O22	0.0415 (11)	0.0204 (8)	0.0157 (8)	−0.0080 (8)	0.0017 (7)	−0.0026 (6)
O23	0.0381 (11)	0.0240 (9)	0.0245 (9)	−0.0163 (8)	0.0059 (8)	−0.0076 (7)
O24	0.0294 (9)	0.0207 (8)	0.0188 (8)	−0.0062 (7)	0.0011 (7)	−0.0048 (6)
O28	0.0317 (10)	0.0255 (9)	0.0267 (9)	−0.0091 (8)	−0.0009 (8)	−0.0069 (7)
O29	0.0331 (11)	0.0326 (11)	0.0359 (11)	−0.0108 (9)	−0.0012 (9)	−0.0081 (9)
O30	0.163 (6)	0.223 (8)	0.168 (6)	−0.066 (6)	0.019 (5)	−0.135 (6)
N1	0.0237 (10)	0.0237 (10)	0.0219 (10)	−0.0077 (8)	−0.0012 (8)	−0.0029 (8)
N2	0.0247 (10)	0.0182 (9)	0.0236 (10)	−0.0035 (8)	−0.0003 (8)	−0.0042 (8)
N3	0.0247 (10)	0.0203 (9)	0.0159 (9)	−0.0029 (8)	−0.0039 (7)	−0.0020 (7)
N5	0.0583 (18)	0.0356 (13)	0.0209 (11)	−0.0168 (13)	−0.0029 (11)	−0.0077 (10)
N6	0.0422 (14)	0.0279 (11)	0.0174 (10)	−0.0137 (10)	0.0029 (9)	−0.0026 (8)
N7	0.0409 (13)	0.0193 (10)	0.0210 (10)	−0.0105 (9)	−0.0007 (9)	−0.0023 (8)
N8	0.0305 (11)	0.0222 (10)	0.0149 (9)	−0.0073 (8)	−0.0005 (8)	−0.0031 (7)
C1	0.0278 (13)	0.0221 (11)	0.0205 (11)	−0.0073 (10)	−0.0034 (9)	−0.0006 (9)
C2	0.0254 (13)	0.0304 (13)	0.0236 (12)	−0.0082 (10)	−0.0031 (10)	−0.0037 (10)
C3	0.0295 (14)	0.0434 (16)	0.0252 (13)	−0.0088 (12)	−0.0050 (11)	−0.0045 (12)
C4	0.0333 (16)	0.055 (2)	0.0343 (16)	−0.0104 (14)	−0.0088 (13)	−0.0112 (14)
C5	0.0291 (15)	0.053 (2)	0.0451 (18)	−0.0129 (14)	−0.0070 (13)	−0.0139 (15)
C6	0.0301 (15)	0.0477 (18)	0.0386 (16)	−0.0171 (13)	−0.0002 (12)	−0.0100 (14)
C7	0.0289 (14)	0.0319 (14)	0.0287 (13)	−0.0127 (11)	−0.0012 (10)	−0.0055 (11)
C8	0.0215 (11)	0.0243 (11)	0.0238 (11)	−0.0029 (9)	0.0014 (9)	−0.0038 (9)
C9	0.0243 (17)	0.0245 (13)	0.028 (2)	−0.0010 (11)	−0.0005 (17)	−0.0035 (12)
C10	0.0283 (18)	0.0311 (15)	0.035 (2)	−0.0016 (13)	−0.0024 (15)	−0.0048 (14)
C11	0.0339 (18)	0.0334 (17)	0.0392 (19)	0.0017 (14)	−0.0087 (14)	−0.0021 (15)
C12	0.0408 (19)	0.0231 (14)	0.0431 (19)	0.0034 (13)	−0.0073 (15)	−0.0007 (14)
C13	0.0392 (19)	0.0214 (14)	0.0377 (18)	−0.0028 (13)	−0.0031 (14)	−0.0037 (13)
C14	0.0314 (17)	0.0219 (13)	0.0262 (17)	0.0003 (12)	−0.0007 (14)	−0.0060 (11)
O6	0.0311 (10)	0.0205 (8)	0.0338 (10)	−0.0033 (7)	−0.0072 (8)	−0.0065 (7)
C8B	0.0215 (11)	0.0243 (11)	0.0238 (11)	−0.0029 (9)	0.0014 (9)	−0.0038 (9)
C9B	0.026 (4)	0.025 (4)	0.029 (4)	−0.001 (4)	−0.001 (4)	−0.004 (4)
C10B	0.029 (5)	0.028 (5)	0.034 (5)	−0.001 (4)	−0.004 (5)	−0.005 (4)
C11B	0.033 (5)	0.030 (5)	0.040 (5)	0.000 (4)	−0.008 (5)	−0.002 (5)
C12B	0.037 (5)	0.026 (4)	0.043 (5)	−0.002 (4)	−0.006 (5)	−0.002 (5)
C13B	0.036 (5)	0.023 (4)	0.037 (5)	−0.003 (4)	−0.005 (5)	−0.005 (4)
C14B	0.032 (4)	0.022 (4)	0.032 (4)	0.000 (4)	−0.004 (4)	−0.006 (4)
O6B	0.0311 (10)	0.0205 (8)	0.0338 (10)	−0.0033 (7)	−0.0072 (8)	−0.0065 (7)
C15	0.0218 (11)	0.0227 (11)	0.0213 (11)	−0.0027 (9)	−0.0034 (9)	−0.0051 (9)
C16	0.0243 (12)	0.0241 (11)	0.0205 (11)	−0.0056 (9)	−0.0029 (9)	−0.0049 (9)
C17	0.0262 (13)	0.0366 (14)	0.0270 (13)	−0.0118 (11)	−0.0049 (10)	−0.0032 (11)
C18	0.0318 (15)	0.0422 (16)	0.0277 (13)	−0.0194 (13)	−0.0004 (11)	−0.0019 (12)
C19	0.0335 (14)	0.0324 (14)	0.0219 (12)	−0.0136 (11)	−0.0008 (10)	−0.0019 (10)
C20	0.0280 (13)	0.0251 (12)	0.0199 (11)	−0.0076 (10)	−0.0040 (9)	−0.0043 (9)
C21	0.0246 (12)	0.0188 (10)	0.0199 (11)	−0.0049 (9)	−0.0032 (9)	−0.0052 (8)
C29	0.053 (2)	0.0406 (16)	0.0195 (12)	−0.0122 (15)	0.0007 (12)	−0.0095 (11)
C30	0.056 (2)	0.059 (2)	0.0245 (14)	−0.0115 (18)	−0.0025 (14)	−0.0135 (14)
C31	0.054 (2)	0.063 (3)	0.055 (2)	−0.005 (2)	−0.0120 (19)	−0.017 (2)
C32	0.069 (3)	0.081 (3)	0.067 (3)	0.002 (3)	−0.026 (2)	−0.020 (3)
C33	0.059 (3)	0.110 (4)	0.062 (3)	−0.011 (3)	−0.013 (2)	−0.043 (3)
C34	0.083 (3)	0.103 (4)	0.045 (2)	−0.041 (3)	−0.008 (2)	−0.028 (3)
C35	0.082 (3)	0.076 (3)	0.0321 (18)	−0.033 (3)	−0.0094 (19)	−0.0145 (18)
C36	0.0396 (16)	0.0312 (13)	0.0226 (12)	−0.0177 (12)	0.0060 (11)	−0.0059 (10)
C37	0.0527 (19)	0.0340 (15)	0.0245 (13)	−0.0172 (14)	0.0115 (13)	−0.0092 (11)
C38	0.052 (2)	0.052 (2)	0.0352 (17)	−0.0122 (17)	0.0099 (15)	−0.0132 (15)
C39	0.060 (3)	0.074 (3)	0.042 (2)	−0.008 (2)	0.0188 (19)	−0.016 (2)
C40	0.088 (3)	0.072 (3)	0.0328 (19)	−0.003 (3)	0.025 (2)	−0.0106 (19)
C41	0.086 (3)	0.055 (2)	0.0259 (16)	−0.005 (2)	0.0157 (18)	−0.0043 (15)
C42	0.066 (2)	0.0315 (15)	0.0238 (13)	−0.0132 (15)	0.0105 (14)	−0.0059 (11)
C43	0.0406 (16)	0.0241 (12)	0.0218 (12)	−0.0088 (11)	0.0004 (11)	−0.0009 (10)
C44	0.0405 (16)	0.0249 (13)	0.0270 (13)	−0.0117 (12)	0.0041 (11)	0.0013 (10)
C45	0.056 (2)	0.0335 (16)	0.0336 (16)	−0.0170 (15)	0.0113 (14)	−0.0010 (12)
C46	0.065 (2)	0.0398 (18)	0.046 (2)	−0.0255 (18)	0.0178 (18)	0.0027 (15)
C47	0.055 (2)	0.0316 (16)	0.054 (2)	−0.0228 (15)	0.0126 (17)	−0.0040 (14)
C48	0.0420 (17)	0.0277 (14)	0.0400 (16)	−0.0177 (13)	0.0070 (13)	−0.0063 (12)
C49	0.0366 (15)	0.0232 (12)	0.0291 (13)	−0.0116 (11)	0.0031 (11)	−0.0011 (10)
C50	0.0235 (12)	0.0247 (11)	0.0213 (11)	−0.0100 (9)	0.0008 (9)	−0.0056 (9)
C51	0.0319 (14)	0.0284 (13)	0.0213 (11)	−0.0144 (11)	0.0028 (10)	−0.0093 (10)
C52	0.069 (2)	0.0378 (16)	0.0302 (15)	−0.0313 (17)	0.0106 (15)	−0.0146 (13)
C53	0.095 (3)	0.0429 (19)	0.0324 (16)	−0.037 (2)	0.0151 (18)	−0.0207 (14)
C54	0.064 (2)	0.0371 (16)	0.0251 (14)	−0.0179 (16)	0.0092 (14)	−0.0132 (12)
C55	0.0400 (16)	0.0285 (13)	0.0211 (12)	−0.0098 (12)	0.0032 (11)	−0.0063 (10)
C56	0.0242 (12)	0.0233 (11)	0.0209 (11)	−0.0050 (9)	−0.0012 (9)	−0.0061 (9)
N10	0.0519 (17)	0.0280 (12)	0.0265 (12)	0.0030 (11)	−0.0066 (11)	−0.0107 (10)
C64	0.070 (3)	0.051 (2)	0.0398 (19)	−0.0020 (19)	−0.0182 (18)	−0.0235 (16)
C65	0.134 (5)	0.072 (3)	0.039 (2)	−0.014 (3)	−0.021 (3)	−0.027 (2)
C66	0.226 (8)	0.058 (3)	0.0236 (18)	−0.029 (4)	0.006 (3)	−0.0124 (19)
C67	0.192 (7)	0.043 (2)	0.0316 (19)	−0.033 (3)	0.030 (3)	−0.0144 (17)
C68	0.103 (3)	0.0325 (16)	0.0265 (15)	−0.0188 (19)	0.0126 (18)	−0.0111 (13)
N12	0.0401 (16)	0.0375 (15)	0.0474 (17)	−0.0042 (12)	0.0003 (13)	0.0078 (12)
C74	0.046 (2)	0.082 (3)	0.0317 (17)	−0.005 (2)	−0.0031 (15)	−0.0073 (18)
C75	0.053 (2)	0.060 (3)	0.060 (3)	−0.006 (2)	0.010 (2)	−0.024 (2)
C76	0.048 (2)	0.046 (2)	0.068 (3)	0.0076 (17)	−0.001 (2)	0.0104 (19)
C77	0.054 (2)	0.083 (3)	0.042 (2)	−0.012 (2)	−0.0176 (18)	0.002 (2)
C78	0.051 (2)	0.049 (2)	0.059 (2)	−0.0147 (17)	−0.0024 (18)	−0.0176 (18)
N13	0.0473 (16)	0.0328 (13)	0.0350 (13)	−0.0145 (12)	0.0043 (12)	−0.0073 (11)
C79	0.056 (2)	0.0347 (16)	0.0404 (17)	−0.0146 (15)	0.0060 (15)	−0.0110 (13)
C80	0.052 (2)	0.0310 (16)	0.067 (3)	−0.0100 (15)	−0.0046 (19)	−0.0126 (16)
C81	0.066 (3)	0.043 (2)	0.085 (3)	−0.028 (2)	0.000 (2)	0.002 (2)
C82	0.077 (3)	0.074 (3)	0.056 (3)	−0.038 (3)	0.018 (2)	−0.004 (2)
C83	0.059 (2)	0.053 (2)	0.0361 (17)	−0.0248 (18)	0.0057 (16)	−0.0119 (15)
N14	0.110 (5)	0.093 (5)	0.062 (4)	−0.056 (4)	−0.020 (4)	−0.012 (4)
C84	0.108 (5)	0.097 (5)	0.066 (5)	−0.052 (4)	−0.014 (4)	−0.015 (4)
C85	0.126 (6)	0.101 (5)	0.069 (5)	−0.059 (5)	0.000 (4)	−0.015 (4)
C86	0.141 (6)	0.105 (5)	0.083 (5)	−0.059 (5)	0.001 (5)	−0.012 (4)
C87	0.141 (6)	0.106 (5)	0.080 (5)	−0.066 (5)	−0.009 (5)	−0.012 (4)
C88	0.127 (6)	0.100 (5)	0.069 (4)	−0.057 (4)	−0.013 (4)	−0.014 (4)
N22	0.113 (5)	0.100 (5)	0.066 (4)	−0.049 (4)	−0.030 (4)	−0.016 (4)
C124	0.109 (5)	0.098 (5)	0.069 (5)	−0.049 (5)	−0.017 (4)	−0.018 (4)
C125	0.119 (5)	0.100 (6)	0.072 (5)	−0.057 (4)	−0.007 (4)	−0.028 (4)
C126	0.140 (6)	0.106 (5)	0.079 (5)	−0.056 (5)	0.001 (5)	−0.019 (4)
C127	0.139 (6)	0.101 (5)	0.076 (5)	−0.054 (5)	−0.003 (5)	−0.015 (4)
C128	0.131 (6)	0.102 (6)	0.071 (5)	−0.047 (5)	−0.016 (5)	−0.013 (5)
N15	0.079 (2)	0.0519 (19)	0.0503 (19)	−0.0192 (18)	−0.0045 (18)	0.0178 (16)
C89	0.078 (4)	0.055 (4)	0.053 (3)	−0.017 (3)	−0.006 (3)	0.016 (3)
C90	0.095 (4)	0.055 (4)	0.055 (3)	−0.016 (4)	0.004 (3)	0.018 (3)
C91	0.093 (4)	0.068 (4)	0.070 (4)	−0.019 (4)	0.008 (4)	0.022 (3)
C92	0.089 (4)	0.078 (4)	0.082 (4)	−0.019 (4)	−0.003 (3)	0.017 (4)
C93	0.087 (4)	0.073 (4)	0.065 (3)	−0.009 (3)	−0.007 (3)	0.018 (3)
N23	0.079 (2)	0.0519 (19)	0.0503 (19)	−0.0192 (18)	−0.0045 (18)	0.0178 (16)
C129	0.078 (4)	0.053 (4)	0.053 (4)	−0.017 (4)	−0.006 (4)	0.015 (4)
C130	0.086 (4)	0.058 (4)	0.056 (4)	−0.019 (4)	−0.002 (4)	0.017 (4)
C131	0.092 (5)	0.073 (5)	0.072 (4)	−0.017 (4)	0.007 (4)	0.016 (4)
C132	0.087 (4)	0.075 (4)	0.075 (4)	−0.015 (4)	0.001 (4)	0.015 (4)
C133	0.083 (4)	0.064 (4)	0.062 (4)	−0.016 (4)	−0.007 (4)	0.013 (4)
N25	0.090 (7)	0.077 (7)	0.074 (7)	−0.012 (7)	−0.010 (7)	0.016 (7)
C139	0.086 (5)	0.066 (5)	0.063 (5)	−0.013 (5)	−0.006 (5)	0.015 (5)
C140	0.087 (3)	0.064 (3)	0.063 (3)	−0.016 (3)	−0.002 (3)	0.017 (3)
C141	0.089 (4)	0.072 (4)	0.071 (4)	−0.015 (4)	0.001 (4)	0.016 (4)
C142	0.090 (7)	0.080 (7)	0.080 (7)	−0.012 (7)	−0.001 (7)	0.014 (7)
C143	0.092 (9)	0.082 (9)	0.080 (9)	−0.008 (9)	−0.005 (9)	0.015 (9)
N16	0.0424 (15)	0.0387 (14)	0.0407 (15)	−0.0140 (12)	−0.0161 (12)	−0.0001 (12)
C94	0.0422 (18)	0.0482 (19)	0.0405 (17)	−0.0228 (15)	−0.0049 (14)	−0.0006 (14)
C95	0.0407 (19)	0.045 (2)	0.058 (2)	−0.0139 (16)	−0.0023 (16)	−0.0016 (17)
C96	0.050 (2)	0.068 (3)	0.062 (3)	0.005 (2)	−0.009 (2)	0.021 (2)
C97	0.061 (3)	0.084 (3)	0.051 (2)	0.008 (2)	0.002 (2)	0.025 (2)
C98	0.050 (2)	0.055 (2)	0.045 (2)	−0.0018 (18)	−0.0028 (17)	0.0047 (17)
N17	0.088 (5)	0.078 (4)	0.064 (4)	−0.012 (4)	0.007 (4)	−0.015 (4)
C99	0.079 (5)	0.067 (4)	0.062 (4)	−0.025 (4)	0.001 (4)	−0.001 (4)
C100	0.076 (4)	0.086 (5)	0.059 (5)	−0.024 (4)	0.006 (4)	0.000 (4)
C101	0.080 (4)	0.095 (6)	0.054 (4)	−0.024 (5)	0.009 (4)	−0.018 (4)
C102	0.082 (5)	0.099 (5)	0.059 (4)	−0.023 (4)	0.007 (4)	−0.010 (4)
C103	0.079 (5)	0.088 (5)	0.054 (4)	−0.015 (4)	0.013 (4)	−0.007 (4)
N24	0.090 (6)	0.085 (5)	0.059 (4)	−0.019 (4)	0.003 (4)	−0.015 (4)
C134	0.077 (5)	0.068 (4)	0.063 (4)	−0.028 (4)	0.001 (4)	−0.004 (4)
C135	0.077 (4)	0.084 (5)	0.055 (4)	−0.027 (4)	0.011 (4)	−0.002 (4)
C136	0.077 (4)	0.088 (5)	0.050 (4)	−0.029 (4)	0.010 (4)	−0.009 (4)
C137	0.088 (5)	0.095 (5)	0.061 (4)	−0.029 (5)	0.005 (4)	−0.010 (4)
C138	0.093 (6)	0.099 (5)	0.059 (4)	−0.031 (5)	0.012 (4)	−0.009 (4)
N18	0.0460 (18)	0.057 (2)	0.059 (2)	−0.0087 (15)	−0.0152 (15)	−0.0168 (16)
C104	0.072 (3)	0.052 (2)	0.066 (3)	−0.024 (2)	−0.020 (2)	−0.005 (2)
C105	0.074 (3)	0.056 (3)	0.066 (3)	−0.016 (2)	−0.017 (2)	−0.006 (2)
C106	0.062 (3)	0.074 (3)	0.065 (3)	−0.009 (2)	−0.013 (2)	−0.033 (2)
C107	0.130 (5)	0.072 (3)	0.053 (3)	−0.019 (3)	−0.034 (3)	−0.006 (2)
C108	0.108 (4)	0.051 (3)	0.077 (3)	0.002 (3)	−0.038 (3)	−0.014 (2)
N19	0.058 (2)	0.069 (2)	0.068 (2)	−0.0134 (19)	0.0030 (19)	−0.016 (2)
C109	0.039 (2)	0.075 (3)	0.068 (3)	0.003 (2)	0.0013 (19)	−0.023 (2)
C110	0.058 (3)	0.116 (5)	0.060 (3)	−0.043 (3)	−0.003 (2)	−0.015 (3)
C111	0.072 (3)	0.058 (2)	0.0367 (18)	−0.022 (2)	0.0018 (18)	−0.0075 (17)
C112	0.0391 (19)	0.045 (2)	0.056 (2)	−0.0035 (16)	0.0038 (16)	0.0015 (17)
C113	0.057 (3)	0.055 (2)	0.064 (3)	−0.014 (2)	−0.005 (2)	−0.001 (2)
N20	0.095 (4)	0.137 (6)	0.119 (5)	−0.054 (4)	−0.023 (4)	−0.038 (4)
C114	0.099 (5)	0.143 (7)	0.142 (7)	−0.070 (5)	0.031 (5)	−0.084 (6)
C115	0.092 (5)	0.110 (6)	0.180 (9)	−0.043 (4)	−0.020 (5)	−0.079 (6)
C116	0.091 (5)	0.117 (6)	0.102 (5)	−0.033 (4)	−0.048 (4)	−0.025 (4)
C117	0.098 (5)	0.147 (8)	0.093 (5)	−0.009 (5)	−0.019 (4)	−0.023 (5)
C118	0.088 (5)	0.120 (6)	0.106 (6)	−0.004 (4)	−0.034 (4)	−0.033 (5)
N21	0.091 (6)	0.077 (8)	0.054 (7)	−0.030 (6)	−0.016 (6)	0.006 (6)
C119	0.089 (6)	0.054 (7)	0.043 (5)	−0.011 (5)	−0.020 (5)	−0.019 (4)
C120	0.071 (6)	0.066 (7)	0.022 (4)	−0.001 (5)	0.000 (4)	−0.015 (4)
C121	0.092 (7)	0.071 (9)	0.054 (6)	−0.021 (7)	−0.017 (6)	0.001 (6)
C122	0.102 (7)	0.072 (9)	0.069 (8)	−0.013 (7)	−0.017 (7)	−0.019 (7)
C123	0.093 (7)	0.086 (10)	0.064 (9)	−0.014 (7)	−0.012 (8)	−0.013 (7)

### Bis(pyridinium) diaquadipyridinehexakis[µ3-salicylhydroximato(3-)]bis[µ2-salicylhydroximato(1-)]hexaaluminiumdysprosium–pyridine–water (1/7.429/1) (2) Geometric parameters (Å, º)

**Table:**

Al1—O24	1.8242 (19)	C20—H20	0.9500
Al1—O2	1.879 (2)	C29—C30	1.481 (5)
Al1—O9	1.9004 (19)	C30—C31	1.381 (6)
Al1—O1	1.9112 (19)	C30—C35	1.407 (6)
Al1—N3	1.976 (2)	C31—C32	1.379 (6)
Al1—N8	2.079 (2)	C31—H31	0.9500
Al2—O3	1.820 (2)	C32—C33	1.379 (8)
Al2—O17	1.876 (2)	C32—H32	0.9500
Al2—O5	1.894 (2)	C33—C34	1.362 (8)
Al2—O4	1.895 (2)	C33—H33	0.9500
Al2—O16	1.948 (2)	C34—C35	1.392 (7)
Al2—N1	1.998 (2)	C34—H34	0.9500
Al2—Dy1B	3.148 (3)	C36—C37	1.483 (4)
Al2—Dy1	3.4330 (9)	C37—C38	1.397 (5)
Al3—O6B	1.808 (2)	C37—C42	1.410 (5)
Al3—O6	1.808 (2)	C38—C39	1.388 (5)
Al3—O26B	1.84 (3)	C38—H38	0.9500
Al3—O26	1.855 (4)	C39—C40	1.377 (7)
Al3—O25	1.879 (3)	C39—H39	0.9500
Al3—N2	1.907 (2)	C40—C41	1.375 (7)
Al3—O28	2.000 (2)	C40—H40	0.9500
Al3—O25B	2.05 (2)	C41—C42	1.415 (4)
Al3—N10	2.119 (3)	C41—H41	0.9500
Al5—O18	1.804 (2)	C43—C44	1.475 (4)
Al5—O20	1.862 (2)	C44—C45	1.408 (4)
Al5—O19	1.906 (2)	C44—C49	1.421 (4)
Al5—O13	1.918 (2)	C45—C46	1.383 (5)
Al5—O14	1.943 (3)	C45—H45	0.9500
Al5—N6	2.028 (3)	C46—C47	1.389 (5)
Al5—Dy1B	3.182 (2)	C46—H46	0.9500
Al5—Dy1	3.4016 (9)	C47—C48	1.378 (4)
Al6—O21	1.811 (2)	C47—H47	0.9500
Al6—O23	1.839 (2)	C48—C49	1.412 (4)
Al6—O22	1.886 (2)	C48—H48	0.9500
Al6—N7	1.936 (2)	C50—C51	1.471 (3)
Al6—O29	1.981 (2)	C51—C52	1.398 (4)
Al6—O12B	2.03 (2)	C51—C56	1.408 (4)
Al6—N11	2.119 (3)	C52—C53	1.385 (4)
O1—N1	1.422 (3)	C52—H52	0.9500
O1—Dy1	2.3565 (17)	C53—C54	1.386 (5)
O1—Dy1B	2.390 (3)	C53—H53	0.9500
O2—C1	1.294 (3)	C54—C55	1.378 (4)
O3—C7	1.324 (3)	C54—H54	0.9500
O4—N2	1.398 (3)	C55—C56	1.408 (4)
O4—Dy1B	2.226 (3)	C55—H55	0.9500
O4—Dy1	2.4140 (18)	N10—C64	1.340 (5)
O5—C8B	1.294 (3)	N10—C68	1.342 (5)
O5—C8	1.294 (3)	C64—C65	1.395 (6)
O7—N3	1.402 (3)	C64—H64	0.9500
O7—Al4B	1.676 (10)	C65—C66	1.368 (9)
O7—Al4	1.905 (2)	C65—H65	0.9500
O7—Dy1	2.3278 (19)	C66—C67	1.364 (9)
O7—Dy1B	2.670 (3)	C66—H66	0.9500
O8—C15	1.300 (3)	C67—C68	1.392 (5)
O8—Al4	1.851 (2)	C67—H67	0.9500
O8—Al4B	2.223 (10)	C68—H68	0.9500
O9—C21	1.341 (3)	N12—C74	1.321 (5)
Dy1—O22	2.3737 (18)	N12—C78	1.341 (5)
Dy1—O25	2.380 (3)	N12—H12A	0.8800
Dy1—O19	2.4000 (18)	C74—C75	1.359 (6)
Dy1—O16	2.499 (2)	C74—H74	0.9500
Dy1—O13	2.5157 (19)	C75—C76	1.376 (7)
Dy1—O10	2.596 (2)	C75—H75	0.9500
Dy1—Al4	3.4698 (12)	C76—C77	1.384 (7)
Al4—O27	1.859 (3)	C76—H76	0.9500
Al4—O11	1.898 (2)	C77—C78	1.365 (6)
Al4—O10	1.945 (3)	C77—H77	0.9500
Al4—N9	1.976 (3)	C78—H78	0.9500
O10—N4	1.374 (3)	N13—C83	1.320 (4)
N4—C22	1.313 (4)	N13—C79	1.333 (4)
N4—H4N	0.8800	C79—C80	1.373 (5)
O11—C22	1.281 (4)	C79—H79	0.9500
C22—C23	1.471 (4)	C80—C81	1.360 (6)
C23—C24	1.397 (5)	C80—H80	0.9500
C23—C28	1.406 (5)	C81—C82	1.387 (6)
C24—C25	1.391 (5)	C81—H81	0.9500
C24—H24	0.9500	C82—C83	1.374 (5)
C25—C26	1.382 (7)	C82—H82	0.9500
C25—H25	0.9500	C83—H83	0.9500
C26—C27	1.369 (8)	N14—C88	1.325 (12)
C26—H26	0.9500	N14—C84	1.346 (11)
C27—C28	1.403 (5)	C84—C85	1.361 (12)
C27—H27	0.9500	C84—H84	0.9500
C28—O12	1.354 (5)	C85—C86	1.351 (12)
O12—H12O	0.8400	C85—H85	0.9500
O25—N9	1.416 (4)	C86—C87	1.379 (12)
N9—C57	1.307 (4)	C86—H86	0.9500
O26—C57	1.301 (4)	C87—C88	1.376 (12)
C57—C58	1.471 (4)	C87—H87	0.9500
C58—C59	1.407 (5)	C88—H88	0.9500
C58—C63	1.409 (5)	N22—C128	1.335 (13)
C59—C60	1.378 (5)	N22—C124	1.339 (11)
C59—H59	0.9500	C124—C125	1.359 (13)
C60—C61	1.390 (6)	C124—H124	0.9500
C60—H60	0.9500	C125—C126	1.372 (12)
C61—C62	1.388 (5)	C125—H125	0.9500
C61—H61	0.9500	C126—C127	1.397 (12)
C62—C63	1.397 (5)	C126—H126	0.9500
C62—H62	0.9500	C127—C128	1.358 (12)
C63—O27	1.344 (4)	C127—H127	0.9500
N11—C73	1.335 (4)	C128—H128	0.9500
N11—C69	1.343 (4)	N15—C89	1.314 (9)
C69—C70	1.378 (5)	N15—C129	1.326 (12)
C69—H69	0.9500	N15—C133	1.330 (12)
C70—C71	1.373 (6)	N15—C93	1.346 (10)
C70—H70	0.9500	C89—N23	1.314 (9)
C71—C72	1.371 (6)	C89—C90	1.353 (10)
C71—H71	0.9500	C89—C130	1.44 (3)
C72—C73	1.385 (5)	C89—H89	0.9500
C72—H72	0.9500	C90—C129	1.26 (2)
C73—H73	0.9500	C90—C91	1.402 (13)
Dy1B—O16	2.142 (3)	C90—C131	1.60 (2)
Dy1B—O19	2.277 (3)	C90—H90	0.9500
Dy1B—O25B	2.353 (15)	C91—C131	0.754 (19)
Dy1B—O22	2.383 (3)	C91—C130	1.21 (3)
Dy1B—O13	2.410 (3)	C91—C92	1.410 (11)
Dy1B—O10B	2.611 (14)	C91—H91	0.9500
Dy1B—N6	2.815 (3)	C92—C132	0.927 (19)
Al4B—O27B	1.848 (14)	C92—C93	1.355 (11)
Al4B—O11B	1.906 (13)	C92—C131	1.50 (2)
Al4B—O10B	1.934 (15)	C92—C133	1.612 (18)
Al4B—N9B	1.970 (14)	C92—H92	0.9500
O10B—N4B	1.392 (16)	C93—C133	0.52 (2)
N4B—C22B	1.325 (16)	C93—N23	1.346 (10)
N4B—H4B	0.88 (2)	C93—C132	1.43 (2)
O11B—C22B	1.289 (16)	C93—H93	0.9500
C22B—C23B	1.470 (15)	N23—C129	1.326 (12)
C23B—C24B	1.403 (17)	N23—C133	1.330 (12)
C23B—C28B	1.407 (17)	C129—C130	1.359 (13)
C24B—C25B	1.393 (18)	C129—H129	0.9500
C24B—H24B	0.9500	C130—C131	1.372 (13)
C25B—C26B	1.381 (19)	C130—H130	0.9500
C25B—H25B	0.9500	C131—C132	1.398 (13)
C26B—C27B	1.349 (18)	C131—H131	0.9500
C26B—H26B	0.9500	C132—C133	1.368 (13)
C27B—C28B	1.434 (17)	C132—H132	0.9500
C27B—H27B	0.9500	C133—H133	0.9500
C28B—O12B	1.339 (17)	N25—C139	1.3900
O12B—H12P	0.8400	N25—C143	1.3900
O25B—N9B	1.412 (16)	C139—C140	1.3900
N9B—C57B	1.295 (16)	C139—H139	0.9500
O26B—C57B	1.305 (17)	C140—C141	1.3900
C57B—C58B	1.486 (16)	C140—H140	0.9500
C58B—C59B	1.385 (17)	C141—C142	1.3900
C58B—C63B	1.404 (17)	C141—H141	0.9500
C59B—C60B	1.398 (18)	C142—C143	1.3900
C59B—H59B	0.9500	C142—H142	0.9500
C60B—C61B	1.381 (18)	C143—H143	0.9500
C60B—H60B	0.9500	N16—C98	1.329 (5)
C61B—C62B	1.371 (18)	N16—C94	1.352 (5)
C61B—H61B	0.9500	N16—H16	0.8800
C62B—C63B	1.396 (17)	C94—C95	1.356 (5)
C62B—H62B	0.9500	C94—H94	0.9500
C63B—O27B	1.338 (16)	C95—C96	1.387 (6)
O13—N5	1.367 (3)	C95—H95	0.9500
O14—C29	1.276 (4)	C96—C97	1.378 (6)
O15—C35	1.352 (6)	C96—H96	0.9500
O15—H15	0.8400	C97—C98	1.364 (6)
O16—N6	1.438 (3)	C97—H97	0.9500
O17—C36	1.291 (4)	C98—H98	0.9500
O18—C42	1.324 (4)	N17—C99	1.310 (11)
O19—N7	1.414 (3)	N17—C103	1.330 (11)
O20—C43	1.300 (3)	C99—C100	1.376 (12)
O21—C49	1.317 (3)	C99—H99	0.9500
O22—N8	1.425 (3)	C100—C101	1.345 (12)
O23—C50	1.302 (3)	C100—H100	0.9500
O24—C56	1.321 (3)	C101—C102	1.413 (12)
O28—H28A	0.837 (18)	C101—H101	0.9500
O28—H28B	0.822 (18)	C102—C103	1.366 (11)
O29—H29A	0.886 (19)	C102—H102	0.9500
O29—H29B	0.913 (19)	C103—H103	0.9500
O30—H30A	0.86 (2)	N24—C134	1.312 (12)
O30—H30B	0.872 (18)	N24—C138	1.356 (12)
N1—C1	1.314 (3)	C134—C135	1.349 (11)
N2—C8B	1.316 (3)	C134—H134	0.9500
N2—C8	1.316 (3)	C135—C136	1.342 (12)
N3—C15	1.308 (3)	C135—H135	0.9500
N5—C29	1.312 (4)	C136—C137	1.415 (12)
N5—H5N	0.8800	C136—H136	0.9500
N6—C36	1.319 (4)	C137—C138	1.373 (11)
N7—C43	1.306 (4)	C137—H137	0.9500
N8—C50	1.312 (3)	C138—H138	0.9500
C1—C2	1.468 (4)	N18—C108	1.310 (6)
C2—C3	1.406 (4)	N18—C104	1.341 (5)
C2—C7	1.413 (4)	C104—C105	1.362 (6)
C3—C4	1.382 (4)	C104—H104	0.9500
C3—H3	0.9500	C105—C106	1.358 (7)
C4—C5	1.386 (5)	C105—H105	0.9500
C4—H4	0.9500	C106—C107	1.413 (7)
C5—C6	1.387 (5)	C106—H106	0.9500
C5—H5	0.9500	C107—C108	1.359 (7)
C6—C7	1.405 (4)	C107—H107	0.9500
C6—H6	0.9500	C108—H108	0.9500
C8—C9	1.476 (4)	N19—C109	1.303 (6)
C9—C10	1.404 (5)	N19—C113	1.303 (6)
C9—C14	1.416 (5)	C109—C110	1.374 (7)
C10—C11	1.393 (5)	C109—H109	0.9500
C10—H10	0.9500	C110—C111	1.448 (7)
C11—C12	1.389 (6)	C110—H110	0.9500
C11—H11	0.9500	C111—C112	1.360 (6)
C12—C13	1.382 (5)	C111—H111	0.9500
C12—H12	0.9500	C112—C113	1.366 (6)
C13—C14	1.411 (4)	C112—H112	0.9500
C13—H13	0.9500	C113—H113	0.9500
C14—O6	1.336 (4)	N20—C118	1.294 (9)
C8B—C9B	1.485 (17)	N20—C114	1.343 (10)
C9B—C10B	1.406 (18)	C114—C115	1.396 (12)
C9B—C14B	1.421 (17)	C114—H114	0.9500
C10B—C11B	1.395 (17)	C115—C116	1.353 (10)
C10B—H10B	0.9500	C115—H115	0.9500
C11B—C12B	1.385 (18)	C116—C117	1.359 (10)
C11B—H11B	0.9500	C116—H116	0.9500
C12B—C13B	1.386 (17)	C117—C118	1.357 (11)
C12B—H12B	0.9500	C117—H117	0.9500
C13B—C14B	1.412 (17)	C118—H118	0.9500
C13B—H13B	0.9500	N21—C119	1.3900
C14B—O6B	1.330 (16)	N21—C123	1.3900
C15—C16	1.470 (4)	C119—C120	1.3900
C16—C17	1.395 (4)	C119—H119	0.9500
C16—C21	1.420 (3)	C120—C121	1.3900
C17—C18	1.385 (4)	C120—H120	0.9500
C17—H17	0.9500	C121—C122	1.3900
C18—C19	1.388 (4)	C121—H121	0.9500
C18—H18	0.9500	C122—C123	1.3900
C19—C20	1.390 (4)	C122—H122	0.9500
C19—H19	0.9500	C123—H123	0.9500
C20—C21	1.404 (3)		
			
O24—Al1—O2	96.27 (9)	O19—N7—Al6	119.80 (16)
O24—Al1—O9	89.38 (8)	C50—N8—O22	110.0 (2)
O2—Al1—O9	88.84 (9)	C50—N8—Al1	125.73 (17)
O24—Al1—O1	170.39 (9)	O22—N8—Al1	123.18 (15)
O2—Al1—O1	81.87 (8)	O2—C1—N1	120.8 (2)
O9—Al1—O1	99.98 (8)	O2—C1—C2	119.5 (2)
O24—Al1—N3	100.49 (9)	N1—C1—C2	119.7 (2)
O2—Al1—N3	161.85 (9)	C3—C2—C7	119.8 (3)
O9—Al1—N3	84.48 (9)	C3—C2—C1	118.1 (2)
O1—Al1—N3	82.70 (9)	C7—C2—C1	122.1 (2)
O24—Al1—N8	85.96 (9)	C4—C3—C2	121.3 (3)
O2—Al1—N8	95.44 (9)	C4—C3—H3	119.4
O9—Al1—N8	173.99 (9)	C2—C3—H3	119.4
O1—Al1—N8	84.84 (8)	C3—C4—C5	118.7 (3)
N3—Al1—N8	92.62 (9)	C3—C4—H4	120.7
O3—Al2—O17	91.28 (10)	C5—C4—H4	120.7
O3—Al2—O5	97.53 (10)	C4—C5—C6	121.4 (3)
O17—Al2—O5	94.68 (9)	C4—C5—H5	119.3
O3—Al2—O4	175.07 (10)	C6—C5—H5	119.3
O17—Al2—O4	93.63 (9)	C5—C6—C7	120.7 (3)
O5—Al2—O4	82.50 (9)	C5—C6—H6	119.7
O3—Al2—O16	102.41 (10)	C7—C6—H6	119.7
O17—Al2—O16	81.82 (9)	O3—C7—C6	119.1 (3)
O5—Al2—O16	159.81 (9)	O3—C7—C2	122.8 (3)
O4—Al2—O16	77.91 (8)	C6—C7—C2	118.0 (3)
O3—Al2—N1	87.98 (9)	O5—C8—N2	120.5 (2)
O17—Al2—N1	169.86 (10)	O5—C8—C9	120.5 (3)
O5—Al2—N1	95.44 (9)	N2—C8—C9	119.0 (3)
O4—Al2—N1	87.11 (9)	C10—C9—C14	119.8 (4)
O16—Al2—N1	88.46 (9)	C10—C9—C8	117.7 (3)
O3—Al2—Dy1B	133.49 (9)	C14—C9—C8	122.4 (3)
O17—Al2—Dy1B	106.73 (8)	C11—C10—C9	121.5 (4)
O5—Al2—Dy1B	122.36 (8)	C11—C10—H10	119.3
O4—Al2—Dy1B	44.24 (7)	C9—C10—H10	119.3
O16—Al2—Dy1B	41.98 (7)	C12—C11—C10	118.7 (3)
N1—Al2—Dy1B	67.05 (8)	C12—C11—H11	120.7
O3—Al2—Dy1	134.54 (8)	C10—C11—H11	120.7
O17—Al2—Dy1	109.95 (7)	C13—C12—C11	120.9 (3)
O5—Al2—Dy1	119.16 (7)	C13—C12—H12	119.5
O4—Al2—Dy1	42.78 (6)	C11—C12—H12	119.5
O16—Al2—Dy1	45.72 (6)	C12—C13—C14	121.6 (3)
N1—Al2—Dy1	64.23 (7)	C12—C13—H13	119.2
O6B—Al3—O26B	103.6 (6)	C14—C13—H13	119.2
O6—Al3—O26	93.61 (11)	O6—C14—C13	118.2 (3)
O6—Al3—O25	176.03 (13)	O6—C14—C9	124.2 (3)
O26—Al3—O25	84.59 (12)	C13—C14—C9	117.6 (3)
O6B—Al3—N2	91.77 (10)	C14—O6—Al3	127.6 (2)
O6—Al3—N2	91.77 (10)	O5—C8B—N2	120.5 (2)
O26B—Al3—N2	164.2 (6)	O5—C8B—C9B	121.2 (10)
O26—Al3—N2	174.50 (12)	N2—C8B—C9B	118.3 (11)
O25—Al3—N2	89.97 (11)	C10B—C9B—C14B	119 (2)
O6B—Al3—O28	90.13 (9)	C10B—C9B—C8B	118 (2)
O6—Al3—O28	90.13 (9)	C14B—C9B—C8B	122 (2)
O26B—Al3—O28	95.5 (11)	C11B—C10B—C9B	121 (2)
O26—Al3—O28	90.78 (14)	C11B—C10B—H10B	119.3
O25—Al3—O28	86.36 (12)	C9B—C10B—H10B	119.3
N2—Al3—O28	88.10 (9)	C12B—C11B—C10B	118.9 (19)
O6B—Al3—O25B	175.4 (7)	C12B—C11B—H11B	120.6
O26B—Al3—O25B	79.5 (7)	C10B—C11B—H11B	120.6
N2—Al3—O25B	84.9 (5)	C11B—C12B—C13B	121.5 (19)
O28—Al3—O25B	93.0 (10)	C11B—C12B—H12B	119.3
O6B—Al3—N10	91.81 (10)	C13B—C12B—H12B	119.3
O6—Al3—N10	91.81 (10)	C12B—C13B—C14B	120.1 (18)
O26B—Al3—N10	85.3 (11)	C12B—C13B—H13B	120.0
O26—Al3—N10	90.35 (15)	C14B—C13B—H13B	120.0
O25—Al3—N10	91.74 (12)	O6B—C14B—C13B	117.0 (17)
N2—Al3—N10	90.58 (11)	O6B—C14B—C9B	123.1 (19)
O28—Al3—N10	177.69 (10)	C13B—C14B—C9B	119.1 (15)
O25B—Al3—N10	85.0 (9)	C14B—O6B—Al3	123.3 (15)
O18—Al5—O20	93.65 (11)	O8—C15—N3	120.7 (2)
O18—Al5—O19	175.23 (12)	O8—C15—C16	120.4 (2)
O20—Al5—O19	82.41 (9)	N3—C15—C16	118.9 (2)
O18—Al5—O13	101.08 (11)	C17—C16—C21	120.2 (2)
O20—Al5—O13	164.73 (11)	C17—C16—C15	118.7 (2)
O19—Al5—O13	83.05 (9)	C21—C16—C15	121.0 (2)
O18—Al5—O14	90.43 (11)	C18—C17—C16	121.2 (3)
O20—Al5—O14	94.16 (11)	C18—C17—H17	119.4
O19—Al5—O14	92.54 (10)	C16—C17—H17	119.4
O13—Al5—O14	81.83 (10)	C17—C18—C19	119.0 (3)
O18—Al5—N6	89.91 (11)	C17—C18—H18	120.5
O20—Al5—N6	99.84 (11)	C19—C18—H18	120.5
O19—Al5—N6	88.12 (10)	C18—C19—C20	120.6 (3)
O13—Al5—N6	84.31 (10)	C18—C19—H19	119.7
O14—Al5—N6	165.95 (11)	C20—C19—H19	119.7
O18—Al5—Dy1B	136.79 (9)	C19—C20—C21	121.4 (2)
O20—Al5—Dy1B	120.47 (8)	C19—C20—H20	119.3
O19—Al5—Dy1B	45.01 (7)	C21—C20—H20	119.3
O13—Al5—Dy1B	49.06 (7)	O9—C21—C20	120.0 (2)
O14—Al5—Dy1B	110.75 (9)	O9—C21—C16	122.6 (2)
N6—Al5—Dy1B	60.71 (8)	C20—C21—C16	117.4 (2)
O18—Al5—Dy1	139.05 (8)	O14—C29—N5	117.6 (3)
O20—Al5—Dy1	121.56 (7)	O14—C29—C30	121.2 (3)
O19—Al5—Dy1	43.26 (6)	N5—C29—C30	121.2 (3)
O13—Al5—Dy1	46.87 (6)	C31—C30—C35	118.8 (4)
O14—Al5—Dy1	105.68 (8)	C31—C30—C29	118.0 (4)
N6—Al5—Dy1	65.74 (7)	C35—C30—C29	123.2 (4)
O21—Al6—O23	93.82 (9)	C32—C31—C30	121.2 (5)
O21—Al6—O22	175.02 (11)	C32—C31—H31	119.4
O23—Al6—O22	83.96 (8)	C30—C31—H31	119.4
O21—Al6—N7	92.25 (10)	C31—C32—C33	119.4 (5)
O23—Al6—N7	173.76 (10)	C31—C32—H32	120.3
O22—Al6—N7	90.10 (9)	C33—C32—H32	120.3
O21—Al6—O29	87.56 (10)	C34—C33—C32	120.8 (5)
O23—Al6—O29	93.48 (10)	C34—C33—H33	119.6
O22—Al6—O29	88.13 (10)	C32—C33—H33	119.6
N7—Al6—O29	88.21 (10)	C33—C34—C35	120.5 (5)
O21—Al6—O12B	90.7 (6)	C33—C34—H34	119.8
O23—Al6—O12B	104.8 (6)	C35—C34—H34	119.8
O22—Al6—O12B	94.2 (6)	O15—C35—C34	121.9 (5)
N7—Al6—O12B	73.7 (6)	O15—C35—C30	118.8 (4)
O29—Al6—O12B	161.7 (6)	C34—C35—C30	119.3 (5)
O21—Al6—N11	88.52 (11)	O17—C36—N6	121.8 (2)
O23—Al6—N11	87.84 (11)	O17—C36—C37	118.0 (3)
O22—Al6—N11	95.84 (11)	N6—C36—C37	120.3 (3)
N7—Al6—N11	90.89 (12)	C38—C37—C42	120.3 (3)
O29—Al6—N11	175.93 (11)	C38—C37—C36	117.3 (3)
N1—O1—Al1	111.91 (14)	C42—C37—C36	122.4 (3)
N1—O1—Dy1	109.53 (13)	C39—C38—C37	121.1 (4)
Al1—O1—Dy1	119.17 (9)	C39—C38—H38	119.4
N1—O1—Dy1B	100.75 (14)	C37—C38—H38	119.4
Al1—O1—Dy1B	124.87 (10)	C40—C39—C38	119.0 (4)
C1—O2—Al1	113.11 (16)	C40—C39—H39	120.5
C7—O3—Al2	131.46 (18)	C38—C39—H39	120.5
N2—O4—Al2	112.22 (15)	C41—C40—C39	120.8 (4)
N2—O4—Dy1B	134.31 (16)	C41—C40—H40	119.6
Al2—O4—Dy1B	99.34 (10)	C39—C40—H40	119.6
N2—O4—Dy1	126.60 (14)	C40—C41—C42	121.8 (4)
Al2—O4—Dy1	105.01 (8)	C40—C41—H41	119.1
C8B—O5—Al2	112.04 (17)	C42—C41—H41	119.1
C8—O5—Al2	112.04 (17)	O18—C42—C37	124.1 (3)
N3—O7—Al4B	126.1 (4)	O18—C42—C41	119.0 (3)
N3—O7—Al4	111.96 (15)	C37—C42—C41	116.8 (4)
N3—O7—Dy1	118.98 (14)	O20—C43—N7	120.5 (3)
Al4—O7—Dy1	109.71 (9)	O20—C43—C44	118.9 (2)
N3—O7—Dy1B	117.18 (15)	N7—C43—C44	120.5 (2)
Al4B—O7—Dy1B	104.4 (4)	C45—C44—C49	119.1 (3)
C15—O8—Al4	112.32 (18)	C45—C44—C43	118.0 (3)
C15—O8—Al4B	104.9 (3)	C49—C44—C43	122.8 (2)
C21—O9—Al1	126.53 (16)	C46—C45—C44	121.5 (3)
O7—Dy1—O1	73.66 (6)	C46—C45—H45	119.2
O7—Dy1—O22	73.93 (7)	C44—C45—H45	119.2
O1—Dy1—O22	73.89 (6)	C45—C46—C47	119.2 (3)
O7—Dy1—O25	75.66 (8)	C45—C46—H46	120.4
O1—Dy1—O25	86.45 (9)	C47—C46—H46	120.4
O22—Dy1—O25	147.35 (9)	C48—C47—C46	120.7 (3)
O7—Dy1—O19	115.44 (7)	C48—C47—H47	119.7
O1—Dy1—O19	137.47 (6)	C46—C47—H47	119.7
O22—Dy1—O19	69.92 (6)	C47—C48—C49	121.5 (3)
O25—Dy1—O19	135.76 (9)	C47—C48—H48	119.3
O7—Dy1—O4	128.62 (6)	C49—C48—H48	119.3
O1—Dy1—O4	69.15 (6)	O21—C49—C48	118.6 (3)
O22—Dy1—O4	125.07 (7)	O21—C49—C44	123.5 (3)
O25—Dy1—O4	67.98 (8)	C48—C49—C44	117.9 (3)
O19—Dy1—O4	115.94 (7)	O23—C50—N8	121.5 (2)
O7—Dy1—O16	146.67 (6)	O23—C50—C51	118.2 (2)
O1—Dy1—O16	82.38 (6)	N8—C50—C51	120.2 (2)
O22—Dy1—O16	77.42 (7)	C52—C51—C56	119.8 (2)
O25—Dy1—O16	126.27 (8)	C52—C51—C50	117.6 (2)
O19—Dy1—O16	68.71 (7)	C56—C51—C50	122.3 (2)
O4—Dy1—O16	58.89 (6)	C53—C52—C51	121.5 (3)
O7—Dy1—O13	132.20 (7)	C53—C52—H52	119.2
O1—Dy1—O13	143.76 (7)	C51—C52—H52	119.2
O22—Dy1—O13	131.61 (6)	C52—C53—C54	118.8 (3)
O25—Dy1—O13	78.80 (9)	C52—C53—H53	120.6
O19—Dy1—O13	62.04 (6)	C54—C53—H53	120.6
O4—Dy1—O13	74.63 (7)	C55—C54—C53	120.5 (3)
O16—Dy1—O13	80.20 (7)	C55—C54—H54	119.8
O7—Dy1—O10	58.82 (7)	C53—C54—H54	119.8
O1—Dy1—O10	130.44 (7)	C54—C55—C56	121.9 (3)
O22—Dy1—O10	103.39 (7)	C54—C55—H55	119.1
O25—Dy1—O10	69.86 (9)	C56—C55—H55	119.1
O19—Dy1—O10	80.10 (7)	O24—C56—C51	123.4 (2)
O4—Dy1—O10	131.49 (7)	O24—C56—C55	119.2 (2)
O16—Dy1—O10	146.60 (7)	C51—C56—C55	117.3 (2)
O13—Dy1—O10	74.68 (7)	C64—N10—C68	117.5 (3)
O7—Dy1—Al5	142.05 (5)	C64—N10—Al3	120.3 (3)
O1—Dy1—Al5	141.27 (5)	C68—N10—Al3	122.1 (2)
O22—Dy1—Al5	98.75 (5)	N10—C64—C65	122.1 (5)
O25—Dy1—Al5	112.57 (8)	N10—C64—H64	119.0
O19—Dy1—Al5	32.98 (5)	C65—C64—H64	119.0
O4—Dy1—Al5	86.56 (5)	C66—C65—C64	119.1 (5)
O16—Dy1—Al5	59.03 (5)	C66—C65—H65	120.4
O13—Dy1—Al5	33.81 (5)	C64—C65—H65	120.4
O10—Dy1—Al5	88.27 (5)	C67—C66—C65	119.8 (4)
O7—Dy1—Al2	131.80 (5)	C67—C66—H66	120.1
O1—Dy1—Al2	58.19 (5)	C65—C66—H66	120.1
O22—Dy1—Al2	93.42 (5)	C66—C67—C68	118.1 (5)
O25—Dy1—Al2	98.06 (7)	C66—C67—H67	120.9
O19—Dy1—Al2	102.24 (5)	C68—C67—H67	120.9
O4—Dy1—Al2	32.21 (5)	N10—C68—C67	123.3 (4)
O16—Dy1—Al2	33.93 (5)	N10—C68—H68	118.3
O13—Dy1—Al2	91.14 (6)	C67—C68—H68	118.3
O10—Dy1—Al2	162.70 (5)	C74—N12—C78	121.9 (3)
Al5—Dy1—Al2	85.06 (2)	C74—N12—H12A	119.1
O7—Dy1—Al4	31.12 (5)	C78—N12—H12A	119.1
O1—Dy1—Al4	97.01 (5)	N12—C74—C75	120.8 (4)
O22—Dy1—Al4	99.91 (6)	N12—C74—H74	119.6
O25—Dy1—Al4	56.20 (7)	C75—C74—H74	119.6
O19—Dy1—Al4	110.40 (6)	C74—C75—C76	119.2 (4)
O4—Dy1—Al4	123.34 (5)	C74—C75—H75	120.4
O16—Dy1—Al4	177.33 (5)	C76—C75—H75	120.4
O13—Dy1—Al4	101.68 (6)	C75—C76—C77	119.1 (4)
O10—Dy1—Al4	33.65 (5)	C75—C76—H76	120.4
Al5—Dy1—Al4	121.69 (3)	C77—C76—H76	120.4
Al2—Dy1—Al4	147.26 (2)	C78—C77—C76	119.4 (4)
O8—Al4—O27	91.87 (10)	C78—C77—H77	120.3
O8—Al4—O11	99.87 (10)	C76—C77—H77	120.3
O27—Al4—O11	86.33 (11)	N12—C78—C77	119.6 (4)
O8—Al4—O7	82.26 (9)	N12—C78—H78	120.2
O27—Al4—O7	173.88 (11)	C77—C78—H78	120.2
O11—Al4—O7	96.32 (10)	C83—N13—C79	118.3 (3)
O8—Al4—O10	160.49 (11)	N13—C79—C80	123.6 (3)
O27—Al4—O10	107.64 (11)	N13—C79—H79	118.2
O11—Al4—O10	82.41 (10)	C80—C79—H79	118.2
O7—Al4—O10	78.23 (10)	C81—C80—C79	117.5 (4)
O8—Al4—N9	97.24 (11)	C81—C80—H80	121.2
O27—Al4—N9	86.40 (12)	C79—C80—H80	121.2
O11—Al4—N9	161.60 (12)	C80—C81—C82	119.9 (4)
O7—Al4—N9	92.62 (11)	C80—C81—H81	120.1
O10—Al4—N9	83.73 (11)	C82—C81—H81	120.1
O8—Al4—Dy1	114.62 (8)	C83—C82—C81	118.4 (4)
O27—Al4—Dy1	144.51 (9)	C83—C82—H82	120.8
O11—Al4—Dy1	110.21 (8)	C81—C82—H82	120.8
O7—Al4—Dy1	39.17 (6)	N13—C83—C82	122.3 (4)
O10—Al4—Dy1	47.70 (7)	N13—C83—H83	118.9
N9—Al4—Dy1	67.98 (9)	C82—C83—H83	118.9
N4—O10—Al4	108.88 (18)	C88—N14—C84	116.7 (13)
N4—O10—Dy1	118.66 (18)	N14—C84—C85	120.9 (12)
Al4—O10—Dy1	98.65 (9)	N14—C84—H84	119.5
C22—N4—O10	117.1 (2)	C85—C84—H84	119.5
C22—N4—H4N	121.5	C86—C85—C84	120.6 (12)
O10—N4—H4N	121.5	C86—C85—H85	119.7
C22—O11—Al4	114.1 (2)	C84—C85—H85	119.7
O11—C22—N4	117.4 (3)	C85—C86—C87	120.6 (11)
O11—C22—C23	120.7 (3)	C85—C86—H86	119.7
N4—C22—C23	121.7 (3)	C87—C86—H86	119.7
C24—C23—C28	119.1 (3)	C88—C87—C86	114.5 (11)
C24—C23—C22	117.7 (3)	C88—C87—H87	122.8
C28—C23—C22	123.1 (3)	C86—C87—H87	122.8
C25—C24—C23	121.1 (4)	N14—C88—C87	126.3 (12)
C25—C24—H24	119.4	N14—C88—H88	116.9
C23—C24—H24	119.4	C87—C88—H88	116.9
C26—C25—C24	118.8 (5)	C128—N22—C124	117.4 (13)
C26—C25—H25	120.6	N22—C124—C125	123.9 (14)
C24—C25—H25	120.6	N22—C124—H124	118.1
C27—C26—C25	121.4 (4)	C125—C124—H124	118.1
C27—C26—H26	119.3	C124—C125—C126	117.2 (11)
C25—C26—H26	119.3	C124—C125—H125	121.4
C26—C27—C28	120.4 (4)	C126—C125—H125	121.4
C26—C27—H27	119.8	C125—C126—C127	120.0 (11)
C28—C27—H27	119.8	C125—C126—H126	120.0
O12—C28—C27	123.1 (4)	C127—C126—H126	120.0
O12—C28—C23	117.8 (3)	C128—C127—C126	117.0 (12)
C27—C28—C23	119.1 (4)	C128—C127—H127	121.5
C28—O12—H12O	109.5	C126—C127—H127	121.5
N9—O25—Al3	109.59 (18)	N22—C128—C127	122.6 (13)
N9—O25—Dy1	117.74 (18)	N22—C128—H128	118.7
Al3—O25—Dy1	130.83 (15)	C127—C128—H128	118.7
C57—N9—O25	111.9 (2)	C89—N15—C133	119.3 (10)
C57—N9—Al4	129.6 (2)	C129—N15—C133	117.3 (10)
O25—N9—Al4	117.7 (2)	C89—N15—C93	117.2 (8)
C57—O26—Al3	110.6 (2)	C129—N15—C93	110.6 (10)
O26—C57—N9	120.9 (3)	N23—C89—C90	126.9 (11)
O26—C57—C58	119.9 (3)	N15—C89—C90	126.9 (11)
N9—C57—C58	119.2 (3)	N23—C89—C130	119.5 (13)
C59—C58—C63	119.3 (3)	N15—C89—C130	119.5 (13)
C59—C58—C57	118.3 (3)	N23—C89—H89	116.5
C63—C58—C57	122.4 (3)	N15—C89—H89	116.5
C60—C59—C58	121.3 (4)	C90—C89—H89	116.5
C60—C59—H59	119.4	C130—C89—H89	123.7
C58—C59—H59	119.4	C129—C90—C91	110.2 (13)
C59—C60—C61	119.0 (3)	C89—C90—C91	115.2 (10)
C59—C60—H60	120.5	C129—C90—C131	108.0 (11)
C61—C60—H60	120.5	C89—C90—C131	107.5 (11)
C62—C61—C60	121.0 (3)	C129—C90—H90	126.4
C62—C61—H61	119.5	C89—C90—H90	122.4
C60—C61—H61	119.5	C91—C90—H90	122.4
C61—C62—C63	120.4 (3)	C131—C90—H90	122.9
C61—C62—H62	119.8	C131—C91—C130	86 (2)
C63—C62—H62	119.8	C131—C91—C90	91 (2)
O27—C63—C62	118.8 (3)	C131—C91—C92	81.5 (16)
O27—C63—C58	122.3 (3)	C130—C91—C92	119.1 (12)
C62—C63—C58	118.9 (3)	C90—C91—C92	119.7 (9)
C63—O27—Al4	126.71 (19)	C131—C91—C132	50.0 (14)
C73—N11—C69	117.1 (3)	C130—C91—C132	106.2 (15)
C73—N11—Al6	122.3 (2)	C90—C91—C132	109.5 (11)
C69—N11—Al6	119.8 (2)	C131—C91—H91	97.7
N11—C69—C70	123.2 (4)	C130—C91—H91	120.4
N11—C69—H69	118.4	C90—C91—H91	120.1
C70—C69—H69	118.4	C92—C91—H91	120.1
C71—C70—C69	119.0 (4)	C132—C91—H91	121.0
C71—C70—H70	120.5	C132—C92—C93	75.3 (12)
C69—C70—H70	120.5	C132—C92—C91	95.2 (14)
C72—C71—C70	118.5 (4)	C93—C92—C91	118.2 (10)
C72—C71—H71	120.7	C132—C92—C131	65.6 (12)
C70—C71—H71	120.7	C93—C92—C131	111.0 (12)
C71—C72—C73	119.4 (4)	C132—C92—C133	57.9 (10)
C71—C72—H72	120.3	C91—C92—C133	114.6 (10)
C73—C72—H72	120.3	C131—C92—C133	99.4 (12)
N11—C73—C72	122.7 (4)	C132—C92—H92	99.1
N11—C73—H73	118.7	C93—C92—H92	120.9
C72—C73—H73	118.7	C91—C92—H92	120.9
O16—Dy1B—O4	67.14 (10)	C131—C92—H92	119.7
O16—Dy1B—O19	77.43 (11)	C133—C92—H92	121.6
O4—Dy1B—O19	130.04 (13)	C133—C93—N15	77.2 (19)
O16—Dy1B—O25B	132.7 (5)	C133—C93—N23	77.2 (19)
O4—Dy1B—O25B	67.0 (5)	C133—C93—C92	111 (3)
O19—Dy1B—O25B	126.8 (8)	N15—C93—C92	122.5 (9)
O16—Dy1B—O22	84.54 (11)	N23—C93—C92	122.5 (9)
O4—Dy1B—O22	134.36 (13)	C133—C93—C132	72 (2)
O19—Dy1B—O22	71.87 (9)	N15—C93—C132	116.7 (11)
O25B—Dy1B—O22	138.1 (7)	N23—C93—C132	116.7 (11)
O16—Dy1B—O1	89.65 (11)	C133—C93—H93	82.2
O4—Dy1B—O1	71.74 (9)	N15—C93—H93	118.7
O19—Dy1B—O1	143.60 (12)	N23—C93—H93	118.7
O25B—Dy1B—O1	86.7 (8)	C92—C93—H93	118.7
O22—Dy1B—O1	73.13 (9)	C132—C93—H93	110.2
O16—Dy1B—O13	90.21 (11)	C89—N23—C133	119.3 (10)
O4—Dy1B—O13	80.22 (10)	C129—N23—C133	117.3 (10)
O19—Dy1B—O13	65.44 (9)	C89—N23—C93	117.2 (8)
O25B—Dy1B—O13	71.3 (8)	C129—N23—C93	110.6 (10)
O22—Dy1B—O13	137.09 (12)	C90—C129—N23	134.4 (17)
O1—Dy1B—O13	149.57 (12)	C90—C129—N15	134.4 (17)
O16—Dy1B—O10B	143.0 (3)	N23—C129—C130	125.3 (15)
O4—Dy1B—O10B	134.9 (4)	N15—C129—C130	125.3 (15)
O19—Dy1B—O10B	66.0 (3)	C90—C129—H129	108.3
O25B—Dy1B—O10B	71.5 (6)	N23—C129—H129	117.4
O22—Dy1B—O10B	89.1 (4)	N15—C129—H129	117.4
O1—Dy1B—O10B	123.2 (4)	C130—C129—H129	117.4
O13—Dy1B—O10B	70.3 (4)	C91—C130—C129	117 (2)
O16—Dy1B—O7	147.69 (12)	C129—C130—C131	116.8 (14)
O4—Dy1B—O7	121.33 (11)	C91—C130—C89	122.4 (17)
O19—Dy1B—O7	107.57 (11)	C131—C130—C89	115.8 (14)
O25B—Dy1B—O7	70.6 (6)	C91—C130—H130	111.6
O22—Dy1B—O7	67.78 (8)	C129—C130—H130	121.6
O1—Dy1B—O7	67.16 (8)	C131—C130—H130	121.6
O13—Dy1B—O7	121.29 (12)	C89—C130—H130	121.5
O10B—Dy1B—O7	56.2 (4)	C91—C131—C130	61 (2)
O16—Dy1B—N6	30.01 (7)	C91—C131—C132	106 (2)
O4—Dy1B—N6	67.78 (9)	C130—C131—C132	119.5 (12)
O19—Dy1B—N6	64.01 (9)	C91—C131—C92	68.7 (17)
O25B—Dy1B—N6	117.4 (7)	C130—C131—C92	103.8 (17)
O22—Dy1B—N6	104.45 (11)	C91—C131—C90	61.1 (17)
O1—Dy1B—N6	116.63 (11)	C132—C131—C90	119.1 (12)
O13—Dy1B—N6	60.35 (9)	C92—C131—C90	103.4 (13)
O10B—Dy1B—N6	120.0 (4)	C91—C131—H131	102.2
O7—Dy1B—N6	170.59 (11)	C130—C131—H131	120.3
O16—Dy1B—Al2	37.46 (7)	C132—C131—H131	120.3
O4—Dy1B—Al2	36.43 (6)	C92—C131—H131	123.7
O19—Dy1B—Al2	114.51 (10)	C90—C131—H131	120.6
O25B—Dy1B—Al2	102.1 (5)	C92—C132—C133	87.0 (15)
O22—Dy1B—Al2	100.76 (9)	C92—C132—C131	77.2 (15)
O1—Dy1B—Al2	63.08 (7)	C133—C132—C131	118.1 (13)
O13—Dy1B—Al2	100.40 (9)	C92—C132—C93	66.0 (12)
O10B—Dy1B—Al2	169.8 (4)	C131—C132—C93	112.2 (12)
O7—Dy1B—Al2	130.06 (9)	C92—C132—C91	53.0 (10)
N6—Dy1B—Al2	55.18 (7)	C133—C132—C91	108.5 (11)
O16—Dy1B—Al5	65.52 (8)	C93—C132—C91	95.5 (10)
O4—Dy1B—Al5	95.39 (9)	C92—C132—H132	105.4
O19—Dy1B—Al5	36.31 (6)	C133—C132—H132	120.9
O25B—Dy1B—Al5	108.2 (8)	C131—C132—H132	120.9
O22—Dy1B—Al5	104.74 (9)	C93—C132—H132	122.7
O1—Dy1B—Al5	155.09 (11)	C91—C132—H132	125.3
O13—Dy1B—Al5	36.96 (6)	C93—C133—N23	80.6 (18)
O10B—Dy1B—Al5	81.1 (3)	C93—C133—N15	80.6 (18)
O7—Dy1B—Al5	136.02 (10)	C93—C133—C132	87 (2)
N6—Dy1B—Al5	38.92 (6)	N23—C133—C132	122.7 (12)
Al2—Dy1B—Al5	93.74 (7)	N15—C133—C132	122.7 (12)
O7—Al4B—O27B	162.6 (9)	C93—C133—C92	52 (2)
O7—Al4B—O11B	95.1 (7)	N23—C133—C92	106.9 (10)
O27B—Al4B—O11B	89.6 (8)	N15—C133—C92	106.9 (10)
O7—Al4B—O10B	86.8 (6)	C93—C133—H133	103.2
O27B—Al4B—O10B	110.5 (9)	N23—C133—H133	118.7
O11B—Al4B—O10B	81.9 (7)	N15—C133—H133	118.7
O7—Al4B—N9B	92.9 (8)	C132—C133—H133	118.7
O27B—Al4B—N9B	86.5 (8)	C92—C133—H133	123.4
O11B—Al4B—N9B	164.9 (11)	C139—N25—C143	120.0
O10B—Al4B—N9B	85.8 (8)	N25—C139—C140	120.0
O7—Al4B—O8	77.2 (4)	N25—C139—H139	120.0
O27B—Al4B—O8	85.4 (7)	C140—C139—H139	120.0
O11B—Al4B—O8	101.8 (6)	C141—C140—C139	120.0
O10B—Al4B—O8	163.9 (7)	C141—C140—H140	120.0
N9B—Al4B—O8	92.4 (8)	C139—C140—H140	120.0
O7—Al4B—Dy1B	47.9 (3)	C140—C141—C142	120.0
O27B—Al4B—Dy1B	144.7 (7)	C140—C141—H141	120.0
O11B—Al4B—Dy1B	109.9 (6)	C142—C141—H141	120.0
O10B—Al4B—Dy1B	47.7 (5)	C143—C142—C141	120.0
N9B—Al4B—Dy1B	66.9 (5)	C143—C142—H142	120.0
O8—Al4B—Dy1B	117.2 (4)	C141—C142—H142	120.0
N4B—O10B—Al4B	107.3 (11)	C142—C143—N25	120.0
N4B—O10B—Dy1B	103.9 (11)	C142—C143—H143	120.0
Al4B—O10B—Dy1B	99.1 (6)	N25—C143—H143	120.0
C22B—N4B—O10B	116.2 (14)	C98—N16—C94	122.1 (3)
C22B—N4B—H4B	107 (10)	C98—N16—H16	119.0
O10B—N4B—H4B	135 (10)	C94—N16—H16	119.0
C22B—O11B—Al4B	110.4 (11)	N16—C94—C95	119.8 (3)
O11B—C22B—N4B	115.8 (14)	N16—C94—H94	120.1
O11B—C22B—C23B	124.6 (15)	C95—C94—H94	120.1
N4B—C22B—C23B	119.4 (14)	C94—C95—C96	118.9 (4)
C24B—C23B—C28B	118.7 (15)	C94—C95—H95	120.6
C24B—C23B—C22B	114.8 (15)	C96—C95—H95	120.6
C28B—C23B—C22B	126.5 (15)	C97—C96—C95	120.1 (4)
C25B—C24B—C23B	120.7 (18)	C97—C96—H96	119.9
C25B—C24B—H24B	119.6	C95—C96—H96	119.9
C23B—C24B—H24B	119.6	C98—C97—C96	118.8 (4)
C26B—C25B—C24B	116.9 (19)	C98—C97—H97	120.6
C26B—C25B—H25B	121.5	C96—C97—H97	120.6
C24B—C25B—H25B	121.5	N16—C98—C97	120.3 (4)
C27B—C26B—C25B	124 (2)	N16—C98—H98	119.9
C27B—C26B—H26B	117.9	C97—C98—H98	119.9
C25B—C26B—H26B	117.9	C99—N17—C103	120.5 (11)
C26B—C27B—C28B	117.5 (17)	N17—C99—C100	121.9 (13)
C26B—C27B—H27B	121.3	N17—C99—H99	119.0
C28B—C27B—H27B	121.3	C100—C99—H99	119.0
O12B—C28B—C23B	116.7 (16)	C101—C100—C99	118.7 (12)
O12B—C28B—C27B	123.5 (17)	C101—C100—H100	120.7
C23B—C28B—C27B	119.8 (15)	C99—C100—H100	120.7
C28B—O12B—Al6	135 (2)	C100—C101—C102	118.7 (12)
C28B—O12B—H12P	109.5	C100—C101—H101	120.6
Al6—O12B—H12P	111.0	C102—C101—H101	120.6
N9B—O25B—Al3	108.6 (11)	C103—C102—C101	117.6 (11)
N9B—O25B—Dy1B	117.9 (12)	C103—C102—H102	121.2
Al3—O25B—Dy1B	128.5 (10)	C101—C102—H102	121.2
C57B—N9B—O25B	110.4 (13)	N17—C103—C102	120.8 (10)
C57B—N9B—Al4B	129.2 (14)	N17—C103—H103	119.6
O25B—N9B—Al4B	118.8 (12)	C102—C103—H103	119.6
C57B—O26B—Al3	114.4 (17)	C134—N24—C138	116.7 (11)
N9B—C57B—O26B	121.9 (17)	N24—C134—C135	123.2 (13)
N9B—C57B—C58B	119.0 (16)	N24—C134—H134	118.4
O26B—C57B—C58B	119.1 (17)	C135—C134—H134	118.4
C59B—C58B—C63B	120.9 (15)	C136—C135—C134	120.3 (12)
C59B—C58B—C57B	118.1 (15)	C136—C135—H135	119.8
C63B—C58B—C57B	121.0 (15)	C134—C135—H135	119.8
C58B—C59B—C60B	122.6 (19)	C135—C136—C137	117.3 (11)
C58B—C59B—H59B	118.7	C135—C136—H136	121.3
C60B—C59B—H59B	118.7	C137—C136—H136	121.3
C61B—C60B—C59B	114.2 (19)	C138—C137—C136	115.2 (12)
C61B—C60B—H60B	122.9	C138—C137—H137	122.4
C59B—C60B—H60B	122.9	C136—C137—H137	122.4
C62B—C61B—C60B	125.4 (19)	N24—C138—C137	123.3 (11)
C62B—C61B—H61B	117.3	N24—C138—H138	118.3
C60B—C61B—H61B	117.3	C137—C138—H138	118.3
C61B—C62B—C63B	119.5 (18)	C108—N18—C104	115.1 (4)
C61B—C62B—H62B	120.3	N18—C104—C105	124.3 (4)
C63B—C62B—H62B	120.3	N18—C104—H104	117.8
O27B—C63B—C62B	118.6 (16)	C105—C104—H104	117.8
O27B—C63B—C58B	124.1 (15)	C106—C105—C104	119.2 (5)
C62B—C63B—C58B	117.2 (16)	C106—C105—H105	120.4
C63B—O27B—Al4B	125.3 (13)	C104—C105—H105	120.4
N5—O13—Al5	110.18 (17)	C105—C106—C107	118.1 (4)
N5—O13—Dy1B	126.82 (19)	C105—C106—H106	121.0
Al5—O13—Dy1B	93.98 (10)	C107—C106—H106	121.0
N5—O13—Dy1	118.87 (17)	C108—C107—C106	116.9 (5)
Al5—O13—Dy1	99.32 (8)	C108—C107—H107	121.6
C29—O14—Al5	112.9 (2)	C106—C107—H107	121.6
C35—O15—H15	109.5	N18—C108—C107	126.4 (5)
N6—O16—Al2	109.30 (15)	N18—C108—H108	116.8
N6—O16—Dy1B	101.85 (16)	C107—C108—H108	116.8
Al2—O16—Dy1B	100.56 (10)	C109—N19—C113	117.6 (4)
N6—O16—Dy1	103.70 (14)	N19—C109—C110	125.4 (4)
Al2—O16—Dy1	100.35 (8)	N19—C109—H109	117.3
C36—O17—Al2	111.57 (18)	C110—C109—H109	117.3
C42—O18—Al5	133.2 (2)	C109—C110—C111	116.6 (4)
N7—O19—Al5	109.63 (15)	C109—C110—H110	121.7
N7—O19—Dy1B	122.37 (15)	C111—C110—H110	121.7
Al5—O19—Dy1B	98.68 (10)	C112—C111—C110	115.9 (4)
N7—O19—Dy1	125.21 (14)	C112—C111—H111	122.1
Al5—O19—Dy1	103.77 (8)	C110—C111—H111	122.1
C43—O20—Al5	111.21 (18)	C111—C112—C113	121.1 (4)
C49—O21—Al6	129.61 (18)	C111—C112—H112	119.4
N8—O22—Al6	111.73 (14)	C113—C112—H112	119.4
N8—O22—Dy1	117.66 (13)	N19—C113—C112	123.4 (5)
Al6—O22—Dy1	129.56 (9)	N19—C113—H113	118.3
N8—O22—Dy1B	121.47 (14)	C112—C113—H113	118.3
Al6—O22—Dy1B	123.55 (10)	C118—N20—C114	117.7 (8)
C50—O23—Al6	112.48 (16)	N20—C114—C115	122.2 (7)
C56—O24—Al1	130.43 (17)	N20—C114—H114	118.9
Al3—O28—H28A	118 (3)	C115—C114—H114	118.9
Al3—O28—H28B	118 (3)	C116—C115—C114	118.3 (7)
H28A—O28—H28B	114 (4)	C116—C115—H115	120.8
Al6—O29—H29A	110 (3)	C114—C115—H115	120.8
Al6—O29—H29B	131 (3)	C115—C116—C117	118.2 (8)
H29A—O29—H29B	107 (4)	C115—C116—H116	120.9
H30A—O30—H30B	97 (10)	C117—C116—H116	120.9
C1—N1—O1	110.5 (2)	C118—C117—C116	120.7 (8)
C1—N1—Al2	130.05 (19)	C118—C117—H117	119.7
O1—N1—Al2	119.47 (15)	C116—C117—H117	119.7
C8B—N2—O4	111.7 (2)	N20—C118—C117	122.9 (8)
C8—N2—O4	111.7 (2)	N20—C118—H118	118.6
C8B—N2—Al3	129.62 (19)	C117—C118—H118	118.6
C8—N2—Al3	129.62 (19)	C119—N21—C123	120.0
O4—N2—Al3	118.02 (16)	N21—C119—C120	120.0
C15—N3—O7	110.4 (2)	N21—C119—H119	120.0
C15—N3—Al1	132.82 (17)	C120—C119—H119	120.0
O7—N3—Al1	116.76 (16)	C119—C120—C121	120.0
C29—N5—O13	117.2 (3)	C119—C120—H120	120.0
C29—N5—H5N	121.4	C121—C120—H120	120.0
O13—N5—H5N	121.4	C122—C121—C120	120.0
C36—N6—O16	110.1 (2)	C122—C121—H121	120.0
C36—N6—Al5	127.27 (19)	C120—C121—H121	120.0
O16—N6—Al5	119.67 (18)	C121—C122—C123	120.0
C36—N6—Dy1B	126.87 (19)	C121—C122—H122	120.0
O16—N6—Dy1B	48.14 (12)	C123—C122—H122	120.0
Al5—N6—Dy1B	80.38 (10)	C122—C123—N21	120.0
C43—N7—O19	111.0 (2)	C122—C123—H123	120.0
C43—N7—Al6	127.9 (2)	N21—C123—H123	120.0
			
O24—Al1—O2—C1	159.64 (18)	C40—C41—C42—C37	−1.2 (7)
O9—Al1—O2—C1	−111.10 (18)	Al5—O20—C43—N7	14.1 (4)
O1—Al1—O2—C1	−10.86 (18)	Al5—O20—C43—C44	−165.6 (2)
N3—Al1—O2—C1	−42.9 (4)	O19—N7—C43—O20	2.8 (4)
N8—Al1—O2—C1	73.13 (18)	Al6—N7—C43—O20	−164.1 (2)
O17—Al2—O3—C7	163.6 (3)	O19—N7—C43—C44	−177.5 (3)
O5—Al2—O3—C7	68.7 (3)	Al6—N7—C43—C44	15.6 (4)
O16—Al2—O3—C7	−114.5 (3)	O20—C43—C44—C45	−4.4 (5)
N1—Al2—O3—C7	−26.6 (3)	N7—C43—C44—C45	175.9 (3)
Dy1B—Al2—O3—C7	−81.7 (3)	O20—C43—C44—C49	175.6 (3)
Dy1—Al2—O3—C7	−76.4 (3)	N7—C43—C44—C49	−4.1 (5)
O17—Al2—O4—N2	−103.32 (15)	C49—C44—C45—C46	−0.2 (5)
O5—Al2—O4—N2	−9.07 (15)	C43—C44—C45—C46	179.8 (4)
O16—Al2—O4—N2	175.86 (16)	C44—C45—C46—C47	−1.0 (6)
N1—Al2—O4—N2	86.81 (15)	C45—C46—C47—C48	1.6 (6)
Dy1B—Al2—O4—N2	146.29 (19)	C46—C47—C48—C49	−1.0 (6)
Dy1—Al2—O4—N2	140.92 (18)	Al6—O21—C49—C48	168.2 (2)
O17—Al2—O4—Dy1B	110.39 (11)	Al6—O21—C49—C44	−13.3 (5)
O5—Al2—O4—Dy1B	−155.36 (11)	C47—C48—C49—O21	178.3 (3)
O16—Al2—O4—Dy1B	29.57 (9)	C47—C48—C49—C44	−0.3 (5)
N1—Al2—O4—Dy1B	−59.48 (10)	C45—C44—C49—O21	−177.6 (3)
O17—Al2—O4—Dy1	115.77 (9)	C43—C44—C49—O21	2.4 (5)
O5—Al2—O4—Dy1	−149.98 (9)	C45—C44—C49—C48	0.8 (5)
O16—Al2—O4—Dy1	34.94 (8)	C43—C44—C49—C48	−179.1 (3)
N1—Al2—O4—Dy1	−54.10 (9)	Al6—O23—C50—N8	1.3 (3)
O3—Al2—O5—C8B	−166.89 (18)	Al6—O23—C50—C51	177.56 (19)
O17—Al2—O5—C8B	101.20 (18)	O22—N8—C50—O23	2.7 (3)
O4—Al2—O5—C8B	8.14 (18)	Al1—N8—C50—O23	−165.81 (19)
O16—Al2—O5—C8B	22.2 (4)	O22—N8—C50—C51	−173.5 (2)
N1—Al2—O5—C8B	−78.21 (19)	Al1—N8—C50—C51	18.0 (4)
Dy1B—Al2—O5—C8B	−12.0 (2)	O23—C50—C51—C52	5.5 (4)
O3—Al2—O5—C8	−166.89 (18)	N8—C50—C51—C52	−178.2 (3)
O17—Al2—O5—C8	101.20 (18)	O23—C50—C51—C56	−167.9 (3)
O4—Al2—O5—C8	8.14 (18)	N8—C50—C51—C56	8.4 (4)
O16—Al2—O5—C8	22.2 (4)	C56—C51—C52—C53	3.7 (6)
N1—Al2—O5—C8	−78.21 (19)	C50—C51—C52—C53	−169.8 (4)
Dy1—Al2—O5—C8	−14.8 (2)	C51—C52—C53—C54	−0.2 (7)
C15—O8—Al4—O27	−164.53 (18)	C52—C53—C54—C55	−2.4 (7)
C15—O8—Al4—O11	108.86 (18)	C53—C54—C55—C56	1.5 (6)
C15—O8—Al4—O7	13.75 (18)	Al1—O24—C56—C51	−22.4 (4)
C15—O8—Al4—O10	13.7 (4)	Al1—O24—C56—C55	160.0 (2)
C15—O8—Al4—N9	−77.9 (2)	C52—C51—C56—O24	177.9 (3)
C15—O8—Al4—Dy1	−8.9 (2)	C50—C51—C56—O24	−8.8 (4)
Al4—O10—N4—C22	3.9 (3)	C52—C51—C56—C55	−4.5 (4)
Dy1—O10—N4—C22	115.5 (3)	C50—C51—C56—C55	168.8 (3)
O8—Al4—O11—C22	−160.6 (2)	C54—C55—C56—O24	179.7 (3)
O27—Al4—O11—C22	108.2 (2)	C54—C55—C56—C51	2.0 (5)
O7—Al4—O11—C22	−77.4 (2)	C68—N10—C64—C65	−0.8 (6)
O10—Al4—O11—C22	−0.2 (2)	Al3—N10—C64—C65	176.9 (4)
N9—Al4—O11—C22	41.2 (5)	N10—C64—C65—C66	1.7 (8)
Dy1—Al4—O11—C22	−39.6 (2)	C64—C65—C66—C67	−1.5 (10)
Al4—O11—C22—N4	2.5 (4)	C65—C66—C67—C68	0.5 (10)
Al4—O11—C22—C23	−172.5 (2)	C64—N10—C68—C67	−0.3 (6)
O10—N4—C22—O11	−4.4 (4)	Al3—N10—C68—C67	−177.9 (4)
O10—N4—C22—C23	170.6 (3)	C66—C67—C68—N10	0.4 (8)
O11—C22—C23—C24	−2.9 (5)	C78—N12—C74—C75	−2.7 (7)
N4—C22—C23—C24	−177.7 (3)	N12—C74—C75—C76	1.8 (7)
O11—C22—C23—C28	173.8 (3)	C74—C75—C76—C77	0.2 (7)
N4—C22—C23—C28	−1.0 (5)	C75—C76—C77—C78	−1.2 (7)
C28—C23—C24—C25	−1.2 (6)	C74—N12—C78—C77	1.6 (6)
C22—C23—C24—C25	175.7 (4)	C76—C77—C78—N12	0.4 (7)
C23—C24—C25—C26	0.0 (7)	C83—N13—C79—C80	−1.8 (6)
C24—C25—C26—C27	1.0 (8)	N13—C79—C80—C81	0.3 (6)
C25—C26—C27—C28	−0.8 (8)	C79—C80—C81—C82	1.3 (7)
C26—C27—C28—O12	−179.8 (5)	C80—C81—C82—C83	−1.4 (8)
C26—C27—C28—C23	−0.4 (7)	C79—N13—C83—C82	1.6 (6)
C24—C23—C28—O12	−179.2 (4)	C81—C82—C83—N13	−0.1 (8)
C22—C23—C28—O12	4.1 (6)	C88—N14—C84—C85	−2 (4)
C24—C23—C28—C27	1.3 (6)	N14—C84—C85—C86	3 (4)
C22—C23—C28—C27	−175.4 (4)	C84—C85—C86—C87	1 (3)
O26—Al3—O25—N9	−13.6 (2)	C85—C86—C87—C88	−6 (2)
N2—Al3—O25—N9	165.7 (2)	C84—N14—C88—C87	−3 (4)
O28—Al3—O25—N9	77.6 (2)	C86—C87—C88—N14	7 (3)
N10—Al3—O25—N9	−103.8 (2)	C128—N22—C124—C125	−1 (4)
O26—Al3—O25—Dy1	−177.4 (2)	N22—C124—C125—C126	5 (4)
N2—Al3—O25—Dy1	1.8 (2)	C124—C125—C126—C127	1 (3)
O28—Al3—O25—Dy1	−86.3 (2)	C125—C126—C127—C128	−12 (3)
N10—Al3—O25—Dy1	92.4 (2)	C124—N22—C128—C127	−11 (4)
Al3—O25—N9—C57	12.3 (3)	C126—C127—C128—N22	17 (3)
Dy1—O25—N9—C57	178.6 (2)	C129—N15—C89—N23	0 (100)
Al3—O25—N9—Al4	−159.20 (15)	C133—N15—C89—N23	0 (100)
Dy1—O25—N9—Al4	7.0 (3)	C93—N15—C89—N23	0 (100)
O6—Al3—O26—C57	−164.0 (3)	C129—N15—C89—C90	−55 (6)
O25—Al3—O26—C57	12.5 (3)	C133—N15—C89—C90	28 (3)
O28—Al3—O26—C57	−73.8 (3)	C93—N15—C89—C90	3 (2)
N10—Al3—O26—C57	104.2 (3)	C129—N15—C89—C130	−60 (7)
Al3—O26—C57—N9	−8.9 (4)	C133—N15—C89—C130	23 (2)
Al3—O26—C57—C58	171.5 (3)	C93—N15—C89—C130	−2 (2)
O25—N9—C57—O26	−2.5 (4)	N23—C89—C90—C129	64 (5)
Al4—N9—C57—O26	167.8 (3)	N15—C89—C90—C129	64 (5)
O25—N9—C57—C58	177.2 (3)	C130—C89—C90—C129	96 (19)
Al4—N9—C57—C58	−12.6 (4)	N23—C89—C90—C91	−1 (3)
O26—C57—C58—C59	−7.7 (5)	N15—C89—C90—C91	−1 (3)
N9—C57—C58—C59	172.7 (3)	C130—C89—C90—C91	31 (16)
O26—C57—C58—C63	170.3 (3)	N23—C89—C90—C131	−30 (3)
N9—C57—C58—C63	−9.4 (5)	N15—C89—C90—C131	−30 (3)
C63—C58—C59—C60	0.5 (6)	C130—C89—C90—C131	2 (16)
C57—C58—C59—C60	178.5 (4)	C129—C90—C91—C131	−91 (2)
C58—C59—C60—C61	0.9 (6)	C89—C90—C91—C131	−80 (2)
C59—C60—C61—C62	−1.2 (7)	C129—C90—C91—C130	−95 (13)
C60—C61—C62—C63	0.2 (6)	C89—C90—C91—C130	−84 (13)
C61—C62—C63—O27	−178.2 (3)	C131—C90—C91—C130	−5 (13)
C61—C62—C63—C58	1.2 (5)	C129—C90—C91—C92	−10 (3)
C59—C58—C63—O27	177.9 (3)	C89—C90—C91—C92	1 (3)
C57—C58—C63—O27	0.0 (5)	C131—C90—C91—C92	80.6 (17)
C59—C58—C63—C62	−1.5 (5)	C129—C90—C91—C132	−43 (2)
C57—C58—C63—C62	−179.5 (3)	C89—C90—C91—C132	−32 (2)
C62—C63—O27—Al4	−147.6 (3)	C131—C90—C91—C132	47.4 (13)
C58—C63—O27—Al4	33.0 (4)	C131—C91—C92—C132	7.1 (19)
O8—Al4—O27—C63	57.2 (3)	C130—C91—C92—C132	−73 (3)
O11—Al4—O27—C63	156.9 (3)	C90—C91—C92—C132	−79 (2)
O10—Al4—O27—C63	−122.2 (2)	C131—C91—C92—C93	83 (2)
N9—Al4—O27—C63	−40.0 (3)	C130—C91—C92—C93	3 (3)
Dy1—Al4—O27—C63	−82.6 (3)	C90—C91—C92—C93	−3 (2)
C73—N11—C69—C70	−1.6 (6)	C132—C91—C92—C93	76.1 (15)
Al6—N11—C69—C70	168.8 (3)	C130—C91—C92—C131	−80 (3)
N11—C69—C70—C71	−1.2 (6)	C90—C91—C92—C131	−86 (2)
C69—C70—C71—C72	2.3 (7)	C132—C91—C92—C131	−7.1 (19)
C70—C71—C72—C73	−0.6 (7)	C131—C91—C92—C133	64 (2)
C69—N11—C73—C72	3.4 (6)	C130—C91—C92—C133	−16 (3)
Al6—N11—C73—C72	−166.8 (3)	C90—C91—C92—C133	−22 (2)
C71—C72—C73—N11	−2.3 (7)	C132—C91—C92—C133	57.0 (12)
N3—O7—Al4B—O27B	−2 (3)	C89—N15—C93—C133	101 (3)
Dy1B—O7—Al4B—O27B	−142 (3)	C129—N15—C93—C133	112 (3)
N3—O7—Al4B—O11B	−107.3 (6)	C89—N15—C93—N23	0 (100)
Dy1B—O7—Al4B—O11B	112.3 (6)	C129—N15—C93—N23	0 (100)
N3—O7—Al4B—O10B	171.1 (5)	C133—N15—C93—N23	0 (100)
Dy1B—O7—Al4B—O10B	30.8 (5)	C89—N15—C93—C92	−5.2 (17)
N3—O7—Al4B—N9B	85.5 (8)	C129—N15—C93—C92	5.4 (19)
Dy1B—O7—Al4B—N9B	−54.9 (7)	C133—N15—C93—C92	−107 (3)
N3—O7—Al4B—O8	−6.4 (4)	C89—N15—C93—C132	39.1 (15)
Dy1B—O7—Al4B—O8	−146.73 (15)	C129—N15—C93—C132	49.7 (17)
N3—O7—Al4B—Dy1B	140.3 (4)	C133—N15—C93—C132	−62 (2)
Al4B—O10B—N4B—C22B	9 (2)	C132—C92—C93—C133	6 (3)
Dy1B—O10B—N4B—C22B	113.7 (18)	C91—C92—C93—C133	−83 (3)
Al4B—O11B—C22B—N4B	−29 (3)	C131—C92—C93—C133	−51 (3)
Al4B—O11B—C22B—C23B	156 (2)	C132—C92—C93—N15	93.2 (17)
O10B—N4B—C22B—O11B	13 (3)	C91—C92—C93—N15	5 (2)
O10B—N4B—C22B—C23B	−172 (2)	C131—C92—C93—N15	37.1 (17)
O11B—C22B—C23B—C24B	19 (4)	C133—C92—C93—N15	88 (3)
N4B—C22B—C23B—C24B	−156 (3)	C132—C92—C93—N23	93.2 (17)
O11B—C22B—C23B—C28B	−158 (3)	C91—C92—C93—N23	5 (2)
N4B—C22B—C23B—C28B	27 (4)	C131—C92—C93—N23	37.1 (17)
C28B—C23B—C24B—C25B	−7 (5)	C133—C92—C93—N23	88 (3)
C22B—C23B—C24B—C25B	176 (3)	C91—C92—C93—C132	−88.1 (16)
C23B—C24B—C25B—C26B	16 (5)	C131—C92—C93—C132	−56.1 (13)
C24B—C25B—C26B—C27B	−17 (5)	C133—C92—C93—C132	−6 (3)
C25B—C26B—C27B—C28B	7 (5)	N15—C89—N23—C129	0 (100)
C24B—C23B—C28B—O12B	178 (3)	C90—C89—N23—C129	−55 (6)
C22B—C23B—C28B—O12B	−5 (5)	C130—C89—N23—C129	−60 (7)
C24B—C23B—C28B—C27B	−3 (5)	N15—C89—N23—C133	0 (100)
C22B—C23B—C28B—C27B	174 (3)	C90—C89—N23—C133	28 (3)
C26B—C27B—C28B—O12B	−178 (3)	C130—C89—N23—C133	23 (2)
C26B—C27B—C28B—C23B	3 (4)	N15—C89—N23—C93	0 (40)
C23B—C28B—O12B—Al6	104 (3)	C90—C89—N23—C93	3 (2)
C27B—C28B—O12B—Al6	−75 (4)	C130—C89—N23—C93	−2 (2)
Al3—O25B—N9B—C57B	17 (3)	C133—C93—N23—C89	101 (3)
Dy1B—O25B—N9B—C57B	174 (2)	N15—C93—N23—C89	0 (100)
Al3—O25B—N9B—Al4B	−150.2 (14)	C92—C93—N23—C89	−5.2 (17)
Dy1B—O25B—N9B—Al4B	7 (3)	C132—C93—N23—C89	39.1 (15)
O6B—Al3—O26B—C57B	−164 (2)	C133—C93—N23—C129	112 (3)
N2—Al3—O26B—C57B	30 (6)	N15—C93—N23—C129	0 (67)
O28—Al3—O26B—C57B	−73 (3)	C92—C93—N23—C129	5.4 (19)
O25B—Al3—O26B—C57B	19 (3)	C132—C93—N23—C129	49.7 (17)
N10—Al3—O26B—C57B	105 (3)	N15—C93—N23—C133	0 (100)
O25B—N9B—C57B—O26B	−2 (5)	C92—C93—N23—C133	−107 (3)
Al4B—N9B—C57B—O26B	163 (3)	C132—C93—N23—C133	−62 (2)
O25B—N9B—C57B—C58B	176 (3)	C89—C90—C129—N23	−92 (7)
Al4B—N9B—C57B—C58B	−19 (5)	C91—C90—C129—N23	27 (4)
Al3—O26B—C57B—N9B	−16 (5)	C131—C90—C129—N23	−3 (4)
Al3—O26B—C57B—C58B	166 (2)	C89—C90—C129—N15	−92 (7)
N9B—C57B—C58B—C59B	176 (3)	C91—C90—C129—N15	27 (4)
O26B—C57B—C58B—C59B	−6 (5)	C131—C90—C129—N15	−3 (4)
N9B—C57B—C58B—C63B	−5 (5)	C89—C90—C129—C130	−88 (19)
O26B—C57B—C58B—C63B	173 (3)	C91—C90—C129—C130	31 (16)
C63B—C58B—C59B—C60B	0 (5)	C131—C90—C129—C130	1 (16)
C57B—C58B—C59B—C60B	179 (3)	C89—N23—C129—C90	101 (8)
C58B—C59B—C60B—C61B	4 (5)	C133—N23—C129—C90	−2 (4)
C59B—C60B—C61B—C62B	−6 (5)	C93—N23—C129—C90	−25 (4)
C60B—C61B—C62B—C63B	3 (5)	C89—N23—C129—N15	0 (100)
C61B—C62B—C63B—O27B	−179 (3)	C133—N23—C129—N15	0 (100)
C61B—C62B—C63B—C58B	1 (4)	C93—N23—C129—N15	0 (100)
C59B—C58B—C63B—O27B	177 (3)	C89—N23—C129—C130	100 (8)
C57B—C58B—C63B—O27B	−2 (4)	C133—N23—C129—C130	−3 (4)
C59B—C58B—C63B—C62B	−3 (4)	C93—N23—C129—C130	−26 (4)
C57B—C58B—C63B—C62B	178 (3)	C89—N15—C129—C90	101 (8)
C62B—C63B—O27B—Al4B	−147 (2)	C133—N15—C129—C90	−2 (4)
C58B—C63B—O27B—Al4B	33 (4)	C93—N15—C129—C90	−25 (4)
O7—Al4B—O27B—C63B	48 (4)	C89—N15—C129—N23	0 (100)
O11B—Al4B—O27B—C63B	154 (2)	C133—N15—C129—N23	0 (100)
O10B—Al4B—O27B—C63B	−125.0 (19)	C93—N15—C129—N23	0 (100)
N9B—Al4B—O27B—C63B	−41 (2)	C89—N15—C129—C130	100 (8)
O8—Al4B—O27B—C63B	52 (2)	C133—N15—C129—C130	−3 (4)
Dy1B—Al4B—O27B—C63B	−81 (2)	C93—N15—C129—C130	−26 (4)
O3—Al2—O17—C36	122.5 (2)	C131—C91—C130—C129	−99 (3)
O5—Al2—O17—C36	−139.88 (19)	C90—C91—C130—C129	77 (14)
O4—Al2—O17—C36	−57.12 (19)	C92—C91—C130—C129	−21 (4)
O16—Al2—O17—C36	20.10 (19)	C132—C91—C130—C129	−52 (3)
N1—Al2—O17—C36	36.8 (7)	C90—C91—C130—C131	175 (13)
Dy1B—Al2—O17—C36	−14.1 (2)	C92—C91—C130—C131	77.6 (19)
Dy1—Al2—O17—C36	−16.5 (2)	C132—C91—C130—C131	46.1 (13)
O20—Al5—O18—C42	−104.6 (3)	C131—C91—C130—C89	−88 (3)
O13—Al5—O18—C42	79.5 (3)	C90—C91—C130—C89	87 (14)
O14—Al5—O18—C42	161.2 (3)	C92—C91—C130—C89	−11 (4)
N6—Al5—O18—C42	−4.7 (3)	C132—C91—C130—C89	−42 (4)
Dy1B—Al5—O18—C42	39.5 (4)	C90—C129—C130—C91	−141 (20)
Dy1—Al5—O18—C42	46.3 (4)	N23—C129—C130—C91	35 (5)
O18—Al5—O20—C43	158.6 (2)	N15—C129—C130—C91	35 (5)
O19—Al5—O20—C43	−18.7 (2)	C90—C129—C130—C131	−178 (20)
O13—Al5—O20—C43	−36.6 (5)	N23—C129—C130—C131	−2 (5)
O14—Al5—O20—C43	−110.7 (2)	N15—C129—C130—C131	−2 (5)
N6—Al5—O20—C43	68.1 (2)	C90—C129—C130—C89	94 (19)
Dy1B—Al5—O20—C43	6.4 (3)	N23—C129—C130—C89	−89 (6)
Dy1—Al5—O20—C43	0.6 (2)	N15—C129—C130—C89	−89 (6)
O23—Al6—O21—C49	−163.4 (3)	N23—C89—C130—C91	11 (4)
N7—Al6—O21—C49	18.0 (3)	N15—C89—C130—C91	11 (4)
O29—Al6—O21—C49	−70.1 (3)	C90—C89—C130—C91	−141 (20)
O12B—Al6—O21—C49	91.7 (6)	N23—C89—C130—C129	71 (5)
N11—Al6—O21—C49	108.9 (3)	N15—C89—C130—C129	71 (5)
O23—Al6—O22—N8	4.84 (17)	C90—C89—C130—C129	−80 (18)
N7—Al6—O22—N8	−177.05 (17)	N23—C89—C130—C131	−27 (4)
O29—Al6—O22—N8	−88.85 (17)	N15—C89—C130—C131	−27 (4)
O12B—Al6—O22—N8	109.3 (6)	C90—C89—C130—C131	−178 (20)
N11—Al6—O22—N8	92.04 (18)	C90—C91—C131—C130	−0.4 (12)
O23—Al6—O22—Dy1	172.68 (14)	C92—C91—C131—C130	−120.4 (11)
N7—Al6—O22—Dy1	−9.21 (15)	C132—C91—C131—C130	−115.5 (14)
O29—Al6—O22—Dy1	78.99 (14)	C130—C91—C131—C132	115.5 (14)
N11—Al6—O22—Dy1	−100.12 (14)	C90—C91—C131—C132	115.1 (14)
O23—Al6—O22—Dy1B	164.68 (15)	C92—C91—C131—C132	−4.9 (13)
N7—Al6—O22—Dy1B	−17.22 (15)	C130—C91—C131—C92	120.4 (11)
O29—Al6—O22—Dy1B	70.98 (14)	C90—C91—C131—C92	120.0 (9)
O12B—Al6—O22—Dy1B	−90.9 (6)	C132—C91—C131—C92	4.9 (13)
O21—Al6—O23—C50	172.04 (19)	C130—C91—C131—C90	0.4 (12)
O22—Al6—O23—C50	−3.49 (19)	C92—C91—C131—C90	−120.0 (8)
O29—Al6—O23—C50	84.26 (19)	C132—C91—C131—C90	−115.1 (14)
O12B—Al6—O23—C50	−96.2 (6)	C129—C130—C131—C91	99 (4)
N11—Al6—O23—C50	−99.6 (2)	C89—C130—C131—C91	110 (3)
O2—Al1—O24—C56	−59.7 (2)	C91—C130—C131—C132	−92 (2)
O9—Al1—O24—C56	−148.4 (2)	C129—C130—C131—C132	6 (5)
N3—Al1—O24—C56	127.3 (2)	C89—C130—C131—C132	18 (4)
N8—Al1—O24—C56	35.4 (2)	C91—C130—C131—C92	−55.8 (16)
Al1—O1—N1—C1	−11.5 (2)	C129—C130—C131—C92	43 (4)
Dy1—O1—N1—C1	−145.93 (17)	C89—C130—C131—C92	55 (3)
Dy1B—O1—N1—C1	−146.21 (18)	C132—C92—C131—C91	−172 (2)
Al1—O1—N1—Al2	168.97 (10)	C93—C92—C131—C91	−110.4 (18)
Dy1—O1—N1—Al2	34.53 (18)	C133—C92—C131—C91	−124.0 (17)
Dy1B—O1—N1—Al2	34.25 (18)	C132—C92—C131—C130	−121 (2)
Al2—O4—N2—C8B	8.4 (2)	C93—C92—C131—C130	−59 (2)
Dy1B—O4—N2—C8B	138.5 (2)	C91—C92—C131—C130	51 (2)
Al2—O4—N2—C8	8.4 (2)	C133—C92—C131—C130	−72.8 (18)
Dy1—O4—N2—C8	139.11 (18)	C93—C92—C131—C132	61.8 (13)
Al2—O4—N2—Al3	−163.43 (11)	C91—C92—C131—C132	172 (2)
Dy1B—O4—N2—Al3	−33.4 (3)	C133—C92—C131—C132	48.2 (11)
Dy1—O4—N2—Al3	−32.8 (2)	C132—C92—C131—C90	−120.9 (16)
Al4B—O7—N3—C15	4.4 (5)	C93—C92—C131—C90	−59.1 (15)
Al4—O7—N3—C15	8.0 (2)	C91—C92—C131—C90	51.2 (14)
Dy1—O7—N3—C15	137.73 (17)	C133—C92—C131—C90	−72.7 (13)
Dy1B—O7—N3—C15	140.36 (18)	C129—C90—C131—C91	99 (2)
Al4B—O7—N3—Al1	−175.1 (4)	C89—C90—C131—C91	111 (2)
Al4—O7—N3—Al1	−171.49 (10)	C129—C90—C131—C132	7 (3)
Dy1—O7—N3—Al1	−41.77 (19)	C89—C90—C131—C132	19 (3)
Dy1B—O7—N3—Al1	−39.14 (19)	C91—C90—C131—C132	−92 (2)
Al5—O13—N5—C29	6.4 (3)	C129—C90—C131—C92	43 (2)
Dy1B—O13—N5—C29	−105.2 (3)	C89—C90—C131—C92	55 (2)
Dy1—O13—N5—C29	−107.2 (3)	C91—C90—C131—C92	−56.1 (15)
Al2—O16—N6—C36	16.3 (3)	C93—C92—C132—C133	−1.9 (9)
Dy1B—O16—N6—C36	122.0 (2)	C91—C92—C132—C133	115.8 (10)
Dy1—O16—N6—C36	122.6 (2)	C131—C92—C132—C133	119.7 (12)
Al2—O16—N6—Al5	−145.54 (13)	C93—C92—C132—C131	−121.7 (10)
Dy1B—O16—N6—Al5	−39.77 (19)	C91—C92—C132—C131	−3.9 (10)
Dy1—O16—N6—Al5	−39.21 (18)	C133—C92—C132—C131	−119.7 (12)
Al2—O16—N6—Dy1B	−105.78 (15)	C91—C92—C132—C93	117.8 (10)
Al5—O19—N7—C43	−17.7 (3)	C131—C92—C132—C93	121.7 (10)
Dy1B—O19—N7—C43	−132.3 (2)	C133—C92—C132—C93	1.9 (9)
Dy1—O19—N7—C43	−141.9 (2)	C93—C92—C132—C91	−117.8 (10)
Al5—O19—N7—Al6	150.36 (13)	C131—C92—C132—C91	3.9 (10)
Dy1B—O19—N7—Al6	35.8 (3)	C133—C92—C132—C91	−115.8 (10)
Dy1—O19—N7—Al6	26.2 (3)	C91—C131—C132—C92	8 (2)
Al6—O22—N8—C50	−5.2 (3)	C130—C131—C132—C92	73 (3)
Dy1—O22—N8—C50	−174.68 (17)	C90—C131—C132—C92	73 (2)
Dy1B—O22—N8—C50	−165.56 (18)	C91—C131—C132—C133	−72 (3)
Al6—O22—N8—Al1	163.58 (12)	C130—C131—C132—C133	−7 (4)
Dy1—O22—N8—Al1	−5.9 (2)	C92—C131—C132—C133	−79.5 (19)
Dy1B—O22—N8—Al1	3.3 (3)	C90—C131—C132—C133	−7 (3)
Al1—O2—C1—N1	7.5 (3)	C91—C131—C132—C93	−50 (3)
Al1—O2—C1—C2	−172.67 (19)	C130—C131—C132—C93	16 (3)
O1—N1—C1—O2	2.8 (3)	C92—C131—C132—C93	−57.1 (14)
Al2—N1—C1—O2	−177.72 (18)	C90—C131—C132—C93	16 (3)
O1—N1—C1—C2	−177.0 (2)	C130—C131—C132—C91	65 (3)
Al2—N1—C1—C2	2.4 (4)	C92—C131—C132—C91	−8 (2)
O2—C1—C2—C3	−11.8 (4)	C90—C131—C132—C91	65 (2)
N1—C1—C2—C3	168.1 (3)	C133—C93—C132—C92	−175 (3)
O2—C1—C2—C7	165.2 (3)	N15—C93—C132—C92	−109.6 (14)
N1—C1—C2—C7	−15.0 (4)	N23—C93—C132—C92	−109.6 (14)
C7—C2—C3—C4	−1.9 (5)	N15—C93—C132—C133	65 (2)
C1—C2—C3—C4	175.1 (3)	N23—C93—C132—C133	65 (2)
C2—C3—C4—C5	−0.6 (5)	C92—C93—C132—C133	175 (3)
C3—C4—C5—C6	2.1 (6)	C133—C93—C132—C131	−111 (3)
C4—C5—C6—C7	−1.0 (6)	N15—C93—C132—C131	−46 (2)
Al2—O3—C7—C6	−159.6 (2)	N23—C93—C132—C131	−46 (2)
Al2—O3—C7—C2	22.5 (4)	C92—C93—C132—C131	63.7 (16)
C5—C6—C7—O3	−179.5 (3)	C133—C93—C132—C91	−129 (2)
C5—C6—C7—C2	−1.5 (5)	N15—C93—C132—C91	−64.4 (13)
C3—C2—C7—O3	−179.1 (3)	N23—C93—C132—C91	−64.4 (13)
C1—C2—C7—O3	4.0 (4)	C92—C93—C132—C91	45.3 (9)
C3—C2—C7—C6	3.0 (4)	C131—C91—C132—C92	−171 (2)
C1—C2—C7—C6	−174.0 (3)	C130—C91—C132—C92	120 (2)
Al2—O5—C8—N2	−5.8 (3)	C90—C91—C132—C92	115.3 (17)
Al2—O5—C8—C9	175.9 (8)	C131—C91—C132—C133	118 (2)
O4—N2—C8—O5	−1.8 (3)	C130—C91—C132—C133	48 (2)
Al3—N2—C8—O5	168.92 (18)	C90—C91—C132—C133	44 (2)
O4—N2—C8—C9	176.6 (8)	C92—C91—C132—C133	−71.4 (15)
Al3—N2—C8—C9	−12.7 (8)	C130—C91—C132—C131	−70 (2)
O5—C8—C9—C10	−2.9 (16)	C90—C91—C132—C131	−74 (2)
N2—C8—C9—C10	178.8 (8)	C92—C91—C132—C131	171 (2)
O5—C8—C9—C14	174.3 (9)	C131—C91—C132—C93	135 (2)
N2—C8—C9—C14	−4.0 (16)	C130—C91—C132—C93	65 (2)
C14—C9—C10—C11	0.4 (16)	C90—C91—C132—C93	61.0 (16)
C8—C9—C10—C11	177.7 (8)	C92—C91—C132—C93	−54.3 (12)
C9—C10—C11—C12	−0.2 (10)	N15—C93—C133—N23	0.000 (1)
C10—C11—C12—C13	−0.5 (6)	C92—C93—C133—N23	120.2 (13)
C11—C12—C13—C14	0.9 (7)	C132—C93—C133—N23	123.9 (10)
C12—C13—C14—O6	179.7 (4)	N23—C93—C133—N15	0.000 (1)
C12—C13—C14—C9	−0.7 (9)	C92—C93—C133—N15	120.2 (13)
C10—C9—C14—O6	179.6 (8)	C132—C93—C133—N15	123.9 (10)
C8—C9—C14—O6	2.5 (17)	N15—C93—C133—C132	−123.9 (10)
C10—C9—C14—C13	0.0 (16)	N23—C93—C133—C132	−123.9 (10)
C8—C9—C14—C13	−177.1 (9)	C92—C93—C133—C132	−3.6 (17)
C13—C14—O6—Al3	−163.9 (3)	N15—C93—C133—C92	−120.2 (13)
C9—C14—O6—Al3	16.6 (9)	N23—C93—C133—C92	−120.2 (13)
O26—Al3—O6—C14	154.8 (3)	C132—C93—C133—C92	3.6 (17)
N2—Al3—O6—C14	−24.1 (3)	C89—N23—C133—C93	−91 (3)
O28—Al3—O6—C14	64.0 (3)	C129—N23—C133—C93	−78 (3)
N10—Al3—O6—C14	−114.8 (3)	C89—N23—C133—N15	0 (100)
Al2—O5—C8B—N2	−5.8 (3)	C129—N23—C133—N15	0 (100)
Al2—O5—C8B—C9B	172 (7)	C93—N23—C133—N15	0 (100)
O4—N2—C8B—O5	−1.8 (3)	C89—N23—C133—C132	−11 (2)
Al3—N2—C8B—O5	168.92 (18)	C129—N23—C133—C132	2 (3)
O4—N2—C8B—C9B	−180 (7)	C93—N23—C133—C132	80 (3)
Al3—N2—C8B—C9B	−9 (7)	C89—N23—C133—C92	−45.5 (17)
O5—C8B—C9B—C10B	−10 (14)	C129—N23—C133—C92	−32 (2)
N2—C8B—C9B—C10B	168 (7)	C93—N23—C133—C92	45.2 (19)
O5—C8B—C9B—C14B	−177 (8)	C89—N15—C133—C93	−91 (3)
N2—C8B—C9B—C14B	1 (14)	C129—N15—C133—C93	−78 (3)
C14B—C9B—C10B—C11B	−8 (15)	C89—N15—C133—N23	0 (100)
C8B—C9B—C10B—C11B	−176 (7)	C129—N15—C133—N23	0 (100)
C9B—C10B—C11B—C12B	7 (9)	C93—N15—C133—N23	0 (100)
C10B—C11B—C12B—C13B	−4 (6)	C89—N15—C133—C132	−11 (2)
C11B—C12B—C13B—C14B	3 (6)	C129—N15—C133—C132	2 (3)
C12B—C13B—C14B—O6B	−175 (4)	C93—N15—C133—C132	80 (3)
C12B—C13B—C14B—C9B	−4 (9)	C89—N15—C133—C92	−45.5 (17)
C10B—C9B—C14B—O6B	176 (8)	C129—N15—C133—C92	−32 (2)
C8B—C9B—C14B—O6B	−16 (15)	C93—N15—C133—C92	45.2 (19)
C10B—C9B—C14B—C13B	7 (14)	C92—C132—C133—C93	5 (2)
C8B—C9B—C14B—C13B	174 (8)	C131—C132—C133—C93	79 (3)
C13B—C14B—O6B—Al3	−150 (3)	C91—C132—C133—C93	54 (3)
C9B—C14B—O6B—Al3	40 (8)	C92—C132—C133—N23	−72 (2)
O26B—Al3—O6B—C14B	147 (3)	C131—C132—C133—N23	2 (3)
N2—Al3—O6B—C14B	−37 (2)	C93—C132—C133—N23	−77 (2)
O28—Al3—O6B—C14B	51 (2)	C91—C132—C133—N23	−22 (2)
N10—Al3—O6B—C14B	−127 (2)	C92—C132—C133—N15	−72 (2)
Al4—O8—C15—N3	−13.8 (3)	C131—C132—C133—N15	2 (3)
Al4B—O8—C15—N3	−7.3 (4)	C93—C132—C133—N15	−77 (2)
Al4—O8—C15—C16	164.02 (19)	C91—C132—C133—N15	−22 (2)
Al4B—O8—C15—C16	170.5 (3)	C131—C132—C133—C92	73.8 (19)
O7—N3—C15—O8	3.6 (3)	C93—C132—C133—C92	−5 (2)
Al1—N3—C15—O8	−177.00 (18)	C91—C132—C133—C92	49.3 (10)
O7—N3—C15—C16	−174.3 (2)	C132—C92—C133—C93	−174 (3)
Al1—N3—C15—C16	5.1 (4)	C91—C92—C133—C93	106 (3)
O8—C15—C16—C17	11.8 (4)	C131—C92—C133—C93	133 (3)
N3—C15—C16—C17	−170.3 (2)	C132—C92—C133—N23	123.3 (18)
O8—C15—C16—C21	−164.7 (2)	C93—C92—C133—N23	−63 (2)
N3—C15—C16—C21	13.2 (4)	C91—C92—C133—N23	43.0 (17)
C21—C16—C17—C18	−2.6 (4)	C131—C92—C133—N23	70.0 (14)
C15—C16—C17—C18	−179.2 (3)	C132—C92—C133—N15	123.3 (18)
C16—C17—C18—C19	0.1 (5)	C93—C92—C133—N15	−63 (2)
C17—C18—C19—C20	1.3 (5)	C91—C92—C133—N15	43.0 (17)
C18—C19—C20—C21	0.0 (4)	C131—C92—C133—N15	70.0 (14)
Al1—O9—C21—C20	144.77 (19)	C93—C92—C133—C132	174 (3)
Al1—O9—C21—C16	−37.4 (3)	C91—C92—C133—C132	−80.3 (16)
C19—C20—C21—O9	175.4 (2)	C131—C92—C133—C132	−53.3 (13)
C19—C20—C21—C16	−2.5 (4)	C143—N25—C139—C140	0.0
C17—C16—C21—O9	−174.1 (2)	N25—C139—C140—C141	0.0
C15—C16—C21—O9	2.3 (4)	C139—C140—C141—C142	0.0
C17—C16—C21—C20	3.8 (4)	C140—C141—C142—C143	0.0
C15—C16—C21—C20	−179.8 (2)	C141—C142—C143—N25	0.0
Al5—O14—C29—N5	−0.6 (4)	C139—N25—C143—C142	0.0
Al5—O14—C29—C30	−178.7 (2)	C98—N16—C94—C95	0.4 (6)
O13—N5—C29—O14	−4.0 (4)	N16—C94—C95—C96	−1.1 (6)
O13—N5—C29—C30	174.2 (3)	C94—C95—C96—C97	1.2 (8)
O14—C29—C30—C31	6.9 (5)	C95—C96—C97—C98	−0.5 (9)
N5—C29—C30—C31	−171.1 (3)	C94—N16—C98—C97	0.2 (7)
O14—C29—C30—C35	−176.0 (3)	C96—C97—C98—N16	−0.2 (9)
N5—C29—C30—C35	5.9 (5)	C103—N17—C99—C100	−2 (3)
C35—C30—C31—C32	1.6 (6)	N17—C99—C100—C101	−3 (3)
C29—C30—C31—C32	178.8 (4)	C99—C100—C101—C102	12 (2)
C30—C31—C32—C33	−1.7 (7)	C100—C101—C102—C103	−16 (2)
C31—C32—C33—C34	0.9 (8)	C99—N17—C103—C102	−3 (2)
C32—C33—C34—C35	−0.2 (8)	C101—C102—C103—N17	12 (2)
C33—C34—C35—O15	178.8 (5)	C138—N24—C134—C135	6 (3)
C33—C34—C35—C30	0.1 (7)	N24—C134—C135—C136	2 (3)
C31—C30—C35—O15	−179.6 (4)	C134—C135—C136—C137	−18 (2)
C29—C30—C35—O15	3.4 (6)	C135—C136—C137—C138	24 (2)
C31—C30—C35—C34	−0.8 (6)	C134—N24—C138—C137	2 (2)
C29—C30—C35—C34	−177.9 (4)	C136—C137—C138—N24	−17 (2)
Al2—O17—C36—N6	−17.1 (3)	C108—N18—C104—C105	−1.4 (8)
Al2—O17—C36—C37	164.3 (2)	N18—C104—C105—C106	1.2 (8)
O16—N6—C36—O17	−0.1 (4)	C104—C105—C106—C107	−0.6 (8)
Al5—N6—C36—O17	160.0 (2)	C105—C106—C107—C108	0.4 (9)
Dy1B—N6—C36—O17	52.1 (4)	C104—N18—C108—C107	1.2 (9)
O16—N6—C36—C37	178.5 (2)	C106—C107—C108—N18	−0.7 (11)
Al5—N6—C36—C37	−21.4 (4)	C113—N19—C109—C110	1.6 (8)
Dy1B—N6—C36—C37	−129.4 (2)	N19—C109—C110—C111	−2.8 (8)
O17—C36—C37—C38	8.3 (4)	C109—C110—C111—C112	1.5 (7)
N6—C36—C37—C38	−170.3 (3)	C110—C111—C112—C113	0.8 (6)
O17—C36—C37—C42	−171.4 (3)	C109—N19—C113—C112	0.9 (7)
N6—C36—C37—C42	10.0 (5)	C111—C112—C113—N19	−2.1 (7)
C42—C37—C38—C39	−1.5 (6)	C118—N20—C114—C115	0.0 (10)
C36—C37—C38—C39	178.8 (4)	N20—C114—C115—C116	1.2 (11)
C37—C38—C39—C40	0.3 (7)	C114—C115—C116—C117	−0.5 (10)
C38—C39—C40—C41	0.4 (8)	C115—C116—C117—C118	−1.4 (11)
C39—C40—C41—C42	0.1 (8)	C114—N20—C118—C117	−2.0 (11)
Al5—O18—C42—C37	−3.2 (5)	C116—C117—C118—N20	2.7 (12)
Al5—O18—C42—C41	178.1 (3)	C123—N21—C119—C120	0.0
C38—C37—C42—O18	−176.9 (3)	N21—C119—C120—C121	0.0
C36—C37—C42—O18	2.8 (5)	C119—C120—C121—C122	0.0
C38—C37—C42—C41	1.8 (5)	C120—C121—C122—C123	0.0
C36—C37—C42—C41	−178.4 (3)	C121—C122—C123—N21	0.0
C40—C41—C42—O18	177.6 (4)	C119—N21—C123—C122	0.0

### Bis(pyridinium) diaquadipyridinehexakis[µ3-salicylhydroximato(3-)]bis[µ2-salicylhydroximato(1-)]hexaaluminiumdysprosium–pyridine–water (1/7.429/1) (2) Hydrogen-bond geometry (Å, º)

**Table:**

*D*—H···*A*	*D*—H	H···*A*	*D*···*A*	*D*—H···*A*
N4—H4*N*···O12	0.88	1.94	2.593 (4)	129
O12—H12*O*···N15	0.84	1.82	2.609 (5)	156
C62—H62···O17^i^	0.95	2.52	3.334 (4)	143
O15—H15···N14	0.84	1.89	2.713 (16)	165
O15—H15···N22	0.84	1.71	2.529 (17)	166
O28—H28*A*···N13	0.84 (2)	1.80 (2)	2.629 (3)	174 (4)
O29—H29*A*···O16	0.89 (2)	1.85 (2)	2.712 (3)	163 (4)
O29—H29*B*···N18	0.91 (2)	1.89 (3)	2.703 (4)	148 (4)
O30—H30*A*···N19	0.86 (2)	2.10 (2)	2.822 (7)	141 (4)
O30—H30*B*···N20	0.87 (2)	2.17 (2)	3.029 (12)	167 (10)
N5—H5*N*···O15	0.88	1.96	2.624 (4)	131
C68—H68···O4	0.95	2.29	3.073 (4)	140
N12—H12*A*···O9	0.88	1.79	2.625 (3)	157
C74—H74···O28	0.95	2.47	3.396 (4)	164
C130—H130···O18^ii^	0.95	2.52	3.36 (2)	149
N16—H16···O27	0.88	1.77	2.634 (4)	168
C94—H94···O3^i^	0.95	2.28	3.131 (4)	149

Symmetry codes: (i) *x*+1, *y*, *z*; (ii) −*x*, −*y*+1, −*z*+1.

### Bis(pyridinium) diaquadipyridinehexakis[µ3-salicylhydroximato(3-)]bis[µ2-salicylhydroximato(1-)]hexaaluminiumytterbium–pyridine–water (1/7.386/1) (3) Crystal data

**Table:**

(C_5_H_6_N)_2_[YbAl_6_(C_7_H_6_NO_3_)_2_(C_7_H_4_NO_3_)_7_(C_5_H_5_N)_1.855_(H_2_O)_2_]·7.386C_5_H_5_N·H_2_O	*Z* = 2
*M**_r_* = 2632.26	*F*(000) = 2695.1
Triclinic, *P*1	*D*_x_ = 1.499 Mg m^−^^3^
*a* = 13.7254 (6) Å	Mo *K*α radiation, λ = 0.71073 Å
*b* = 16.0597 (8) Å	Cell parameters from 9673 reflections
*c* = 28.2523 (12) Å	θ = 2.4–30.6°
α = 80.5334 (16)°	µ = 0.94 mm^−^^1^
β = 83.7992 (17)°	*T* = 150 K
γ = 72.0307 (17)°	Block, colourless
*V* = 5832.2 (5) Å^3^	0.21 × 0.20 × 0.16 mm

### Bis(pyridinium) diaquadipyridinehexakis[µ3-salicylhydroximato(3-)]bis[µ2-salicylhydroximato(1-)]hexaaluminiumytterbium–pyridine–water (1/7.386/1) (3) Data collection

**Table:**

Bruker AXS D8 Quest CMOS diffractometer	33129 independent reflections
Radiation source: fine focus sealed tube X-ray source	26948 reflections with *I* > 2σ(*I*)
Triumph curved graphite crystal monochromator	*R*_int_ = 0.043
Detector resolution: 10.4167 pixels mm^-1^	θ_max_ = 30.6°, θ_min_ = 2.4°
ω and phi scans	*h* = −16→19
Absorption correction: multi-scan (SADABS; Krause *et al.*, 2015)	*k* = −22→22
*T*_min_ = 0.226, *T*_max_ = 0.266	*l* = −40→39
103008 measured reflections	

### Bis(pyridinium) diaquadipyridinehexakis[µ3-salicylhydroximato(3-)]bis[µ2-salicylhydroximato(1-)]hexaaluminiumytterbium–pyridine–water (1/7.386/1) (3) Refinement

**Table:**

Refinement on *F*^2^	Primary atom site location: structure-invariant direct methods
Least-squares matrix: full	Secondary atom site location: difference Fourier map
*R*[*F*^2^ > 2σ(*F*^2^)] = 0.048	Hydrogen site location: mixed
*wR*(*F*^2^) = 0.126	H atoms treated by a mixture of independent and constrained refinement
*S* = 1.03	*w* = 1/[σ^2^(*F*_o_^2^) + (0.0572*P*)^2^ + 8.2486*P*] where *P* = (*F*_o_^2^ + 2*F*_c_^2^)/3
33129 reflections	(Δ/σ)_max_ = 0.001
2113 parameters	Δρ_max_ = 1.27 e Å^−^^3^
2126 restraints	Δρ_min_ = −1.15 e Å^−^^3^

### Bis(pyridinium) diaquadipyridinehexakis[µ3-salicylhydroximato(3-)]bis[µ2-salicylhydroximato(1-)]hexaaluminiumytterbium–pyridine–water (1/7.386/1) (3) Special details

**Table:**

Geometry. All esds (except the esd in the dihedral angle between two l.s. planes) are estimated using the full covariance matrix. The cell esds are taken into account individually in the estimation of esds in distances, angles and torsion angles; correlations between esds in cell parameters are only used when they are defined by crystal symmetry. An approximate (isotropic) treatment of cell esds is used for estimating esds involving l.s. planes.
Refinement. The structure was solved by isomorphous replacement from its Dy and Gd analogues, RR1_61 and RR1_65. A section of the main molecule is disordered induced by presence or absence of an aluminum coordinated pyridine ligand. In the absence of the pyridine moiety a salicylate ligand swings into the space otherwise occupied by the pyridine, and the phenol oxygen atom coordinates to the aluminum (Al6). The movement of the salicylate ligand induces shifts to the Yb ion, to the aluminum ion the other two oxygen atoms are coordinated to (Al4) and one of the other salicylate ligands coordinated to Al4. The substantial movement of the first salicylate ligand induces a shift of the solvate pyridine its phenol oxygen is hydrogen bonded to. Another solvate pyridine molecule (adjacent to the second disordered salicylate ligand) was included in the disorder. Atoms C24B and C27B were constrained to have identical ADPs. The two Yb ions were constrained to have identical ADPs. Equivalent sections of the two disordered moieties were restrained to have similar geometries. The minor solvate pyridine ring was constrained to resemble an ideal hexagon with C-C distances of 1.39 Angstrom. The major moiety solvate pyridine ring was refined as additionally disordered. The nitrogen atoms of these two moieties were constrained to share position and ADP. Its two moieties and the other disordered pyridyl ring were restrained to have similar geometries as another not disordered pyridine ring. Uij components of ADPs for disordered atoms closer to each other than 2.0 Angstrom were restrained to be similar. Subject to these conditions the occupancy ratio refined to 0.8181 (14) to 0.1819 (14). The occupancy rates for the additionally split ring are 0.391 (3) and 0.324 (3).Two other solvate pyridyl rings are independently disordered with a shared occupancy ratio (N14, N17 vs N22, N24). The disordered moieties were 14 restrained to have similar geometries as another not disordered pyridine ring. Uij components of ADPs for disordered atoms closer to each other than 2.0 Angstrom were restrained to be similar. Subject to these conditions the occupancy ratio for the one disordered in a general position refined to 0.473 (7) to 0.527 (7). Another solvate pyridyl ring is 1:1 disordered around an inversion center and partially occupied. The disordered moieties were restrained to have similar geometries as another not disordered pyridine ring. Uij components of ADPs for disordered atoms closer to each other than 2.0 Angstrom were restrained to be similar. Subject to these conditions the occupancy rate refined to two times 0.386 (5).Water H atom positions and some amine H atom positions were refined and O-H and selected N-H distances were restrained to 0.84 (2) and 0.88 (2) Angstrom, respectively. Some water, amine and phenol H atom positions were further restrained based on hydrogen bonding considerations (phenol H atoms were placed in calculated positions, but were allowed to rotate around the C-O axis).

### Bis(pyridinium) diaquadipyridinehexakis[µ3-salicylhydroximato(3-)]bis[µ2-salicylhydroximato(1-)]hexaaluminiumytterbium–pyridine–water (1/7.386/1) (3) Fractional atomic coordinates and isotropic or equivalent isotropic displacement parameters (Å^2^)

**Table:**

	*x*	*y*	*z*	*U*_iso_*/*U*_eq_	Occ. (<1)
Al1	−0.04092 (7)	0.87325 (5)	0.12675 (3)	0.02231 (16)	
Al2	−0.32654 (7)	0.92911 (6)	0.23661 (3)	0.02613 (17)	
Al3	−0.13066 (7)	1.09896 (5)	0.25917 (3)	0.02484 (17)	
Al5	−0.15112 (9)	0.75674 (6)	0.36537 (3)	0.0355 (2)	
Al6	−0.05717 (7)	0.61729 (6)	0.23439 (3)	0.02670 (18)	
O1	−0.12608 (15)	0.91200 (12)	0.18188 (7)	0.0236 (4)	
O2	−0.16813 (16)	0.88859 (13)	0.10279 (7)	0.0263 (4)	
O3	−0.41286 (18)	0.89282 (16)	0.20612 (8)	0.0358 (5)	
O4	−0.22893 (16)	0.96625 (12)	0.26319 (7)	0.0242 (4)	
O5	−0.38462 (16)	1.05214 (14)	0.21910 (8)	0.0292 (4)	
O7	0.07372 (16)	0.84698 (13)	0.21019 (7)	0.0271 (4)	
O8	0.20070 (16)	0.92781 (15)	0.18155 (8)	0.0308 (4)	
O9	−0.03867 (15)	0.98390 (13)	0.09081 (7)	0.0245 (4)	
Yb1	−0.07121 (3)	0.84942 (2)	0.25794 (2)	0.02054 (6)	0.8181 (14)
Al4	0.16863 (13)	0.88039 (10)	0.24265 (7)	0.0214 (3)	0.8181 (14)
O10	0.1031 (2)	0.81729 (18)	0.29448 (12)	0.0250 (6)	0.8181 (14)
N4	0.1641 (3)	0.7317 (2)	0.30375 (11)	0.0297 (6)	0.8181 (14)
H4N	0.144779	0.692667	0.325220	0.036*	0.8181 (14)
O11	0.2726 (2)	0.77027 (17)	0.24635 (10)	0.0257 (5)	0.8181 (14)
C22	0.2529 (3)	0.7109 (2)	0.27896 (14)	0.0284 (7)	0.8181 (14)
C23	0.3317 (3)	0.6254 (2)	0.29054 (15)	0.0363 (8)	0.8181 (14)
C24	0.4242 (3)	0.6105 (3)	0.26345 (19)	0.0427 (10)	0.8181 (14)
H24	0.432345	0.653073	0.236909	0.051*	0.8181 (14)
C25	0.5056 (4)	0.5346 (3)	0.2742 (2)	0.0594 (13)	0.8181 (14)
H25	0.568649	0.524897	0.255279	0.071*	0.8181 (14)
C26	0.4923 (5)	0.4734 (3)	0.3134 (3)	0.0678 (15)	0.8181 (14)
H26	0.547208	0.421272	0.321175	0.081*	0.8181 (14)
C27	0.4027 (5)	0.4861 (3)	0.3409 (2)	0.0627 (14)	0.8181 (14)
H27	0.396019	0.443407	0.367683	0.075*	0.8181 (14)
C28	0.3196 (4)	0.5624 (3)	0.32993 (18)	0.0483 (11)	0.8181 (14)
O12	0.2277 (3)	0.5783 (2)	0.35537 (14)	0.0617 (10)	0.8181 (14)
H12O	0.230346	0.537828	0.378599	0.093*	0.8181 (14)
O25	−0.0424 (3)	0.9832 (3)	0.2648 (3)	0.0231 (10)	0.8181 (14)
N9	0.0605 (3)	0.9856 (2)	0.26072 (14)	0.0229 (8)	0.8181 (14)
O26	−0.0160 (4)	1.1305 (3)	0.2675 (2)	0.0288 (10)	0.8181 (14)
C57	0.0669 (3)	1.0638 (3)	0.26371 (14)	0.0234 (8)	0.8181 (14)
C58	0.1686 (3)	1.0780 (3)	0.26207 (15)	0.0275 (8)	0.8181 (14)
C59	0.1735 (3)	1.1640 (3)	0.25940 (16)	0.0346 (9)	0.8181 (14)
H59	0.111912	1.211824	0.258205	0.042*	0.8181 (14)
C60	0.2667 (4)	1.1807 (3)	0.25845 (19)	0.0440 (10)	0.8181 (14)
H60	0.269174	1.239661	0.256594	0.053*	0.8181 (14)
C61	0.3566 (4)	1.1106 (4)	0.26024 (19)	0.0450 (10)	0.8181 (14)
H61	0.420842	1.121814	0.259619	0.054*	0.8181 (14)
C62	0.3536 (3)	1.0251 (3)	0.26292 (16)	0.0352 (8)	0.8181 (14)
H62	0.415823	0.977951	0.263608	0.042*	0.8181 (14)
C63	0.2598 (3)	1.0067 (3)	0.26463 (14)	0.0273 (7)	0.8181 (14)
O27	0.2599 (2)	0.9221 (2)	0.26888 (10)	0.0262 (6)	0.8181 (14)
N11	0.0993 (3)	0.5503 (2)	0.24415 (13)	0.0293 (7)	0.8181 (14)
C69	0.1251 (3)	0.4813 (3)	0.27911 (16)	0.0394 (9)	0.8181 (14)
H69	0.073353	0.471252	0.302528	0.047*	0.8181 (14)
C70	0.2230 (4)	0.4246 (3)	0.28256 (19)	0.0470 (10)	0.8181 (14)
H70	0.238353	0.377316	0.308316	0.056*	0.8181 (14)
C71	0.2979 (4)	0.4367 (3)	0.2486 (2)	0.0482 (10)	0.8181 (14)
H71	0.365340	0.396862	0.249700	0.058*	0.8181 (14)
C72	0.2739 (4)	0.5076 (4)	0.21290 (18)	0.0502 (11)	0.8181 (14)
H72	0.324770	0.518037	0.189070	0.060*	0.8181 (14)
C73	0.1740 (3)	0.5639 (3)	0.21215 (17)	0.0417 (9)	0.8181 (14)
H73	0.158189	0.613897	0.187990	0.050*	0.8181 (14)
Yb1B	−0.09399 (13)	0.84739 (13)	0.26167 (7)	0.02054 (6)	0.1819 (14)
Al4B	0.1569 (6)	0.8569 (4)	0.2486 (3)	0.0223 (13)	0.1819 (14)
O10B	0.0848 (9)	0.7940 (7)	0.2981 (5)	0.026 (2)	0.1819 (14)
N4B	0.1209 (10)	0.7068 (8)	0.2912 (5)	0.030 (2)	0.1819 (14)
H4B	0.078 (6)	0.676 (8)	0.290 (6)	0.037*	0.1819 (14)
O11B	0.2491 (8)	0.7406 (7)	0.2467 (4)	0.0245 (19)	0.1819 (14)
C22B	0.2013 (11)	0.6836 (8)	0.2607 (6)	0.031 (2)	0.1819 (14)
C23B	0.2390 (11)	0.5924 (9)	0.2486 (7)	0.038 (2)	0.1819 (14)
C24B	0.3426 (11)	0.5614 (10)	0.2336 (7)	0.0391 (17)	0.1819 (14)
H24B	0.387124	0.595810	0.235214	0.047*	0.1819 (14)
C25B	0.3810 (14)	0.4804 (11)	0.2164 (8)	0.049 (3)	0.1819 (14)
H25B	0.449327	0.460398	0.203115	0.059*	0.1819 (14)
C26B	0.3141 (13)	0.4310 (13)	0.2198 (10)	0.051 (3)	0.1819 (14)
H26B	0.343384	0.370580	0.215761	0.062*	0.1819 (14)
C27B	0.2138 (12)	0.4584 (10)	0.2280 (7)	0.0391 (17)	0.1819 (14)
H27B	0.170635	0.426218	0.221171	0.047*	0.1819 (14)
C28B	0.1739 (11)	0.5393 (10)	0.2479 (7)	0.039 (2)	0.1819 (14)
O12B	0.0747 (10)	0.5672 (9)	0.2650 (6)	0.037 (2)	0.1819 (14)
H12P	0.069352	0.544469	0.293742	0.055*	0.1819 (14)
O25B	−0.0479 (14)	0.9712 (11)	0.2705 (15)	0.022 (3)	0.1819 (14)
N9B	0.0574 (11)	0.9643 (8)	0.2687 (8)	0.023 (2)	0.1819 (14)
O26B	−0.0057 (17)	1.1116 (11)	0.2747 (10)	0.022 (3)	0.1819 (14)
C57B	0.0714 (12)	1.0386 (10)	0.2726 (8)	0.025 (2)	0.1819 (14)
C58B	0.1771 (11)	1.0471 (10)	0.2699 (8)	0.026 (2)	0.1819 (14)
C59B	0.1890 (13)	1.1288 (12)	0.2694 (8)	0.035 (2)	0.1819 (14)
H59B	0.130254	1.179307	0.268888	0.042*	0.1819 (14)
C60B	0.2868 (13)	1.1387 (12)	0.2694 (8)	0.039 (2)	0.1819 (14)
H60B	0.295186	1.195468	0.268788	0.047*	0.1819 (14)
C61B	0.3706 (13)	1.0644 (13)	0.2705 (8)	0.039 (3)	0.1819 (14)
H61B	0.437637	1.069979	0.270184	0.047*	0.1819 (14)
C62B	0.3582 (12)	0.9827 (12)	0.2720 (7)	0.033 (2)	0.1819 (14)
H62B	0.417306	0.932475	0.273437	0.040*	0.1819 (14)
C63B	0.2622 (11)	0.9701 (11)	0.2714 (7)	0.026 (2)	0.1819 (14)
O27B	0.2554 (9)	0.8879 (8)	0.2754 (5)	0.025 (2)	0.1819 (14)
O13	−0.11370 (19)	0.86272 (14)	0.34489 (7)	0.0324 (5)	
O14	−0.0359 (2)	0.73062 (17)	0.40542 (9)	0.0464 (6)	
O15	0.0846 (4)	0.9367 (3)	0.40559 (15)	0.0932 (14)	
H15	0.098193	0.976183	0.417430	0.140*	
O16	−0.24018 (17)	0.81870 (14)	0.26900 (7)	0.0292 (4)	
O17	−0.40691 (17)	0.92421 (16)	0.29449 (8)	0.0332 (5)	
O18	−0.2411 (2)	0.78753 (17)	0.41566 (8)	0.0457 (6)	
O19	−0.06286 (19)	0.71750 (13)	0.31142 (7)	0.0321 (5)	
O20	−0.1570 (2)	0.64113 (16)	0.37475 (8)	0.0459 (7)	
O21	−0.0856 (2)	0.51349 (14)	0.25011 (8)	0.0355 (5)	
O22	−0.04363 (18)	0.73095 (13)	0.21439 (7)	0.0312 (5)	
O23	−0.02988 (18)	0.60566 (14)	0.17045 (8)	0.0319 (5)	
O24	0.03731 (16)	0.81787 (13)	0.07812 (7)	0.0261 (4)	
O28	−0.09387 (18)	1.10333 (14)	0.18847 (8)	0.0309 (4)	
H28A	−0.066 (3)	1.143 (2)	0.1766 (14)	0.046*	
H28B	−0.070 (3)	1.0558 (18)	0.1775 (15)	0.046*	
O29	−0.2072 (2)	0.67226 (17)	0.22686 (10)	0.0422 (6)	
H29A	−0.226 (4)	0.7271 (18)	0.2360 (17)	0.063*	
H29B	−0.252 (3)	0.666 (3)	0.2057 (14)	0.063*	
O30	0.4147 (6)	0.4636 (6)	0.0132 (3)	0.172 (3)	
H30A	0.437 (3)	0.486 (7)	0.034 (4)	0.257*	
H30B	0.398 (9)	0.416 (5)	0.024 (5)	0.257*	
N1	−0.22875 (19)	0.91198 (16)	0.17895 (8)	0.0250 (5)	
N2	−0.24119 (18)	1.05637 (15)	0.24970 (9)	0.0241 (5)	
N3	0.06560 (19)	0.88916 (15)	0.16264 (8)	0.0242 (5)	
N5	−0.0382 (2)	0.86538 (19)	0.37205 (10)	0.0363 (6)	
H5N	−0.017819	0.912849	0.369661	0.044*	
N6	−0.2648 (2)	0.81431 (17)	0.31999 (9)	0.0308 (5)	
N7	−0.0814 (2)	0.64186 (16)	0.29948 (9)	0.0337 (6)	
N8	−0.02648 (19)	0.74791 (15)	0.16365 (8)	0.0254 (5)	
C1	−0.2429 (2)	0.90087 (18)	0.13547 (10)	0.0252 (5)	
C2	−0.3454 (2)	0.9027 (2)	0.12375 (11)	0.0288 (6)	
C3	−0.3630 (3)	0.9075 (2)	0.07532 (12)	0.0348 (7)	
H3	−0.310739	0.913993	0.051318	0.042*	
C4	−0.4551 (3)	0.9032 (3)	0.06174 (13)	0.0422 (8)	
H4	−0.466506	0.906923	0.028783	0.051*	
C5	−0.5302 (3)	0.8931 (3)	0.09724 (14)	0.0438 (8)	
H5	−0.592682	0.887913	0.088436	0.053*	
C6	−0.5160 (3)	0.8906 (3)	0.14507 (13)	0.0416 (8)	
H6	−0.569448	0.885013	0.168555	0.050*	
C7	−0.4235 (3)	0.8961 (2)	0.15973 (11)	0.0328 (6)	
C8	−0.3257 (2)	1.09669 (19)	0.22724 (11)	0.0263 (5)	0.8181 (14)
C9	−0.3543 (4)	1.1932 (3)	0.2134 (2)	0.0289 (12)	0.8181 (14)
C10	−0.4438 (4)	1.2331 (4)	0.18837 (18)	0.0352 (12)	0.8181 (14)
H10	−0.481954	1.197455	0.180558	0.042*	0.8181 (14)
C11	−0.4771 (4)	1.3240 (3)	0.17490 (19)	0.0452 (12)	0.8181 (14)
H11	−0.537364	1.350572	0.157750	0.054*	0.8181 (14)
C12	−0.4213 (4)	1.3758 (3)	0.18675 (19)	0.0448 (10)	0.8181 (14)
H12	−0.443666	1.438044	0.177710	0.054*	0.8181 (14)
C13	−0.3330 (4)	1.3371 (3)	0.21172 (17)	0.0381 (10)	0.8181 (14)
H13	−0.296271	1.373874	0.219604	0.046*	0.8181 (14)
C14	−0.2961 (3)	1.2453 (3)	0.22571 (16)	0.0296 (11)	0.8181 (14)
O6	−0.21040 (17)	1.21288 (13)	0.24918 (8)	0.0318 (5)	0.8181 (14)
C8B	−0.3257 (2)	1.09669 (19)	0.22724 (11)	0.0263 (5)	0.1819 (14)
C9B	−0.341 (2)	1.1912 (11)	0.2066 (12)	0.031 (3)	0.1819 (14)
C10B	−0.4243 (18)	1.2351 (14)	0.1780 (10)	0.034 (3)	0.1819 (14)
H10B	−0.467010	1.202757	0.170775	0.041*	0.1819 (14)
C11B	−0.4465 (17)	1.3252 (13)	0.1597 (9)	0.043 (3)	0.1819 (14)
H11B	−0.505216	1.353424	0.141598	0.052*	0.1819 (14)
C12B	−0.3828 (16)	1.3731 (12)	0.1679 (8)	0.044 (3)	0.1819 (14)
H12B	−0.394887	1.433020	0.153698	0.053*	0.1819 (14)
C13B	−0.3008 (16)	1.3325 (12)	0.1972 (8)	0.039 (3)	0.1819 (14)
H13B	−0.258922	1.365838	0.204102	0.046*	0.1819 (14)
C14B	−0.2797 (18)	1.2429 (12)	0.2166 (9)	0.033 (3)	0.1819 (14)
O6B	−0.21040 (17)	1.21288 (13)	0.24918 (8)	0.0318 (5)	0.1819 (14)
C15	0.1337 (2)	0.93221 (19)	0.15125 (10)	0.0263 (6)	
C16	0.1310 (2)	0.98785 (19)	0.10420 (10)	0.0266 (6)	
C17	0.2117 (2)	1.0233 (2)	0.08908 (11)	0.0330 (6)	
H17	0.267322	1.010252	0.109111	0.040*	
C18	0.2126 (3)	1.0773 (2)	0.04544 (12)	0.0358 (7)	
H18	0.268098	1.101203	0.035662	0.043*	
C19	0.1312 (3)	1.0960 (2)	0.01625 (11)	0.0324 (6)	
H19	0.131810	1.131485	−0.014134	0.039*	
C20	0.0487 (2)	1.06301 (19)	0.03128 (10)	0.0262 (5)	
H20	−0.006647	1.076851	0.011002	0.031*	
C21	0.0454 (2)	1.00953 (18)	0.07588 (10)	0.0234 (5)	
C29	0.0024 (3)	0.7940 (2)	0.40177 (12)	0.0421 (8)	
C30	0.0921 (3)	0.7866 (3)	0.42913 (14)	0.0516 (10)	
C31	0.1403 (4)	0.7050 (4)	0.45400 (18)	0.0702 (15)	
H31	0.114699	0.656511	0.453624	0.084*	
C32	0.2265 (5)	0.6931 (4)	0.4798 (2)	0.0844 (18)	
H32	0.258428	0.637119	0.497289	0.101*	
C33	0.2637 (5)	0.7613 (5)	0.4796 (2)	0.0854 (19)	
H33	0.322567	0.752876	0.496847	0.103*	
C34	0.2188 (4)	0.8420 (5)	0.45534 (19)	0.0786 (16)	
H34	0.246539	0.889247	0.455827	0.094*	
C35	0.1318 (4)	0.8569 (4)	0.42945 (16)	0.0632 (12)	
C36	−0.3535 (3)	0.8724 (2)	0.32876 (11)	0.0337 (7)	
C37	−0.3963 (3)	0.8808 (2)	0.37885 (12)	0.0397 (8)	
C38	−0.4968 (3)	0.9354 (3)	0.38552 (15)	0.0525 (10)	
H38	−0.534234	0.965579	0.358234	0.063*	
C39	−0.5429 (4)	0.9463 (4)	0.43125 (17)	0.0671 (13)	
H39	−0.611175	0.983412	0.435440	0.081*	
C40	−0.4869 (5)	0.9019 (4)	0.47063 (16)	0.0713 (14)	
H40	−0.517528	0.908613	0.502126	0.086*	
C41	−0.3879 (4)	0.8484 (3)	0.46485 (15)	0.0638 (13)	
H41	−0.351919	0.818555	0.492588	0.077*	
C42	−0.3379 (4)	0.8365 (2)	0.41890 (12)	0.0460 (9)	
C43	−0.1290 (3)	0.6050 (2)	0.33577 (11)	0.0375 (7)	
C44	−0.1534 (3)	0.5228 (2)	0.33233 (12)	0.0391 (8)	
C45	−0.1984 (4)	0.4834 (2)	0.37322 (14)	0.0526 (11)	
H45	−0.211957	0.510087	0.401799	0.063*	
C46	−0.2234 (4)	0.4065 (3)	0.37288 (16)	0.0626 (13)	
H46	−0.252829	0.380017	0.401078	0.075*	
C47	−0.2051 (4)	0.3681 (3)	0.33067 (16)	0.0545 (11)	
H47	−0.223613	0.316061	0.329840	0.065*	
C48	−0.1602 (3)	0.4055 (2)	0.29015 (14)	0.0440 (9)	
H48	−0.147916	0.378422	0.261735	0.053*	
C49	−0.1320 (3)	0.4832 (2)	0.28986 (12)	0.0361 (7)	
C50	−0.0190 (2)	0.67730 (18)	0.14440 (10)	0.0249 (5)	
C51	0.0097 (2)	0.6754 (2)	0.09269 (11)	0.0298 (6)	
C52	0.0129 (4)	0.5992 (3)	0.07354 (14)	0.0496 (10)	
H52	−0.013309	0.555603	0.092746	0.059*	
C53	0.0528 (4)	0.5863 (3)	0.02778 (15)	0.0588 (12)	
H53	0.054967	0.534064	0.015495	0.071*	
C54	0.0901 (3)	0.6505 (2)	−0.00028 (13)	0.0442 (8)	
H54	0.120690	0.641078	−0.031522	0.053*	
C55	0.0832 (3)	0.7275 (2)	0.01675 (11)	0.0325 (6)	
H55	0.106990	0.771627	−0.003434	0.039*	
C56	0.0416 (2)	0.74267 (18)	0.06356 (10)	0.0257 (5)	
N10	−0.1705 (2)	1.08997 (17)	0.33428 (10)	0.0338 (6)	
C64	−0.1319 (3)	1.1308 (3)	0.36193 (14)	0.0488 (9)	
H64	−0.084255	1.161170	0.347704	0.059*	
C65	−0.1593 (5)	1.1303 (4)	0.41073 (16)	0.0694 (14)	
H65	−0.130646	1.159692	0.429533	0.083*	
C66	−0.2291 (5)	1.0862 (3)	0.43167 (15)	0.0711 (15)	
H66	−0.249157	1.085636	0.464944	0.085*	
C67	−0.2684 (4)	1.0439 (3)	0.40414 (14)	0.0619 (13)	
H67	−0.315974	1.013049	0.417704	0.074*	
C68	−0.2370 (3)	1.0472 (2)	0.35556 (13)	0.0438 (8)	
H68	−0.264199	1.017343	0.336352	0.053*	
N12	−0.2038 (2)	1.1054 (2)	0.06042 (12)	0.0461 (8)	
H12A	−0.157464	1.055084	0.069616	0.055*	
C74	−0.2342 (3)	1.1660 (3)	0.08932 (15)	0.0562 (11)	
H74	−0.207476	1.153952	0.120201	0.067*	
C75	−0.3028 (4)	1.2454 (3)	0.07591 (18)	0.0614 (12)	
H75	−0.323501	1.288926	0.096997	0.074*	
C76	−0.3422 (4)	1.2623 (3)	0.03144 (18)	0.0625 (12)	
H76	−0.389003	1.318103	0.020962	0.075*	
C77	−0.3126 (3)	1.1967 (4)	0.00234 (16)	0.0625 (12)	
H77	−0.341206	1.205954	−0.027938	0.075*	
C78	−0.2420 (3)	1.1186 (3)	0.01731 (17)	0.0558 (10)	
H78	−0.219981	1.073615	−0.002866	0.067*	
N13	−0.0147 (3)	1.23113 (19)	0.15011 (11)	0.0417 (7)	
C79	−0.0253 (3)	1.2908 (2)	0.17981 (15)	0.0487 (9)	
H79	−0.061736	1.283754	0.210035	0.058*	
C80	0.0130 (3)	1.3610 (2)	0.16919 (16)	0.0505 (10)	
H80	0.003989	1.401684	0.191332	0.061*	
C81	0.0649 (4)	1.3706 (3)	0.1255 (2)	0.0656 (13)	
H81	0.093021	1.418578	0.116797	0.079*	
C82	0.0763 (4)	1.3099 (4)	0.09403 (18)	0.0713 (14)	
H82	0.111735	1.315817	0.063452	0.086*	
C83	0.0355 (3)	1.2413 (3)	0.10792 (14)	0.0508 (9)	
H83	0.043514	1.199522	0.086510	0.061*	
N14	0.132 (2)	1.0829 (12)	0.4242 (6)	0.089 (2)	0.473 (7)
C84	0.127 (3)	1.1113 (13)	0.4669 (6)	0.092 (3)	0.473 (7)
H84	0.132636	1.070329	0.495571	0.110*	0.473 (7)
C85	0.1151 (14)	1.1971 (10)	0.4695 (5)	0.097 (3)	0.473 (7)
H85	0.118193	1.214781	0.499670	0.117*	0.473 (7)
C86	0.0985 (15)	1.2584 (10)	0.4297 (5)	0.111 (3)	0.473 (7)
H86	0.087778	1.318799	0.432319	0.134*	0.473 (7)
C87	0.0970 (15)	1.2333 (10)	0.3848 (5)	0.109 (3)	0.473 (7)
H87	0.082130	1.274900	0.356545	0.131*	0.473 (7)
C88	0.1185 (15)	1.1445 (10)	0.3847 (5)	0.102 (3)	0.473 (7)
H88	0.124603	1.124397	0.354429	0.123*	0.473 (7)
N22	0.128 (2)	1.0652 (13)	0.4296 (6)	0.095 (3)	0.527 (7)
C124	0.122 (2)	1.0946 (14)	0.4724 (6)	0.096 (3)	0.527 (7)
H124	0.088957	1.068030	0.499152	0.116*	0.527 (7)
C125	0.1610 (13)	1.1592 (10)	0.4798 (5)	0.098 (3)	0.527 (7)
H125	0.161473	1.173005	0.511193	0.118*	0.527 (7)
C126	0.1995 (13)	1.2039 (10)	0.4402 (4)	0.111 (3)	0.527 (7)
H126	0.229974	1.247967	0.443921	0.134*	0.527 (7)
C127	0.1937 (14)	1.1842 (10)	0.3946 (4)	0.112 (3)	0.527 (7)
H127	0.202694	1.223012	0.366396	0.135*	0.527 (7)
C128	0.1744 (14)	1.1071 (10)	0.3924 (5)	0.105 (3)	0.527 (7)
H128	0.194255	1.081149	0.363580	0.126*	0.527 (7)
N15	0.1794 (5)	0.4684 (4)	0.42411 (18)	0.0753 (13)	0.427 (3)
C89	0.2285 (14)	0.4309 (15)	0.4625 (6)	0.077 (3)	0.427 (3)
H89	0.295630	0.436279	0.462182	0.093*	0.427 (3)
C90	0.1975 (13)	0.3853 (18)	0.5030 (7)	0.087 (3)	0.427 (3)
H90	0.238563	0.360206	0.529804	0.105*	0.427 (3)
C91	0.0978 (12)	0.3792 (11)	0.5011 (5)	0.096 (3)	0.427 (3)
H91	0.067992	0.350129	0.528141	0.115*	0.427 (3)
C92	0.0407 (12)	0.4158 (11)	0.4594 (5)	0.100 (3)	0.427 (3)
H92	−0.024577	0.407960	0.457649	0.120*	0.427 (3)
C93	0.0818 (10)	0.4623 (11)	0.4221 (5)	0.096 (3)	0.427 (3)
H93	0.043383	0.490436	0.394797	0.115*	0.427 (3)
N23	0.1794 (5)	0.4684 (4)	0.42411 (18)	0.0753 (13)	0.391 (3)
C129	0.2246 (14)	0.4188 (16)	0.4624 (6)	0.075 (3)	0.391 (3)
H129	0.297410	0.397381	0.459289	0.090*	0.391 (3)
C130	0.1804 (13)	0.3945 (19)	0.5057 (7)	0.081 (3)	0.391 (3)
H130	0.219317	0.355663	0.530691	0.097*	0.391 (3)
C131	0.0742 (12)	0.4302 (11)	0.5111 (5)	0.091 (3)	0.391 (3)
H131	0.037441	0.417863	0.540531	0.109*	0.391 (3)
C132	0.0233 (12)	0.4849 (12)	0.4719 (5)	0.097 (3)	0.391 (3)
H132	−0.049265	0.508792	0.474274	0.116*	0.391 (3)
C133	0.0770 (9)	0.5040 (11)	0.4303 (5)	0.086 (3)	0.391 (3)
H133	0.041126	0.543863	0.404776	0.103*	0.391 (3)
N25	0.076 (2)	0.4201 (16)	0.3605 (6)	0.112 (4)	0.1819 (14)
C139	0.1416 (19)	0.4411 (15)	0.3874 (8)	0.095 (3)	0.1819 (14)
H139	0.183287	0.476648	0.372424	0.113*	0.1819 (14)
C140	0.1467 (19)	0.4099 (16)	0.4363 (8)	0.091 (3)	0.1819 (14)
H140	0.191789	0.424261	0.454669	0.109*	0.1819 (14)
C141	0.086 (2)	0.3579 (17)	0.4582 (6)	0.102 (4)	0.1819 (14)
H141	0.089229	0.336590	0.491616	0.122*	0.1819 (14)
C142	0.020 (2)	0.3369 (17)	0.4313 (9)	0.107 (5)	0.1819 (14)
H142	−0.021832	0.301305	0.446318	0.128*	0.1819 (14)
C143	0.0148 (19)	0.3680 (17)	0.3825 (9)	0.112 (6)	0.1819 (14)
H143	−0.030336	0.353691	0.364073	0.134*	0.1819 (14)
N16	0.4145 (3)	0.8007 (2)	0.31105 (12)	0.0499 (8)	
H16	0.368597	0.843712	0.294832	0.060*	
C94	0.5087 (3)	0.7697 (3)	0.29014 (15)	0.0501 (9)	
H94	0.524894	0.794679	0.258529	0.060*	
C95	0.5810 (4)	0.7025 (3)	0.31416 (18)	0.0589 (11)	
H95	0.647360	0.679184	0.299435	0.071*	
C96	0.5555 (4)	0.6689 (4)	0.3604 (2)	0.0781 (16)	
H96	0.605469	0.622917	0.378015	0.094*	
C97	0.4585 (5)	0.7014 (4)	0.3814 (2)	0.093 (2)	
H97	0.440696	0.677878	0.413077	0.112*	
C98	0.3881 (4)	0.7687 (3)	0.35534 (17)	0.0653 (13)	
H98	0.320775	0.792387	0.368987	0.078*	
N17	0.4593 (12)	1.2702 (13)	0.4156 (7)	0.091 (3)	0.473 (7)
C99	0.5467 (16)	1.2688 (19)	0.3902 (8)	0.084 (3)	0.473 (7)
H99	0.592545	1.294025	0.401373	0.101*	0.473 (7)
C100	0.5731 (18)	1.2321 (16)	0.3484 (6)	0.086 (3)	0.473 (7)
H100	0.633760	1.235309	0.329420	0.103*	0.473 (7)
C101	0.5101 (16)	1.1914 (14)	0.3350 (6)	0.090 (3)	0.473 (7)
H101	0.532677	1.153412	0.310970	0.108*	0.473 (7)
C102	0.4104 (13)	1.2057 (12)	0.3571 (6)	0.100 (3)	0.473 (7)
H102	0.358740	1.191919	0.342788	0.119*	0.473 (7)
C103	0.3897 (13)	1.2399 (12)	0.3996 (6)	0.095 (3)	0.473 (7)
H103	0.326564	1.242194	0.417635	0.114*	0.473 (7)
N24	0.4949 (11)	1.2571 (12)	0.4167 (6)	0.095 (3)	0.527 (7)
C134	0.5681 (15)	1.2689 (17)	0.3847 (7)	0.084 (3)	0.527 (7)
H134	0.617331	1.293631	0.393238	0.100*	0.527 (7)
C135	0.5752 (17)	1.2468 (15)	0.3398 (6)	0.089 (3)	0.527 (7)
H135	0.628578	1.256270	0.317266	0.107*	0.527 (7)
C136	0.5066 (16)	1.2114 (12)	0.3274 (5)	0.089 (3)	0.527 (7)
H136	0.500745	1.208295	0.294612	0.106*	0.527 (7)
C137	0.4433 (12)	1.1789 (11)	0.3636 (5)	0.101 (3)	0.527 (7)
H137	0.411838	1.135855	0.359158	0.121*	0.527 (7)
C138	0.4305 (12)	1.2141 (11)	0.4057 (5)	0.096 (3)	0.527 (7)
H138	0.375095	1.208335	0.427886	0.116*	0.527 (7)
N18	−0.2585 (3)	0.6305 (3)	0.14619 (15)	0.0630 (10)	
C104	−0.2298 (4)	0.5410 (3)	0.1488 (2)	0.0679 (13)	
H104	−0.208761	0.507195	0.178776	0.082*	
C105	−0.2287 (5)	0.4968 (4)	0.1124 (2)	0.0747 (14)	
H105	−0.210670	0.434103	0.117284	0.090*	
C106	−0.2536 (4)	0.5428 (4)	0.0685 (2)	0.0747 (15)	
H106	−0.250366	0.512842	0.041815	0.090*	
C107	−0.2847 (5)	0.6369 (4)	0.0631 (2)	0.0812 (16)	
H107	−0.303966	0.673058	0.033450	0.097*	
C108	−0.2842 (5)	0.6700 (5)	0.1039 (2)	0.100 (2)	
H108	−0.306522	0.732685	0.100941	0.120*	
N19	0.3769 (4)	0.5758 (3)	0.08478 (18)	0.0754 (12)	
C109	0.4537 (4)	0.5945 (3)	0.09772 (19)	0.0652 (12)	
H109	0.519910	0.558464	0.088423	0.078*	
C110	0.4508 (5)	0.6611 (5)	0.1237 (2)	0.0857 (19)	
H110	0.510626	0.670014	0.133281	0.103*	
C111	0.3447 (4)	0.7179 (3)	0.13515 (16)	0.0598 (11)	
H111	0.332169	0.766827	0.152180	0.072*	
C112	0.2683 (3)	0.6954 (3)	0.11994 (17)	0.0550 (10)	
H112	0.199235	0.729453	0.125971	0.066*	
C113	0.2882 (4)	0.6251 (3)	0.0962 (2)	0.0698 (13)	
H113	0.230937	0.611234	0.087139	0.084*	
N20	0.3916 (5)	0.2872 (5)	0.0601 (3)	0.1070 (19)	
C114	0.4394 (6)	0.2036 (7)	0.0553 (3)	0.111 (3)	
H114	0.479852	0.190464	0.026477	0.133*	
C115	0.4327 (6)	0.1330 (6)	0.0914 (4)	0.118 (3)	
H115	0.469829	0.073649	0.087640	0.142*	
C116	0.3714 (6)	0.1526 (6)	0.1317 (3)	0.100 (2)	
H116	0.363955	0.107325	0.156787	0.120*	
C117	0.3212 (7)	0.2390 (7)	0.1350 (3)	0.118 (3)	
H117	0.277176	0.254179	0.162553	0.141*	
C118	0.3327 (5)	0.3035 (6)	0.0997 (3)	0.102 (2)	
H118	0.296957	0.363129	0.103446	0.122*	
N21	0.4232 (16)	0.4639 (13)	0.5240 (8)	0.084 (4)	0.386 (5)
C119	0.4662 (18)	0.4480 (16)	0.4806 (9)	0.077 (5)	0.386 (5)
H119	0.450003	0.407394	0.464121	0.093*	0.386 (5)
C120	0.533 (3)	0.491 (2)	0.4610 (11)	0.085 (5)	0.386 (5)
H120	0.572357	0.474661	0.432323	0.102*	0.386 (5)
C121	0.5484 (18)	0.5602 (16)	0.4813 (9)	0.084 (5)	0.386 (5)
H121	0.586848	0.597364	0.464736	0.101*	0.386 (5)
C122	0.5043 (16)	0.5702 (15)	0.5259 (9)	0.072 (5)	0.386 (5)
H122	0.510995	0.615470	0.542031	0.087*	0.386 (5)
C123	0.453 (3)	0.517 (2)	0.5465 (9)	0.075 (5)	0.386 (5)
H123	0.434815	0.516190	0.580028	0.090*	0.386 (5)

### Bis(pyridinium) diaquadipyridinehexakis[µ3-salicylhydroximato(3-)]bis[µ2-salicylhydroximato(1-)]hexaaluminiumytterbium–pyridine–water (1/7.386/1) (3) Atomic displacement parameters (Å^2^)

**Table:**

	*U*^11^	*U*^22^	*U*^33^	*U*^12^	*U*^13^	*U*^23^
Al1	0.0268 (4)	0.0217 (4)	0.0182 (4)	−0.0045 (3)	−0.0042 (3)	−0.0056 (3)
Al2	0.0284 (4)	0.0318 (4)	0.0220 (4)	−0.0132 (4)	−0.0011 (3)	−0.0064 (3)
Al3	0.0268 (4)	0.0234 (4)	0.0262 (4)	−0.0068 (3)	−0.0039 (3)	−0.0086 (3)
Al5	0.0602 (7)	0.0296 (4)	0.0183 (4)	−0.0149 (4)	−0.0033 (4)	−0.0039 (4)
Al6	0.0328 (5)	0.0250 (4)	0.0268 (4)	−0.0148 (3)	0.0055 (4)	−0.0088 (3)
O1	0.0252 (9)	0.0258 (9)	0.0202 (9)	−0.0063 (7)	−0.0039 (8)	−0.0054 (7)
O2	0.0288 (10)	0.0317 (10)	0.0188 (9)	−0.0074 (8)	−0.0037 (8)	−0.0060 (8)
O3	0.0381 (12)	0.0528 (14)	0.0266 (11)	−0.0268 (11)	−0.0002 (9)	−0.0091 (10)
O4	0.0309 (10)	0.0225 (9)	0.0213 (9)	−0.0096 (8)	−0.0012 (8)	−0.0061 (7)
O5	0.0234 (10)	0.0338 (10)	0.0320 (11)	−0.0085 (8)	−0.0029 (8)	−0.0079 (9)
O7	0.0351 (11)	0.0251 (9)	0.0188 (9)	−0.0035 (8)	−0.0077 (8)	−0.0032 (8)
O8	0.0266 (10)	0.0408 (11)	0.0262 (10)	−0.0077 (9)	−0.0098 (8)	−0.0068 (9)
O9	0.0246 (9)	0.0246 (9)	0.0241 (9)	−0.0058 (7)	−0.0051 (8)	−0.0039 (8)
Yb1	0.02534 (15)	0.02103 (6)	0.01699 (9)	−0.00851 (10)	−0.00090 (10)	−0.00466 (5)
Al4	0.0240 (6)	0.0218 (7)	0.0204 (6)	−0.0087 (5)	−0.0057 (5)	−0.0021 (5)
O10	0.0309 (14)	0.0218 (13)	0.0211 (12)	−0.0062 (10)	−0.0029 (11)	−0.0020 (11)
N4	0.0388 (16)	0.0251 (13)	0.0240 (14)	−0.0092 (12)	−0.0044 (12)	0.0011 (11)
O11	0.0281 (13)	0.0229 (12)	0.0278 (12)	−0.0102 (10)	−0.0044 (10)	−0.0014 (10)
C22	0.0326 (17)	0.0239 (14)	0.0291 (17)	−0.0070 (13)	−0.0081 (14)	−0.0032 (13)
C23	0.042 (2)	0.0250 (16)	0.040 (2)	−0.0075 (15)	−0.0102 (17)	−0.0003 (15)
C24	0.035 (2)	0.0293 (18)	0.062 (3)	−0.0076 (15)	−0.0101 (19)	−0.0025 (18)
C25	0.045 (3)	0.032 (2)	0.095 (4)	−0.0006 (19)	−0.015 (3)	−0.006 (2)
C26	0.063 (3)	0.037 (2)	0.095 (4)	−0.001 (2)	−0.029 (3)	0.005 (3)
C27	0.077 (3)	0.034 (2)	0.068 (3)	−0.005 (2)	−0.027 (3)	0.013 (2)
C28	0.062 (3)	0.035 (2)	0.044 (2)	−0.0112 (19)	−0.017 (2)	0.0090 (18)
O12	0.080 (3)	0.0403 (18)	0.049 (2)	−0.0094 (17)	0.0045 (19)	0.0170 (15)
O25	0.0228 (14)	0.0226 (15)	0.026 (3)	−0.0074 (11)	−0.0035 (13)	−0.0073 (17)
N9	0.0229 (13)	0.0231 (15)	0.0254 (19)	−0.0085 (12)	−0.0062 (12)	−0.0045 (14)
O26	0.0330 (18)	0.0217 (17)	0.033 (3)	−0.0064 (14)	−0.0083 (15)	−0.0074 (17)
C57	0.0289 (16)	0.0230 (17)	0.0204 (18)	−0.0090 (14)	−0.0058 (13)	−0.0031 (14)
C58	0.0312 (17)	0.0265 (17)	0.0292 (18)	−0.0137 (15)	−0.0076 (14)	−0.0025 (15)
C59	0.041 (2)	0.0268 (18)	0.041 (2)	−0.0150 (16)	−0.0064 (17)	−0.0066 (16)
C60	0.049 (2)	0.037 (2)	0.055 (3)	−0.0256 (19)	−0.004 (2)	−0.0062 (19)
C61	0.043 (2)	0.044 (2)	0.059 (3)	−0.028 (2)	−0.006 (2)	−0.009 (2)
C62	0.0325 (18)	0.037 (2)	0.041 (2)	−0.0164 (17)	−0.0043 (16)	−0.0075 (18)
C63	0.0328 (17)	0.0292 (17)	0.0271 (17)	−0.0169 (15)	−0.0073 (14)	−0.0053 (14)
O27	0.0301 (13)	0.0269 (14)	0.0255 (13)	−0.0131 (12)	−0.0081 (10)	−0.0018 (12)
N11	0.0331 (17)	0.0262 (14)	0.0299 (17)	−0.0117 (13)	0.0003 (14)	−0.0036 (13)
C69	0.0373 (19)	0.0382 (19)	0.040 (2)	−0.0116 (16)	0.0002 (17)	0.0013 (16)
C70	0.043 (2)	0.040 (2)	0.052 (2)	−0.0062 (17)	−0.0052 (19)	0.0015 (19)
C71	0.038 (2)	0.050 (2)	0.049 (2)	−0.0011 (18)	−0.0012 (19)	−0.007 (2)
C72	0.0339 (19)	0.062 (2)	0.046 (2)	−0.0083 (18)	0.0038 (18)	0.000 (2)
C73	0.0359 (19)	0.045 (2)	0.041 (2)	−0.0132 (16)	0.0003 (17)	0.0028 (17)
Yb1B	0.02534 (15)	0.02103 (6)	0.01699 (9)	−0.00851 (10)	−0.00090 (10)	−0.00466 (5)
Al4B	0.024 (2)	0.024 (3)	0.022 (3)	−0.006 (2)	−0.006 (2)	−0.008 (2)
O10B	0.033 (4)	0.024 (4)	0.020 (4)	−0.006 (4)	−0.002 (4)	−0.007 (4)
N4B	0.038 (4)	0.025 (4)	0.027 (4)	−0.006 (4)	−0.006 (4)	−0.002 (4)
O11B	0.023 (4)	0.023 (4)	0.026 (4)	−0.008 (3)	−0.002 (3)	0.001 (3)
C22B	0.036 (4)	0.024 (3)	0.033 (4)	−0.010 (3)	−0.010 (3)	0.000 (3)
C23B	0.032 (4)	0.039 (4)	0.042 (4)	−0.013 (4)	0.003 (4)	0.003 (4)
C24B	0.038 (3)	0.032 (3)	0.042 (3)	−0.012 (3)	0.006 (3)	0.009 (3)
C25B	0.042 (5)	0.046 (5)	0.051 (5)	−0.007 (5)	0.006 (5)	0.000 (5)
C26B	0.045 (5)	0.048 (5)	0.054 (5)	−0.011 (5)	0.005 (5)	−0.001 (5)
C27B	0.038 (3)	0.032 (3)	0.042 (3)	−0.012 (3)	0.006 (3)	0.009 (3)
C28B	0.035 (3)	0.040 (3)	0.042 (3)	−0.013 (3)	0.002 (3)	0.001 (3)
O12B	0.034 (4)	0.034 (4)	0.041 (5)	−0.011 (4)	−0.001 (4)	0.001 (4)
O25B	0.019 (5)	0.023 (5)	0.023 (5)	−0.005 (4)	−0.005 (4)	0.000 (5)
N9B	0.025 (4)	0.022 (4)	0.021 (4)	−0.007 (3)	−0.004 (3)	−0.004 (4)
O26B	0.025 (5)	0.022 (5)	0.021 (5)	−0.005 (4)	−0.013 (4)	−0.003 (5)
C57B	0.025 (4)	0.024 (4)	0.025 (4)	−0.006 (4)	−0.004 (3)	−0.006 (4)
C58B	0.033 (3)	0.024 (4)	0.027 (4)	−0.015 (3)	−0.005 (3)	−0.006 (3)
C59B	0.038 (4)	0.032 (4)	0.039 (4)	−0.016 (4)	−0.008 (4)	−0.001 (4)
C60B	0.040 (4)	0.035 (4)	0.047 (4)	−0.019 (4)	−0.008 (4)	−0.004 (4)
C61B	0.039 (4)	0.035 (4)	0.047 (4)	−0.017 (4)	−0.006 (4)	−0.004 (4)
C62B	0.034 (4)	0.035 (4)	0.034 (4)	−0.013 (4)	−0.004 (4)	−0.005 (4)
C63B	0.029 (4)	0.029 (4)	0.027 (4)	−0.016 (4)	−0.008 (3)	−0.005 (4)
O27B	0.027 (4)	0.029 (4)	0.024 (4)	−0.010 (4)	−0.009 (3)	−0.007 (4)
O13	0.0477 (13)	0.0311 (10)	0.0209 (10)	−0.0113 (9)	−0.0086 (9)	−0.0067 (8)
O14	0.0717 (18)	0.0388 (13)	0.0263 (12)	−0.0087 (12)	−0.0139 (12)	−0.0057 (10)
O15	0.126 (4)	0.101 (3)	0.079 (3)	−0.061 (3)	−0.058 (3)	0.002 (2)
O16	0.0409 (12)	0.0317 (10)	0.0179 (9)	−0.0147 (9)	0.0003 (8)	−0.0051 (8)
O17	0.0344 (12)	0.0457 (12)	0.0254 (10)	−0.0194 (10)	0.0024 (9)	−0.0094 (9)
O18	0.0690 (18)	0.0431 (13)	0.0205 (11)	−0.0101 (13)	0.0004 (11)	−0.0065 (10)
O19	0.0527 (14)	0.0242 (9)	0.0221 (10)	−0.0133 (9)	−0.0046 (9)	−0.0056 (8)
O20	0.087 (2)	0.0318 (11)	0.0204 (11)	−0.0236 (13)	0.0054 (12)	−0.0029 (9)
O21	0.0561 (15)	0.0303 (10)	0.0283 (11)	−0.0262 (10)	0.0075 (10)	−0.0072 (9)
O22	0.0514 (13)	0.0233 (9)	0.0175 (9)	−0.0080 (9)	−0.0029 (9)	−0.0042 (8)
O23	0.0444 (13)	0.0274 (10)	0.0309 (11)	−0.0202 (9)	0.0079 (10)	−0.0119 (9)
O24	0.0340 (11)	0.0225 (9)	0.0219 (9)	−0.0062 (8)	−0.0018 (8)	−0.0073 (8)
O28	0.0388 (12)	0.0291 (10)	0.0287 (11)	−0.0139 (9)	0.0003 (9)	−0.0089 (9)
O29	0.0443 (14)	0.0428 (13)	0.0453 (15)	−0.0186 (11)	−0.0016 (12)	−0.0116 (12)
O30	0.173 (6)	0.224 (8)	0.160 (7)	−0.078 (6)	0.016 (5)	−0.124 (6)
N1	0.0268 (12)	0.0288 (11)	0.0221 (11)	−0.0111 (9)	−0.0037 (9)	−0.0046 (9)
N2	0.0265 (11)	0.0216 (10)	0.0243 (11)	−0.0058 (9)	−0.0008 (9)	−0.0064 (9)
N3	0.0291 (12)	0.0227 (10)	0.0181 (10)	−0.0009 (9)	−0.0066 (9)	−0.0046 (9)
N5	0.0474 (16)	0.0400 (14)	0.0245 (13)	−0.0127 (12)	−0.0072 (12)	−0.0103 (11)
N6	0.0455 (15)	0.0335 (13)	0.0187 (11)	−0.0195 (12)	0.0010 (11)	−0.0053 (10)
N7	0.0578 (18)	0.0209 (11)	0.0243 (12)	−0.0148 (11)	−0.0024 (12)	−0.0026 (10)
N8	0.0333 (13)	0.0251 (11)	0.0183 (11)	−0.0070 (9)	−0.0026 (9)	−0.0066 (9)
C1	0.0303 (14)	0.0258 (12)	0.0207 (12)	−0.0084 (11)	−0.0047 (11)	−0.0042 (10)
C2	0.0317 (15)	0.0324 (14)	0.0253 (14)	−0.0118 (12)	−0.0053 (12)	−0.0056 (12)
C3	0.0359 (17)	0.0433 (17)	0.0279 (15)	−0.0126 (14)	−0.0067 (13)	−0.0074 (13)
C4	0.0383 (18)	0.058 (2)	0.0337 (17)	−0.0127 (16)	−0.0125 (15)	−0.0120 (16)
C5	0.0349 (17)	0.062 (2)	0.044 (2)	−0.0202 (16)	−0.0119 (15)	−0.0154 (17)
C6	0.0363 (18)	0.060 (2)	0.0382 (18)	−0.0259 (16)	−0.0007 (15)	−0.0132 (17)
C7	0.0348 (16)	0.0412 (16)	0.0294 (15)	−0.0190 (13)	−0.0045 (13)	−0.0076 (13)
C8	0.0253 (13)	0.0272 (12)	0.0255 (13)	−0.0055 (10)	0.0009 (11)	−0.0075 (11)
C9	0.029 (2)	0.0276 (16)	0.025 (2)	−0.0003 (14)	0.0001 (18)	−0.0078 (15)
C10	0.032 (2)	0.0351 (18)	0.033 (3)	−0.0009 (17)	−0.0033 (19)	−0.0059 (18)
C11	0.039 (2)	0.041 (2)	0.042 (3)	0.0065 (19)	−0.0085 (19)	−0.002 (2)
C12	0.052 (3)	0.0292 (18)	0.044 (2)	0.0018 (18)	−0.008 (2)	−0.0039 (18)
C13	0.048 (2)	0.0235 (16)	0.038 (2)	−0.0017 (17)	−0.0034 (18)	−0.0086 (17)
C14	0.033 (2)	0.0240 (15)	0.028 (2)	−0.0019 (15)	0.0007 (17)	−0.0084 (15)
O6	0.0370 (11)	0.0233 (9)	0.0362 (11)	−0.0066 (8)	−0.0067 (9)	−0.0084 (8)
C8B	0.0253 (13)	0.0272 (12)	0.0255 (13)	−0.0055 (10)	0.0009 (11)	−0.0075 (11)
C9B	0.031 (4)	0.028 (4)	0.030 (4)	−0.002 (4)	0.000 (4)	−0.007 (4)
C10B	0.034 (5)	0.031 (4)	0.033 (5)	−0.003 (4)	−0.003 (4)	−0.006 (4)
C11B	0.041 (5)	0.037 (4)	0.041 (5)	0.004 (4)	−0.005 (4)	−0.003 (4)
C12B	0.048 (5)	0.031 (4)	0.043 (5)	0.002 (4)	−0.003 (4)	−0.003 (4)
C13B	0.044 (5)	0.027 (4)	0.038 (5)	0.000 (4)	−0.001 (4)	−0.008 (4)
C14B	0.038 (4)	0.023 (4)	0.033 (4)	0.001 (4)	−0.002 (4)	−0.009 (4)
O6B	0.0370 (11)	0.0233 (9)	0.0362 (11)	−0.0066 (8)	−0.0067 (9)	−0.0084 (8)
C15	0.0248 (13)	0.0294 (13)	0.0240 (13)	−0.0030 (11)	−0.0038 (11)	−0.0096 (11)
C16	0.0264 (14)	0.0315 (14)	0.0225 (13)	−0.0061 (11)	−0.0039 (11)	−0.0087 (11)
C17	0.0265 (14)	0.0470 (17)	0.0292 (15)	−0.0135 (13)	−0.0056 (12)	−0.0077 (13)
C18	0.0329 (16)	0.0492 (18)	0.0311 (16)	−0.0194 (14)	−0.0016 (13)	−0.0079 (14)
C19	0.0377 (17)	0.0379 (16)	0.0244 (14)	−0.0150 (13)	−0.0009 (12)	−0.0051 (12)
C20	0.0289 (14)	0.0283 (13)	0.0230 (13)	−0.0078 (11)	−0.0033 (11)	−0.0083 (11)
C21	0.0247 (13)	0.0235 (12)	0.0231 (13)	−0.0056 (10)	−0.0027 (11)	−0.0079 (10)
C29	0.056 (2)	0.0441 (18)	0.0239 (15)	−0.0060 (16)	−0.0052 (15)	−0.0124 (14)
C30	0.050 (2)	0.071 (3)	0.0292 (17)	−0.003 (2)	−0.0079 (16)	−0.0188 (18)
C31	0.073 (3)	0.074 (3)	0.053 (3)	0.009 (2)	−0.028 (2)	−0.025 (2)
C32	0.076 (4)	0.093 (4)	0.066 (3)	0.015 (3)	−0.034 (3)	−0.022 (3)
C33	0.069 (3)	0.121 (5)	0.065 (3)	−0.001 (3)	−0.028 (3)	−0.047 (4)
C34	0.070 (3)	0.125 (5)	0.053 (3)	−0.032 (3)	−0.018 (3)	−0.030 (3)
C35	0.069 (3)	0.092 (4)	0.038 (2)	−0.029 (3)	−0.016 (2)	−0.018 (2)
C36	0.0476 (19)	0.0427 (17)	0.0214 (13)	−0.0285 (15)	0.0023 (13)	−0.0078 (13)
C37	0.059 (2)	0.0427 (18)	0.0250 (15)	−0.0263 (17)	0.0065 (15)	−0.0093 (14)
C38	0.056 (2)	0.069 (3)	0.0341 (19)	−0.021 (2)	0.0095 (18)	−0.0140 (19)
C39	0.068 (3)	0.081 (3)	0.046 (2)	−0.016 (3)	0.017 (2)	−0.017 (2)
C40	0.094 (4)	0.079 (3)	0.032 (2)	−0.017 (3)	0.023 (2)	−0.014 (2)
C41	0.094 (4)	0.061 (3)	0.0269 (18)	−0.014 (3)	0.007 (2)	−0.0038 (18)
C42	0.079 (3)	0.0399 (18)	0.0224 (15)	−0.0244 (19)	0.0101 (17)	−0.0090 (14)
C43	0.062 (2)	0.0290 (14)	0.0218 (14)	−0.0159 (15)	−0.0012 (14)	−0.0023 (12)
C44	0.062 (2)	0.0277 (14)	0.0289 (16)	−0.0190 (15)	0.0078 (15)	−0.0018 (13)
C45	0.085 (3)	0.0353 (18)	0.0362 (19)	−0.0248 (19)	0.018 (2)	−0.0027 (15)
C46	0.095 (4)	0.044 (2)	0.051 (2)	−0.037 (2)	0.027 (2)	−0.0017 (19)
C47	0.069 (3)	0.0364 (18)	0.062 (3)	−0.0290 (19)	0.020 (2)	−0.0083 (18)
C48	0.062 (2)	0.0325 (16)	0.0430 (19)	−0.0256 (16)	0.0133 (17)	−0.0097 (15)
C49	0.0500 (19)	0.0273 (14)	0.0330 (16)	−0.0178 (14)	0.0061 (14)	−0.0031 (13)
C50	0.0279 (14)	0.0271 (13)	0.0239 (13)	−0.0123 (11)	0.0025 (11)	−0.0097 (11)
C51	0.0370 (16)	0.0321 (14)	0.0266 (14)	−0.0165 (12)	0.0041 (12)	−0.0132 (12)
C52	0.081 (3)	0.047 (2)	0.0372 (19)	−0.040 (2)	0.0158 (19)	−0.0230 (17)
C53	0.104 (4)	0.050 (2)	0.040 (2)	−0.045 (2)	0.019 (2)	−0.0262 (18)
C54	0.064 (2)	0.0429 (18)	0.0294 (17)	−0.0191 (17)	0.0103 (16)	−0.0182 (15)
C55	0.0435 (18)	0.0301 (14)	0.0242 (14)	−0.0102 (13)	0.0009 (13)	−0.0077 (12)
C56	0.0281 (14)	0.0262 (12)	0.0234 (13)	−0.0063 (11)	−0.0036 (11)	−0.0076 (11)
N10	0.0419 (15)	0.0288 (12)	0.0289 (13)	−0.0031 (11)	−0.0040 (12)	−0.0122 (11)
C64	0.061 (2)	0.054 (2)	0.0369 (19)	−0.0157 (19)	−0.0080 (18)	−0.0205 (17)
C65	0.099 (4)	0.082 (3)	0.038 (2)	−0.027 (3)	−0.008 (2)	−0.033 (2)
C66	0.123 (5)	0.068 (3)	0.0276 (19)	−0.035 (3)	0.007 (2)	−0.018 (2)
C67	0.112 (4)	0.049 (2)	0.0289 (18)	−0.033 (2)	0.018 (2)	−0.0156 (17)
C68	0.068 (3)	0.0369 (17)	0.0285 (16)	−0.0182 (17)	0.0085 (16)	−0.0129 (14)
N12	0.0400 (16)	0.0394 (15)	0.0498 (19)	−0.0056 (13)	−0.0044 (14)	0.0088 (14)
C74	0.046 (2)	0.078 (3)	0.0345 (19)	−0.004 (2)	−0.0017 (17)	−0.010 (2)
C75	0.057 (3)	0.064 (3)	0.062 (3)	−0.007 (2)	0.005 (2)	−0.030 (2)
C76	0.051 (2)	0.046 (2)	0.071 (3)	0.0054 (18)	−0.003 (2)	0.009 (2)
C77	0.048 (2)	0.087 (3)	0.047 (2)	−0.010 (2)	−0.018 (2)	−0.002 (2)
C78	0.054 (2)	0.057 (2)	0.060 (3)	−0.014 (2)	−0.004 (2)	−0.024 (2)
N13	0.0579 (19)	0.0359 (14)	0.0361 (15)	−0.0205 (14)	0.0038 (14)	−0.0094 (12)
C79	0.068 (3)	0.0372 (18)	0.044 (2)	−0.0187 (18)	0.0079 (19)	−0.0139 (16)
C80	0.056 (2)	0.0338 (17)	0.065 (3)	−0.0142 (16)	−0.001 (2)	−0.0163 (18)
C81	0.065 (3)	0.048 (2)	0.089 (4)	−0.029 (2)	0.002 (3)	−0.004 (2)
C82	0.086 (4)	0.084 (4)	0.055 (3)	−0.051 (3)	0.018 (3)	−0.007 (3)
C83	0.063 (3)	0.058 (2)	0.0374 (19)	−0.028 (2)	0.0064 (18)	−0.0113 (18)
N14	0.116 (5)	0.105 (6)	0.071 (5)	−0.060 (5)	−0.020 (4)	−0.022 (4)
C84	0.117 (5)	0.107 (6)	0.073 (5)	−0.054 (5)	−0.015 (4)	−0.028 (5)
C85	0.130 (6)	0.110 (6)	0.074 (5)	−0.061 (5)	−0.007 (5)	−0.025 (5)
C86	0.147 (6)	0.115 (6)	0.089 (5)	−0.062 (5)	−0.006 (5)	−0.020 (5)
C87	0.143 (6)	0.118 (6)	0.085 (5)	−0.066 (5)	−0.011 (5)	−0.012 (5)
C88	0.133 (6)	0.117 (6)	0.077 (5)	−0.060 (5)	−0.009 (5)	−0.022 (5)
N22	0.115 (5)	0.116 (6)	0.073 (4)	−0.051 (5)	−0.024 (4)	−0.023 (4)
C124	0.118 (5)	0.112 (6)	0.077 (5)	−0.048 (5)	−0.016 (4)	−0.029 (5)
C125	0.128 (6)	0.111 (6)	0.076 (5)	−0.056 (5)	−0.009 (5)	−0.033 (4)
C126	0.149 (6)	0.117 (6)	0.081 (5)	−0.054 (5)	−0.004 (5)	−0.025 (5)
C127	0.152 (6)	0.117 (6)	0.083 (5)	−0.056 (5)	−0.010 (5)	−0.020 (5)
C128	0.135 (6)	0.118 (6)	0.075 (5)	−0.051 (5)	−0.015 (5)	−0.019 (5)
N15	0.101 (3)	0.066 (3)	0.053 (2)	−0.029 (2)	−0.009 (2)	0.019 (2)
C89	0.101 (4)	0.068 (5)	0.055 (4)	−0.024 (4)	−0.007 (4)	0.015 (4)
C90	0.119 (5)	0.069 (5)	0.056 (4)	−0.019 (5)	0.005 (4)	0.019 (4)
C91	0.117 (5)	0.082 (5)	0.069 (4)	−0.020 (4)	0.002 (4)	0.020 (4)
C92	0.112 (5)	0.088 (4)	0.081 (4)	−0.018 (4)	−0.002 (4)	0.018 (4)
C93	0.110 (4)	0.086 (4)	0.070 (4)	−0.015 (4)	−0.008 (4)	0.020 (4)
N23	0.101 (3)	0.066 (3)	0.053 (2)	−0.029 (2)	−0.009 (2)	0.019 (2)
C129	0.099 (4)	0.065 (5)	0.054 (4)	−0.027 (4)	−0.007 (4)	0.014 (4)
C130	0.112 (5)	0.067 (5)	0.052 (4)	−0.022 (5)	0.000 (4)	0.017 (4)
C131	0.113 (5)	0.079 (5)	0.065 (5)	−0.021 (5)	0.004 (5)	0.014 (4)
C132	0.110 (5)	0.089 (5)	0.075 (5)	−0.021 (5)	0.002 (5)	0.011 (5)
C133	0.105 (4)	0.077 (4)	0.065 (4)	−0.021 (4)	−0.010 (4)	0.016 (4)
N25	0.122 (7)	0.103 (7)	0.088 (7)	−0.015 (7)	−0.013 (7)	0.017 (7)
C139	0.112 (5)	0.085 (5)	0.071 (5)	−0.019 (5)	−0.008 (5)	0.015 (5)
C140	0.110 (4)	0.080 (4)	0.068 (4)	−0.020 (4)	−0.005 (4)	0.017 (4)
C141	0.117 (6)	0.090 (6)	0.080 (5)	−0.018 (5)	−0.004 (5)	0.014 (5)
C142	0.116 (7)	0.099 (7)	0.087 (7)	−0.019 (7)	−0.006 (7)	0.011 (7)
C143	0.122 (9)	0.107 (9)	0.088 (9)	−0.019 (9)	−0.012 (9)	0.013 (8)
N16	0.054 (2)	0.0524 (19)	0.0481 (19)	−0.0197 (16)	−0.0205 (16)	0.0000 (15)
C94	0.046 (2)	0.069 (3)	0.043 (2)	−0.030 (2)	−0.0094 (17)	−0.0005 (19)
C95	0.049 (2)	0.064 (3)	0.065 (3)	−0.020 (2)	−0.009 (2)	−0.003 (2)
C96	0.056 (3)	0.082 (4)	0.072 (3)	−0.003 (3)	−0.013 (2)	0.028 (3)
C97	0.070 (4)	0.107 (5)	0.063 (3)	0.003 (3)	0.001 (3)	0.035 (3)
C98	0.060 (3)	0.072 (3)	0.051 (3)	−0.008 (2)	−0.008 (2)	0.008 (2)
N17	0.109 (6)	0.090 (5)	0.070 (4)	−0.026 (5)	0.008 (5)	−0.015 (4)
C99	0.099 (6)	0.087 (5)	0.064 (5)	−0.031 (5)	0.011 (5)	−0.006 (4)
C100	0.092 (5)	0.098 (5)	0.061 (5)	−0.025 (5)	0.012 (4)	−0.010 (4)
C101	0.098 (5)	0.108 (6)	0.059 (5)	−0.025 (5)	0.012 (4)	−0.015 (5)
C102	0.105 (6)	0.117 (6)	0.065 (5)	−0.023 (5)	0.010 (5)	−0.010 (5)
C103	0.105 (6)	0.104 (6)	0.063 (4)	−0.023 (5)	0.014 (5)	−0.007 (5)
N24	0.112 (6)	0.099 (5)	0.068 (4)	−0.029 (5)	0.009 (5)	−0.011 (4)
C134	0.098 (6)	0.087 (4)	0.062 (5)	−0.027 (5)	0.006 (5)	−0.011 (4)
C135	0.096 (5)	0.103 (5)	0.061 (5)	−0.025 (4)	0.014 (4)	−0.011 (4)
C136	0.098 (5)	0.107 (6)	0.059 (5)	−0.028 (5)	0.010 (4)	−0.018 (5)
C137	0.111 (6)	0.113 (6)	0.067 (4)	−0.025 (5)	0.011 (5)	−0.008 (5)
C138	0.114 (6)	0.111 (6)	0.063 (4)	−0.037 (5)	0.007 (5)	−0.013 (5)
N18	0.060 (2)	0.071 (3)	0.065 (2)	−0.0194 (19)	−0.016 (2)	−0.021 (2)
C104	0.079 (3)	0.050 (2)	0.079 (4)	−0.021 (2)	−0.020 (3)	−0.007 (2)
C105	0.081 (4)	0.073 (3)	0.065 (3)	−0.014 (3)	−0.016 (3)	−0.001 (3)
C106	0.073 (3)	0.083 (4)	0.074 (4)	−0.014 (3)	−0.016 (3)	−0.035 (3)
C107	0.110 (5)	0.079 (4)	0.058 (3)	−0.026 (3)	−0.025 (3)	−0.009 (3)
C108	0.097 (5)	0.098 (4)	0.082 (4)	0.031 (4)	−0.047 (4)	−0.039 (4)
N19	0.067 (3)	0.081 (3)	0.078 (3)	−0.020 (2)	−0.002 (2)	−0.013 (2)
C109	0.047 (2)	0.070 (3)	0.069 (3)	0.000 (2)	0.002 (2)	−0.020 (3)
C110	0.074 (4)	0.140 (6)	0.064 (3)	−0.064 (4)	−0.008 (3)	−0.004 (3)
C111	0.076 (3)	0.065 (3)	0.041 (2)	−0.026 (2)	−0.001 (2)	−0.005 (2)
C112	0.041 (2)	0.051 (2)	0.066 (3)	−0.0053 (17)	−0.008 (2)	−0.002 (2)
C113	0.069 (3)	0.062 (3)	0.074 (3)	−0.014 (2)	−0.007 (3)	−0.008 (3)
N20	0.087 (4)	0.137 (6)	0.119 (5)	−0.044 (4)	−0.016 (4)	−0.051 (4)
C114	0.102 (5)	0.145 (7)	0.127 (6)	−0.074 (5)	0.031 (5)	−0.087 (6)
C115	0.080 (4)	0.121 (6)	0.186 (9)	−0.037 (4)	−0.020 (5)	−0.091 (7)
C116	0.096 (5)	0.116 (6)	0.099 (5)	−0.025 (4)	−0.048 (4)	−0.030 (4)
C117	0.107 (6)	0.146 (8)	0.082 (5)	−0.004 (5)	−0.019 (4)	−0.021 (5)
C118	0.072 (4)	0.124 (6)	0.101 (5)	−0.001 (4)	−0.028 (4)	−0.034 (5)
N21	0.086 (10)	0.092 (10)	0.056 (6)	−0.004 (7)	0.011 (7)	−0.012 (7)
C119	0.091 (11)	0.082 (10)	0.058 (8)	−0.016 (8)	0.009 (8)	−0.034 (7)
C120	0.089 (11)	0.092 (9)	0.057 (9)	0.000 (8)	0.001 (8)	−0.022 (8)
C121	0.081 (11)	0.085 (10)	0.066 (9)	0.000 (8)	−0.008 (8)	0.000 (8)
C122	0.075 (10)	0.073 (9)	0.055 (7)	0.006 (7)	−0.006 (7)	−0.023 (7)
C123	0.074 (9)	0.092 (9)	0.034 (6)	0.013 (7)	0.005 (6)	−0.020 (6)

### Bis(pyridinium) diaquadipyridinehexakis[µ3-salicylhydroximato(3-)]bis[µ2-salicylhydroximato(1-)]hexaaluminiumytterbium–pyridine–water (1/7.386/1) (3) Geometric parameters (Å, º)

**Table:**

Al1—O24	1.824 (2)	C20—C21	1.408 (4)
Al1—O2	1.872 (2)	C20—H20	0.9500
Al1—O9	1.903 (2)	C29—C30	1.487 (5)
Al1—O1	1.912 (2)	C30—C31	1.382 (7)
Al1—N3	1.966 (2)	C30—C35	1.397 (7)
Al1—N8	2.070 (2)	C31—C32	1.404 (7)
Al1—Yb1	3.6545 (9)	C31—H31	0.9500
Al2—O3	1.816 (2)	C32—C33	1.343 (9)
Al2—O17	1.876 (2)	C32—H32	0.9500
Al2—O4	1.890 (2)	C33—C34	1.355 (9)
Al2—O5	1.891 (2)	C33—H33	0.9500
Al2—O16	1.947 (2)	C34—C35	1.405 (7)
Al2—N1	1.995 (3)	C34—H34	0.9500
Al2—Yb1B	3.164 (2)	C36—C37	1.483 (4)
Al2—Yb1	3.4232 (10)	C37—C38	1.400 (6)
Al3—O6B	1.813 (2)	C37—C42	1.416 (5)
Al3—O6	1.813 (2)	C38—C39	1.389 (6)
Al3—O26	1.844 (6)	C38—H38	0.9500
Al3—O25	1.873 (4)	C39—C40	1.386 (7)
Al3—O26B	1.89 (2)	C39—H39	0.9500
Al3—N2	1.905 (2)	C40—C41	1.375 (7)
Al3—O28	2.002 (2)	C40—H40	0.9500
Al3—O25B	2.007 (19)	C41—C42	1.415 (5)
Al3—N10	2.126 (3)	C41—H41	0.9500
Al5—O18	1.806 (3)	C43—C44	1.478 (5)
Al5—O20	1.858 (2)	C44—C45	1.401 (5)
Al5—O19	1.906 (2)	C44—C49	1.411 (5)
Al5—O13	1.910 (2)	C45—C46	1.383 (5)
Al5—O14	1.944 (3)	C45—H45	0.9500
Al5—N6	2.022 (3)	C46—C47	1.395 (6)
Al5—Yb1B	3.182 (2)	C46—H46	0.9500
Al5—Yb1	3.3814 (10)	C47—C48	1.378 (5)
Al6—O21	1.803 (2)	C47—H47	0.9500
Al6—O23	1.833 (2)	C48—C49	1.414 (4)
Al6—O22	1.880 (2)	C48—H48	0.9500
Al6—N7	1.917 (3)	C50—C51	1.474 (4)
Al6—O12B	1.970 (13)	C51—C56	1.399 (4)
Al6—O29	1.995 (3)	C51—C52	1.404 (4)
Al6—N11	2.110 (4)	C52—C53	1.371 (5)
O1—N1	1.421 (3)	C52—H52	0.9500
O1—Yb1	2.3165 (19)	C53—C54	1.387 (5)
O1—Yb1B	2.348 (3)	C53—H53	0.9500
O2—C1	1.294 (4)	C54—C55	1.373 (5)
O3—C7	1.325 (4)	C54—H54	0.9500
O4—N2	1.397 (3)	C55—C56	1.411 (4)
O4—Yb1B	2.215 (3)	C55—H55	0.9500
O4—Yb1	2.397 (2)	N10—C64	1.339 (4)
O5—C8B	1.292 (3)	N10—C68	1.342 (5)
O5—C8	1.292 (3)	C64—C65	1.388 (6)
O7—N3	1.401 (3)	C64—H64	0.9500
O7—Al4B	1.716 (8)	C65—C66	1.387 (8)
O7—Al4	1.918 (3)	C65—H65	0.9500
O7—Yb1	2.273 (2)	C66—C67	1.360 (7)
O7—Yb1B	2.585 (3)	C66—H66	0.9500
O8—C15	1.301 (3)	C67—C68	1.391 (5)
O8—Al4	1.834 (3)	C67—H67	0.9500
O8—Al4B	2.172 (8)	C68—H68	0.9500
O9—C21	1.346 (3)	N12—C74	1.314 (6)
Yb1—O25	2.342 (4)	N12—C78	1.337 (5)
Yb1—O22	2.346 (2)	N12—H12A	0.8800
Yb1—O19	2.371 (2)	C74—C75	1.354 (6)
Yb1—O13	2.491 (2)	C74—H74	0.9500
Yb1—O16	2.494 (2)	C75—C76	1.374 (7)
Yb1—O10	2.580 (3)	C75—H75	0.9500
Yb1—N1	3.103 (2)	C76—C77	1.377 (7)
Al4—O27	1.857 (3)	C76—H76	0.9500
Al4—O11	1.895 (3)	C77—C78	1.362 (6)
Al4—O10	1.941 (3)	C77—H77	0.9500
Al4—N9	1.972 (4)	C78—H78	0.9500
O10—N4	1.370 (4)	N13—C83	1.323 (5)
N4—C22	1.315 (5)	N13—C79	1.339 (4)
N4—H4N	0.8800	C79—C80	1.363 (5)
O11—C22	1.284 (4)	C79—H79	0.9500
C22—C23	1.474 (5)	C80—C81	1.367 (6)
C23—C24	1.385 (6)	C80—H80	0.9500
C23—C28	1.409 (6)	C81—C82	1.390 (7)
C24—C25	1.392 (6)	C81—H81	0.9500
C24—H24	0.9500	C82—C83	1.368 (6)
C25—C26	1.392 (8)	C82—H82	0.9500
C25—H25	0.9500	C83—H83	0.9500
C26—C27	1.360 (9)	N14—C84	1.346 (12)
C26—H26	0.9500	N14—C88	1.352 (13)
C27—C28	1.410 (7)	C84—C85	1.349 (13)
C27—H27	0.9500	C84—H84	0.9500
C28—O12	1.356 (6)	C85—C86	1.357 (13)
O12—H12O	0.8400	C85—H85	0.9500
O25—N9	1.417 (5)	C86—C87	1.394 (13)
N9—C57	1.302 (5)	C86—H86	0.9500
O26—C57	1.310 (5)	C87—C88	1.365 (13)
C57—C58	1.477 (5)	C87—H87	0.9500
C58—C59	1.392 (5)	C88—H88	0.9500
C58—C63	1.411 (6)	N22—C124	1.356 (11)
C59—C60	1.382 (6)	N22—C128	1.360 (13)
C59—H59	0.9500	C124—C125	1.354 (13)
C60—C61	1.391 (7)	C124—H124	0.9500
C60—H60	0.9500	C125—C126	1.375 (13)
C61—C62	1.376 (6)	C125—H125	0.9500
C61—H61	0.9500	C126—C127	1.392 (12)
C62—C63	1.402 (5)	C126—H126	0.9500
C62—H62	0.9500	C127—C128	1.357 (12)
C63—O27	1.344 (5)	C127—H127	0.9500
N11—C73	1.338 (6)	C128—H128	0.9500
N11—C69	1.342 (5)	N15—C89	1.299 (11)
C69—C70	1.374 (6)	N15—C129	1.322 (11)
C69—H69	0.9500	N15—C133	1.348 (12)
C70—C71	1.365 (7)	N15—C93	1.381 (12)
C70—H70	0.9500	C89—N23	1.299 (11)
C71—C72	1.373 (7)	C89—C90	1.357 (12)
C71—H71	0.9500	C89—C130	1.45 (3)
C72—C73	1.390 (6)	C89—H89	0.9500
C72—H72	0.9500	C90—C129	1.25 (3)
C73—H73	0.9500	C90—C91	1.409 (14)
Yb1B—O16	2.175 (3)	C90—C131	1.63 (3)
Yb1B—O19	2.264 (3)	C90—H90	0.9500
Yb1B—O25B	2.322 (14)	C91—C131	0.87 (2)
Yb1B—O22	2.363 (3)	C91—C130	1.26 (3)
Yb1B—O13	2.382 (3)	C91—C92	1.427 (13)
Yb1B—O10B	2.602 (12)	C91—H91	0.9500
Yb1B—N6	2.853 (3)	C92—C132	1.17 (2)
Yb1B—N1	2.985 (3)	C92—C93	1.363 (12)
Al4B—O27B	1.847 (12)	C92—C131	1.65 (2)
Al4B—O11B	1.911 (11)	C92—C133	1.70 (2)
Al4B—O10B	1.951 (13)	C92—H92	0.9500
Al4B—N9B	1.966 (13)	C93—C133	0.73 (2)
O10B—N4B	1.373 (13)	C93—N23	1.381 (12)
N4B—C22B	1.320 (15)	C93—C132	1.58 (2)
N4B—H4B	0.89 (2)	C93—H93	0.9500
O11B—C22B	1.274 (14)	N23—C129	1.322 (11)
C22B—C23B	1.479 (14)	N23—C133	1.348 (12)
C23B—C24B	1.398 (15)	C129—C130	1.358 (13)
C23B—C28B	1.416 (15)	C129—H129	0.9500
C24B—C25B	1.392 (17)	C130—C131	1.394 (14)
C24B—H24B	0.9500	C130—H130	0.9500
C25B—C26B	1.372 (18)	C131—C132	1.398 (13)
C25B—H25B	0.9500	C131—H131	0.9500
C26B—C27B	1.317 (17)	C132—C133	1.361 (13)
C26B—H26B	0.9500	C132—H132	0.9500
C27B—C28B	1.428 (16)	C133—H133	0.9500
C27B—H27B	0.9500	N25—C139	1.3900
C28B—O12B	1.355 (15)	N25—C143	1.3900
O12B—H12P	0.8400	C139—C140	1.3900
O25B—N9B	1.410 (15)	C139—H139	0.9500
N9B—C57B	1.289 (14)	C140—C141	1.3900
O26B—C57B	1.321 (15)	C140—H140	0.9500
C57B—C58B	1.491 (14)	C141—C142	1.3900
C58B—C59B	1.369 (16)	C141—H141	0.9500
C58B—C63B	1.412 (16)	C142—C143	1.3900
C59B—C60B	1.401 (16)	C142—H142	0.9500
C59B—H59B	0.9500	C143—H143	0.9500
C60B—C61B	1.377 (18)	N16—C98	1.329 (6)
C60B—H60B	0.9500	N16—C94	1.342 (6)
C61B—C62B	1.369 (17)	N16—H16	0.8800
C61B—H61B	0.9500	C94—C95	1.362 (6)
C62B—C63B	1.395 (15)	C94—H94	0.9500
C62B—H62B	0.9500	C95—C96	1.381 (7)
C63B—O27B	1.338 (15)	C95—H95	0.9500
O13—N5	1.369 (3)	C96—C97	1.378 (8)
O14—C29	1.269 (5)	C96—H96	0.9500
O15—C35	1.347 (7)	C97—C98	1.375 (7)
O15—H15	0.8400	C97—H97	0.9500
O16—N6	1.439 (3)	C98—H98	0.9500
O17—C36	1.296 (4)	N17—C99	1.325 (12)
O18—C42	1.322 (5)	N17—C103	1.345 (12)
O19—N7	1.414 (3)	C99—C100	1.371 (13)
O20—C43	1.296 (4)	C99—H99	0.9500
O21—C49	1.327 (4)	C100—C101	1.350 (13)
O22—N8	1.422 (3)	C100—H100	0.9500
O23—C50	1.300 (3)	C101—C102	1.408 (13)
O24—C56	1.321 (3)	C101—H101	0.9500
O28—H28A	0.841 (19)	C102—C103	1.368 (12)
O28—H28B	0.828 (19)	C102—H102	0.9500
O29—H29A	0.910 (19)	C103—H103	0.9500
O29—H29B	0.943 (19)	N24—C134	1.314 (12)
O30—H30A	0.87 (2)	N24—C138	1.364 (12)
O30—H30B	0.865 (19)	C134—C135	1.359 (12)
N1—C1	1.313 (4)	C134—H134	0.9500
N2—C8B	1.314 (4)	C135—C136	1.341 (12)
N2—C8	1.314 (4)	C135—H135	0.9500
N3—C15	1.310 (4)	C136—C137	1.409 (13)
N5—C29	1.311 (5)	C136—H136	0.9500
N5—H5N	0.8800	C137—C138	1.371 (12)
N6—C36	1.313 (5)	C137—H137	0.9500
N7—C43	1.313 (4)	C138—H138	0.9500
N8—C50	1.308 (4)	N18—C108	1.289 (7)
C1—C2	1.471 (4)	N18—C104	1.359 (6)
C2—C3	1.400 (4)	C104—C105	1.338 (8)
C2—C7	1.411 (4)	C104—H104	0.9500
C3—C4	1.385 (5)	C105—C106	1.354 (8)
C3—H3	0.9500	C105—H105	0.9500
C4—C5	1.386 (5)	C106—C107	1.425 (8)
C4—H4	0.9500	C106—H106	0.9500
C5—C6	1.378 (5)	C107—C108	1.346 (8)
C5—H5	0.9500	C107—H107	0.9500
C6—C7	1.409 (4)	C108—H108	0.9500
C6—H6	0.9500	N19—C113	1.271 (7)
C8—C9	1.473 (5)	N19—C109	1.285 (7)
C9—C10	1.404 (5)	C109—C110	1.382 (8)
C9—C14	1.423 (6)	C109—H109	0.9500
C10—C11	1.389 (6)	C110—C111	1.495 (8)
C10—H10	0.9500	C110—H110	0.9500
C11—C12	1.389 (7)	C111—C112	1.340 (6)
C11—H11	0.9500	C111—H111	0.9500
C12—C13	1.389 (6)	C112—C113	1.349 (7)
C12—H12	0.9500	C112—H112	0.9500
C13—C14	1.406 (5)	C113—H113	0.9500
C13—H13	0.9500	N20—C118	1.319 (9)
C14—O6	1.332 (5)	N20—C114	1.322 (10)
C8B—C9B	1.493 (15)	C114—C115	1.413 (12)
C9B—C10B	1.404 (16)	C114—H114	0.9500
C9B—C14B	1.428 (15)	C115—C116	1.360 (11)
C10B—C11B	1.403 (16)	C115—H115	0.9500
C10B—H10B	0.9500	C116—C117	1.358 (11)
C11B—C12B	1.390 (17)	C116—H116	0.9500
C11B—H11B	0.9500	C117—C118	1.349 (11)
C12B—C13B	1.395 (16)	C117—H117	0.9500
C12B—H12B	0.9500	C118—H118	0.9500
C13B—C14B	1.405 (15)	N21—C123	1.319 (16)
C13B—H13B	0.9500	N21—C119	1.331 (15)
C14B—O6B	1.321 (15)	C119—C120	1.342 (17)
C15—C16	1.472 (4)	C119—H119	0.9500
C16—C17	1.394 (4)	C120—C121	1.404 (17)
C16—C21	1.414 (4)	C120—H120	0.9500
C17—C18	1.387 (5)	C121—C122	1.350 (16)
C17—H17	0.9500	C121—H121	0.9500
C18—C19	1.390 (5)	C122—C123	1.304 (16)
C18—H18	0.9500	C122—H122	0.9500
C19—C20	1.391 (4)	C123—H123	0.9500
C19—H19	0.9500		
			
O24—Al1—O2	96.38 (10)	C36—N6—Yb1B	126.8 (2)
O24—Al1—O9	89.13 (9)	O16—N6—Yb1B	48.13 (12)
O2—Al1—O9	88.93 (9)	Al5—N6—Yb1B	79.49 (10)
O24—Al1—O1	170.18 (10)	C43—N7—O19	110.7 (3)
O2—Al1—O1	81.94 (9)	C43—N7—Al6	128.6 (2)
O9—Al1—O1	100.48 (9)	O19—N7—Al6	119.85 (18)
O24—Al1—N3	100.81 (10)	C50—N8—O22	109.9 (2)
O2—Al1—N3	161.40 (10)	C50—N8—Al1	125.9 (2)
O9—Al1—N3	84.32 (9)	O22—N8—Al1	123.12 (16)
O1—Al1—N3	82.28 (10)	O2—C1—N1	121.0 (3)
O24—Al1—N8	86.31 (10)	O2—C1—C2	119.6 (2)
O2—Al1—N8	95.82 (10)	N1—C1—C2	119.4 (3)
O9—Al1—N8	173.75 (11)	C3—C2—C7	120.1 (3)
O1—Al1—N8	84.25 (9)	C3—C2—C1	117.9 (3)
N3—Al1—N8	92.33 (10)	C7—C2—C1	122.0 (3)
O24—Al1—Yb1	140.95 (7)	C4—C3—C2	121.3 (3)
O2—Al1—Yb1	108.57 (7)	C4—C3—H3	119.4
O9—Al1—Yb1	120.04 (7)	C2—C3—H3	119.4
O1—Al1—Yb1	33.57 (6)	C3—C4—C5	118.6 (3)
N3—Al1—Yb1	61.05 (7)	C3—C4—H4	120.7
N8—Al1—Yb1	62.22 (7)	C5—C4—H4	120.7
O3—Al2—O17	91.37 (10)	C6—C5—C4	121.3 (3)
O3—Al2—O4	174.82 (11)	C6—C5—H5	119.3
O17—Al2—O4	93.79 (10)	C4—C5—H5	119.3
O3—Al2—O5	97.61 (11)	C5—C6—C7	121.1 (3)
O17—Al2—O5	94.56 (11)	C5—C6—H6	119.4
O4—Al2—O5	82.47 (9)	C7—C6—H6	119.4
O3—Al2—O16	102.88 (11)	O3—C7—C6	119.1 (3)
O17—Al2—O16	81.90 (10)	O3—C7—C2	123.3 (3)
O4—Al2—O16	77.41 (9)	C6—C7—C2	117.5 (3)
O5—Al2—O16	159.26 (10)	O5—C8—N2	120.0 (3)
O3—Al2—N1	88.24 (10)	O5—C8—C9	120.7 (3)
O17—Al2—N1	169.91 (11)	N2—C8—C9	119.3 (3)
O4—Al2—N1	86.60 (10)	C10—C9—C14	120.6 (4)
O5—Al2—N1	95.48 (10)	C10—C9—C8	117.2 (4)
O16—Al2—N1	88.37 (10)	C14—C9—C8	122.1 (4)
O3—Al2—Yb1B	134.00 (10)	C11—C10—C9	120.7 (5)
O17—Al2—Yb1B	107.39 (9)	C11—C10—H10	119.6
O4—Al2—Yb1B	43.48 (7)	C9—C10—H10	119.6
O5—Al2—Yb1B	121.36 (8)	C12—C11—C10	119.3 (4)
O16—Al2—Yb1B	42.59 (7)	C12—C11—H11	120.3
N1—Al2—Yb1B	66.28 (8)	C10—C11—H11	120.3
O3—Al2—Yb1	134.64 (9)	C11—C12—C13	120.4 (4)
O17—Al2—Yb1	110.36 (8)	C11—C12—H12	119.8
O4—Al2—Yb1	42.46 (6)	C13—C12—H12	119.8
O5—Al2—Yb1	118.70 (7)	C12—C13—C14	122.1 (4)
O16—Al2—Yb1	45.82 (7)	C12—C13—H13	119.0
N1—Al2—Yb1	63.62 (7)	C14—C13—H13	119.0
O6—Al3—O26	92.96 (14)	O6—C14—C13	118.7 (4)
O6—Al3—O25	175.4 (2)	O6—C14—C9	124.5 (4)
O26—Al3—O25	85.23 (17)	C13—C14—C9	116.8 (4)
O6B—Al3—O26B	102.0 (5)	C14—O6—Al3	127.8 (2)
O6B—Al3—N2	91.85 (10)	O5—C8B—N2	120.0 (3)
O6—Al3—N2	91.85 (10)	O5—C8B—C9B	122.8 (8)
O26—Al3—N2	174.97 (16)	N2—C8B—C9B	116.6 (8)
O25—Al3—N2	89.86 (15)	C10B—C9B—C14B	116.7 (14)
O26B—Al3—N2	165.8 (5)	C10B—C9B—C8B	119.2 (14)
O6B—Al3—O28	89.73 (10)	C14B—C9B—C8B	123.9 (14)
O6—Al3—O28	89.73 (10)	C11B—C10B—C9B	122.1 (17)
O26—Al3—O28	90.3 (2)	C11B—C10B—H10B	118.9
O25—Al3—O28	86.0 (3)	C9B—C10B—H10B	118.9
O26B—Al3—O28	95.1 (9)	C12B—C11B—C10B	120.1 (16)
N2—Al3—O28	88.27 (10)	C12B—C11B—H11B	119.9
O6B—Al3—O25B	177.5 (5)	C10B—C11B—H11B	119.9
O26B—Al3—O25B	80.4 (7)	C11B—C12B—C13B	119.4 (15)
N2—Al3—O25B	85.8 (5)	C11B—C12B—H12B	120.3
O28—Al3—O25B	90.8 (11)	C13B—C12B—H12B	120.3
O6B—Al3—N10	91.69 (11)	C12B—C13B—C14B	120.6 (16)
O6—Al3—N10	91.69 (11)	C12B—C13B—H13B	119.7
O26—Al3—N10	91.0 (2)	C14B—C13B—H13B	119.7
O25—Al3—N10	92.6 (3)	O6B—C14B—C13B	116.7 (14)
O26B—Al3—N10	86.0 (9)	O6B—C14B—C9B	121.8 (15)
N2—Al3—N10	90.30 (11)	C13B—C14B—C9B	120.8 (14)
O28—Al3—N10	178.02 (10)	C14B—O6B—Al3	123.5 (10)
O25B—Al3—N10	87.7 (11)	O8—C15—N3	120.6 (3)
O18—Al5—O20	93.52 (12)	O8—C15—C16	120.7 (3)
O18—Al5—O19	174.94 (13)	N3—C15—C16	118.8 (3)
O20—Al5—O19	82.28 (11)	C17—C16—C21	120.1 (3)
O18—Al5—O13	101.34 (12)	C17—C16—C15	118.8 (3)
O20—Al5—O13	164.69 (13)	C21—C16—C15	120.9 (3)
O19—Al5—O13	83.04 (10)	C18—C17—C16	121.5 (3)
O18—Al5—O14	91.07 (13)	C18—C17—H17	119.3
O20—Al5—O14	94.35 (13)	C16—C17—H17	119.3
O19—Al5—O14	92.06 (12)	C17—C18—C19	119.0 (3)
O13—Al5—O14	81.83 (11)	C17—C18—H18	120.5
O18—Al5—N6	89.76 (12)	C19—C18—H18	120.5
O20—Al5—N6	100.12 (12)	C18—C19—C20	120.4 (3)
O19—Al5—N6	88.21 (11)	C18—C19—H19	119.8
O13—Al5—N6	83.74 (10)	C20—C19—H19	119.8
O14—Al5—N6	165.43 (12)	C19—C20—C21	121.4 (3)
O18—Al5—Yb1B	137.44 (10)	C19—C20—H20	119.3
O20—Al5—Yb1B	120.75 (9)	C21—C20—H20	119.3
O19—Al5—Yb1B	44.63 (7)	O9—C21—C20	119.9 (2)
O13—Al5—Yb1B	48.22 (7)	O9—C21—C16	122.6 (3)
O14—Al5—Yb1B	108.95 (10)	C20—C21—C16	117.5 (3)
N6—Al5—Yb1B	61.85 (8)	O14—C29—N5	117.6 (3)
O18—Al5—Yb1	139.67 (9)	O14—C29—C30	121.2 (3)
O20—Al5—Yb1	121.40 (8)	N5—C29—C30	121.3 (4)
O19—Al5—Yb1	42.87 (6)	C31—C30—C35	118.9 (4)
O13—Al5—Yb1	46.60 (7)	C31—C30—C29	117.8 (4)
O14—Al5—Yb1	104.43 (9)	C35—C30—C29	123.3 (4)
N6—Al5—Yb1	66.40 (8)	C30—C31—C32	120.7 (6)
O21—Al6—O23	94.02 (10)	C30—C31—H31	119.7
O21—Al6—O22	172.71 (12)	C32—C31—H31	119.7
O23—Al6—O22	83.91 (9)	C33—C32—C31	119.7 (6)
O21—Al6—N7	92.59 (11)	C33—C32—H32	120.2
O23—Al6—N7	173.38 (11)	C31—C32—H32	120.2
O22—Al6—N7	89.57 (10)	C32—C33—C34	121.2 (5)
O21—Al6—O12B	91.0 (4)	C32—C33—H33	119.4
O23—Al6—O12B	106.7 (5)	C34—C33—H33	119.4
O22—Al6—O12B	96.3 (4)	C33—C34—C35	120.9 (6)
N7—Al6—O12B	72.9 (5)	C33—C34—H34	119.5
O21—Al6—O29	86.42 (12)	C35—C34—H34	119.5
O23—Al6—O29	93.03 (12)	O15—C35—C30	119.0 (4)
O22—Al6—O29	86.71 (11)	O15—C35—C34	122.2 (5)
N7—Al6—O29	87.70 (13)	C30—C35—C34	118.7 (5)
O12B—Al6—O29	160.3 (5)	O17—C36—N6	121.9 (3)
O21—Al6—N11	89.41 (13)	O17—C36—C37	117.5 (3)
O23—Al6—N11	87.59 (13)	N6—C36—C37	120.6 (3)
O22—Al6—N11	97.48 (12)	C38—C37—C42	120.5 (3)
N7—Al6—N11	92.15 (14)	C38—C37—C36	117.7 (3)
O29—Al6—N11	175.81 (12)	C42—C37—C36	121.8 (4)
N1—O1—Al1	111.88 (15)	C39—C38—C37	121.3 (4)
N1—O1—Yb1	109.95 (14)	C39—C38—H38	119.3
Al1—O1—Yb1	119.28 (9)	C37—C38—H38	119.3
N1—O1—Yb1B	101.93 (14)	C40—C39—C38	118.5 (5)
Al1—O1—Yb1B	124.82 (11)	C40—C39—H39	120.7
C1—O2—Al1	113.03 (17)	C38—C39—H39	120.7
C7—O3—Al2	131.0 (2)	C41—C40—C39	121.1 (4)
N2—O4—Al2	112.07 (16)	C41—C40—H40	119.5
N2—O4—Yb1B	132.71 (16)	C39—C40—H40	119.5
Al2—O4—Yb1B	100.55 (10)	C40—C41—C42	122.1 (4)
N2—O4—Yb1	125.91 (15)	C40—C41—H41	119.0
Al2—O4—Yb1	105.37 (9)	C42—C41—H41	119.0
C8B—O5—Al2	112.26 (18)	O18—C42—C41	119.3 (4)
C8—O5—Al2	112.26 (18)	O18—C42—C37	124.2 (3)
N3—O7—Al4B	123.8 (3)	C41—C42—C37	116.5 (4)
N3—O7—Al4	111.32 (17)	O20—C43—N7	120.4 (3)
N3—O7—Yb1	119.47 (16)	O20—C43—C44	119.1 (3)
Al4—O7—Yb1	110.54 (11)	N7—C43—C44	120.5 (3)
N3—O7—Yb1B	117.93 (16)	C45—C44—C49	119.7 (3)
Al4B—O7—Yb1B	105.8 (3)	C45—C44—C43	117.9 (3)
C15—O8—Al4	112.8 (2)	C49—C44—C43	122.3 (3)
C15—O8—Al4B	106.2 (3)	C46—C45—C44	121.4 (4)
C21—O9—Al1	126.18 (17)	C46—C45—H45	119.3
O7—Yb1—O1	74.30 (7)	C44—C45—H45	119.3
O7—Yb1—O25	76.30 (15)	C45—C46—C47	119.3 (3)
O1—Yb1—O25	87.55 (19)	C45—C46—H46	120.4
O7—Yb1—O22	74.50 (8)	C47—C46—H46	120.4
O1—Yb1—O22	74.12 (7)	C48—C47—C46	120.2 (3)
O25—Yb1—O22	148.76 (18)	C48—C47—H47	119.9
O7—Yb1—O19	114.80 (8)	C46—C47—H47	119.9
O1—Yb1—O19	138.07 (7)	C47—C48—C49	121.6 (4)
O25—Yb1—O19	134.1 (2)	C47—C48—H48	119.2
O22—Yb1—O19	69.99 (7)	C49—C48—H48	119.2
O7—Yb1—O4	129.59 (7)	O21—C49—C44	124.0 (3)
O1—Yb1—O4	69.53 (7)	O21—C49—C48	118.2 (3)
O25—Yb1—O4	68.37 (12)	C44—C49—C48	117.8 (3)
O22—Yb1—O4	124.87 (7)	O23—C50—N8	121.4 (3)
O19—Yb1—O4	115.59 (8)	O23—C50—C51	118.3 (2)
O7—Yb1—O13	132.07 (7)	N8—C50—C51	120.1 (2)
O1—Yb1—O13	142.67 (7)	C56—C51—C52	119.5 (3)
O25—Yb1—O13	77.0 (2)	C56—C51—C50	122.5 (3)
O22—Yb1—O13	132.08 (7)	C52—C51—C50	117.7 (3)
O19—Yb1—O13	62.64 (7)	C53—C52—C51	121.6 (3)
O4—Yb1—O13	73.19 (7)	C53—C52—H52	119.2
O7—Yb1—O16	147.45 (7)	C51—C52—H52	119.2
O1—Yb1—O16	82.90 (7)	C52—C53—C54	119.0 (3)
O25—Yb1—O16	126.26 (12)	C52—C53—H53	120.5
O22—Yb1—O16	77.13 (8)	C54—C53—H53	120.5
O19—Yb1—O16	68.73 (8)	C55—C54—C53	120.5 (3)
O4—Yb1—O16	58.73 (7)	C55—C54—H54	119.8
O13—Yb1—O16	79.54 (7)	C53—C54—H54	119.8
O7—Yb1—O10	58.97 (9)	C54—C55—C56	121.5 (3)
O1—Yb1—O10	131.55 (8)	C54—C55—H55	119.3
O25—Yb1—O10	70.62 (14)	C56—C55—H55	119.3
O22—Yb1—O10	102.81 (9)	O24—C56—C51	123.4 (3)
O19—Yb1—O10	78.10 (9)	O24—C56—C55	118.8 (3)
O4—Yb1—O10	132.32 (8)	C51—C56—C55	117.7 (3)
O13—Yb1—O10	74.86 (9)	C64—N10—C68	117.4 (3)
O16—Yb1—O10	144.78 (8)	C64—N10—Al3	120.0 (3)
O7—Yb1—N1	98.65 (7)	C68—N10—Al3	122.5 (2)
O1—Yb1—N1	25.49 (7)	N10—C64—C65	122.2 (4)
O25—Yb1—N1	100.61 (17)	N10—C64—H64	118.9
O22—Yb1—N1	73.29 (7)	C65—C64—H64	118.9
O19—Yb1—N1	119.77 (7)	C66—C65—C64	119.2 (4)
O4—Yb1—N1	56.22 (7)	C66—C65—H65	120.4
O13—Yb1—N1	125.01 (7)	C64—C65—H65	120.4
O16—Yb1—N1	57.56 (6)	C67—C66—C65	119.4 (4)
O10—Yb1—N1	157.00 (8)	C67—C66—H66	120.3
O7—Yb1—Al5	141.26 (5)	C65—C66—H66	120.3
O1—Yb1—Al5	141.74 (5)	C66—C67—C68	118.1 (4)
O25—Yb1—Al5	110.85 (19)	C66—C67—H67	120.9
O22—Yb1—Al5	98.90 (5)	C68—C67—H67	120.9
O19—Yb1—Al5	33.17 (6)	N10—C68—C67	123.7 (4)
O4—Yb1—Al5	86.01 (5)	N10—C68—H68	118.1
O13—Yb1—Al5	33.84 (5)	C67—C68—H68	118.1
O16—Yb1—Al5	59.03 (5)	C74—N12—C78	121.3 (4)
O10—Yb1—Al5	86.68 (7)	C74—N12—H12A	119.4
N1—Yb1—Al5	116.25 (5)	C78—N12—H12A	119.4
O7—Yb1—Al2	132.73 (5)	N12—C74—C75	121.2 (4)
O1—Yb1—Al2	58.47 (5)	N12—C74—H74	119.4
O25—Yb1—Al2	98.56 (11)	C75—C74—H74	119.4
O22—Yb1—Al2	93.16 (6)	C74—C75—C76	119.3 (4)
O19—Yb1—Al2	102.37 (6)	C74—C75—H75	120.4
O4—Yb1—Al2	32.17 (5)	C76—C75—H75	120.4
O13—Yb1—Al2	90.13 (6)	C75—C76—C77	118.8 (4)
O16—Yb1—Al2	34.05 (5)	C75—C76—H76	120.6
O10—Yb1—Al2	162.98 (7)	C77—C76—H76	120.6
N1—Yb1—Al2	35.16 (5)	C78—C77—C76	119.5 (4)
Al5—Yb1—Al2	85.07 (3)	C78—C77—H77	120.3
O8—Al4—O27	92.16 (13)	C76—C77—H77	120.3
O8—Al4—O11	99.10 (14)	N12—C78—C77	119.9 (4)
O27—Al4—O11	86.45 (14)	N12—C78—H78	120.0
O8—Al4—O7	82.50 (12)	C77—C78—H78	120.0
O27—Al4—O7	174.12 (14)	C83—N13—C79	118.0 (3)
O11—Al4—O7	96.81 (13)	N13—C79—C80	123.8 (4)
O8—Al4—O10	159.52 (15)	N13—C79—H79	118.1
O27—Al4—O10	108.32 (15)	C80—C79—H79	118.1
O11—Al4—O10	82.53 (13)	C79—C80—C81	117.6 (4)
O7—Al4—O10	77.04 (13)	C79—C80—H80	121.2
O8—Al4—N9	97.65 (14)	C81—C80—H80	121.2
O27—Al4—N9	86.73 (16)	C80—C81—C82	119.5 (4)
O11—Al4—N9	162.12 (17)	C80—C81—H81	120.2
O7—Al4—N9	91.51 (15)	C82—C81—H81	120.2
O10—Al4—N9	83.97 (14)	C83—C82—C81	118.7 (4)
O8—Al4—Yb1	114.10 (10)	C83—C82—H82	120.7
O27—Al4—Yb1	144.69 (12)	C81—C82—H82	120.7
O11—Al4—Yb1	110.62 (10)	N13—C83—C82	122.4 (4)
O7—Al4—Yb1	38.10 (7)	N13—C83—H83	118.8
O10—Al4—Yb1	47.69 (10)	C82—C83—H83	118.8
N9—Al4—Yb1	67.50 (11)	C84—N14—C88	117.2 (13)
N4—O10—Al4	109.2 (2)	N14—C84—C85	121.0 (13)
N4—O10—Yb1	119.5 (2)	N14—C84—H84	119.5
Al4—O10—Yb1	98.49 (13)	C85—C84—H84	119.5
C22—N4—O10	116.7 (3)	C84—C85—C86	120.8 (13)
C22—N4—H4N	121.7	C84—C85—H85	119.6
O10—N4—H4N	121.7	C86—C85—H85	119.6
C22—O11—Al4	113.7 (2)	C85—C86—C87	120.5 (12)
O11—C22—N4	117.7 (3)	C85—C86—H86	119.8
O11—C22—C23	120.2 (4)	C87—C86—H86	119.8
N4—C22—C23	121.9 (3)	C88—C87—C86	114.9 (12)
C24—C23—C28	119.3 (4)	C88—C87—H87	122.5
C24—C23—C22	117.7 (4)	C86—C87—H87	122.5
C28—C23—C22	122.8 (4)	N14—C88—C87	125.3 (12)
C23—C24—C25	121.6 (5)	N14—C88—H88	117.4
C23—C24—H24	119.2	C87—C88—H88	117.4
C25—C24—H24	119.2	C124—N22—C128	115.1 (13)
C24—C25—C26	118.2 (5)	C125—C124—N22	124.6 (14)
C24—C25—H25	120.9	C125—C124—H124	117.7
C26—C25—H25	120.9	N22—C124—H124	117.7
C27—C26—C25	121.8 (5)	C124—C125—C126	117.5 (11)
C27—C26—H26	119.1	C124—C125—H125	121.2
C25—C26—H26	119.1	C126—C125—H125	121.2
C26—C27—C28	120.3 (5)	C125—C126—C127	119.5 (11)
C26—C27—H27	119.9	C125—C126—H126	120.2
C28—C27—H27	119.9	C127—C126—H126	120.2
O12—C28—C23	117.9 (4)	C128—C127—C126	116.9 (12)
O12—C28—C27	123.2 (4)	C128—C127—H127	121.5
C23—C28—C27	118.9 (5)	C126—C127—H127	121.5
C28—O12—H12O	109.5	C127—C128—N22	122.8 (12)
N9—O25—Al3	109.1 (3)	C127—C128—H128	118.6
N9—O25—Yb1	117.8 (3)	N22—C128—H128	118.6
Al3—O25—Yb1	131.0 (2)	C89—N15—C133	116.8 (12)
C57—N9—O25	112.3 (3)	C129—N15—C133	115.2 (11)
C57—N9—Al4	129.3 (3)	C89—N15—C93	118.3 (10)
O25—N9—Al4	117.8 (3)	C129—N15—C93	111.7 (11)
C57—O26—Al3	110.3 (3)	N23—C89—C90	129.2 (15)
N9—C57—O26	120.8 (4)	N15—C89—C90	129.2 (15)
N9—C57—C58	119.9 (4)	N23—C89—C130	122.8 (15)
O26—C57—C58	119.4 (4)	N15—C89—C130	122.8 (15)
C59—C58—C63	119.7 (4)	N23—C89—H89	115.4
C59—C58—C57	118.7 (4)	N15—C89—H89	115.4
C63—C58—C57	121.6 (4)	C90—C89—H89	115.4
C60—C59—C58	120.9 (4)	C130—C89—H89	121.2
C60—C59—H59	119.5	C129—C90—C91	108.3 (15)
C58—C59—H59	119.5	C89—C90—C91	112.6 (13)
C59—C60—C61	119.4 (4)	C129—C90—C131	107.0 (13)
C59—C60—H60	120.3	C89—C90—C131	106.5 (15)
C61—C60—H60	120.3	C129—C90—H90	127.4
C62—C61—C60	120.6 (4)	C89—C90—H90	123.7
C62—C61—H61	119.7	C91—C90—H90	123.7
C60—C61—H61	119.7	C131—C90—H90	120.5
C61—C62—C63	120.8 (4)	C131—C91—C130	80 (2)
C61—C62—H62	119.6	C131—C91—C90	88 (2)
C63—C62—H62	119.6	C131—C91—C92	88.5 (17)
O27—C63—C62	118.9 (4)	C130—C91—C92	121.7 (14)
O27—C63—C58	122.6 (3)	C90—C91—C92	121.4 (11)
C62—C63—C58	118.5 (4)	C131—C91—C132	48.9 (13)
C63—O27—Al4	126.3 (2)	C130—C91—C132	101.4 (15)
C73—N11—C69	117.3 (4)	C90—C91—C132	106.4 (14)
C73—N11—Al6	122.4 (3)	C131—C91—H91	93.4
C69—N11—Al6	119.4 (3)	C130—C91—H91	118.3
N11—C69—C70	123.0 (4)	C90—C91—H91	119.3
N11—C69—H69	118.5	C92—C91—H91	119.3
C70—C69—H69	118.5	C132—C91—H91	119.4
C71—C70—C69	119.4 (4)	C132—C92—C93	76.9 (13)
C71—C70—H70	120.3	C132—C92—C91	87.6 (14)
C69—C70—H70	120.3	C93—C92—C91	118.6 (12)
C70—C71—C72	118.8 (4)	C132—C92—C131	56.4 (10)
C70—C71—H71	120.6	C93—C92—C131	110.4 (14)
C72—C71—H71	120.6	C132—C92—C133	52.7 (9)
C71—C72—C73	119.0 (4)	C91—C92—C133	109.5 (12)
C71—C72—H72	120.5	C131—C92—C133	91.2 (12)
C73—C72—H72	120.5	C132—C92—H92	105.2
N11—C73—C72	122.5 (4)	C93—C92—H92	120.7
N11—C73—H73	118.7	C91—C92—H92	120.7
C72—C73—H73	118.7	C131—C92—H92	119.5
O16—Yb1B—O4	66.27 (9)	C133—C92—H92	124.4
O16—Yb1B—O19	76.46 (10)	C133—C93—C92	105 (2)
O4—Yb1B—O19	128.51 (11)	C133—C93—N15	72.1 (13)
O16—Yb1B—O25B	133.0 (5)	C92—C93—N15	119.8 (11)
O4—Yb1B—O25B	68.1 (5)	C133—C93—N23	72.1 (13)
O19—Yb1B—O25B	127.3 (9)	C92—C93—N23	119.8 (11)
O16—Yb1B—O1	89.51 (10)	C133—C93—C132	59.2 (16)
O4—Yb1B—O1	72.13 (9)	C92—C93—C132	45.9 (10)
O19—Yb1B—O1	143.23 (11)	N15—C93—C132	106.2 (12)
O25B—Yb1B—O1	86.9 (10)	N23—C93—C132	106.2 (12)
O16—Yb1B—O22	83.30 (10)	C133—C93—H93	92.9
O4—Yb1B—O22	133.37 (11)	C92—C93—H93	120.1
O19—Yb1B—O22	71.51 (9)	N15—C93—H93	120.1
O25B—Yb1B—O22	139.0 (8)	N23—C93—H93	120.1
O1—Yb1B—O22	73.22 (9)	C132—C93—H93	114.5
O16—Yb1B—O13	88.70 (10)	C89—N23—C133	116.8 (12)
O4—Yb1B—O13	78.63 (9)	C129—N23—C133	115.2 (11)
O19—Yb1B—O13	65.92 (9)	C89—N23—C93	118.3 (10)
O25B—Yb1B—O13	71.9 (10)	C129—N23—C93	111.7 (11)
O1—Yb1B—O13	148.73 (11)	C90—C129—N23	137.2 (18)
O22—Yb1B—O13	137.38 (11)	C90—C129—N15	137.2 (18)
O16—Yb1B—O7	147.90 (11)	N23—C129—C130	128.3 (15)
O4—Yb1B—O7	123.19 (10)	N15—C129—C130	128.3 (15)
O19—Yb1B—O7	107.39 (10)	C90—C129—H129	106.8
O25B—Yb1B—O7	70.8 (7)	N23—C129—H129	115.8
O1—Yb1B—O7	68.17 (8)	N15—C129—H129	115.8
O22—Yb1B—O7	68.65 (9)	C130—C129—H129	115.8
O13—Yb1B—O7	122.43 (10)	C91—C130—C129	111 (2)
O16—Yb1B—O10B	141.2 (3)	C129—C130—C131	115.6 (13)
O4—Yb1B—O10B	135.1 (3)	C91—C130—C89	116.5 (16)
O19—Yb1B—O10B	65.3 (3)	C131—C130—C89	115.1 (13)
O25B—Yb1B—O10B	71.9 (6)	C91—C130—H130	113.5
O1—Yb1B—O10B	125.4 (3)	C129—C130—H130	122.2
O22—Yb1B—O10B	90.7 (3)	C131—C130—H130	122.2
O13—Yb1B—O10B	70.1 (3)	C89—C130—H130	122.1
O7—Yb1B—O10B	57.5 (3)	C91—C131—C130	62 (2)
O16—Yb1B—N6	29.51 (8)	C91—C131—C132	103.3 (19)
O4—Yb1B—N6	66.74 (8)	C130—C131—C132	118.0 (12)
O19—Yb1B—N6	63.39 (9)	C91—C131—C90	59.8 (17)
O25B—Yb1B—N6	117.9 (8)	C132—C131—C90	117.1 (12)
O1—Yb1B—N6	116.03 (10)	C91—C131—C92	59.9 (15)
O22—Yb1B—N6	103.01 (10)	C130—C131—C92	100.4 (16)
O13—Yb1B—N6	59.38 (8)	C90—C131—C92	97.9 (14)
O7—Yb1B—N6	169.76 (11)	C91—C131—H131	103.1
O10B—Yb1B—N6	118.4 (3)	C130—C131—H131	121.0
O16—Yb1B—N1	62.01 (8)	C132—C131—H131	121.0
O4—Yb1B—N1	59.49 (8)	C90—C131—H131	121.9
O19—Yb1B—N1	129.07 (10)	C92—C131—H131	121.5
O25B—Yb1B—N1	103.0 (8)	C92—C132—C133	84.3 (13)
O1—Yb1B—N1	27.75 (7)	C92—C132—C131	79.5 (14)
O22—Yb1B—N1	75.43 (9)	C133—C132—C131	120.5 (13)
O13—Yb1B—N1	135.38 (10)	C92—C132—C93	57.2 (10)
O7—Yb1B—N1	94.92 (8)	C131—C132—C93	112.6 (13)
O10B—Yb1B—N1	152.3 (3)	C92—C132—C91	52.2 (9)
N6—Yb1B—N1	88.41 (8)	C133—C132—C91	107.1 (12)
O16—Yb1B—Al2	37.28 (6)	C93—C132—C91	90.0 (11)
O4—Yb1B—Al2	35.96 (6)	C92—C132—H132	106.4
O19—Yb1B—Al2	113.36 (9)	C133—C132—H132	119.8
O25B—Yb1B—Al2	102.6 (5)	C131—C132—H132	119.8
O1—Yb1B—Al2	63.05 (7)	C93—C132—H132	120.5
O22—Yb1B—Al2	99.68 (8)	C91—C132—H132	126.5
O13—Yb1B—Al2	98.71 (8)	C93—C133—N23	77.1 (13)
O7—Yb1B—Al2	131.09 (8)	C93—C133—N15	77.1 (13)
O10B—Yb1B—Al2	168.5 (3)	C93—C133—C132	93 (2)
N6—Yb1B—Al2	54.65 (6)	N23—C133—C132	122.2 (11)
N1—Yb1B—Al2	37.72 (5)	N15—C133—C132	122.2 (11)
O7—Al4B—O27B	164.1 (6)	C93—C133—C92	50.8 (15)
O7—Al4B—O11B	95.3 (5)	N23—C133—C92	101.6 (10)
O27B—Al4B—O11B	89.7 (6)	N15—C133—C92	101.6 (10)
O7—Al4B—O10B	85.5 (5)	C93—C133—H133	99.7
O27B—Al4B—O10B	110.2 (7)	N23—C133—H133	118.9
O11B—Al4B—O10B	82.1 (5)	N15—C133—H133	118.9
O7—Al4B—N9B	91.8 (6)	C132—C133—H133	118.9
O27B—Al4B—N9B	87.1 (7)	C92—C133—H133	123.3
O11B—Al4B—N9B	164.7 (8)	C139—N25—C143	120.0
O10B—Al4B—N9B	85.0 (6)	N25—C139—C140	120.0
O7—Al4B—O8	78.1 (3)	N25—C139—H139	120.0
O27B—Al4B—O8	86.1 (5)	C140—C139—H139	120.0
O11B—Al4B—O8	101.9 (5)	C141—C140—C139	120.0
O10B—Al4B—O8	163.3 (6)	C141—C140—H140	120.0
N9B—Al4B—O8	92.8 (6)	C139—C140—H140	120.0
O7—Al4B—Yb1B	45.8 (2)	C140—C141—C142	120.0
O27B—Al4B—Yb1B	145.2 (6)	C140—C141—H141	120.0
O11B—Al4B—Yb1B	109.5 (4)	C142—C141—H141	120.0
O10B—Al4B—Yb1B	47.9 (4)	C141—C142—C143	120.0
N9B—Al4B—Yb1B	66.6 (5)	C141—C142—H142	120.0
O8—Al4B—Yb1B	116.3 (3)	C143—C142—H142	120.0
N4B—O10B—Al4B	105.8 (9)	C142—C143—N25	120.0
N4B—O10B—Yb1B	103.2 (9)	C142—C143—H143	120.0
Al4B—O10B—Yb1B	98.3 (5)	N25—C143—H143	120.0
C22B—N4B—O10B	117.8 (12)	C98—N16—C94	122.4 (4)
C22B—N4B—H4B	111 (5)	C98—N16—H16	118.8
O10B—N4B—H4B	121 (6)	C94—N16—H16	118.8
C22B—O11B—Al4B	109.7 (9)	N16—C94—C95	120.1 (4)
O11B—C22B—N4B	116.4 (11)	N16—C94—H94	120.0
O11B—C22B—C23B	122.7 (12)	C95—C94—H94	120.0
N4B—C22B—C23B	120.5 (11)	C94—C95—C96	118.5 (5)
C24B—C23B—C28B	119.6 (12)	C94—C95—H95	120.8
C24B—C23B—C22B	117.1 (12)	C96—C95—H95	120.8
C28B—C23B—C22B	123.1 (12)	C97—C96—C95	120.7 (5)
C25B—C24B—C23B	120.3 (14)	C97—C96—H96	119.6
C25B—C24B—H24B	119.9	C95—C96—H96	119.6
C23B—C24B—H24B	119.9	C98—C97—C96	118.4 (5)
C26B—C25B—C24B	116.0 (15)	C98—C97—H97	120.8
C26B—C25B—H25B	122.0	C96—C97—H97	120.8
C24B—C25B—H25B	122.0	N16—C98—C97	119.9 (5)
C27B—C26B—C25B	127.0 (17)	N16—C98—H98	120.1
C27B—C26B—H26B	116.5	C97—C98—H98	120.1
C25B—C26B—H26B	116.5	C99—N17—C103	120.7 (13)
C26B—C27B—C28B	116.0 (15)	N17—C99—C100	122.2 (14)
C26B—C27B—H27B	122.0	N17—C99—H99	118.9
C28B—C27B—H27B	122.0	C100—C99—H99	118.9
O12B—C28B—C23B	119.6 (13)	C101—C100—C99	117.7 (13)
O12B—C28B—C27B	121.5 (13)	C101—C100—H100	121.1
C23B—C28B—C27B	119.0 (12)	C99—C100—H100	121.1
C28B—O12B—Al6	133.9 (14)	C100—C101—C102	119.3 (14)
C28B—O12B—H12P	109.5	C100—C101—H101	120.4
Al6—O12B—H12P	113.8	C102—C101—H101	120.4
N9B—O25B—Al3	109.7 (10)	C103—C102—C101	118.5 (14)
N9B—O25B—Yb1B	118.4 (11)	C103—C102—H102	120.8
Al3—O25B—Yb1B	128.6 (10)	C101—C102—H102	120.8
C57B—N9B—O25B	111.6 (12)	N17—C103—C102	119.5 (13)
C57B—N9B—Al4B	129.0 (12)	N17—C103—H103	120.3
O25B—N9B—Al4B	118.1 (11)	C102—C103—H103	120.3
C57B—O26B—Al3	112.3 (14)	C134—N24—C138	118.7 (12)
N9B—C57B—O26B	122.1 (15)	N24—C134—C135	121.6 (13)
N9B—C57B—C58B	120.6 (13)	N24—C134—H134	119.2
O26B—C57B—C58B	117.0 (15)	C135—C134—H134	119.2
C59B—C58B—C63B	121.5 (13)	C136—C135—C134	119.8 (12)
C59B—C58B—C57B	119.0 (13)	C136—C135—H135	120.1
C63B—C58B—C57B	119.3 (12)	C134—C135—H135	120.1
C58B—C59B—C60B	120.6 (16)	C135—C136—C137	119.3 (13)
C58B—C59B—H59B	119.7	C135—C136—H136	120.3
C60B—C59B—H59B	119.7	C137—C136—H136	120.3
C61B—C60B—C59B	118.6 (16)	C138—C137—C136	114.8 (13)
C61B—C60B—H60B	120.7	C138—C137—H137	122.6
C59B—C60B—H60B	120.7	C136—C137—H137	122.6
C62B—C61B—C60B	120.6 (15)	N24—C138—C137	122.1 (12)
C62B—C61B—H61B	119.7	N24—C138—H138	118.9
C60B—C61B—H61B	119.7	C137—C138—H138	118.9
C61B—C62B—C63B	122.6 (15)	C108—N18—C104	112.4 (5)
C61B—C62B—H62B	118.7	C105—C104—N18	125.2 (5)
C63B—C62B—H62B	118.7	C105—C104—H104	117.4
O27B—C63B—C62B	119.3 (14)	N18—C104—H104	117.4
O27B—C63B—C58B	124.5 (12)	C104—C105—C106	119.0 (6)
C62B—C63B—C58B	116.1 (14)	C104—C105—H105	120.5
C63B—O27B—Al4B	124.7 (10)	C106—C105—H105	120.5
N5—O13—Al5	110.14 (18)	C105—C106—C107	118.9 (5)
N5—O13—Yb1B	125.58 (19)	C105—C106—H106	120.6
Al5—O13—Yb1B	95.07 (10)	C107—C106—H106	120.6
N5—O13—Yb1	118.37 (18)	C108—C107—C106	113.9 (6)
Al5—O13—Yb1	99.56 (9)	C108—C107—H107	123.1
C29—O14—Al5	112.9 (2)	C106—C107—H107	123.1
C35—O15—H15	109.5	N18—C108—C107	130.5 (6)
N6—O16—Al2	109.21 (17)	N18—C108—H108	114.7
N6—O16—Yb1B	102.36 (16)	C107—C108—H108	114.7
Al2—O16—Yb1B	100.12 (10)	C113—N19—C109	116.6 (5)
N6—O16—Yb1	104.17 (15)	N19—C109—C110	127.2 (5)
Al2—O16—Yb1	100.14 (9)	N19—C109—H109	116.4
C36—O17—Al2	111.3 (2)	C110—C109—H109	116.4
C42—O18—Al5	133.2 (2)	C109—C110—C111	113.7 (5)
N7—O19—Al5	109.60 (18)	C109—C110—H110	123.1
N7—O19—Yb1B	122.83 (17)	C111—C110—H110	123.1
Al5—O19—Yb1B	99.10 (11)	C112—C111—C110	115.8 (5)
N7—O19—Yb1	125.12 (16)	C112—C111—H111	122.1
Al5—O19—Yb1	103.96 (9)	C110—C111—H111	122.1
C43—O20—Al5	111.5 (2)	C111—C112—C113	120.9 (5)
C49—O21—Al6	129.6 (2)	C111—C112—H112	119.6
N8—O22—Al6	111.98 (15)	C113—C112—H112	119.6
N8—O22—Yb1	117.33 (14)	N19—C113—C112	125.7 (6)
Al6—O22—Yb1	130.17 (11)	N19—C113—H113	117.1
N8—O22—Yb1B	120.99 (15)	C112—C113—H113	117.1
Al6—O22—Yb1B	124.95 (11)	C118—N20—C114	117.6 (8)
C50—O23—Al6	112.62 (18)	N20—C114—C115	122.5 (7)
C56—O24—Al1	130.45 (19)	N20—C114—H114	118.8
Al3—O28—H28A	115 (3)	C115—C114—H114	118.8
Al3—O28—H28B	118 (3)	C116—C115—C114	118.1 (7)
H28A—O28—H28B	115 (4)	C116—C115—H115	121.0
Al6—O29—H29A	109 (3)	C114—C115—H115	121.0
Al6—O29—H29B	132 (3)	C117—C116—C115	117.9 (9)
H29A—O29—H29B	113 (4)	C117—C116—H116	121.0
H30A—O30—H30B	115 (10)	C115—C116—H116	121.0
C1—N1—O1	110.2 (2)	C118—C117—C116	121.1 (9)
C1—N1—Al2	130.3 (2)	C118—C117—H117	119.4
O1—N1—Al2	119.54 (16)	C116—C117—H117	119.4
C1—N1—Yb1B	145.7 (2)	N20—C118—C117	122.7 (8)
O1—N1—Yb1B	50.32 (11)	N20—C118—H118	118.6
Al2—N1—Yb1B	76.01 (8)	C117—C118—H118	118.6
C1—N1—Yb1	142.61 (19)	C123—N21—C119	118.9 (14)
Al2—N1—Yb1	81.22 (8)	N21—C119—C120	117.5 (15)
C8B—N2—O4	112.1 (2)	N21—C119—H119	121.2
C8—N2—O4	112.1 (2)	C120—C119—H119	121.2
C8B—N2—Al3	130.0 (2)	C119—C120—C121	122.4 (16)
C8—N2—Al3	130.0 (2)	C119—C120—H120	118.8
O4—N2—Al3	117.50 (17)	C121—C120—H120	118.8
C15—N3—O7	110.6 (2)	C122—C121—C120	115.5 (15)
C15—N3—Al1	133.0 (2)	C122—C121—H121	122.3
O7—N3—Al1	116.37 (17)	C120—C121—H121	122.3
C29—N5—O13	117.0 (3)	C123—C122—C121	119.0 (15)
C29—N5—H5N	121.5	C123—C122—H122	120.5
O13—N5—H5N	121.5	C121—C122—H122	120.5
C36—N6—O16	110.2 (2)	C122—C123—N21	124.3 (15)
C36—N6—Al5	127.5 (2)	C122—C123—H123	117.9
O16—N6—Al5	119.0 (2)	N21—C123—H123	117.9
			
O24—Al1—O2—C1	159.11 (19)	C38—C37—C42—C41	2.0 (6)
O9—Al1—O2—C1	−111.88 (19)	C36—C37—C42—C41	−178.9 (4)
O1—Al1—O2—C1	−11.14 (19)	Al5—O20—C43—N7	14.2 (5)
N3—Al1—O2—C1	−43.3 (4)	Al5—O20—C43—C44	−164.5 (3)
N8—Al1—O2—C1	72.2 (2)	O19—N7—C43—O20	3.5 (5)
Yb1—Al1—O2—C1	9.6 (2)	Al6—N7—C43—O20	−165.9 (3)
O17—Al2—O3—C7	163.7 (3)	O19—N7—C43—C44	−177.8 (3)
O5—Al2—O3—C7	68.9 (3)	Al6—N7—C43—C44	12.8 (5)
O16—Al2—O3—C7	−114.3 (3)	O20—C43—C44—C45	−5.3 (6)
N1—Al2—O3—C7	−26.4 (3)	N7—C43—C44—C45	176.0 (4)
Yb1B—Al2—O3—C7	−80.3 (3)	O20—C43—C44—C49	175.3 (4)
Yb1—Al2—O3—C7	−75.5 (3)	N7—C43—C44—C49	−3.4 (6)
O17—Al2—O4—N2	−103.48 (17)	C49—C44—C45—C46	−0.6 (7)
O5—Al2—O4—N2	−9.37 (17)	C43—C44—C45—C46	180.0 (4)
O16—Al2—O4—N2	175.71 (17)	C44—C45—C46—C47	−1.1 (8)
N1—Al2—O4—N2	86.62 (17)	C45—C46—C47—C48	1.5 (8)
Yb1B—Al2—O4—N2	145.1 (2)	C46—C47—C48—C49	−0.3 (7)
Yb1—Al2—O4—N2	140.2 (2)	Al6—O21—C49—C44	−11.5 (5)
O17—Al2—O4—Yb1B	111.43 (11)	Al6—O21—C49—C48	169.7 (3)
O5—Al2—O4—Yb1B	−154.46 (11)	C45—C44—C49—O21	−177.0 (4)
O16—Al2—O4—Yb1B	30.61 (10)	C43—C44—C49—O21	2.4 (6)
N1—Al2—O4—Yb1B	−58.47 (11)	C45—C44—C49—C48	1.8 (6)
O17—Al2—O4—Yb1	116.36 (10)	C43—C44—C49—C48	−178.8 (4)
O5—Al2—O4—Yb1	−149.53 (10)	C47—C48—C49—O21	177.5 (4)
O16—Al2—O4—Yb1	35.54 (9)	C47—C48—C49—C44	−1.4 (6)
N1—Al2—O4—Yb1	−53.54 (10)	Al6—O23—C50—N8	0.6 (4)
O3—Al2—O5—C8B	−166.2 (2)	Al6—O23—C50—C51	176.2 (2)
O17—Al2—O5—C8B	101.8 (2)	O22—N8—C50—O23	1.9 (4)
O4—Al2—O5—C8B	8.6 (2)	Al1—N8—C50—O23	−166.6 (2)
O16—Al2—O5—C8B	22.7 (4)	O22—N8—C50—C51	−173.6 (3)
N1—Al2—O5—C8B	−77.3 (2)	Al1—N8—C50—C51	17.8 (4)
Yb1B—Al2—O5—C8B	−11.8 (2)	O23—C50—C51—C56	−167.8 (3)
O3—Al2—O5—C8	−166.2 (2)	N8—C50—C51—C56	7.8 (5)
O17—Al2—O5—C8	101.8 (2)	O23—C50—C51—C52	6.7 (5)
O4—Al2—O5—C8	8.6 (2)	N8—C50—C51—C52	−177.7 (3)
O16—Al2—O5—C8	22.7 (4)	C56—C51—C52—C53	4.3 (6)
N1—Al2—O5—C8	−77.3 (2)	C50—C51—C52—C53	−170.4 (4)
Yb1—Al2—O5—C8	−14.4 (2)	C51—C52—C53—C54	−0.6 (8)
C15—O8—Al4—O27	−164.1 (2)	C52—C53—C54—C55	−2.7 (7)
C15—O8—Al4—O11	109.1 (2)	C53—C54—C55—C56	2.2 (6)
C15—O8—Al4—O7	13.4 (2)	Al1—O24—C56—C51	−21.7 (4)
C15—O8—Al4—O10	16.1 (5)	Al1—O24—C56—C55	160.2 (2)
C15—O8—Al4—N9	−77.1 (2)	C52—C51—C56—O24	177.2 (3)
C15—O8—Al4—Yb1	−8.4 (2)	C50—C51—C56—O24	−8.3 (5)
Al4—O10—N4—C22	3.3 (4)	C52—C51—C56—C55	−4.7 (5)
Yb1—O10—N4—C22	115.4 (3)	C50—C51—C56—C55	169.8 (3)
O8—Al4—O11—C22	−159.8 (2)	C54—C55—C56—O24	179.7 (3)
O27—Al4—O11—C22	108.5 (3)	C54—C55—C56—C51	1.5 (5)
O7—Al4—O11—C22	−76.4 (3)	C68—N10—C64—C65	−0.5 (6)
O10—Al4—O11—C22	−0.5 (3)	Al3—N10—C64—C65	177.0 (4)
N9—Al4—O11—C22	40.8 (6)	N10—C64—C65—C66	−0.1 (8)
Yb1—Al4—O11—C22	−39.7 (3)	C64—C65—C66—C67	0.6 (9)
Al4—O11—C22—N4	2.5 (4)	C65—C66—C67—C68	−0.4 (8)
Al4—O11—C22—C23	−172.2 (3)	C64—N10—C68—C67	0.7 (6)
O10—N4—C22—O11	−4.0 (5)	Al3—N10—C68—C67	−176.7 (3)
O10—N4—C22—C23	170.6 (3)	C66—C67—C68—N10	−0.3 (7)
O11—C22—C23—C24	−3.4 (6)	C78—N12—C74—C75	−2.2 (7)
N4—C22—C23—C24	−177.8 (4)	N12—C74—C75—C76	0.8 (8)
O11—C22—C23—C28	171.5 (4)	C74—C75—C76—C77	1.8 (8)
N4—C22—C23—C28	−3.0 (6)	C75—C76—C77—C78	−2.9 (8)
C28—C23—C24—C25	0.0 (7)	C74—N12—C78—C77	1.1 (7)
C22—C23—C24—C25	175.1 (4)	C76—C77—C78—N12	1.5 (7)
C23—C24—C25—C26	−0.4 (8)	C83—N13—C79—C80	−0.4 (7)
C24—C25—C26—C27	0.0 (9)	N13—C79—C80—C81	0.2 (7)
C25—C26—C27—C28	0.6 (10)	C79—C80—C81—C82	0.2 (7)
C24—C23—C28—O12	−179.2 (4)	C80—C81—C82—C83	−0.5 (8)
C22—C23—C28—O12	6.0 (7)	C79—N13—C83—C82	0.1 (7)
C24—C23—C28—C27	0.6 (7)	C81—C82—C83—N13	0.3 (8)
C22—C23—C28—C27	−174.2 (4)	C88—N14—C84—C85	−3 (4)
C26—C27—C28—O12	178.8 (6)	N14—C84—C85—C86	6 (4)
C26—C27—C28—C23	−0.9 (8)	C84—C85—C86—C87	−2 (3)
O26—Al3—O25—N9	−13.5 (5)	C85—C86—C87—C88	−3 (3)
N2—Al3—O25—N9	165.4 (4)	C84—N14—C88—C87	−3 (4)
O28—Al3—O25—N9	77.2 (4)	C86—C87—C88—N14	6 (3)
N10—Al3—O25—N9	−104.3 (4)	C128—N22—C124—C125	−1 (4)
O26—Al3—O25—Yb1	−176.4 (5)	N22—C124—C125—C126	7 (4)
N2—Al3—O25—Yb1	2.5 (5)	C124—C125—C126—C127	3 (3)
O28—Al3—O25—Yb1	−85.8 (5)	C125—C126—C127—C128	−17 (3)
N10—Al3—O25—Yb1	92.8 (5)	C126—C127—C128—N22	23 (3)
Al3—O25—N9—C57	12.6 (6)	C124—N22—C128—C127	−14 (3)
Yb1—O25—N9—C57	178.1 (4)	C129—N15—C89—N23	0 (100)
Al3—O25—N9—Al4	−159.0 (3)	C133—N15—C89—N23	0 (100)
Yb1—O25—N9—Al4	6.5 (6)	C93—N15—C89—N23	0 (100)
O6—Al3—O26—C57	−163.7 (4)	C129—N15—C89—C90	−48 (9)
O25—Al3—O26—C57	12.0 (4)	C133—N15—C89—C90	34 (4)
O28—Al3—O26—C57	−74.0 (4)	C93—N15—C89—C90	−1 (4)
N10—Al3—O26—C57	104.5 (4)	C129—N15—C89—C130	−57 (10)
O25—N9—C57—O26	−3.3 (6)	C133—N15—C89—C130	26 (3)
Al4—N9—C57—O26	167.1 (4)	C93—N15—C89—C130	−9 (3)
O25—N9—C57—C58	177.7 (4)	N23—C89—C90—C129	62 (8)
Al4—N9—C57—C58	−11.9 (6)	N15—C89—C90—C129	62 (8)
Al3—O26—C57—N9	−8.0 (6)	C130—C89—C90—C129	112 (17)
Al3—O26—C57—C58	171.1 (3)	N23—C89—C90—C91	0 (5)
N9—C57—C58—C59	172.2 (4)	N15—C89—C90—C91	0 (5)
O26—C57—C58—C59	−6.9 (6)	C130—C89—C90—C91	50 (12)
N9—C57—C58—C63	−9.7 (6)	N23—C89—C90—C131	−33 (4)
O26—C57—C58—C63	171.1 (4)	N15—C89—C90—C131	−33 (4)
C63—C58—C59—C60	1.0 (7)	C130—C89—C90—C131	17 (12)
C57—C58—C59—C60	179.1 (4)	C129—C90—C91—C131	−93 (3)
C58—C59—C60—C61	−0.1 (7)	C89—C90—C91—C131	−85 (3)
C59—C60—C61—C62	0.0 (8)	C129—C90—C91—C130	−101 (8)
C60—C61—C62—C63	−1.0 (7)	C89—C90—C91—C130	−93 (9)
C61—C62—C63—O27	−177.8 (4)	C131—C90—C91—C130	−8 (8)
C61—C62—C63—C58	1.9 (6)	C129—C90—C91—C92	−6 (3)
C59—C58—C63—O27	177.7 (4)	C89—C90—C91—C92	2 (4)
C57—C58—C63—O27	−0.3 (6)	C131—C90—C91—C92	87 (2)
C59—C58—C63—C62	−1.9 (6)	C129—C90—C91—C132	−47 (3)
C57—C58—C63—C62	−179.9 (4)	C89—C90—C91—C132	−39 (3)
C62—C63—O27—Al4	−146.9 (3)	C131—C90—C91—C132	46.0 (13)
C58—C63—O27—Al4	33.5 (5)	C131—C91—C92—C132	9.0 (18)
O8—Al4—O27—C63	57.2 (3)	C130—C91—C92—C132	−68 (2)
O11—Al4—O27—C63	156.2 (3)	C90—C91—C92—C132	−78 (2)
O10—Al4—O27—C63	−122.9 (3)	C131—C91—C92—C93	83 (2)
N9—Al4—O27—C63	−40.3 (3)	C130—C91—C92—C93	6 (3)
Yb1—Al4—O27—C63	−82.3 (4)	C90—C91—C92—C93	−4 (3)
C73—N11—C69—C70	−1.2 (7)	C132—C91—C92—C93	73.7 (18)
Al6—N11—C69—C70	168.0 (4)	C130—C91—C92—C131	−77 (2)
N11—C69—C70—C71	−1.5 (8)	C90—C91—C92—C131	−87 (3)
C69—C70—C71—C72	2.4 (8)	C132—C91—C92—C131	−9.0 (18)
C70—C71—C72—C73	−0.8 (8)	C131—C91—C92—C133	58 (2)
C69—N11—C73—C72	2.9 (7)	C130—C91—C92—C133	−19 (3)
Al6—N11—C73—C72	−165.9 (4)	C90—C91—C92—C133	−29 (3)
C71—C72—C73—N11	−2.0 (8)	C132—C91—C92—C133	48.9 (11)
N3—O7—Al4B—O27B	1 (2)	C132—C92—C93—C133	6.3 (19)
Yb1B—O7—Al4B—O27B	−140 (2)	C91—C92—C93—C133	−74 (2)
N3—O7—Al4B—O11B	−106.9 (5)	C131—C92—C93—C133	−40 (2)
Yb1B—O7—Al4B—O11B	112.2 (4)	C132—C92—C93—N15	83.9 (18)
N3—O7—Al4B—O10B	171.4 (4)	C91—C92—C93—N15	4 (3)
Yb1B—O7—Al4B—O10B	30.5 (4)	C131—C92—C93—N15	38 (2)
N3—O7—Al4B—N9B	86.6 (6)	C133—C92—C93—N15	77.6 (19)
Yb1B—O7—Al4B—N9B	−54.3 (6)	C132—C92—C93—N23	83.9 (18)
N3—O7—Al4B—O8	−5.8 (4)	C91—C92—C93—N23	4 (3)
Yb1B—O7—Al4B—O8	−146.74 (15)	C131—C92—C93—N23	38 (2)
N3—O7—Al4B—Yb1B	140.9 (3)	C133—C92—C93—N23	77.6 (19)
Al4B—O10B—N4B—C22B	10.2 (17)	C91—C92—C93—C132	−79.9 (18)
Yb1B—O10B—N4B—C22B	112.9 (13)	C131—C92—C93—C132	−46.2 (12)
Al4B—O11B—C22B—N4B	−28.0 (18)	C133—C92—C93—C132	−6.3 (19)
Al4B—O11B—C22B—C23B	159.1 (14)	C89—N15—C93—C133	95 (2)
O10B—N4B—C22B—O11B	12 (2)	C129—N15—C93—C133	103 (2)
O10B—N4B—C22B—C23B	−175.1 (15)	C89—N15—C93—C92	−2 (3)
O11B—C22B—C23B—C24B	19 (3)	C129—N15—C93—C92	6 (2)
N4B—C22B—C23B—C24B	−154.1 (17)	C133—N15—C93—C92	−97 (2)
O11B—C22B—C23B—C28B	−156.3 (18)	C89—N15—C93—N23	0 (100)
N4B—C22B—C23B—C28B	31 (3)	C129—N15—C93—N23	0 (100)
C28B—C23B—C24B—C25B	2 (3)	C133—N15—C93—N23	0 (100)
C22B—C23B—C24B—C25B	−172.6 (18)	C89—N15—C93—C132	46 (2)
C23B—C24B—C25B—C26B	−6 (3)	C129—N15—C93—C132	53.9 (18)
C24B—C25B—C26B—C27B	15 (4)	C133—N15—C93—C132	−49.2 (16)
C25B—C26B—C27B—C28B	−18 (4)	N15—C89—N23—C129	0 (100)
C24B—C23B—C28B—O12B	176.3 (18)	C90—C89—N23—C129	−48 (9)
C22B—C23B—C28B—O12B	−9 (3)	C130—C89—N23—C129	−57 (10)
C24B—C23B—C28B—C27B	−5 (3)	N15—C89—N23—C133	0 (100)
C22B—C23B—C28B—C27B	169.4 (17)	C90—C89—N23—C133	34 (4)
C26B—C27B—C28B—O12B	−169 (2)	C130—C89—N23—C133	26 (3)
C26B—C27B—C28B—C23B	12 (3)	N15—C89—N23—C93	0 (36)
C23B—C28B—O12B—Al6	105 (2)	C90—C89—N23—C93	−1 (4)
C27B—C28B—O12B—Al6	−73 (2)	C130—C89—N23—C93	−9 (3)
Al3—O25B—N9B—C57B	16 (3)	C133—C93—N23—C89	95 (2)
Yb1B—O25B—N9B—C57B	178 (2)	C92—C93—N23—C89	−2 (3)
Al3—O25B—N9B—Al4B	−151.9 (14)	N15—C93—N23—C89	0 (100)
Yb1B—O25B—N9B—Al4B	10 (3)	C132—C93—N23—C89	46 (2)
O6B—Al3—O26B—C57B	−165.2 (17)	C133—C93—N23—C129	103 (2)
N2—Al3—O26B—C57B	29 (5)	C92—C93—N23—C129	6 (2)
O28—Al3—O26B—C57B	−74.4 (18)	N15—C93—N23—C129	0 (51)
O25B—Al3—O26B—C57B	16 (2)	C132—C93—N23—C129	53.9 (18)
N10—Al3—O26B—C57B	103.9 (18)	C92—C93—N23—C133	−97 (2)
O25B—N9B—C57B—O26B	−4 (3)	N15—C93—N23—C133	0 (100)
Al4B—N9B—C57B—O26B	162.6 (19)	C132—C93—N23—C133	−49.2 (16)
O25B—N9B—C57B—C58B	−178 (2)	C89—C90—C129—N23	−100 (11)
Al4B—N9B—C57B—C58B	−11 (3)	C91—C90—C129—N23	22 (5)
Al3—O26B—C57B—N9B	−11 (3)	C131—C90—C129—N23	−12 (5)
Al3—O26B—C57B—C58B	162.8 (17)	C89—C90—C129—N15	−100 (11)
N9B—C57B—C58B—C59B	174 (2)	C91—C90—C129—N15	22 (5)
O26B—C57B—C58B—C59B	−1 (3)	C131—C90—C129—N15	−12 (5)
N9B—C57B—C58B—C63B	−11 (3)	C89—C90—C129—C130	−71 (18)
O26B—C57B—C58B—C63B	175 (2)	C91—C90—C129—C130	50 (13)
C63B—C58B—C59B—C60B	1 (3)	C131—C90—C129—C130	17 (13)
C57B—C58B—C59B—C60B	177 (2)	C89—N23—C129—C90	113 (13)
C58B—C59B—C60B—C61B	0 (3)	C133—N23—C129—C90	11 (4)
C59B—C60B—C61B—C62B	−1 (3)	C93—N23—C129—C90	−22 (4)
C60B—C61B—C62B—C63B	1 (3)	C89—N23—C129—N15	0 (100)
C61B—C62B—C63B—O27B	−176.6 (19)	C133—N23—C129—N15	0 (100)
C61B—C62B—C63B—C58B	−1 (3)	C93—N23—C129—N15	0 (100)
C59B—C58B—C63B—O27B	175.0 (19)	C89—N23—C129—C130	107 (13)
C57B—C58B—C63B—O27B	−1 (3)	C133—N23—C129—C130	5 (4)
C59B—C58B—C63B—C62B	0 (3)	C93—N23—C129—C130	−28 (4)
C57B—C58B—C63B—C62B	−175.9 (19)	C89—N15—C129—C90	113 (13)
C62B—C63B—O27B—Al4B	−150.0 (15)	C133—N15—C129—C90	11 (4)
C58B—C63B—O27B—Al4B	35 (3)	C93—N15—C129—C90	−22 (4)
O7—Al4B—O27B—C63B	45 (3)	C89—N15—C129—N23	0 (100)
O11B—Al4B—O27B—C63B	153.4 (15)	C133—N15—C129—N23	0 (100)
O10B—Al4B—O27B—C63B	−125.0 (14)	C93—N15—C129—N23	0 (100)
N9B—Al4B—O27B—C63B	−41.5 (15)	C89—N15—C129—C130	107 (13)
O8—Al4B—O27B—C63B	51.5 (14)	C133—N15—C129—C130	5 (4)
Yb1B—Al4B—O27B—C63B	−81.3 (17)	C93—N15—C129—C130	−28 (4)
O3—Al2—O17—C36	123.0 (2)	C131—C91—C130—C129	−104 (3)
O4—Al2—O17—C36	−56.5 (2)	C90—C91—C130—C129	68 (8)
O5—Al2—O17—C36	−139.3 (2)	C92—C91—C130—C129	−23 (4)
O16—Al2—O17—C36	20.2 (2)	C132—C91—C130—C129	−60 (3)
N1—Al2—O17—C36	35.4 (7)	C90—C91—C130—C131	172 (8)
Yb1B—Al2—O17—C36	−14.4 (2)	C92—C91—C130—C131	82 (2)
Yb1—Al2—O17—C36	−16.3 (2)	C132—C91—C130—C131	44.2 (11)
O20—Al5—O18—C42	−106.7 (3)	C131—C91—C130—C89	−97 (3)
O13—Al5—O18—C42	77.0 (3)	C90—C91—C130—C89	75 (8)
O14—Al5—O18—C42	158.9 (3)	C92—C91—C130—C89	−16 (4)
N6—Al5—O18—C42	−6.6 (3)	C132—C91—C130—C89	−53 (3)
Yb1B—Al5—O18—C42	38.8 (4)	C90—C129—C130—C91	−118 (16)
Yb1—Al5—O18—C42	44.8 (4)	N23—C129—C130—C91	37 (5)
O18—Al5—O20—C43	157.8 (3)	N15—C129—C130—C91	37 (5)
O19—Al5—O20—C43	−19.3 (3)	C90—C129—C130—C131	−159 (17)
O13—Al5—O20—C43	−36.0 (6)	N23—C129—C130—C131	−4 (5)
O14—Al5—O20—C43	−110.8 (3)	N15—C129—C130—C131	−4 (5)
N6—Al5—O20—C43	67.4 (3)	C90—C129—C130—C89	113 (17)
Yb1B—Al5—O20—C43	4.3 (3)	N23—C129—C130—C89	−92 (9)
Yb1—Al5—O20—C43	−1.0 (3)	N15—C129—C130—C89	−92 (9)
O23—Al6—O21—C49	−165.1 (3)	N23—C89—C130—C91	18 (4)
N7—Al6—O21—C49	15.2 (3)	N15—C89—C130—C91	18 (4)
O12B—Al6—O21—C49	88.1 (5)	C90—C89—C130—C91	−117 (15)
O29—Al6—O21—C49	−72.3 (3)	N23—C89—C130—C129	72 (7)
N11—Al6—O21—C49	107.3 (3)	N15—C89—C130—C129	72 (7)
O23—Al6—O22—N8	3.12 (19)	C90—C89—C130—C129	−63 (17)
N7—Al6—O22—N8	−178.0 (2)	N23—C89—C130—C131	−24 (4)
O12B—Al6—O22—N8	109.3 (5)	N15—C89—C130—C131	−24 (4)
O29—Al6—O22—N8	−90.29 (19)	C90—C89—C130—C131	−159 (16)
N11—Al6—O22—N8	89.9 (2)	C90—C91—C131—C130	−1.1 (11)
O23—Al6—O22—Yb1	174.39 (16)	C92—C91—C131—C130	−122.6 (13)
N7—Al6—O22—Yb1	−6.74 (17)	C132—C91—C131—C130	−114.9 (13)
O29—Al6—O22—Yb1	80.98 (16)	C130—C91—C131—C132	114.9 (13)
N11—Al6—O22—Yb1	−98.85 (17)	C90—C91—C131—C132	113.8 (14)
O23—Al6—O22—Yb1B	166.74 (16)	C92—C91—C131—C132	−7.7 (15)
N7—Al6—O22—Yb1B	−14.39 (16)	C130—C91—C131—C90	1.1 (11)
O12B—Al6—O22—Yb1B	−87.1 (5)	C92—C91—C131—C90	−121.5 (11)
O29—Al6—O22—Yb1B	73.33 (15)	C132—C91—C131—C90	−113.8 (14)
O21—Al6—O23—C50	170.8 (2)	C130—C91—C131—C92	122.6 (13)
O22—Al6—O23—C50	−2.1 (2)	C90—C91—C131—C92	121.5 (11)
O12B—Al6—O23—C50	−96.9 (5)	C132—C91—C131—C92	7.7 (15)
O29—Al6—O23—C50	84.2 (2)	C129—C130—C131—C91	93 (3)
N11—Al6—O23—C50	−99.9 (2)	C89—C130—C131—C91	101 (3)
O2—Al1—O24—C56	−61.2 (2)	C91—C130—C131—C132	−91 (2)
O9—Al1—O24—C56	−150.0 (2)	C129—C130—C131—C132	2 (4)
N3—Al1—O24—C56	126.0 (2)	C89—C130—C131—C132	11 (4)
N8—Al1—O24—C56	34.3 (2)	C91—C130—C131—C90	−20 (25)
Yb1—Al1—O24—C56	69.1 (3)	C129—C130—C131—C90	72 (24)
Al1—O1—N1—C1	−11.5 (3)	C89—C130—C131—C90	81 (25)
Yb1—O1—N1—C1	−146.39 (18)	C91—C130—C131—C92	−47.8 (14)
Yb1B—O1—N1—C1	−146.99 (19)	C129—C130—C131—C92	45 (3)
Al1—O1—N1—Al2	169.30 (11)	C89—C130—C131—C92	54 (3)
Yb1—O1—N1—Al2	34.4 (2)	C129—C90—C131—C91	98 (3)
Yb1B—O1—N1—Al2	33.77 (19)	C89—C90—C131—C91	106 (2)
Al1—O1—N1—Yb1B	135.53 (16)	C129—C90—C131—C130	−103 (26)
Al1—O1—N1—Yb1	134.94 (17)	C89—C90—C131—C130	−95 (26)
Al2—O4—N2—C8B	8.6 (3)	C91—C90—C131—C130	159 (25)
Yb1B—O4—N2—C8B	138.7 (2)	C129—C90—C131—C132	7 (3)
Al2—O4—N2—C8	8.6 (3)	C89—C90—C131—C132	16 (3)
Yb1—O4—N2—C8	138.9 (2)	C91—C90—C131—C132	−90 (2)
Al2—O4—N2—Al3	−164.42 (12)	C129—C90—C131—C92	49 (2)
Yb1B—O4—N2—Al3	−34.4 (3)	C89—C90—C131—C92	58 (2)
Yb1—O4—N2—Al3	−34.1 (2)	C91—C90—C131—C92	−48.1 (13)
Al4B—O7—N3—C15	3.6 (4)	C132—C92—C131—C91	−169 (2)
Al4—O7—N3—C15	7.3 (3)	C93—C92—C131—C91	−112 (2)
Yb1—O7—N3—C15	138.1 (2)	C133—C92—C131—C91	−127.0 (18)
Yb1B—O7—N3—C15	140.3 (2)	C132—C92—C131—C130	−119.8 (17)
Al4B—O7—N3—Al1	−176.6 (3)	C93—C92—C131—C130	−62 (2)
Al4—O7—N3—Al1	−172.85 (12)	C91—C92—C131—C130	49 (2)
Yb1—O7—N3—Al1	−42.1 (2)	C133—C92—C131—C130	−77.6 (16)
Yb1B—O7—N3—Al1	−39.9 (2)	C93—C92—C131—C132	57.5 (14)
Al5—O13—N5—C29	7.8 (4)	C91—C92—C131—C132	169 (2)
Yb1B—O13—N5—C29	−104.4 (3)	C133—C92—C131—C132	42.2 (10)
Yb1—O13—N5—C29	−105.7 (3)	C132—C92—C131—C90	−121.1 (16)
Al2—O16—N6—C36	16.3 (3)	C93—C92—C131—C90	−63.6 (17)
Yb1B—O16—N6—C36	121.8 (2)	C91—C92—C131—C90	48.1 (15)
Yb1—O16—N6—C36	122.6 (2)	C133—C92—C131—C90	−78.9 (13)
Al2—O16—N6—Al5	−144.56 (13)	C93—C92—C132—C133	−3.3 (10)
Yb1B—O16—N6—Al5	−39.1 (2)	C91—C92—C132—C133	116.8 (12)
Yb1—O16—N6—Al5	−38.28 (19)	C131—C92—C132—C133	122.5 (13)
Al2—O16—N6—Yb1B	−105.48 (15)	C93—C92—C132—C131	−125.7 (12)
Al5—O19—N7—C43	−18.8 (3)	C91—C92—C132—C131	−5.6 (11)
Yb1B—O19—N7—C43	−134.3 (3)	C133—C92—C132—C131	−122.5 (13)
Yb1—O19—N7—C43	−143.1 (2)	C91—C92—C132—C93	120.1 (12)
Al5—O19—N7—Al6	151.64 (16)	C131—C92—C132—C93	125.7 (12)
Yb1B—O19—N7—Al6	36.2 (3)	C133—C92—C132—C93	3.3 (10)
Yb1—O19—N7—Al6	27.3 (3)	C93—C92—C132—C91	−120.1 (12)
Al6—O22—N8—C50	−3.5 (3)	C131—C92—C132—C91	5.6 (11)
Yb1—O22—N8—C50	−175.99 (19)	C133—C92—C132—C91	−116.8 (12)
Yb1B—O22—N8—C50	−167.85 (19)	C91—C131—C132—C92	9.6 (19)
Al6—O22—N8—Al1	165.47 (13)	C130—C131—C132—C92	75 (2)
Yb1—O22—N8—Al1	−7.0 (3)	C90—C131—C132—C92	72 (2)
Yb1B—O22—N8—Al1	1.1 (3)	C91—C131—C132—C133	−67 (3)
Al1—O2—C1—N1	7.9 (3)	C130—C131—C132—C133	−2 (3)
Al1—O2—C1—C2	−172.2 (2)	C90—C131—C132—C133	−5 (3)
O1—N1—C1—O2	2.5 (4)	C92—C131—C132—C133	−76.9 (19)
Al2—N1—C1—O2	−178.33 (19)	C91—C131—C132—C93	−38 (2)
Yb1B—N1—C1—O2	−45.6 (5)	C130—C131—C132—C93	28 (3)
Yb1—N1—C1—O2	−37.2 (4)	C90—C131—C132—C93	25 (3)
O1—N1—C1—C2	−177.3 (2)	C92—C131—C132—C93	−47.6 (12)
Al2—N1—C1—C2	1.8 (4)	C130—C131—C132—C91	66 (3)
Yb1B—N1—C1—C2	134.6 (3)	C90—C131—C132—C91	63 (2)
Yb1—N1—C1—C2	142.9 (2)	C92—C131—C132—C91	−9.6 (19)
O2—C1—C2—C3	−11.8 (4)	C133—C93—C132—C92	−173 (2)
N1—C1—C2—C3	168.1 (3)	N15—C93—C132—C92	−116.0 (14)
O2—C1—C2—C7	165.7 (3)	N23—C93—C132—C92	−116.0 (14)
N1—C1—C2—C7	−14.5 (4)	C92—C93—C132—C133	173 (2)
C7—C2—C3—C4	−2.2 (5)	N15—C93—C132—C133	56.9 (15)
C1—C2—C3—C4	175.3 (3)	N23—C93—C132—C133	56.9 (15)
C2—C3—C4—C5	−0.4 (6)	C133—C93—C132—C131	−113 (2)
C3—C4—C5—C6	2.2 (6)	C92—C93—C132—C131	59.8 (16)
C4—C5—C6—C7	−1.4 (6)	N15—C93—C132—C131	−56.2 (18)
Al2—O3—C7—C6	−159.4 (3)	N23—C93—C132—C131	−56.2 (18)
Al2—O3—C7—C2	22.3 (5)	C133—C93—C132—C91	−129.8 (19)
C5—C6—C7—O3	−179.5 (3)	C92—C93—C132—C91	43.1 (9)
C5—C6—C7—C2	−1.2 (5)	N15—C93—C132—C91	−72.9 (12)
C3—C2—C7—O3	−178.8 (3)	N23—C93—C132—C91	−72.9 (12)
C1—C2—C7—O3	3.8 (5)	C131—C91—C132—C92	−168 (2)
C3—C2—C7—C6	2.9 (5)	C130—C91—C132—C92	126.4 (19)
C1—C2—C7—C6	−174.5 (3)	C90—C91—C132—C92	119.5 (18)
Al2—O5—C8—N2	−6.2 (3)	C131—C91—C132—C133	124 (2)
Al2—O5—C8—C9	176.4 (3)	C130—C91—C132—C133	58 (2)
O4—N2—C8—O5	−1.6 (4)	C90—C91—C132—C133	51 (2)
Al3—N2—C8—O5	170.4 (2)	C92—C91—C132—C133	−68.3 (15)
O4—N2—C8—C9	175.8 (3)	C130—C91—C132—C131	−66 (2)
Al3—N2—C8—C9	−12.2 (5)	C90—C91—C132—C131	−72 (2)
O5—C8—C9—C10	−4.2 (6)	C92—C91—C132—C131	168 (2)
N2—C8—C9—C10	178.4 (4)	C131—C91—C132—C93	145 (2)
O5—C8—C9—C14	174.2 (4)	C130—C91—C132—C93	79.8 (17)
N2—C8—C9—C14	−3.3 (7)	C90—C91—C132—C93	72.9 (17)
C14—C9—C10—C11	0.3 (8)	C92—C91—C132—C93	−46.6 (11)
C8—C9—C10—C11	178.7 (5)	C92—C93—C133—N23	117.0 (11)
C9—C10—C11—C12	−0.5 (8)	N15—C93—C133—N23	0.000 (1)
C10—C11—C12—C13	0.1 (8)	C132—C93—C133—N23	122.3 (11)
C11—C12—C13—C14	0.4 (8)	C92—C93—C133—N15	117.0 (11)
C12—C13—C14—O6	179.4 (5)	N23—C93—C133—N15	0.000 (1)
C12—C13—C14—C9	−0.5 (7)	C132—C93—C133—N15	122.3 (11)
C10—C9—C14—O6	−179.7 (4)	C92—C93—C133—C132	−5.3 (15)
C8—C9—C14—O6	2.0 (8)	N15—C93—C133—C132	−122.3 (11)
C10—C9—C14—C13	0.2 (7)	N23—C93—C133—C132	−122.3 (11)
C8—C9—C14—C13	−178.1 (5)	N15—C93—C133—C92	−117.0 (12)
C13—C14—O6—Al3	−164.6 (3)	N23—C93—C133—C92	−117.0 (12)
C9—C14—O6—Al3	15.3 (6)	C132—C93—C133—C92	5.3 (16)
O26—Al3—O6—C14	156.4 (4)	C89—N23—C133—C93	−101 (2)
N2—Al3—O6—C14	−22.2 (3)	C129—N23—C133—C93	−91 (2)
O28—Al3—O6—C14	66.1 (3)	C89—N23—C133—N15	0 (100)
N10—Al3—O6—C14	−112.5 (3)	C129—N23—C133—N15	0 (100)
Al2—O5—C8B—N2	−6.2 (3)	C93—N23—C133—N15	0 (100)
Al2—O5—C8B—C9B	165.4 (19)	C89—N23—C133—C132	−15 (3)
O4—N2—C8B—O5	−1.6 (4)	C129—N23—C133—C132	−5 (3)
Al3—N2—C8B—O5	170.4 (2)	C93—N23—C133—C132	86 (2)
O4—N2—C8B—C9B	−173.7 (17)	C89—N23—C133—C92	−56.3 (18)
Al3—N2—C8B—C9B	−1.8 (18)	C129—N23—C133—C92	−45.8 (19)
O5—C8B—C9B—C10B	2 (4)	C93—N23—C133—C92	44.8 (14)
N2—C8B—C9B—C10B	173 (2)	C89—N15—C133—C93	−101 (2)
O5—C8B—C9B—C14B	177 (2)	C129—N15—C133—C93	−91 (2)
N2—C8B—C9B—C14B	−11 (4)	C89—N15—C133—N23	0 (100)
C14B—C9B—C10B—C11B	0 (5)	C129—N15—C133—N23	0 (100)
C8B—C9B—C10B—C11B	176 (3)	C93—N15—C133—N23	0 (100)
C9B—C10B—C11B—C12B	3 (4)	C89—N15—C133—C132	−15 (3)
C10B—C11B—C12B—C13B	−4 (4)	C129—N15—C133—C132	−5 (3)
C11B—C12B—C13B—C14B	3 (4)	C93—N15—C133—C132	86 (2)
C12B—C13B—C14B—O6B	−171 (2)	C89—N15—C133—C92	−56.3 (18)
C12B—C13B—C14B—C9B	0 (4)	C129—N15—C133—C92	−45.8 (19)
C10B—C9B—C14B—O6B	169 (3)	C93—N15—C133—C92	44.8 (14)
C8B—C9B—C14B—O6B	−7 (5)	C92—C132—C133—C93	6.0 (18)
C10B—C9B—C14B—C13B	−2 (5)	C131—C132—C133—C93	80 (3)
C8B—C9B—C14B—C13B	−178 (3)	C91—C132—C133—C93	54 (2)
C13B—C14B—O6B—Al3	−151.4 (17)	C92—C132—C133—N23	−71 (2)
C9B—C14B—O6B—Al3	38 (3)	C131—C132—C133—N23	3 (3)
O26B—Al3—O6B—C14B	144.8 (17)	C93—C132—C133—N23	−76.9 (18)
N2—Al3—O6B—C14B	−38.6 (15)	C91—C132—C133—N23	−23 (2)
O28—Al3—O6B—C14B	49.7 (15)	C92—C132—C133—N15	−71 (2)
N10—Al3—O6B—C14B	−128.9 (15)	C131—C132—C133—N15	3 (3)
Al4—O8—C15—N3	−13.7 (3)	C93—C132—C133—N15	−76.9 (18)
Al4B—O8—C15—N3	−7.4 (4)	C91—C132—C133—N15	−23 (2)
Al4—O8—C15—C16	164.3 (2)	C131—C132—C133—C92	74.3 (19)
Al4B—O8—C15—C16	170.6 (3)	C93—C132—C133—C92	−6.0 (18)
O7—N3—C15—O8	3.8 (4)	C91—C132—C133—C92	47.5 (10)
Al1—N3—C15—O8	−175.9 (2)	C132—C92—C133—C93	−172 (2)
O7—N3—C15—C16	−174.2 (2)	C91—C92—C133—C93	117 (2)
Al1—N3—C15—C16	6.0 (4)	C131—C92—C133—C93	143 (2)
O8—C15—C16—C17	11.2 (4)	C132—C92—C133—N23	125.3 (16)
N3—C15—C16—C17	−170.8 (3)	C93—C92—C133—N23	−62.5 (15)
O8—C15—C16—C21	−165.1 (3)	C91—C92—C133—N23	54.2 (17)
N3—C15—C16—C21	12.9 (4)	C131—C92—C133—N23	80.6 (13)
C21—C16—C17—C18	−2.7 (5)	C132—C92—C133—N15	125.3 (16)
C15—C16—C17—C18	−179.0 (3)	C93—C92—C133—N15	−62.5 (15)
C16—C17—C18—C19	−0.2 (5)	C91—C92—C133—N15	54.2 (17)
C17—C18—C19—C20	1.8 (5)	C131—C92—C133—N15	80.6 (13)
C18—C19—C20—C21	−0.6 (5)	C93—C92—C133—C132	172 (2)
Al1—O9—C21—C20	144.7 (2)	C91—C92—C133—C132	−71.1 (16)
Al1—O9—C21—C16	−37.9 (4)	C131—C92—C133—C132	−44.7 (11)
C19—C20—C21—O9	175.3 (3)	C143—N25—C139—C140	0.0
C19—C20—C21—C16	−2.2 (4)	N25—C139—C140—C141	0.0
C17—C16—C21—O9	−173.6 (3)	C139—C140—C141—C142	0.0
C15—C16—C21—O9	2.6 (4)	C140—C141—C142—C143	0.0
C17—C16—C21—C20	3.8 (4)	C141—C142—C143—N25	0.0
C15—C16—C21—C20	−179.9 (3)	C139—N25—C143—C142	0.0
Al5—O14—C29—N5	−1.0 (4)	C98—N16—C94—C95	0.7 (7)
Al5—O14—C29—C30	−179.3 (3)	N16—C94—C95—C96	−1.4 (7)
O13—N5—C29—O14	−4.6 (5)	C94—C95—C96—C97	1.4 (9)
O13—N5—C29—C30	173.6 (3)	C95—C96—C97—C98	−0.8 (11)
O14—C29—C30—C31	7.8 (6)	C94—N16—C98—C97	−0.1 (8)
N5—C29—C30—C31	−170.3 (4)	C96—C97—C98—N16	0.1 (10)
O14—C29—C30—C35	−174.5 (4)	C103—N17—C99—C100	−4 (4)
N5—C29—C30—C35	7.3 (6)	N17—C99—C100—C101	−4 (4)
C35—C30—C31—C32	1.0 (7)	C99—C100—C101—C102	15 (3)
C29—C30—C31—C32	178.8 (5)	C100—C101—C102—C103	−18 (3)
C30—C31—C32—C33	−1.2 (9)	C99—N17—C103—C102	1 (3)
C31—C32—C33—C34	0.7 (9)	C101—C102—C103—N17	9 (3)
C32—C33—C34—C35	−0.1 (9)	C138—N24—C134—C135	6 (3)
C31—C30—C35—O15	−179.5 (5)	N24—C134—C135—C136	0 (3)
C29—C30—C35—O15	2.9 (7)	C134—C135—C136—C137	−15 (3)
C31—C30—C35—C34	−0.4 (7)	C135—C136—C137—C138	23 (2)
C29—C30—C35—C34	−178.1 (4)	C134—N24—C138—C137	4 (3)
C33—C34—C35—O15	179.0 (5)	C136—C137—C138—N24	−17 (2)
C33—C34—C35—C30	0.0 (8)	C108—N18—C104—C105	−0.9 (9)
Al2—O17—C36—N6	−17.2 (4)	N18—C104—C105—C106	3.0 (9)
Al2—O17—C36—C37	163.0 (2)	C104—C105—C106—C107	−2.8 (9)
O16—N6—C36—O17	0.0 (4)	C105—C106—C107—C108	0.8 (9)
Al5—N6—C36—O17	158.8 (2)	C104—N18—C108—C107	−1.6 (11)
Yb1B—N6—C36—O17	52.2 (4)	C106—C107—C108—N18	1.5 (12)
O16—N6—C36—C37	179.8 (2)	C113—N19—C109—C110	1.6 (9)
Al5—N6—C36—C37	−21.4 (4)	N19—C109—C110—C111	−2.7 (9)
Yb1B—N6—C36—C37	−128.0 (3)	C109—C110—C111—C112	1.5 (7)
O17—C36—C37—C38	8.1 (5)	C110—C111—C112—C113	0.6 (7)
N6—C36—C37—C38	−171.7 (3)	C109—N19—C113—C112	0.9 (9)
O17—C36—C37—C42	−171.0 (3)	C111—C112—C113—N19	−1.9 (9)
N6—C36—C37—C42	9.2 (5)	C118—N20—C114—C115	−2.6 (10)
C42—C37—C38—C39	−1.3 (6)	N20—C114—C115—C116	2.4 (11)
C36—C37—C38—C39	179.6 (4)	C114—C115—C116—C117	−0.6 (10)
C37—C38—C39—C40	0.2 (8)	C115—C116—C117—C118	−0.9 (11)
C38—C39—C40—C41	0.2 (9)	C114—N20—C118—C117	1.0 (11)
C39—C40—C41—C42	0.5 (9)	C116—C117—C118—N20	0.8 (12)
Al5—O18—C42—C41	−179.7 (3)	C123—N21—C119—C120	−4 (3)
Al5—O18—C42—C37	−1.4 (6)	N21—C119—C120—C121	−9 (4)
C40—C41—C42—O18	176.8 (5)	C119—C120—C121—C122	11 (4)
C40—C41—C42—C37	−1.6 (7)	C120—C121—C122—C123	0 (2)
C38—C37—C42—O18	−176.3 (4)	C121—C122—C123—N21	−13 (4)
C36—C37—C42—O18	2.7 (5)	C119—N21—C123—C122	16 (4)

### Bis(pyridinium) diaquadipyridinehexakis[µ3-salicylhydroximato(3-)]bis[µ2-salicylhydroximato(1-)]hexaaluminiumytterbium–pyridine–water (1/7.386/1) (3) Hydrogen-bond geometry (Å, º)

**Table:**

*D*—H···*A*	*D*—H	H···*A*	*D*···*A*	*D*—H···*A*
N4—H4*N*···O12	0.88	1.95	2.593 (4)	129
O12—H12*O*···N15	0.84	1.79	2.578 (6)	156
C62—H62···O17^i^	0.95	2.52	3.331 (5)	144
O15—H15···N14	0.84	1.95	2.762 (14)	162
O15—H15···N22	0.84	1.70	2.526 (16)	168
O28—H28*A*···N13	0.84 (2)	1.80 (2)	2.638 (4)	177 (4)
O29—H29*A*···O16	0.91 (2)	1.82 (2)	2.702 (3)	163 (5)
O29—H29*B*···N18	0.94 (2)	1.88 (3)	2.698 (5)	144 (4)
O30—H30*A*···N19	0.87 (2)	2.12 (2)	2.828 (8)	138 (4)
O30—H30*B*···N20	0.87 (2)	2.17 (2)	3.020 (12)	167 (10)
N5—H5*N*···O15	0.88	1.98	2.635 (4)	130
C68—H68···O4	0.95	2.30	3.078 (4)	139
N12—H12*A*···O9	0.88	1.78	2.612 (4)	157
C74—H74···O28	0.95	2.48	3.407 (5)	164
C130—H130···O18^ii^	0.95	2.49	3.306 (19)	144
N16—H16···O27	0.88	1.77	2.637 (4)	170
C94—H94···O3^i^	0.95	2.28	3.150 (5)	152

Symmetry codes: (i) *x*+1, *y*, *z*; (ii) −*x*, −*y*+1, −*z*+1.
